# Washing machine ownership and girls’ school attendance: a cross-sectional analysis of adolescents in 19 middle-income countries

**DOI:** 10.1007/s10888-023-09612-7

**Published:** 2024-01-27

**Authors:** Omar Karlsson, Jan-Walter De Neve

**Affiliations:** 1 Visiting Research Fellow, Duke University Population Research Institute, Duke University, 140 Science Dr, Durham, NC 27710, USA; 2 Centre for Economic Demography, School of Economics and Management, Lund University, P.O. Box 7083, 220 07 Lund, Sweden; 3 Heidelberg Institute of Global Health, Medical Faculty and University Hospital, University of Heidelberg, Im Neuenheimer Feld 130.3, 69120 Heidelberg, Germany

**Keywords:** School attendance, Household work, Washing machine, Middle-income countries

## Abstract

Excessive work among adolescents may compromise educational development. Without home appliances, household work can take over 50 h a week and an additional 30 h when an infant is present. School-aged girls are often tasked with doing laundry, which is time-consuming and inflexible without a washing machine. We determined the association between washing machine ownership and school attendance among adolescents ages 10–19 years in 19 middle-income countries between 2000 and 2021 (*N* = 1,622,514). We controlled for socioeconomic and demographic characteristics, all neighborhood-level factors, and examined differences by sex, age, household wealth, and period. No relationship between washing machine ownership and school attendance was found in most countries: However, there was a substantial association for girls in Türkiye and a small to moderate association for girls in Egypt and Albania. In Türkiye, for example, girls living in households with a washing machine had 28% (95% CI 19, 37) greater school attendance compared to girls living in households which did not. No association was observed for boys. The results suggest that household ownership of a washing machine does generally not improve school attendance among girls, except possibly in specific contexts.

## 1 Introduction

Schooling is widely held as a cornerstone of economic as well as human development (De Neve et al. 2020). For individuals, acquiring education improves their own and their children’s income, health, and well-being (Karlsson et al. 2018; De Neve et al. 2015). An educated population helps increase entrepreneurial activity, economic growth, and tax revenue to finance essential public infrastructure. It also provides skills for filling the increasingly complex roles countries need to stay competitive and attain the highest living standards for their citizens. Girls’ education has historically lagged behind that of boys and continues to do so in many countries (UNESCO 2022), while female education is believed to hold particular potential, for example, for improving child health (Caldwell 1979). Therefore, numerous development goals center on education with a special focus on girls (WHO 2015).

UNESCO (2022) estimated that 89% of the relevant age group was enrolled in primary education in 2018 globally. However, the enrolment rate fell to 66% for secondary school. Many factors contribute to the fall in enrollment after primary school, such as poor school quality, long travel times from the household to school, and lack of finances for school fees. Poverty is an underlying cause (Jabbarian et al. 2022). “Child labor”—defined as “work that deprives children of their childhood, their potential and their dignity, and that is harmful to physical and mental development” and “interferes with their schooling”—is another common consequence of poverty that negatively affects schooling in many countries (ILO 2022b; Putnick and Bornstein 2015). School-aged girls are more often tasked with work inside the home—such as cooking, cleaning, and laundry—while boys work outside the home or in family production (i.e., on a family farm), mirroring other gendered work.

Household appliances can potentially reduce the workload from household work (Cowan 1983; Mokyr 2000) and therefore reduce the need for employing children within the household, enabling them to attend school. For example, in a study from China, 12–18-year-old girls spent 102 min less on household work per week and were 17% more likely to attend school when living in households with a washing machine, while the effect was much less pronounced among boys (Kerr 2019). In India, ownership of time-saving household appliances, such as a refrigerator, increased school enrollment and decreased employment rates among adolescents ages 12–18 years (Bhargava and Kerr 2021). In addition to reducing the time spent on household chores, washing machines can also increase school attendance by removing microorganisms and thereby improving children’s physical health – e.g., through washing clothes at 130 degrees Fahrenheit or higher, particularly in contexts where infectious diseases are common among school-age children (Karlsson et al. 2020; Shi et al. 2022).

The importance of household appliances for girls’ school attendance is likely to be highly context-dependent and vary according to gender norms, school access, and other factors, such as school quality, fertility, and the need for labor within the home. However, to our knowledge, no study has comprehensively examined the quantitative relationship between ownership of a washing machine and school attendance across a wide range of contexts. For example, no similar studies have been identified from the African region, where children relatively frequently engage in domestic work at an early age and school attendance remains persistently low (Graetz et al. 2020). To begin to address this gap in the literature, this study therefore examined the association between ownership of a washing machine and school attendance using nationally representative data on 1.6 million adolescents living in 19 middle-income countries between 2000 and 2021. We studied heterogeneity in the association across sex, age, household wealth, and year and adjusted for socioeconomic and demographic characteristics as well as all observed and unobserved factors at the level of the adolescent’s neighborhood or village.

## 2 Data and methods

### 2.1 Data

The empirical analyses presented in this paper were based on individual-level data from the nationally representative Demographic and Health Surveys (DHS), which are conducted regularly in numerous low- and middle-income countries. Standardized questionnaires and measures were used so that the surveys are comparable between countries and survey years. The sampling was based on stratified multi-stage sampling. Stratification was usually done by geographic or administrative sub-national regions crossed with the type of residence (urban or rural). Primary sampling units, usually neighborhoods or villages (often the enumeration areas of the most recent census), were sampled within each stratum based on a probability proportional to population size. Households were sampled from the primary sampling units. Several questionnaires are administered in each household. This study only used data from the household schedule, which collects basic information about each household member—such as age, sex, and education—and the household questionnaire, which collects basic information about the household, such as ownership of certain assets (e.g., washing machine, refrigerator, television) and amenities (e.g., electricity, type of toilet).

The DHS response rates have been very high, typically exceeding 90% (Corsi et al. 2012). Non-responses are not replaced, and smaller population segments are oversampled to acquire adequate observations for analysis. Sampling weights—calculated as the inverse probability of being included in the survey—are provided to adjust for oversampling and non-responses and to improve precision (Aliaga and Ren 2006). Additional information on the DHS surveys is available elsewhere (Aliaga and Ren 2006; Corsi et al. 2012), as well as DHS final country reports (DHS 2022).

### 2.2 Study population and inclusion criterion

Following the WHO (2022) definition of adolescents, we included 10–19-year-olds. Information on ownership of washing machines is not a part of the core questionnaire in the DHS but is sometimes added in the surveys to meet local conditions and needs (e.g., washing machine ownership was presumably added according to country income level to obtain appropriate data for constructing an asset index). All surveys including this information were considered for inclusion. The analysis was restricted to countries where at least 5% and at most 95% of the analytical sample attended school or had a washing machine to ensure sufficient variation in the outcome and exposure variables.

Only usual residents of interviewed households were considered. The complete sample consisted of 1,622,514 adolescents in 38 surveys from 19 countries: Albania, Armenia, Azerbaijan, Colombia, Egypt, Gabon, Guatemala, Guyana, India, Indonesia, Kyrgyz Republic, Moldova, Morocco, Pakistan, Peru, Philippines, South Africa, Tajikistan, and Türkiye (Supplementary Table S1 and S2). These countries were all classified by the World Bank as middle-income at or around the time when surveyed. A few countries were still classified as low-income at the time of survey—specifically, Pakistan in the earliest of three surveys, conducted in 2006 (became a middle-income country in 2008); Tajikistan in the earlier of two surveys, conducted in 2012 (became a middle-income country in 2014); the Kyrgyz Republic, which was surveyed only in 2012 (became a middle-income country in 2013); and Moldova, which was only surveyed in 2005 (became a middle-income country in 2006). The number of surveys for each country ranged from one to five surveys. The earliest survey was conducted in 2000, and the most recent survey was in 2019–21. Most countries had more than one survey, with Egypt having the most surveys (five).

We excluded observations with missing values for school attendance (*n* = 1,213), washing machine (hereafter also called washer) ownership (*n* = 538), covariates (*n* = 6,222), and, lastly, neighborhoods with only a single valid observation (*n* = 758) since at least two observations are needed within a neighborhood when comparing neighbors (see Section 2.6 for more information on this criterion). The remaining sample for analysis comprised 1,614,264 adolescents 10–19 years old (Supplementary Table S1). The smallest analytical sample was from Moldova (*n* = 5,457 from a single survey from 2010) and the largest was from India (*n* = 1,075,968 from two surveys conducted in 2015–16 and 2019–21).

### 2.3 Exposure

The main independent variable of interest was a binary indicator for whether the household where the adolescent resided owned a washing machine. Most surveys did not clearly distinguish whether the question referred to automatic or manual washing machines. However, the Integrated Public Use Microdata Series (IPUMS) (2022) indicates that these variables refer to an automatic washing machine. Further, survey reports suggest that these data refer to automatic washing machines, calling them “electrical appliances,” “modern conveniences,” “appliances,” and “durable goods” in their final reports (DHS 2022). As a sensitivity analysis, described below, we also restrict the analytical sample to adolescents living in households with access to electricity since electricity access is required for automatic washing machines (Supplementary Fig. S16 and Table S24).

We focused on washing machines rather than other time-saving home appliances for the following reason: doing laundry is a particularly time-consuming and inflexible work, commonly done by adolescent girls, which often needs to be done by hand after fetching water or traveling to a water source in the mornings (during school hours) to be dry and ready by night time (Assaad et al. 2010). Data on the number of hours spent doing domestic and economic activities in the past week was not consistently available across surveys. We were, therefore, unable to study the association between domestic and economic activities of adolescents, such as doing laundry or farming, and school attendance.

### 2.4 Outcome

The main outcome studied was a binary indicator for whether an adolescent 10–19 years old attended school during the school year in which the survey took place, constructed from the household schedule, which collects basic information on all household members. The variable was created from the question: “Did [NAME] attend school or any early childhood education program at any time during the [YEAR] school year?” In addition, the total number of school years completed in the survey year was studied as an outcome (see Section 2.8 below).

### 2.5 Control variables

Control variables were added to account for demographic, socioeconomic, and spatial differences which may confound the relationship between washer ownership and school attendance, including fridge ownership, TV ownership, having a flush toilet, a wealth index z-score (linear and squared), number of household members (linear and squared), number of household members under the age of five (linear and squared), age of the adolescent (linear and squared), highest education level of an adult male in the household, and highest education level of an adult female in the household, as well as all neighborhood-level factors. The highest education levels of adult males and females in the household only referred to adults above age 25 and were categorized as 1) no education, 2) primary, 3) secondary, 4) higher, and 5) no adult male/female in the household. The household wealth index—constructed using principal component analysis of various assets and amenities—was provided with the DHS datasets. The wealth index factor scores were converted into survey-specific z-scores by subtracting an overall (i.e., including all households in a survey) weighted mean and dividing by the standard deviation.

The wealth index was constructed including washing machine ownership and its inclusion as an independent variable might therefore plausibly control away some of the effect of washing machine ownership on school attendance. Washing machine ownership was, however, only one of many variables used to construct the household wealth index, and individual items generally do not contribute much to the index (Howe et al. 2012). Nevertheless, we addressed this potential concern in the Section 2.8 where we excluded wealth index from our models.

Further, controlling for having a flush toilet, fridge, and TV at home could be problematic due to multicollinearity or bad control problems—which would also obscure an effect of washing machine ownership on school attendance—since a large majority of those who own a washing machine also own a TV and a fridge, and most also have a flush toilet (Supplementary Table S3). However, calculating the variance inflation factor for the main terms in our main model does not indicate serious multicollinearity (Supplementary Table S4). This issue was also addressed with a sensitivity analysis where these covariates were excluded from the models (shown in the Section 2.8).

### 2.6 Analysis

Our analysis proceeded in two steps. First, descriptive statistics show the proportion of adolescents attending school (by sex) and living in households with washing machines. We also show how those proportions and ownership of other everyday household items have evolved over time, in the Supplement.

Second, modified Poisson regression models (i.e., with sandwich standard errors) were used to estimate the association between washer ownership and school attendance (Zou 2004; Zou and Donner 2013). All models estimated school attendance (y) for an adolescent (i) in neighborhood (n) in terms for being female (f), washer ownership, $\left(x_{1}\right)$ and interaction between being female and washer ownership $\left(f\times x_{1}\right)$, as well as control variables $\left(x_{2}\ldots x_{J}\right)$, all also interacted with being female $\left(f\times x_{j}\right)$.

(1) $$Poisson\left(y_{in}=1\right)=\alpha +\beta f_{in}+\sigma {\bar{f}}_{n}+\sum_{j=1}^{J}\left[\rho_{j}{\left(f\times x_{j}\right)}_{in}+\pi_{j}x_{j,in}+\gamma_{j}{\left(\bar{f\times x_{j}}\right)}_{n}+\tau_{j}{\bar{x}}_{j,n}\right]$$

The model was adjusted for neighborhood-level factors by adding a (weighted) neighborhood-level mean for all independent variables including interaction terms ($\overset{-}{f\times x},\;\overset{‾}{x}$, and $\overset{‾}{f}$) as independent variables (Schunck and Perales 2017; Wooldridge 2019). The neighborhoods (or villages in rural areas) were indicated by the primary sampling units. Note that were EQ1 estimated using the same variables but a linear regression instead of Poisson regression, all parameters below the neighborhood level would be identical if dummy coded variables for neighborhoods were added as independent variables instead of the neighborhood level means of all the independent variables (although the former approach might not be computationally feasible due to the high number of neighborhoods). However, since these are estimated using Poisson models, the parameters differ between the two approaches, most likely only slightly, but the interpretation remains the same.

The exponent of the coefficients for washer ownership $\left(exp\left(\pi_{1}\right)\right)$ gives the rate ratios for males, showing the relative difference in school attendance for males with a washer at home compared to males without (i.e., school attendance for those with a washer divided by that of those without a washer). A rate ratio of one means an equal rate. A rate ratio above one means greater school attendance for those with a washer at home (e.g., a rate ratio of 1.2 means 1.2 times the rate or 20% greater rate). A rate ratio below one means a lower rate for those with a washer at home (e.g., a rate ratio of 0.4 means 0.4 times the rate or a 60% lower rate). The exponentiated coefficient for the interaction between being female and having a washer $\left(exp\left(\rho_{1}\right)\right)$ shows the relative difference in rate ratio for having a washer for females compared to males. Multiplying the exponentiated coefficient for washer ownership by the exponentiated interaction term $\left(exp\left[\rho_{1}\right]\times exp\left[\pi_{1}\right]\right.$ or $exp\left[\rho_{1}+\pi_{1}\right]$) gives the rate ratio for females (done using post-estimation following Eq. 1).

Except for the term for being a female, all independent variables, including the neighborhood-level averages, were centered around a (weighted) mean. The exponentiated constant (exp(α)), therefore, shows school attendance for males with the mean on all other independent variables. Multiplying the exponentiated constant by the exponentiated coefficient for females (exp(α)×exp(β) or exp(α+β)) gives the school attendance for females (done using post-estimation following Eq. 1).

Estimates were weighted using sampling weights rescaled to sum up to one in each survey. Each survey, therefore, contributed equally to the country estimates rather than being affected by differences in sample sizes between surveys or changes in the underlying population size. Weights for pooled estimates were further rescaled such that each country contributed equally to these estimates. Robust standard errors used to construct 95% confidence intervals were adjusted for clustering at the level of primary sampling units.

### 2.7 Stratification

Results were further stratified by age groups (10–13 years, 14–16 years, and 17–19 years) and survey year categories. The stratification across survey years was only done for countries with more than one survey. Since the year of the survey and length of time between surveys varied, the periodization was not the same in all countries: The surveys fell in three periods: 2000–08, 2008–2016, and 2013–2021 (see Supplementary Table S5). At most three periods were constructed for each country, even for countries with more than three surveys. Finally, results were stratified across terciles of the household wealth index (i.e., dividing the final analytical sample for each country into three equally sized groups according to the household wealth index).

### 2.8 Supplementary and sensitivity analyses

First, we compared the role of household ownership of a washing machine with other household assets and utilities with varying relevance for adolescents’ time-use and school attendance. To do so, we considered the relationship between school attendance and the presence in the household of 1) a refrigerator (Supplementary Fig. S3 and Table S11), 2) piped drinking water (Supplementary Fig. S4 and Table S12), 3) electricity (Supplementary Fig. S5 and Table S13), 4) a car (Supplementary Fig. S6 and Table S14), 5) a motorbike (Supplementary Fig. S7 and Table S15), 6) clean cooking fuel (Supplementary Fig. S8 and Table S16), 7) a flush toilet (Supplementary Fig. S9 and Table S17), and, lastly, 8) a TV (Supplementary Fig. S10 and Table S18). Most of these items could have an impact on school attendance through similar pathways as washer ownership, that is by reducing the burden from household work (Supplementary Appendix S1). Further, a potential impact may differ between boys and girls if the type of work that is reduced is gendered. However, ownership of a TV seems unlikely to affect school attendance differentially between girls and boys—while still being linked to household wealth—and can serve as a placebo test.

Two, estimates were obtained using logit regression models (Supplementary Fig. S11 and Table S19) and linear regression models (Supplementary Fig. S12 and Table S20) instead of Poisson regression models. Three, control variables for the presence of a flush toilet, fridge, and TV in the household (Supplementary Fig. S13 and Table S21) and the control variable for household wealth index z-score (Supplementary Fig. S14 and Table S22) were excluded from the models. Four, the sample was restricted to descendants of the household head since others could potentially include spouses or staff (Supplementary Fig. S15 and Table S23). Five, we restricted the sample to households with access to electricity (Supplementary Fig. S16 and Table S24). Six, the results were stratified according to the overall share of adolescents attending school (Supplementary Fig. S17 and Table S25). Seven, the association of washer ownership with the years of education the adolescent had completed in the year of the survey was estimated, overall (Supplementary Fig. S18 and Table S26) and stratified across survey years (Supplementary Fig. S19 and Table S27).

Eight, the results were stratified according to the extent of female disadvantage in school attendance at the regional level (Supplementary Fig. S20 and Table S28). Nine, results were obtained from a model adjusting for household level factors, estimating the difference in the school attendance of boys and girls within the same households, in households with and without a washer (Supplementary Fig. S21 and Table S29). The models included baseline terms only for being female and age (since these were the only independent variables that varied within households), as well as interaction terms for female with age and all other independent variables.

See Supplementary Appendix S1 for more details and results from supplementary and sensitivity analyses.

## 3 Results

### 3.1 Descriptive statistics

Surveys with available data were conducted from 2000–2021 (Table 1). The share of adolescents living in households with a washing machine ranged from 0.0722 (95% CI 0.0508, 0.0936) in Gabon (surveyed in 2012) to 0.866 (95% CI 0.852, 0.879) in Türkiye (surveyed in 2003–04, 2008, 2013). Washer ownership rose over time in all countries with more than one survey, except Egypt (Supplementary Fig. S2 and Table S5). The increase was substantial in many countries, for example, Türkiye—where 0.73 (95% CI 0.71, 0.76) of adolescents had a washer at home in 2003–04, which rose to 0.96 (95% CI 0.95, 0.97) in 2013—and Colombia, where ownership increased from 0.24 (95% CI 0.23, 0.26) in 2004–05 to 0.62 (95% CI 0.60, 0.64) in 2015–16. Ownership of other household appliances, such as fridges and especially TVs, was greater than washers in almost all countries.

The share of adolescents 10–19 years attending school during the survey year ranged from 0.515 (95% CI 0.50, 0.53) in Pakistan (where surveys were conducted 2006–07, 2012–13, and 2017–18) to 0.91 (95% CI 0.90, 0.92) in the Kyrgyz Republic (from one survey, conducted in 2012). A lower share of girls attended school in most countries, particularly in Pakistan (− 0.116; 95% CI − 0.129, − 0.103), Azerbaijan (− 0.0879; 95% CI − 0.11, − 0.0658), Tajikistan (−0.0868; 95% CI − 0.102, − 0.0714), Morocco (− 0.0835; 95% CI − 0.103, − 0.0637), Guatemala (− 0.0766; 95% CI − 0.0921, − 0.061), and Türkiye (− 0.0678; 95% CI − 0.0823, − 0.0534). A greater share of girls than boys attended school in Moldova (0.0496; 95% CI 0.03, 0.0692), the Philippines (0.0467; 95% CI 0.0358, 0.0576), Indonesia (0.0194; 95% CI 0.00795, 0.0308), and Colombia (0.0165; 95% CI 0.00696, 0.026). School attendance increased over time in all countries with more than one survey, except for girls in Guyana, where it decreased somewhat between the two surveys conducted there, in 2005 and 2009 (Supplementary Fig. S1 and Table S5).

### 3.2 Main results

**Fig. 1:**
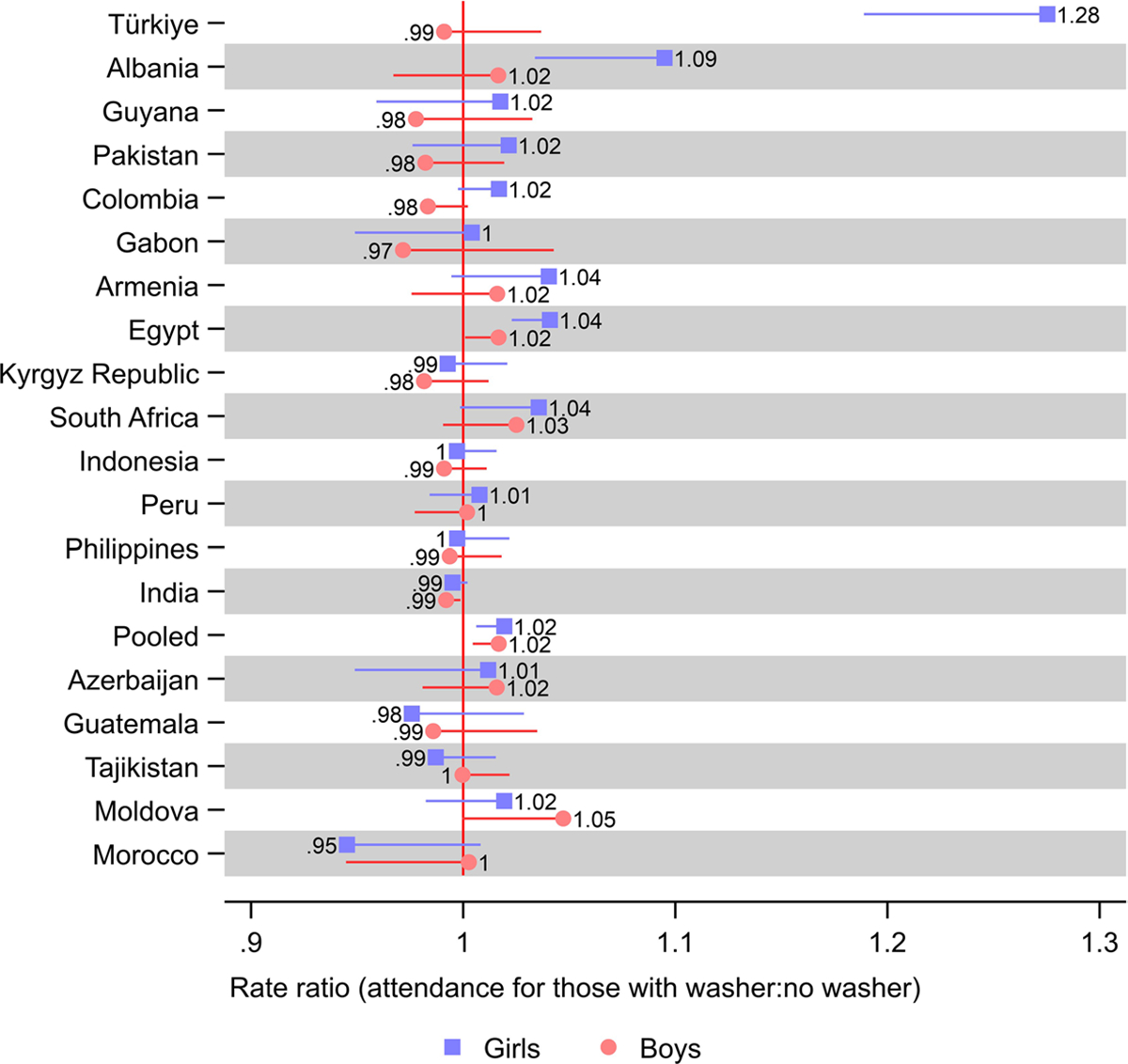
Results from poisson regression models of school attendance on washer ownership. Notes: Countries were ordered according to the relative difference in rate ratio between boys and girls. 95% confidence intervals are shown. Estimates were weighted using sampling weights rescaled to sum up to one in each survey. Pooled models were further rescaled such that each country contributed equally to the estimates. All models included a baseline term for being female and baseline terms and interactions with being female for washer ownership, fridge ownership, TV ownership, having flush toilet, a wealth index z-score, number of household members, number of household members under age five, age, highest education level of a male in household, and highest education level of a female in household, as well as adjusting for neighborhood. Upper confidence limits were omitted for estimates above one and lower confidence limits were omitted for estimates below one, for improved readability. See Supplementary Table S7 for tabulated estimates

The difference in school attendance between adolescents in households with and without a washer was the largest for girls in Türkiye, where those in households with a washing machine at home had a 28% greater school attendance, indicated by a rate ratio (RR) of 1.28 (95% CI 1.19, 1.37) (Fig. 1; Supplementary Tables S6 and S7 show the full model outputs and tabulated estimates). Girls with a washer at home were 9% (RR 1.09; 95% CI 1.03, 1.16) more likely to attend school than girls without a washer at home in Albania and 4% (RR 1.04; 95% CI 1.02, 1.06) in Egypt.

The difference was not statistically significant (at a 5% level) for the other sixteen countries studied. However, boys with a washer at home were 5% (RR 1.05; 95% CI 0.9995, 1.10) more likely to attend school than boys that did not in Moldova (although the difference was only statistically significant at a 10% level). Girls in South Africa and Armenia had a 4% (RR 1.04; 95% CI 0.999, 1.07 and 0.994, 1.088) greater school attendance, and boys in South Africa had a 3% (RR 1.03; 95% CI 0.99, 1.06) greater school attendance when living in households with a washer, although none of these differences were statistically significant. In Morocco, girls with a washer at home were 5% (RR 0.95; 95% CI 0.89, 1.01) less likely to attend school than those without a washer, although the difference was not statistically significant.

The interaction terms indicating the relative difference in the association of washer ownership between boys and girls was only statistically significant in Türkiye (1.29; 95% CI 1.19, 1.39), Albania (1.08; 95% CI 1.01, 1.16), Colombia (1.03; 95% CI 1.009, 1.06), and Egypt (1.02; 95% CI 1.003, 1.05) (Supplementary Table S7).

### 3.3 Results stratified by survey year

**Fig. 2:**
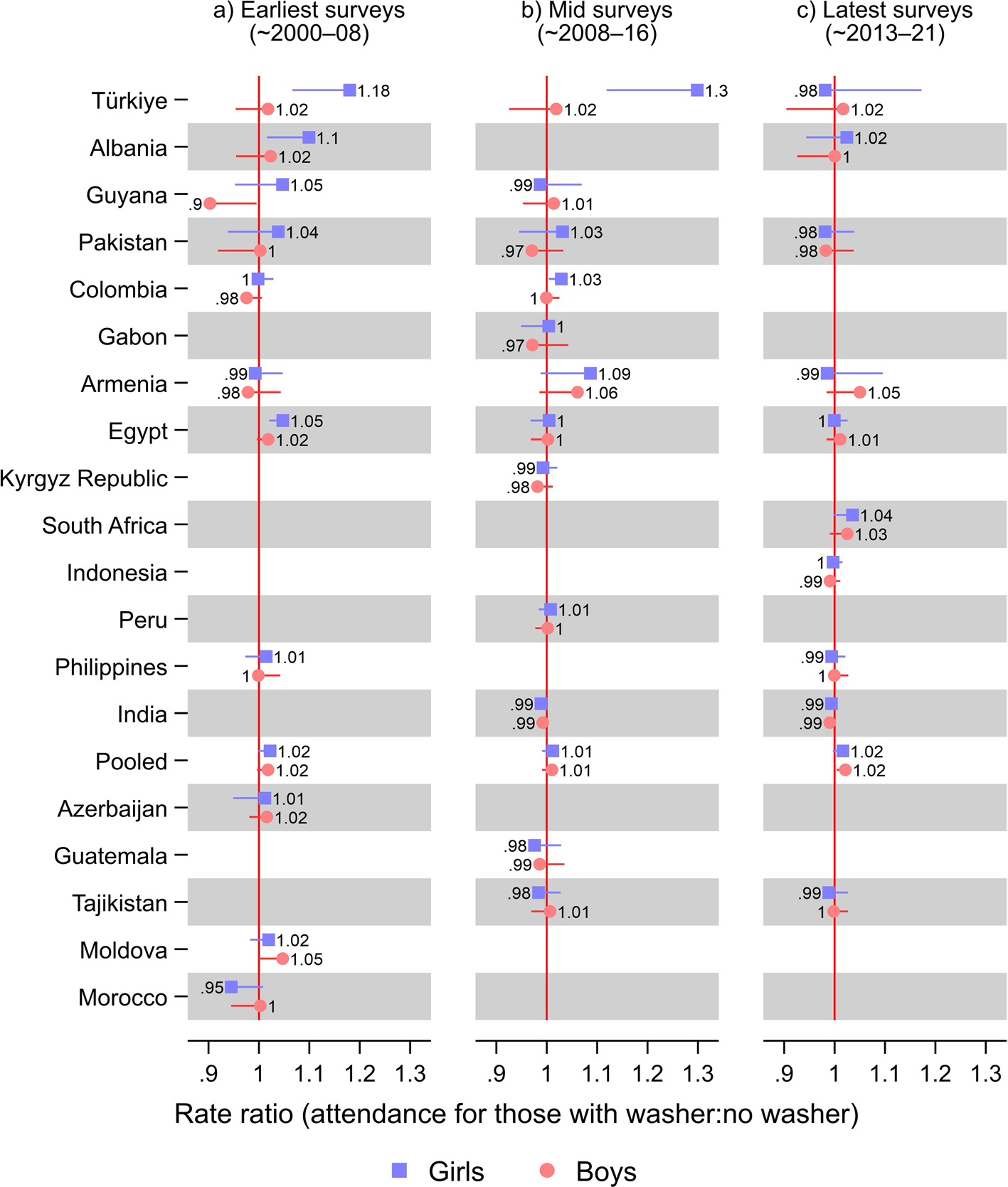
Results from poisson regression models of school attendance on washer ownership: stratified by survey year. Notes: Countries were ordered according to the relative difference in rate ratio between boys and girls from Fig. 1. 95% confidence intervals are shown. Estimates were weighted using sampling weights rescaled to sum up to one in each survey. Pooled models were further rescaled such that each country contributed equally to the estimates. All models included a baseline term for being female and baseline terms and interactions with being female for washer ownership, fridge ownership, TV ownership, having flush toilet, a wealth index z-score, number of household members, number of household members under age five, age, highest education level of a male in household, and highest education level of a female in household, as well as adjusting for neighborhood. Upper confidence limits were omitted for estimates above one and lower confidence limits were omitted for estimates below one, for improved readability. See Supplementary Table S8 for tabulated estimates

In Türkiye, in the earliest survey period (2003–04), girls with a washer at home had 18% (RR 1.18; 95% CI 1.07, 1.31) greater school attendance than girls without a washer at home and 30% (RR 1.30; 95% CI 1.12, 1.51) greater in the middle survey period (2008; Fig. 2: Supplementary Table S8 shows tabulated estimates). In the most recent survey for Türkiye (2013), however, that difference between girls and boys disappeared, and girls with a washer at home had a non-statistically significant 2% (RR 0.98; 95% CI 0.82, 1.17) lower attendance than girls without a washer. Note that the proportion with a washer at home had reached 0.96 (95% CI 0.95, 0.97) in Türkiye in 2013 (Supplementary Table S8).

Only in the earliest survey period in Egypt (2000, 2003, and 2005) and Albania (2008–09) did girls with a washer at home have an advantage in school attendance (RR 1.05; 95% CI 1.02, 1.08 in Egypt and RR 1.10; 95% CI 1.02, 1.2 in Albania). In the latest survey for Albania, however, which was conducted in 2017–18, washer ownership had become very common (0.93; 95% CI 0.91, 0.94), while ownership was considerably lower in the earliest survey, 2008–09 (0.78; 95% CI 75, 0.81). In Guyana, boys with a washer at home had a 10% (RR 0.9; 95% CI 0.82, 0.995) lower school attendance than boys without a washer in the earliest survey period (2005). Other differences were not statistically significant. Notably, no country with a survey in the most recent period, 2013–21, had a statistically significant association between washer ownership and school attendance.

### 3.4 Results stratified by household wealth

**Fig. 3:**
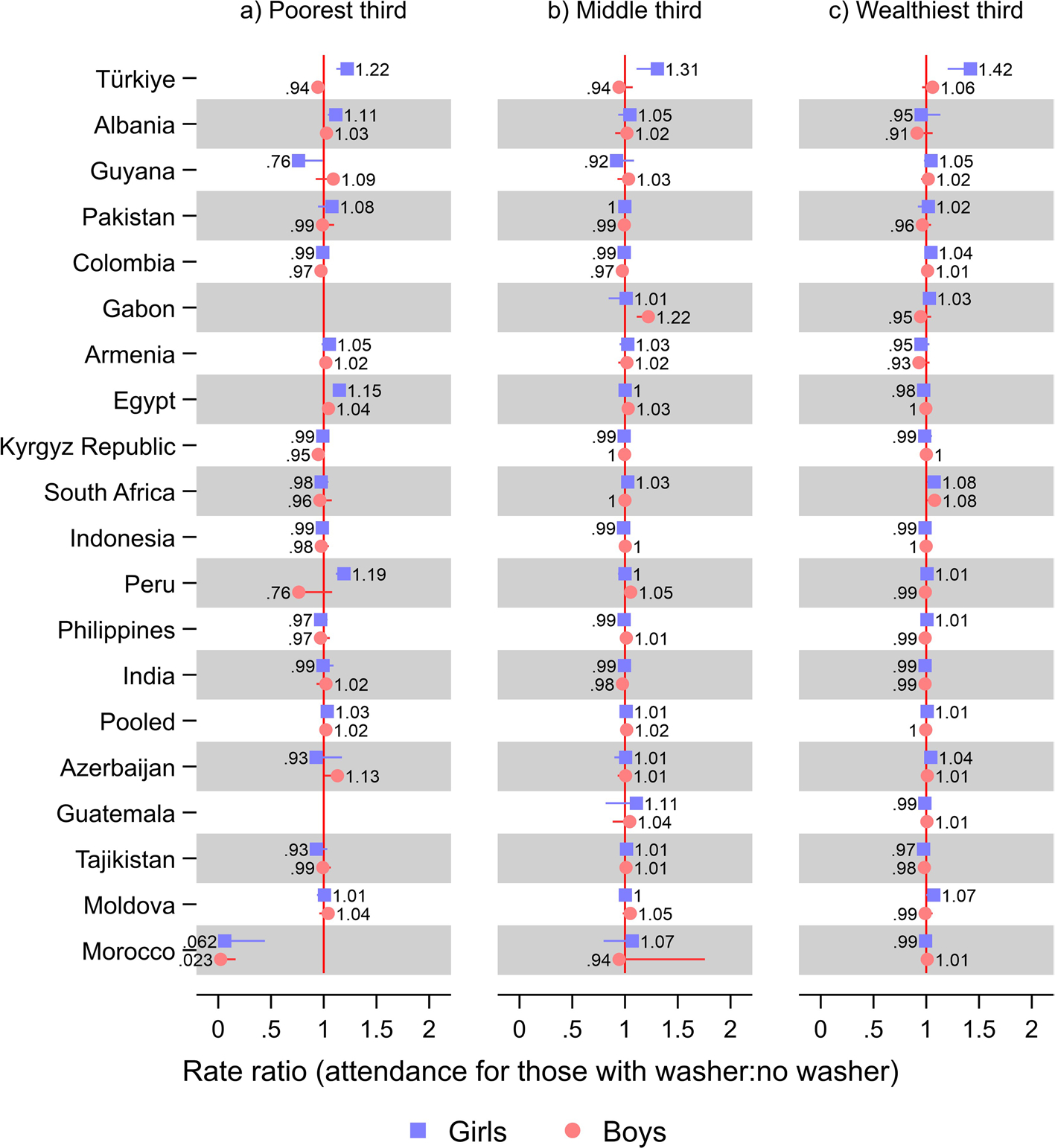
Results from Poisson regression models of school attendance on washer ownership. Notes: Countries were ordered according to the relative difference in rate ratio between boys and girls from Fig. 1. 95% confidence intervals are shown. Estimates were weighted using sampling weights rescaled to sum up to one in each survey. Pooled models were further rescaled such that each country contributed equally to the estimates. All models included a baseline term for being female and baseline terms and interactions with being female for washer ownership, fridge ownership, TV ownership, having flush toilet, a wealth index z-score, number of household members, number of household members under age five, age, highest education level of a male in household, and highest education level of a female in household, as well as adjusting for neighborhood. Upper confidence limits were omitted for estimates above one and lower confidence limits were omitted for estimates below one, for improved readability. See Supplementary Table S9 for tabulated estimates

In Türkiye, the relative advantage of girls with a washer at home in terms of school attendance was 22% (RR 1.22; 95% CI 1.12, 1.33) among the poorest third, 31% (RR 1.31; 95% CI 1.11 1.54) among the middle third, and 42% (RR 1.42; 95% CI 1.20, 1.68) among the wealthiest third (Fig. 3; Supplementary Table S9 shows tabulated estimates). In Albania, among the poorest third, girls with a washer at home had an 11% (RR 1.11; 95% CI 1.04, 1.20) greater school attendance than girls that did not: This difference was small and not statistically significant for the middle (RR 1.05; 95% CI 0.94, 1.18) and wealthiest thirds (RR 0.95; 95% CI 0.80, 1.13), for whom the proportion washer ownership was very high, 0.99 (95% CI 0.989, 0.9969) (Supplementary Table S9). In Egypt, in the poorest third, girls with a washer at home had 15% (RR 1.15; 95% CI 1.10, 1.20) greater school attendance than girls without, while the difference was small in the wealthiest third (RR 0.98; 95% CI; 96, 0.99) and absent and non-statistically significant for the middle wealth tercile (1.00; 95% CI 0.96, 1.05).

In Guyana, among the poorest third, girls with a washer at home had 34% (RR 0.76; 95% CI 0.59, 0.99) less school attendance. The proportion of adolescents with a washer at home was, however, very low, at just 0.00587 (95% CI −0.00162, 0.0134) (Supplementary Table S9). Further, in Morocco, only relatively small and non-statistically significant differences in school attendance were observed between adolescents with and without a washer at home for the middle (eg for girls, RR 1.07; 95% CI 0.80, 1.43) and wealthiest thirds (eg for girls, RR 0.99; 95% CI 0.93, 1.06). In contrast, the difference according to washer ownership was particularly large for the poorest third of boys (RR 0.023; 95% CI 0.003, 0.17) and girls (RR 0.062; 95% CI 0.009, 0.44). However, the confidence intervals were wide, and the proportion owning a washer was minimal among this group, 0.0007 (95% CI 0, 0.002) (Supplementary Table S9). In Guatemala and Gabon, among the poorest third, washing machine ownership was too low for the models to be estimated (Supplementary Table S9).

### 3.5 Results stratified by age

**Fig. 4:**
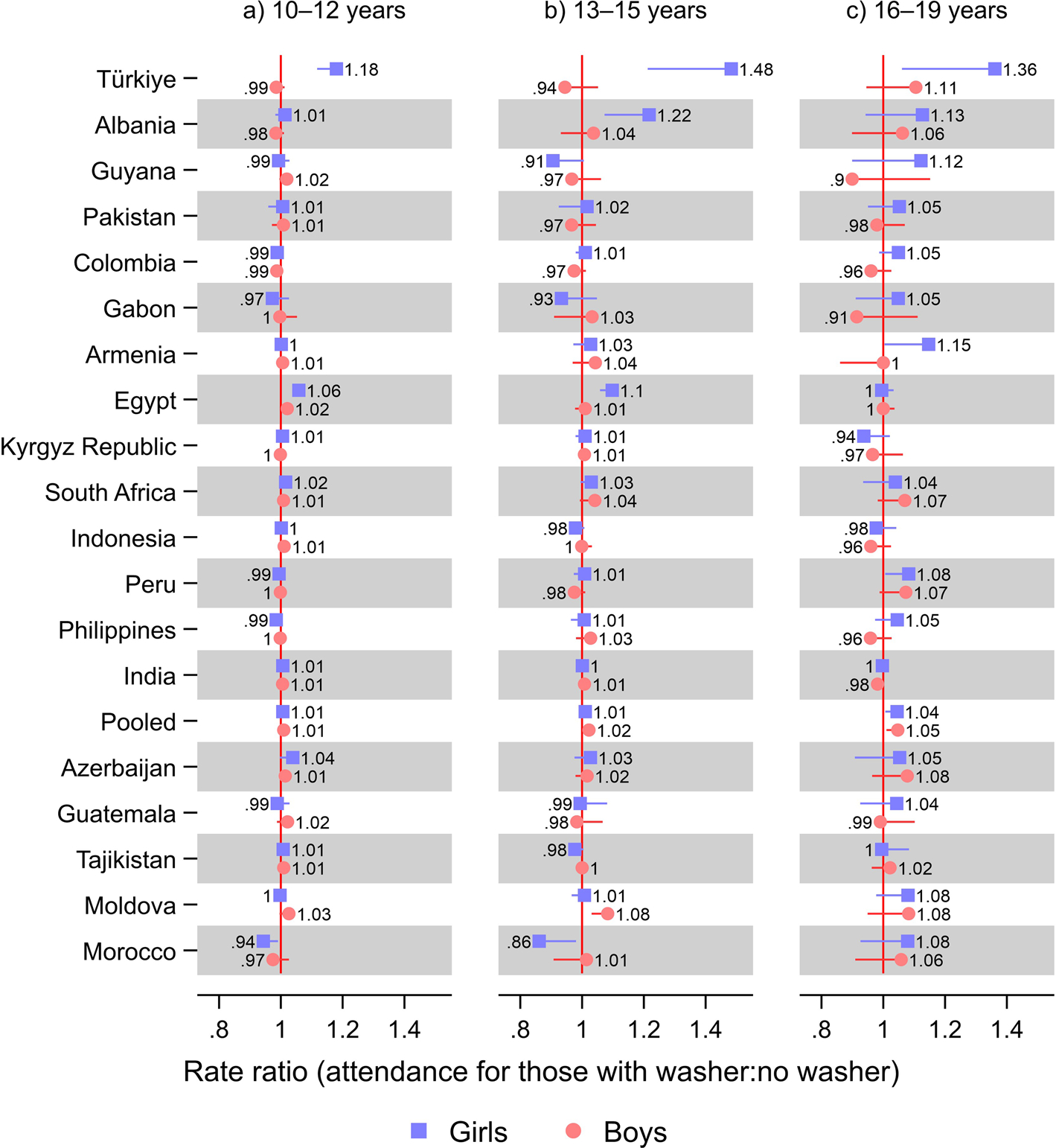
Results from Poisson regression models of school attendance on washer ownership: stratified by age. Notes: Countries were ordered according to the relative difference in rate ratio between boys and girls from Fig. 1. 95% confidence intervals are shown. Estimates were weighted using sampling weights rescaled to sum up to one in each survey. Pooled models were further rescaled such that each country contributed equally to the estimates. All models included a baseline term for being female and baseline terms and interactions with being female for washer ownership, fridge ownership, TV ownership, having flush toilet, a wealth index z-score, number of household members, number of household members under age five, age, highest education level of a male in household, and highest education level of a female in household, as well as adjusting for neighborhood. Upper confidence limits were omitted for estimates above one and lower confidence limits were omitted for estimates below one, for improved readability. See Supplementary Table S10 for tabulated estimates

In Türkiye, girls with a washer at home had greater school attendance than girls without, at all ages: 18% (RR 1.18; 95% CI 1.12, 1.25) at 10–12 years; 48% (RR 1.48; 95% CI 1.21, 1.81) at 13–15; and 36% (RR 1.36; 95% CI 1.06, 1.75) greater at 16–19 years (Fig. 4; Supplementary Table S10 shows tabulated estimates). In Albania, there was no difference according to washer ownership for 10–12-year-olds (for girls, RR 1.01; 95% CI 0.98, 1.05)—who almost had a universal school attendance (0.98; 95% CI 0.97, 0.98)—while the association was strong for 13–15-year-old girls (RR 1.22; 95% CI 1.07, 1.38). In Albania, the association with school attendance was also strong for 16–19-year-old girls (RR 1.13; 95% CI 0.94, 1.35), but the rate ratio was not statistically significant. In Egypt, having a washer at home was associated with 6% (RR 1.06; 95% CI 1.04, 1.08) greater school attendance among 10–12-year-old girls and 10% (RR 1.10; 95% CI 1.06, 1.14) greater among 13–15-year-old girls, while there was no difference for 16–19-year-olds (RR 1, 95% CI 0.96, 1.03). In Armenia, having a washer was associated with a 15% (RR 1.15; 95% CI 1.004, 1.31) greater school attendance for 16–19-year-old girls, while the difference was slight (RR 1.03; 95% CI 0.97, 1.09 at ages 13–15) or non-existent and not statistically significant at other ages (RR 1; 95% CI 0.99, 1.02 at 10–12 years).

### 3.6 Results from supplementary and sensitivity analyses

Our supplementary and sensitivity analyses generally corroborated our results (Supplementary Appendix S1). The association between school attendance and TV ownership—a placebo exposure—was mostly small but statistically significant in a few cases (Supplementary Fig. S10 and Table S18): In Pakistan, girls in households with a TV had a statistically significant 7% greater school attendance than girls that did not have a TV, while boys with a TV had 4% lower school attendance than boys that did not. In Türkiye the association with TV ownership was substantial, negative, and statistically significant, both for boys and girls. The association was further positive and statistically significant for boys in Colombia and girls in Armenia.

When studying years of education as an outcome, instead of school attendance, Türkiye also had by far the largest association, where girls with a washing machine at home had a 0.73 (95% CI: 0.54, 0.93) more years of education than those without a washer (Supplementary Fig. S18). The absolute difference, obtained from a linear regression model, between girls with and without a washer at home in Turkey was 11 percentage points (95% CI: 0.081, 0.15) (Supplementary Fig. S12 and Table S20).

When adjusting for household level factors and estimating the difference in school attendance between boys and girls within the same household, our main results were corroborated for Türkiye, where girls in households without a washer have 17% (RR: 0.83; 95% CI: 0.67, 0.70) less school attendance than boys, while in households with a washer girls have a 9% (RR: 1.09; 95% CI: 0.99, 1.20) advantage, although the latter was not statistically significant (Supplementary Fig. S21 and Table S29). In addition to Türkiye, the interaction terms for being female and having a washer was statistically significant, indicating the girls were at a less of a disadvantage in households with a washer, in Egypt (95% 1.017, 1.073) and Pakistan (95% CI: 1.004, 1.132), although the differences were rather small. In Albania, girls were at an advantage in terms of school attendance, both in households with a washer (RR: 1.1;95% CI: 995, 1.224) and without (RR 1.01; 95% CI: 0.98, 1.05): although the advantage was greater in households with a washer, the interaction terms between being female and having a washer was not statistically signiciant (1.09; 95% CI: 0.98, 1.21).

Other results for supplementary and sensitivity analyses are presented in Appendix S1.

## 4 Discussion

Using nationally representative data on 1.6 million adolescents ages 10–19 years living in 19 middle-income countries, this paper found no clear relationship between household washing machine ownership and school attendance in most countries. However, in Türkiye, young women living in a household with a washing machine at home had 28% greater school attendance (11 percentage points) and completed 0.73 more total years of schooling than girls who did not have access to a washing machine in the household. We also observed a statistically significant positive association between washing machine ownership and school attendance for girls in Egypt and Albania, although much smaller than for Türkiye. In Egypt, for example, girls with a washer at home had 4% greater school attendance compared to girls who did not.

With economic development and rising incomes, households have started acquiring household appliances and other household technology, which make domestic work more efficient, freeing up time for other activities (Brenneman 2014). Since women and girls are commonly the primary caretakers of households, these gains in efficiency can also improve their opportunities outside the home in terms of economic activity and education (Greenwood et al. 2005). However, although appliances can increase efficiency, they do not necessarily reduce the time spent on household production (Ramey and Francis 2009), and the time freed up has been suggested to be re-allocated to improvements in household hygiene and direct care of children (Mokyr 2000).

Globally, 7.9% of children 5–17 years old engage in economic activity (ILO 2022a). When also considering household chores, this share increases to 9.5%, according to the International Labour Organization. However, these indicators consider “child labor” to consist of at least one hour of economic activity among 5–11-year-olds, 14 h among 12–14-year-olds, and 43 h among 15–14-year-olds, and household chores for at least 21 h for 5–14-year-olds, while household chores among 15–17-year-olds are not considered to be “child labor” by the ILO (ILO 2022b). Therefore, frequent work at an early age, including household chores, can potentially compromise the educational development of a higher share of children than indicated by the ILO child labor statistics.

For boys, the association between washing machine ownership and school attendance was small and mostly not statistically significant in all countries. Indeed, boys generally engage more in child labor, particularly in family work (e.g., on a family farm) and work outside the home, while girls usually have been found to do more household work (i.e., chores), mirroring commonly observed gender norms (Putnick and Bornstein 2016). Without modern home appliances and utilities, household work can take over 50 h per week, with an additional 15–30 h when there is an infant to care for in the household (Ramey and Francis 2009). In low- and middle-income countries, most households do not own appliances like washing machines and many do not have access to piped water or electricity. Laundry may need to be washed by hand after fetching water or traveling to a water source in the mornings (during school hours) to be dry and ready by night time (Assaad et al. 2010).

The few cases of positive relationship between washing machine ownership and girls’ school attendance also appeared to be period dependent and the association varied across survey years within countries. For example, the positive association was not observed in the most recent surveys in Türkiye (2013), Albania (2017–18), and Egypt (2014), when school attendance and washing machine ownership rate were very high, particularly in Türkiye and Albania, and therefore little variance to identify a relationship. Moreover, school policies in recent decades have increasingly moved to raising the compulsory schooling age and further enforcing these policies (e.g., through penalties and, in extremis, short-term imprisonment of parents who do not send their children to school), which may increase the opportunity costs of keeping adolescents at home. For example, Türkiye increased compulsory schooling from 8 to 12 years in 2012.

Even though our results indicated that a relationship between washing machine ownership and school attendance was not a general phenomenon, the relationship we observed in Türkiye may be real and based on specific attributes of the Turkish context, for example, gender norms. There may be specific structural, economic, or cultural factors present which make washing machine ownership beneficial for girl’s school attendance. In this paper, we were unable to explore these factors.

### 4.1 Study limitations

Despite our effort to determine the relationship between household ownership of a washing machine and adolescents’ school attendance, our study has several limitations.

First, washing machine ownership is not random, and the estimated relationship between washer ownership and school attendance may suffer from confounding. The inherent link between washer ownership and living standards and socioeconomic status—which in turn improves school attendance—is clear. However, we controlled for several measures of living standards and socioeconomic status, although the impact of some residual confounding cannot be ruled out. Further, the pattern observed for washer ownership was generally not observed for ownership of other assets, which were less likely to affect schooling but also inherently linked to living standards, primarily TV ownership.

Parents with a strong preference for their child to attend school may also be more likely to prioritize getting a washing machine to reduce the burden of child labor, as well as engaging in other behaviors that increase the child’s school attendance and thereby bias the relationship upwards. Despite a potential upward confounding bias from living standards and parental preferences, we do not find a general effect of washer ownership on school attendance.

Second, the ownership of a washing machine signals living standards varyingly, depending on the context. In places where ownership is close to universal, non-ownership is more likely to signal relative disadvantage. Where education is nearly universal, non-attendance is expected to reflect relative disadvantages to a greater extent than where overall school attendance is less common. We restricted our main analyses to countries with between 5 and 95% washer ownership and school attendance (with the average across countries being 48% washer ownership and 82% school attendance) to ensure sufficient variance for obtaining estimates. However, the extent of saturation (i.e., complete presence or absence) in washer ownership and school attendance should be kept in mind, especially in sub-analyses (e.g., when studying the relationships across time or household wealth).

Third, we did not have data on children’s time use, which would have allowed us to examine the association between domestic and economic activities and schooling outcomes more directly. For example, although we generally do not find an association with school attendance, washer ownership may have instead increased economic activity outside the home among girls. Further, acquiring a washer may in some cases have enabled adolescents to do more household work, for example if the family switched from using laundry services to doing laundry themselves (Cowan 1983). Fourth, our primary outcome indicated any school attendance in the year of the survey but contained little information about the consistency of attendance throughout the school year. However, our results were similar when using the total number of years of education completed as our outcome. Fifth, although unlikely, our models are vulnerable to reverse causality because of potential misalignment in the timing between our exposure and outcome. For example, if children learned about the benefits of washing machines at school and induced their households to purchase them.

## 5 Conclusions

This paper found no clear link between washing machine ownership and school attendance for either girls or boys across several middle-income countries. However, in Türkiye, girls with a washing machine at home were substantially more likely to attend school and had completed more total years of schooling than girls without a washing machine at home. No such differences were observed for boys. Similarly, girls in Egypt and Albania appeared to be more likely to attend school when they had a washer at home, although the links were much smaller than in Türkiye. While washing machine ownership does not generally improve girls’ school attendance, it may do so in some contexts.

The United Nations’ Sustainable Development Goals move beyond primary education to focus on achieving universal secondary education. To achieve universal secondary education, policymakers should consider labor-saving household technologies, which reduce the opportunity cost for families to send their children to school. Further studies could test the causal effects of household ownership of washing machines and improving access to, for example, public laundromats to further improve girls’ school performance.

## Supplementary Material

Supplemental file

## Figures and Tables

**Table 1:** Descriptive statistics

	School attendance				Washer ownership	Survey years
	Both	Male	Female	Difference	Both	

Albania	.836	.84	.833	− .0074	.853	2008–09, 2017–18
(*N* = 13,749)	[.825, .847]	[.827, .853]	[.818, .848]	[− .0246, .00979]	[.835, .871]	
Armenia	.85	.844	.856	.0114	.845	2005, 2010, 2015–16
(*N* = 10,925)	[.84, .86]	[.83, .859]	[.842, .869]	[− .00699, .0298]	[.83, .859]	
Azerbaijan	.871	.915	.827	− .0879***	.207	2006
(*N* = 6,203)	[.857, .885]	[.902, .929]	[.807, .848]	[− .11, − .0658]	[.178, .235]	
Colombia	.799	.791	.808	.0165***	.431	2004–05, 2015–16
(*N* = 61,245)	[.793, .805]	[.783, .799]	[.8, .815]	[.00696, .026]	[.416, .446]	
Egypt	.763	.788	.738	− .0496***	.809	2000, 2003, 2005, 2008, 2014
(*N* = 102,079)	[.758, .769]	[.782, .793]	[.731, .745]	[− .0563, − .0428]	[.802, .815]	
Gabon	.902	.898	.905	.00617	.0722	2012
(*N* = 8,462)	[.889, .914]	[.882, .915]	[.89, .919]	[− .0134, .0257]	[.0508, .0936]	
Guatemala	.684	.722	.646	− .0766***	.113	2014–15
(*N* = 24,453)	[.671, .696]	[.708, .736]	[.63, .661]	[− .0921, − .061]	[.099, .127]	
Guyana	.771	.77	.773	.00341	.179	2005, 2009
(*N* = 6,801)	[.756, .787]	[.749, .791]	[.753, .793]	[− .0218, .0286]	[.158, .2]	
India	.781	.797	.765	− .0315***	.13	2015–16, 2019–21
(*N* = 1,075,968)	[.78, .783]	[.795, .799]	[.763, .767]	[− .0338, − .0292]	[.128, .133]	
Indonesia	.822	.813	.832	.0194***	.35	2017
(*N* = 35,687)	[.816, .829]	[.804, .822]	[.824, .841]	[.00795, .0308]	[.335, .365]	
Kyrgyz Republic	.91	.906	.913	.00738	.638	2012
(*N* = 6,551)	[.897, .923]	[.891, .92]	[.897, .93]	[− .0104, .0252]	[.606, .671]	
Moldova	.875	.85	.9	.0496***	.653	2005
(*N* = 5,457)	[.863, .887]	[.833, .867]	[.886, .913]	[.03, .0692]	[.627, .679]	
Morocco	.605	.648	.564	− .0835***	.138	2003–04
(*N* = 14,022)	[.583, .628]	[.625, .671]	[.538, .59]	[− .103, − .0637]	[.117, .16]	
Pakistan	.515	.572	.457	− .116***	.499	2006–07, 2012–13, 2017–18
(*N* = 70,840)	[.502, .528]	[.559, .585]	[.44, .473]	[− .129, − .103]	[.479, .519]	
Peru	.796	.794	.798	.00459	.174	2009, 2010, 2011, 2012
(*N* = 82,853)	[.791, .801]	[.788, .8]	[.792, .805]	[− .0027, .0119]	[.164, .184]	
Philippines	.815	.793	.84	.0467***	.349	2003, 2017
(*N* =39,196)	[.808, .823]	[.783, .803]	[.832, .848]	[.0358, .0576]	[.329, .368]	
South Africa	.912	.919	.903	− .0159*	.375	2016
(*N* = 7,000)	[.903, .92]	[.908, .931]	[.891, .916]	[− .032, .000166]	[.341, .409]	
Tajikistan	.848	.891	.804	− .0868***	.261	2012, 2017
(*N* = 16,544)	[.839, .858]	[.881, .901]	[.79, .818]	[− .102, − .0714]	[.238, .283]	
Türkiye	.738	.771	.703	− .0678***	.866	2003–04, 2008, 2013
(*N* = 26,229)	[.728, .747]	[.761, .782]	[.69, .717]	[− .0823, − .0534]	[.852, .879]	
Pooled	.824	.832	.816	− .0159***	.475	
(*N* = 1,614,264)	[.82, .828]	[.827, .837]	[.811, .821]	[− .0217, − .0101]	[.464, .486]	

Estimates were weighted using sampling weights rescaled to sum up to one for each survey. Pooled models were further rescaled such that each country contributed equally to the estimates. 95% confidence intervals shown in brackets were adjusted for clustering at the level of primary sampling units. Total number of observations are shown in parentheses below countries names. See Supplementary Table S2 for descriptive statistics for other variables

## Data Availability

All Demographic and Health Surveys are available at https://dhsprogram.com (requiring a simple application).
