# Supplementary material for: Washing machine ownership and girls’ school attendance: a cross-sectional analysis of adolescents in 19 middle-income countries
Source: J Econ Inequal. Author manuscript; Available in PMC 2025 Mar 1. (PMC11870392; doi:10.1007/s10888-023-09612-7)
Supplement: Supplemental file [file NIHMS2011458-supplement-Supplemental_file.pdf]

SUPPLEMENT

Washing machine ownership and girls' school attendance: a cross-sectional analysis of adolescents in 19 middle-income countries

SUPPLEMENT 1: Information on data and descriptive statistics ... 3

Table S1. Sample sizes and data ... 4

Table S2A. Descriptive statistics ... 5

Table S2B. Descriptive statistics ... 7

Table S2C. Descriptive statistics ... 9

Table S2D. Descriptive statistics ... 11

Table S3. Combinations of household amenities ... 13

Table S4. Variance inflation factor (VIF) for main independent variables and the relationship between washer ownership and the wealth index ... 16

Figure S1. Proportion attending school across time by sex ... 17

Figure S2. Proportion living in households with a washer, TV, and fridge across time ... 18

Table S5. Proportion attending school by sex and proportion owning a washer, TV, and fridge across time ... 19

SUPPLEMENT 2: Full model outputs for main results ... 21

Table S6A. Results from Poisson regression models of school attendance on washer ownership ... 22

Table S6B. Results from Poisson regression models of school attendance on washer ownership ... 25

Table S6C. Results from Poisson regression models of school attendance on washer ownership ... 28

Table S6D. Results from Poisson regression models of school attendance on washer ownership ... 31

SUPPLEMENT 3: Tabulated estimates from main Figures ... 34

Table S7. Results from Poisson regression models of school attendance on washer ownership ... 35

Table S8. Results from Poisson regression models of school attendance on washer ownership: stratified by survey year ... 37

Table S9. Results from Poisson regression models of school attendance on washer ownership: stratified by household wealth ... 40

Table S10. Results from Poisson regression models of school attendance on washer ownership: stratified by age ... 44

SUPPLEMENT 4: Supplementary and sensitivity analyses ... 48

Appendix S1. Supplementary and sensitivity analyses ... 49

Figure S3. Results from Poisson regression models of school attendance on fridge ownership ... 54

Figure S4. Results from Poisson regression models of school attendance on piped water ... 55

Figure S5. Results from Poisson regression models of school attendance on electricity ... 56

Figure S6. Results from Poisson regression models of school attendance on car ownership ... 57

Figure S7. Results from Poisson regression models of school attendance on scooter ownership ... 58

Figure S8. Results from Poisson regression models of school attendance on clean cooking ... 59

Figure S9. Results from Poisson regression models of school attendance on flush toilet ... 60

Figure S10. Results from Poisson regression models of school attendance on TV ownership ... 61

Figure S11. Results from logistic regression models of school attendance on washer ownership ... 62

Figure S12. Results from linear regression models of school attendance on washer ownership ... 63

Figure S13. Results from Poisson regression models of school attendance on washer ownership: excluding flush toilet, fridge, and TV from independent variables ... 64

Figure S14. Results from Poisson regression models of school attendance on washer ownership: excluding the wealth index from independent variables ... 65

Figure S15. Results from Poisson regression models of school attendance on washer ownership: only including descendants of household head ... 66

Figure S16. Results from Poisson regression models of school attendance on washer ownership: only including households with electricity ... 67

Figure S17. Results from Poisson regression models of school attendance on washer ownership: stratified by overall school attendance in region ... 68

Figure S18. Results from linear regression models of school attendance on washer ownership: number of complete years of education as an outcome ... 69

Figure S19. Results from linear regression models of school attendance on washer ownership: number of complete years of education as an outcome, by survey year ... 70

Figure S20. Results from Poisson regression models of school attendance on washer ownership: stratified by the extent of female disadvantage in school attendance at the regional level ... 71

Figure S21. Results from Poisson regression models of school attendance on being female interacted with washer ownership adjusting for all household level factors ... 72

SUPPLEMENT 5: Tabulated estimates from sensitivity analyses Figures: Main model parameters and combinations ... 73

Table S11. Results from Poisson regression models of school attendance on fridge ownership ... 73

Table S12. Results from Poisson regression models of school attendance on piped water ... 74

Table S13. Results from Poisson regression models of school attendance on electricity ... 75

Table S14. Results from Poisson regression models of school attendance on car ownership ... 76

Table S15. Results from Poisson regression models of school attendance on scooter ownership ... 77

Table S16. Results from Poisson regression models of school attendance on clean cooking ... 78

Table S17. Results from Poisson regression models of school attendance on flush toilet ... 79

Table S18. Results from Poisson regression models of school attendance on TV ownership ... 80

Table S19. Results from logistic regression models of school attendance on washer ownership ... 81

Table S20. Results from linear regression models of school attendance on washer ownership ... 83

Table S21. Results from Poisson regression models of school attendance on washer ownership: excluding flush toilet, fridge ownership, and TV ownership from independent variables ... 85

Table S22. Results from Poisson regression models of school attendance on washer ownership: excluding the wealth index from independent variables ... 87

Table S23. Results from Poisson regression models of school attendance on washer ownership: only including descendants of household head ... 89

Table S24. Results from Poisson regression models of school attendance on washer ownership: only including households with electricity ... 91

Table S25. Results from Poisson regression models of school attendance on washer ownership: stratified by overall school attendance in region ... 92

Table S26. Results from linear regression models of school attendance on washer ownership: number of complete years of education as an outcome ... 96

Table S27. Results from linear regression models of school attendance on washer ownership: number of complete years of education as an outcome, by survey years ... 98

Table S28. Results from Poisson regression models of school attendance on washer ownership: stratified by the extent of female disadvantage in school attendance at the regional level ... 101

Table S29. Results from Poisson regression models of school attendance on being female interacted with washer ownership adjusting for all household level factors ... 105

SUPPLEMENT 1: Information on data and descriptive statistics

Table S1. Sample sizes and data

	Total observations	Missing info on schooling	Missing info on washer	Missing covariates	Single valid observation in neighborhood	Valid observations
Pooled	1,622,514	1,213	538	6,222	758	1,614,264
Albania 2008–09	6,374	0	0	2	0	6,372
Albania 2017–18	7,417	0	0	23	17	7,377
Armenia 2005	4,555	14	6	18	0	4,523
Armenia 2010	3,198	17	3	2	6	3,171
Armenia 2015–16	3,239	1	0	1	6	3,231
Azerbaijan 2006	6,260	28	12	29	0	6,203
Colombia 2004–05	32,303	12	0	358	154	31,782
Colombia 2015–16	29,988	0	0	209	316	29,463
Egypt 2000	22,457	0	0	6	3	22,448
Egypt 2003	12,600	1	2	9	23	12,567
Egypt 2005	25,307	149	2	64	4	25,096
Egypt 2008	19,441	25	5	21	12	19,382
Egypt 2014	22,636	2	6	11	35	22,586
Gabon 2012	8,882	116	31	282	4	8,462
Guatemala 2014–15	24,490	27	2	9	0	24,453
Guyana 2005	2,382	29	5	111	0	2,243
Guyana 2009	5,103	98	35	452	3	4,558
India 2015–16	561,311	74	0	2,763	15	558,460
India 2019–21	518,191	63	0	563	57	517,508
Indonesia 2017	35,957	181	17	79	0	35,687
Kyrgyz Republic 2012	6,590	27	5	11	0	6,551
Moldova 2005	5,519	35	11	27	0	5,457
Morocco 2003–04	14,072	7	5	38	0	14,022
Pakistan 2006–07	26,148	68	335	457	0	25,609
Pakistan 2012–13	22,220	50	27	51	0	22,109
Pakistan 2017–18	23,139	5	4	11	0	23,122
Peru 2009	22,152	0	0	62	1	22,089
Peru 2010	20,706	0	0	17	2	20,687
Peru 2011	19,919	0	0	34	3	19,882
Peru 2012	20,233	0	0	30	8	20,195
Philippines 2003	13,892	14	4	35	3	13,840
Philippines 2017	25,386	1	0	29	0	25,356
South Africa 2016	7,249	0	0	198	51	7,000
Tajikistan 2012	8,339	74	5	10	0	8,250
Tajikistan 2017	8,298	0	0	4	0	8,294
Türkiye 2003–04	9,642	42	4	74	15	9,509
Türkiye 2008	8,711	25	2	60	8	8,618
Türkiye 2013	8,208	28	10	62	12	8,102

Notes: Total observations refers to adolescents 10–19 that were residents in the interviewed households.

Table S2A. Descriptive statistics

	Pooled	Albania	Armenia	Azerbaijan	Colombia
School attendance	.824	.836	.85	.871	.799
	[.82, .828]	[.825, .847]	[.84, .86]	[.857, .885]	[.793, .805]
Household owns washer	.475	.853	.845	.207	.431
	[.464, .486]	[.835, .871]	[.83, .859]	[.178, .235]	[.416, .446]
Female	.5	.497	.495	.502	.497
	[.497, .504]	[.486, .507]	[.484, .506]	[.487, .518]	[.492, .503]
Household wealth index z-score	-.0805	-.16	-.0205	-.1	-.139
	[-.106, -.0551]	[-.23, -.0897]	[-.0958, .0548]	[-.221, .0205]	[-.174, -.104]
Age (years)	14.39	14.5	14.47	14.43	14.43
	[14.37, 14.42]	[14.44, 14.56]	[14.39, 14.55]	[14.34, 14.51]	[14.39, 14.47]
Age of household head (years)	48.59	50.64	53.37	48.6	46.9
	[48.41, 48.78]	[50.23, 51.04]	[52.82, 53.92]	[47.92, 49.28]	[46.69, 47.11]
Number of household members	5.761	4.89	4.941	5.105	5.437
	[5.714, 5.809]	[4.83, 4.949]	[4.875, 5.007]	[4.988, 5.222]	[5.392, 5.483]
Number of household members under 5 years	.465	.161	.159	.169	.432
	[.451, .479]	[.148, .173]	[.146, .173]	[.147, .191]	[.419, .446]
Highest female education level in household:					
None	.085	.00984	.000405	.00614	.0514
	[.0811, .0889]	[.00585, .0138]	[-.0000216, .000831]	[.00285, .00943]	[.0473, .0555]
Primary	.185	.538	.0378	.0104	.344
	[.178, .192]	[.514, .561]	[.0296, .0459]	[.00647, .0144]	[.333, .355]
Secondary	.502	.332	.527	.851	.382
	[.494, .511]	[.314, .35]	[.501, .552]	[.829, .873]	[.37, .394]
Higher	.188	.105	.422	.122	.17
	[.181, .195]	[.0913, .119]	[.396, .448]	[.101, .143]	[.161, .18]
No female in household	.0396	.0156	.0133	.0107	.0518
	[.0374, .0418]	[.0103, .0209]	[.00983, .0168]	[.00664, .0147]	[.0487, .0548]
Highest male education level in household:					
None	.0444	.00834	.00109	.00151	.0543
	[.042, .0467]	[.00535, .0113]	[2.04e-06, .00217]	[.00034, .00267]	[.0502, .0585]
Primary	.161	.412	.0557	.00928	.309
	[.155, .167]	[.391, .434]	[.0463, .0652]	[.00442, .0141]	[.299, .319]
Secondary	.438	.385	.446	.69	.295
	[.43, .445]	[.369, .402]	[.422, .469]	[.663, .716]	[.286, .304]
Higher	.188	.102	.348	.178	.124
	[.182, .194]	[.089, .114]	[.326, .37]	[.155, .2]	[.116, .132]
No male in household	.169	.0924	.15	.122	.218

	Pooled	Albania	Armenia	Azerbaijan	Colombia
	[.164, .174]	[.0828, .102]	[.137, .163]	[.106, .138]	[.211, .225]
Household owns refrigerator	.71	.95	.898	.756	.745
	[.701, .719]	[.942, .957]	[.885, .91]	[.72, .791]	[.734, .756]
Household owns TV	.879	.988	.955	.959	.895
	[.873, .885]	[.985, .991]	[.947, .963]	[.949, .968]	[.888, .902]
Household has flush toilet	.524	.975	.682	.412	.897
	[.511, .538]	[.969, .981]	[.647, .716]	[.347, .476]	[.889, .905]
Household owns motorbike*	.0768	.121	.00552	.0123	.185
	[.0736, .0801]	[.106, .135]	[.00324, .0078]	[.00638, .0183]	[.177, .192]
Household owns car*	.262	.355	.375	.234	.118
	[.255, .269]	[.336, .374]	[.357, .393]	[.211, .258]	[.109, .126]
Household uses clean cooking fuel*	.748	.68	.967	.885	.822
	[.738, .758]	[.651, .709]	[.958, .975]	[.854, .916]	[.811, .832]
Household has piped drinking water*	.64	.614	.935	.529	.812
	[.627, .653]	[.583, .645]	[.917, .954]	[.468, .589]	[.802, .823]
Household has electricity*	.933	NA	.9987	.9954	.968
	[.926, .94]		[.9971, 1]	[.99, 1.001]	[.963, .972]
Observations	1,610,736	13,749	10,922	6,203	61,245

Notes: Estimates were weighted using sampling weights rescaled to sum up to one for each survey. Estimates from pooled sample were further rescaled such that each country contributed equally to the estimates. 95% confidence intervals shown in brackets were adjusted for clustering at the level of primary sampling units. *A few missing observations were excluded for these variables (these variables were not used in the main models, only supplementary analyses).

Table S2B. Descriptive statistics

	Egypt	Gabon	Guatemala	Guyana	India
School attendance	.763	.902	.684	.771	.781
	[.758, .769]	[.889, .914]	[.671, .696]	[.756, .787]	[.78, .783]
Household owns washer	.809	.0722	.113	.179	.13
	[.802, .815]	[.0508, .0936]	[.099, .127]	[.158, .2]	[.128, .133]
Female	.486	.521	.501	.507	.486
	[.482, .489]	[.501, .54]	[.493, .509]	[.494, .521]	[.485, .487]
Household wealth index z-score	-.225	.135	-.198	-.0554	-.125
	[-.25, -.2]	[.0214, .248]	[-.259, -.136]	[-.15, .0397]	[-.134, -.116]
Age (years)	14.48	14.22	14.33	14.11	14.41
	[14.46, 14.5]	[14.11, 14.32]	[14.29, 14.38]	[14.03, 14.2]	[14.41, 14.42]
Age of household head (years)	47.89	46.39	45.31	46.01	47.6
	[47.77, 48.01]	[45.71, 47.07]	[45.03, 45.59]	[45.41, 46.61]	[47.55, 47.65]
Number of household members	6.789	7.597	6.669	5.863	5.894
	[6.724, 6.855]	[7.346, 7.848]	[6.567, 6.771]	[5.674, 6.052]	[5.878, 5.91]
Number of household members under 5 years	.589	1.164	.654	.525	.309
	[.573, .606]	[1.076, 1.252]	[.622, .687]	[.473, .577]	[.305, .312]
Highest female education level in household:					
None	.445	.0501	.304	.0173	.431
	[.436, .454]	[.0391, .0612]	[.282, .325]	[.0107, .024]	[.428, .434]
Primary	.188	.249	.477	.289	.16
	[.183, .194]	[.218, .28]	[.459, .494]	[.263, .314]	[.158, .161]
Secondary	.27	.516	.145	.582	.324
	[.263, .276]	[.486, .546]	[.131, .158]	[.556, .608]	[.321, .326]
Higher	.0712	.0932	.0359	.0616	.063
	[.0669, .0755]	[.0685, .118]	[.0301, .0418]	[.0489, .0744]	[.0616, .0644]
No female in household	.0261	.0922	.0394	.0505	.0229
	[.0246, .0277]	[.0793, .105]	[.0352, .0437]	[.0416, .0595]	[.0223, .0234]
Highest male education level in household:					
None	.238	.0292	.161	.014	.202
	[.231, .244]	[.0203, .0381]	[.148, .175]	[.00804, .02]	[.2, .204]
Primary	.2	.125	.432	.262	.156
	[.195, .205]	[.106, .144]	[.417, .448]	[.237, .287]	[.154, .157]
Secondary	.35	.434	.146	.443	.435
	[.344, .356]	[.4, .468]	[.134, .157]	[.419, .466]	[.432, .437]
Higher	.131	.153	.0405	.0579	.109
	[.126, .136]	[.12, .186]	[.0339, .0471]	[.0468, .0689]	[.107, .111]
No male in household	.0812	.258	.22	.223	.0986

	Egypt	Gabon	Guatemala	Guyana	India
	[.0781, .0842]	[.233, .283]	[.209, .23]	[.202, .244]	[.0973, .0999]
Household owns refrigerator	.807	.388	.388	.591	.293
	[.799, .816]	[.344, .431]	[.365, .411]	[.554, .629]	[.289, .296]
Household owns TV	.946	.893	.708	.808	.625
	[.943, .95]	[.87, .915]	[.684, .733]	[.776, .84]	[.622, .629]
Household has flush toilet	.97	.336	.532	.466	.56
	[.967, .973]	[.288, .384]	[.503, .562]	[.43, .502]	[.556, .564]
Household owns motorbike*	.0377	.0133	.186	.0816	.419
	[.0352, .0403]	[.00415, .0225]	[.171, .201]	[.0678, .0953]	[.416, .422]
Household owns car*	.074	.204	.171	.157	.0522
	[.0699, .0781]	[.166, .242]	[.157, .185]	[.138, .176]	[.051, .0534]
Household uses clean cooking fuel*	.986	.851	.285	.731	.439
	[.984, .988]	[.82, .882]	[.256, .313]	[.696, .767]	[.434, .443]
Household has piped drinking water*	.899	.867	.606	.425	.414
	[.891, .906]	[.829, .904]	[.581, .632]	[.384, .465]	[.409, .419]
Household has electricity*	.99	.923	.869	.749	.91
	[.989, .992]	[.901, .944]	[.85, .887]	[.71, .788]	[.908, .912]
Observations	100,459	8,457	24,452	6,798	1,075,968

Notes: Estimates were weighted using sampling weights rescaled to sum up to one for each survey. Estimates from pooled sample were further rescaled such that each country contributed equally to the estimates. 95% confidence intervals shown in brackets were adjusted for clustering at the level of primary sampling units. *A few missing observations were excluded for these variables (these variables were not used in the main models, only supplementary analyses).

Table S2C. Descriptive statistics

	Indonesia	Kyrgyz Republic	Moldova	Morocco	Pakistan
School attendance	.822	.91	.875	.605	.515
	[.816, .829]	[.897, .923]	[.863, .887]	[.583, .628]	[.502, .528]
Household owns washer	.35	.638	.653	.138	.499
	[.335, .365]	[.606, .671]	[.627, .679]	[.117, .16]	[.479, .519]
Female	.486	.527	.496	.511	.495
	[.48, .493]	[.514, .541]	[.481, .51]	[.502, .52]	[.49, .5]
Household wealth index z-score	.0534	-.231	-.00755	-.141	-.0679
	[.0119, .095]	[-.333, -.13]	[-.0996, .0845]	[-.234, -.0489]	[-.115, -.021]
Age (years)	14.27	14.35	14.64	14.42	14.32
	[14.23, 14.3]	[14.24, 14.45]	[14.54, 14.73]	[14.36, 14.47]	[14.29, 14.35]
Age of household head (years)	47.72	49.89	45.14	49.31	48.51
	[47.51, 47.94]	[49.07, 50.7]	[44.67, 45.61]	[48.95, 49.68]	[48.29, 48.73]
Number of household members	5.076	5.486	4.219	7.317	9.396
	[5.029, 5.122]	[5.368, 5.604]	[4.151, 4.288]	[7.152, 7.481]	[9.233, 9.558]
Number of household members under 5 years	.403	.555	.148	.642	1.135
	[.391, .415]	[.518, .592]	[.132, .165]	[.601, .682]	[1.092, 1.178]
Highest female education level in household:					
None	.0415	.00575	.00438	.705	.633
	[.0365, .0465]	[.00234, .00917]	[.00158, .00718]	[.681, .729]	[.617, .649]
Primary	.395	.00792	.0111	.139	.132
	[.382, .409]	[.00299, .0128]	[.00682, .0153]	[.127, .152]	[.125, .139]
Secondary	.42	.567	.563	.108	.136
	[.408, .433]	[.532, .603]	[.539, .588]	[.0938, .121]	[.127, .145]
Higher	.103	.384	.339	.0299	.0718
	[.0947, .111]	[.352, .416]	[.315, .363]	[.023, .0367]	[.0654, .0782]
No female in household	.0401	.035	.0822	.0185	.0272
	[.0355, .0448]	[.0267, .0432]	[.0714, .093]	[.0152, .0219]	[.0247, .0296]
Highest male education level in household:					
None	.0205	.00317	.00414	.43	.305
	[.0174, .0236]	[.00111, .00522]	[.00136, .00691]	[.407, .454]	[.293, .317]
Primary	.341	.0038	.00673	.245	.156
	[.328, .355]	[.00177, .00584]	[.00375, .00971]	[.231, .259]	[.148, .163]
Secondary	.447	.507	.572	.171	.287
	[.436, .459]	[.475, .538]	[.549, .595]	[.155, .187]	[.278, .297]
Higher	.109	.336	.215	.0563	.166
	[.1, .117]	[.309, .364]	[.196, .235]	[.0456, .067]	[.157, .175]
No male in household	.0818	.15	.202	.097	.0864

	Indonesia	Kyrgyz Republic	Moldova	Morocco	Pakistan
	[.0764, .0873]	[.131, .169]	[.186, .217]	[.088, .106]	[.0811, .0917]
Household owns refrigerator	.612	.78	.79	.517	.438
	[.597, .627]	[.754, .805]	[.767, .813]	[.479, .554]	[.42, .455]
Household owns TV	.908	.987	.782	.647	.591
	[.899, .917]	[.981, .993]	[.759, .805]	[.612, .681]	[.573, .61]
Household has flush toilet	.863	.105	.27	.789	.693
	[.852, .874]	[.071, .139]	[.23, .31]	[.759, .819]	[.672, .713]
Household owns motorbike*	.832	.0134	.0992	.12	.362
	[.822, .842]	[.00833, .0184]	[.0854, .113]	[.107, .134]	[.347, .377]
Household owns car*	.137	.491	.284	.143	.081
	[.128, .146]	[.467, .515]	[.264, .304]	[.126, .161]	[.0735, .0885]
Household uses clean cooking fuel*	.731	.682	.859	.106	.358
	[.715, .747]	[.637, .726]	[.834, .885]	[.0822, .13]	[.333, .382]
Household has piped drinking water*	.098	.769	.338	.655	.355
	[.0877, .108]	[.719, .818]	[.297, .379]	[.61, .699]	[.335, .374]
Household has electricity*	.971	.9983	.993	.749	.92
	[.966, .977]	[.9964, 1]	[.989, .9959]	[.713, .784]	[.905, .935]
Observations	35,685	6,549	5,456	14,019	70,830

Notes: Estimates were weighted using sampling weights rescaled to sum up to one for each survey. Estimates from pooled sample were further rescaled such that each country contributed equally to the estimates. 95% confidence intervals shown in brackets were adjusted for clustering at the level of primary sampling units. *A few missing observations were excluded for these variables (these variables were not used in the main models, only supplementary analyses).

Table S2D. Descriptive statistics

	Peru	Philippines	South Africa	Tajikistan	Türkiye
School attendance	.796	.815	.912	.848	.738
	[.791, .801]	[.808, .823]	[.903, .92]	[.839, .858]	[.728, .747]
Household owns washer	.174	.349	.375	.261	.866
	[.164, .184]	[.329, .368]	[.341, .409]	[.238, .283]	[.852, .879]
Female	.483	.479	.492	.489	.494
	[.478, .487]	[.472, .486]	[.477, .506]	[.479, .498]	[.487, .502]
Household wealth index z-score	-.0761	-.0375	-.0887	-.16	-.0946
	[-.108, -.0442]	[-.0832, .00811]	[-.186, .00801]	[-.223, -.0964]	[-.137, -.0523]
Age (years)	14.35	14.25	14.42	14.47	14.51
	[14.33, 14.38]	[14.2, 14.3]	[14.33, 14.5]	[14.42, 14.53]	[14.46, 14.55]
Age of household head (years)	46.07	46.65	50.63	50.23	45.58
	[45.89, 46.25]	[46.35, 46.95]	[49.79, 51.46]	[49.72, 50.74]	[45.33, 45.83]
Number of household members	5.424	6.238	5.872	7.213	5.952
	[5.389, 5.458]	[6.168, 6.308]	[5.702, 6.042]	[7.069, 7.357]	[5.836, 6.069]
Number of household members under 5 years	.499	.568	.732	.745	.428
	[.487, .51]	[.546, .589]	[.684, .781]	[.706, .783]	[.399, .456]
Highest female education level in household:					
None	.0807	.0248	.0771	.00926	.261
	[.076, .0854]	[.0208, .0289]	[.0645, .0897]	[.00538, .0131]	[.244, .278]
Primary	.361	.308	.165	.019	.523
	[.352, .371]	[.294, .323]	[.147, .184]	[.0147, .0234]	[.508, .538]
Secondary	.335	.346	.567	.796	.147
	[.326, .344]	[.334, .357]	[.544, .591]	[.781, .812]	[.137, .156]
Higher	.159	.266	.116	.164	.0425
	[.151, .166]	[.251, .282]	[.0985, .133]	[.148, .179]	[.037, .0481]
No female in household	.0647	.0545	.0745	.0113	.0265
	[.0617, .0678]	[.0467, .0623]	[.0634, .0857]	[.00808, .0145]	[.0231, .0298]
Highest male education level in household:					
None	.0187	.019	.0508	.0038	.0651
	[.0169, .0205]	[.0157, .0223]	[.042, .0596]	[.00184, .00575]	[.0571, .073]
Primary	.27	.318	.108	.00894	.481
	[.261, .279]	[.304, .333]	[.0955, .121]	[.00638, .0115]	[.467, .496]
Secondary	.377	.313	.356	.506	.301
	[.368, .385]	[.302, .323]	[.335, .378]	[.487, .525]	[.289, .313]
Higher	.166	.246	.0666	.404	.0869
	[.159, .174]	[.233, .258]	[.0536, .0797]	[.386, .423]	[.0792, .0946]
No male in household	.168	.104	.418	.0768	.0655

	Peru	Philippines	South Africa	Tajikistan	Türkiye
	[.163, .173]	[.0953, .113]	[.397, .439]	[.068, .0857]	[.0601, .0709]
Household owns refrigerator	.397	.39	.793	.54	.965
	[.385, .41]	[.373, .408]	[.764, .821]	[.51, .57]	[.959, .97]
Household owns TV	.804	.713	.809	.975	.971
	[.794, .813]	[.696, .729]	[.782, .837]	[.969, .982]	[.966, .975]
Household has flush toilet	.54	.834	.502	.146	.764
	[.524, .556]	[.82, .849]	[.453, .55]	[.126, .167]	[.743, .784]
Household owns motorbike*	.112	.26	.0205	.0188	.0643
	[.107, .118]	[.247, .272]	[.0128, .0282]	[.0136, .024]	[.057, .0715]
Household owns car*	.0934	.0902	.261	.364	.227
	[.0881, .0986]	[.0819, .0985]	[.237, .285]	[.347, .381]	[.214, .24]
Household uses clean cooking fuel*	.587	.449	.739	.725	
	[.572, .602]	[.412, .485]	[.704, .775]	[.697, .754]	
Household has piped drinking water*	.79	.411	.828	.624	.58
	[.778, .801]	[.39, .431]	[.795, .86]	[.586, .662]	[.554, .606]
Household has electricity*	.859	.84	.902	.993	
	[.849, .869]	[.826, .854]	[.873, .931]	[.988, .9978]	
Observations	82,853	38,662	7,000	16,542	25,881

Notes: Estimates were weighted using sampling weights rescaled to sum up to one for each survey. Estimates from pooled sample were further rescaled such that each country contributed equally to the estimates. 95% confidence intervals shown in brackets were adjusted for clustering at the level of primary sampling units. *A few missing observations were excluded for these variables (these variables were not used in the main models, only supplementary analyses).

Table S3. Combinations of household amenities

	No washer/no flush toilet	No washer/flush toilet	Washer/no flush toilet	Washer/flush toilet
Pooled	.324	.201	.152	.323
	[.313, .335]	[.194, .209]	[.143, .16]	[.313, .334]
Albania	.0115	.136	.0139	.839
	[.00797, .015]	[.119, .153]	[.0097, .018]	[.82, .858]
Armenia	.0709	.0844	.247	.597
	[.0592, .0825]	[.0732, .0956]	[.218, .276]	[.565, .629]
Azerbaijan	.519	.274	.069	.138
	[.46, .578]	[.226, .322]	[.0511, .0869]	[.108, .167]
Colombia	.0923	.477	.0109	.42
	[.0849, .0998]	[.463, .491]	[.00888, .0128]	[.405, .435]
Egypt	.0178	.173	.0124	.796
	[.0157, .0199]	[.167, .18]	[.0108, .0141]	[.789, .803]
Gabon	.659	.269	.00544	.0667
	[.611, .706]	[.229, .31]	[-.000157, .011]	[.0459, .0876]
Guatemala	.463	.424	.00484	.108
	[.433, .492]	[.4, .448]	[.00304, .00664]	[.0943, .122]
Guyana	.501	.32	.0337	.145
	[.465, .536]	[.293, .347]	[.0237, .0438]	[.126, .165]
India	.431	.438	.00829	.122
	[.427, .435]	[.435, .442]	[.00785, .00873]	[.12, .125]
Indonesia	.128	.522	.00891	.341
	[.117, .139]	[.508, .536]	[.00697, .0109]	[.326, .356]
Kyrgyz Republic	.345	.0172	.55	.0877
	[.31, .379]	[.00851, .026]	[.517, .584]	[.059, .116]
Moldova	.304	.0428	.426	.227
	[.275, .333]	[.0324, .0532]	[.395, .457]	[.193, .261]
Morocco	.21	.651	.000423	.138
	[.181, .24]	[.621, .681]	[-.000299, .00114]	[.116, .16]
Pakistan	.262	.239	.0456	.453
	[.242, .282]	[.227, .252]	[.0403, .051]	[.433, .474]
Peru	.45	.375	.00989	.164
	[.434, .466]	[.363, .388]	[.00808, .0117]	[.155, .174]
Philippines	.161	.49	.00503	.344
	[.146, .175]	[.474, .507]	[.00356, .00651]	[.324, .363]
South Africa	.421	.203	.0772	.298
	[.377, .465]	[.174, .233]	[.0632, .0913]	[.262, .334]
Tajikistan	.677	.0622	.176	.0842
	[.65, .705]	[.0517, .0727]	[.158, .195]	[.0709, .0976]
Türkiye	.0808	.0537	.156	.71
	[.0691, .0925]	[.0467, .0606]	[.141, .171]	[.689, .731]

	No washer/no fridge	No washer/fridge	Washer/no fridge	Washer/fridge
Pooled	.258	.267	.0324	.442
	[.249, .267]	[.259, .276]	[.0303, .0346]	[.432, .453]
Albania	.0334	.114	.017	.836
	[.0273, .0396]	[.0986, .129]	[.0134, .0206]	[.817, .854]
Armenia	.0576	.0976	.0446	.8
	[.0487, .0666]	[.0876, .108]	[.0379, .0513]	[.783, .817]
Azerbaijan	.233	.561	.0117	.195
	[.198, .267]	[.528, .593]	[.00613, .0172]	[.166, .223]
Colombia	.236	.333	.019	.412
	[.226, .246]	[.323, .344]	[.0168, .0213]	[.396, .427]
Egypt	.0725	.119	.12	.688
	[.0678, .0772]	[.113, .124]	[.114, .126]	[.68, .697]
Gabon	.607	.321	.0055	.0667
	[.563, .65]	[.284, .358]	[.00195, .00906]	[.046, .0874]
Guatemala	.604	.283	.00836	.105
	[.58, .627]	[.266, .3]	[.00634, .0104]	[.0912, .118]
Guyana	.398	.423	.0103	.169
	[.362, .434]	[.392, .453]	[.00518, .0154]	[.148, .19]
India	.695	.175	.0124	.118
	[.691, .698]	[.172, .177]	[.0119, .0129]	[.116, .12]

Indonesia	.365	.285	.0227	.327
	[.35, .381]	[.275, .295]	[.0198, .0256]	[.312, .342]
Kyrgyz Republic	.139	.222	.0809	.557
	[.12, .159]	[.195, .25]	[.0674, .0944]	[.523, .591]
Moldova	.153	.194	.0571	.596
	[.134, .172]	[.176, .211]	[.0459, .0683]	[.568, .624]
Morocco	.482	.379	.00108	.137
	[.445, .52]	[.349, .41]	[.000299, .00186]	[.116, .159]
Pakistan	.425	.0764	.138	.361
	[.405, .444]	[.0707, .082]	[.129, .146]	[.344, .378]
Peru	.591	.235	.0116	.163
	[.578, .604]	[.227, .243]	[.0101, .0132]	[.153, .172]
Philippines	.534	.118	.0762	.272
	[.515, .552]	[.11, .125]	[.0691, .0833]	[.255, .29]
South Africa	.198	.427	.00994	.365
	[.169, .226]	[.397, .457]	[.00641, .0135]	[.332, .399]
Tajikistan	.437	.303	.0234	.237
	[.407, .467]	[.285, .321]	[.0188, .0281]	[.215, .259]
Türkiye	.0264	.108	.00883	.857
	[.0216, .0312]	[.0967, .119]	[.00675, .0109]	[.843, .87]

	No washer/no TV	No washer/TV	Washer/no TV	Washer/TV
Pooled	.106	.419	.0147	.46
	[.1, .112]	[.408, .429]	[.0132, .0162]	[.449, .471]
Albania	.00661	.141	.00503	.848
	[.00418, .00904]	[.123, .158]	[.00311, .00695]	[.83, .866]
Armenia	.0268	.128	.0185	.826
	[.0208, .0329]	[.116, .14]	[.0144, .0226]	[.81, .842]
Azerbaijan	.0415	.752		.207
	[.0318, .0511]	[.724, .78]		[.178, .235]
Colombia	.1	.469	.00466	.426
	[.0938, .107]	[.456, .482]	[.00369, .00563]	[.411, .441]
Egypt	.0283	.163	.0254	.783
	[.0258, .0307]	[.157, .169]	[.0233, .0276]	[.777, .79]
Gabon	.107	.821	.000237	.0719
	[.0852, .129]	[.793, .848]	[-.00023, .000704]	[.0506, .0933]
Guatemala	.29	.597	.00152	.111
	[.266, .314]	[.575, .619]	[.000645, .0024]	[.0976, .125]
Guyana	.189	.632	.0029	.176
	[.157, .221]	[.601, .662]	[.000311, .0055]	[.156, .197]
India	.368	.502	.00685	.124
	[.364, .371]	[.498, .505]	[.00643, .00727]	[.121, .126]
Indonesia	.0875	.563	.00423	.346
	[.0788, .0962]	[.549, .577]	[.00308, .00538]	[.331, .361]
Kyrgyz Republic	.0103	.351	.00261	.636
	[.00535, .0153]	[.319, .383]	[.000694, .00453]	[.603, .668]
Moldova	.15	.197	.068	.585
	[.13, .169]	[.18, .214]	[.0573, .0786]	[.558, .612]
Morocco	.353	.509	.000551	.138
	[.318, .387]	[.478, .539]	[-.0000369, .00114]	[.116, .16]
Pakistan	.315	.186	.0942	.405
	[.296, .334]	[.177, .196]	[.0872, .101]	[.386, .424]
Peru	.195	.63	.00076	.173
	[.186, .205]	[.62, .641]	[.000412, .00111]	[.164, .183]
Philippines	.279	.372	.00862	.34
	[.263, .295]	[.359, .386]	[.0069, .0103]	[.321, .36]
South Africa	.175	.449	.0154	.36
	[.148, .203]	[.419, .479]	[.0108, .0199]	[.326, .394]
Tajikistan	.0234	.716	.0012	.259
	[.0172, .0296]	[.693, .739]	[.000262, .00214]	[.237, .282]
Türkiye	.0114	.123	.018	.848
	[.00865, .0142]	[.11, .136]	[.0148, .0211]	[.834, .861]

Notes: 95% confidence intervals adjusted for clustering at the level of primary sampling units are shown in brackets below. Estimates were weighted using sampling weights, rescaled to sum up to one for the analytical sample each survey. Estimates from pooled sample were further rescaled such that each country contributed equally to the estimates.

Table S4. Variance inflation factor (VIF) for main independent variables and the relationship between washer ownership and the wealth index

	Washer ownership		Female×Washer ownership		Washer on wealth
	R squared	VIF	R Squared	VIF	R Squared
Albania	0.73	3.65	0.69	3.21	0.27
Armenia	0.68	3.11	0.65	2.84	0.10
Azerbaijan	0.73	3.69	0.70	3.34	0.31
Colombia	0.79	4.76	0.73	3.64	0.32
Egypt	0.67	3.07	0.63	2.71	0.15
Gabon	0.77	4.36	0.75	4.07	0.39
Guatemala	0.77	4.29	0.75	4.01	0.50
Guyana	0.73	3.73	0.72	3.60	0.36
India	0.78	4.45	0.74	3.81	0.47
Indonesia	0.76	4.10	0.73	3.68	0.41
Kyrgyz Republic	0.66	2.93	0.63	2.69	0.02
Moldova	0.70	3.39	0.68	3.14	0.24
Morocco	0.81	5.30	0.79	4.78	0.49
Pakistan	0.80	4.96	0.77	4.43	0.53
Peru	0.80	4.93	0.78	4.52	0.52
Philippines	0.79	4.75	0.76	4.22	0.50
South Africa	0.79	4.71	0.76	4.18	0.45
Tajikistan	0.72	3.54	0.68	3.08	0.28
Türkiye	0.73	3.74	0.66	2.97	0.14

Notes: The R squared and the variance inflation factor were estimated using a linear regression of the two main independent variables (indicated at the top of each column) on all other independent variables (including neighborhood means and interaction terms) in the main model (in Figure 1 and Supplementary Tables S6 and S7). The variance inflation factor was calculated as 1/(1-R squared). The column labelled 'Washer on wealth shows the R squared from a regression of washer ownership on the wealth index z-scores (including linear and squared terms). Estimates were weighted using sampling weights, rescaled to sum up to one for the analytical sample each survey.

Figure S1. Proportion attending school across time by sex

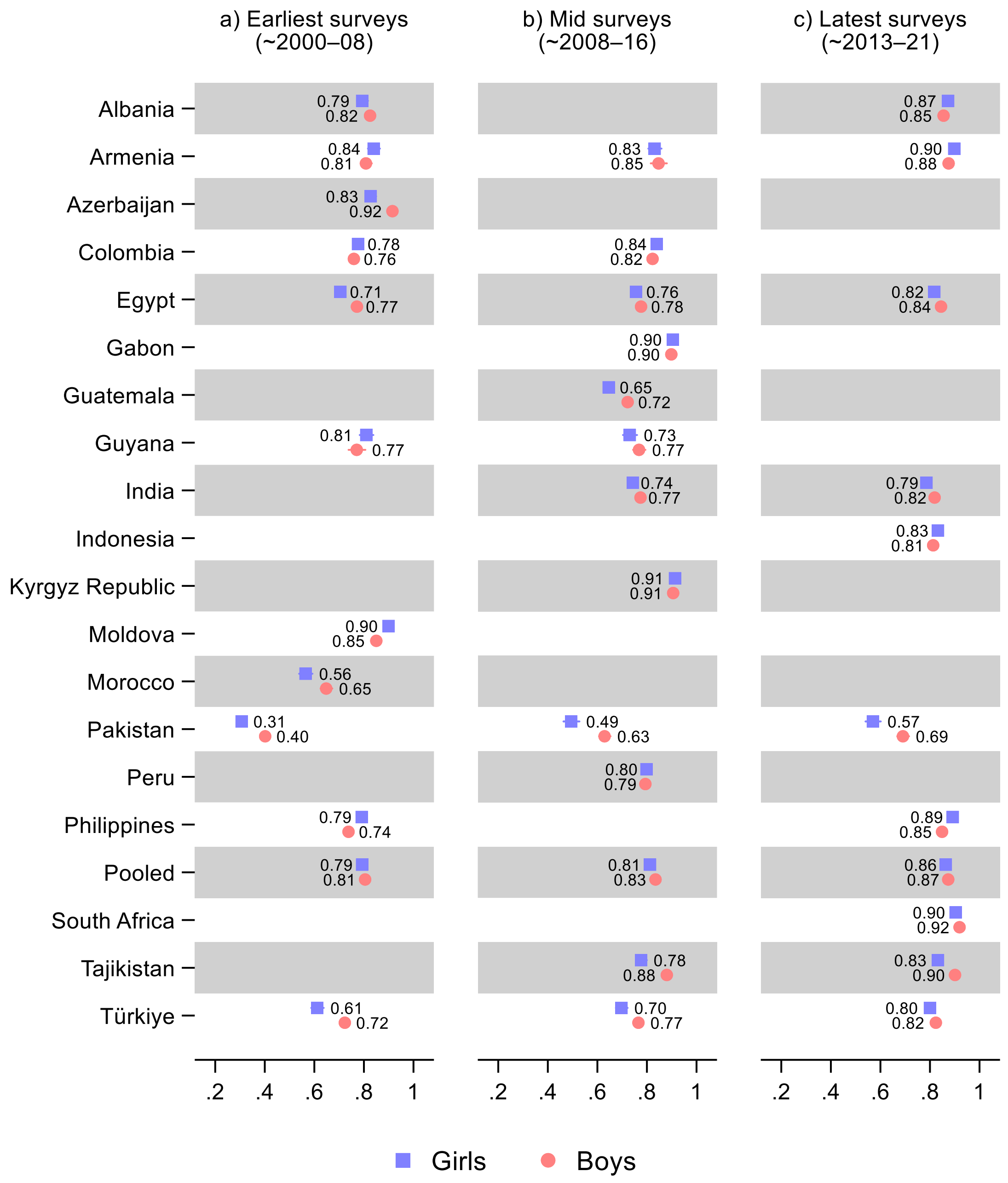

Notes: Estimates were weighted using sampling weights rescaled to sum up to one in each survey. Estimates from pooled sample were further rescaled such that each country contributed equally to the estimates. 95% confidence intervals are shown (in some cases symbols covers confidence intervals). See Supplementary Table S5 for tabulated estimates. Surveys were divided into the periods indicated in the top of each panel when possible: in some countries other periodization were used (see Supplementary Table S5 for exact periodization for each country).

Figure S2. Proportion living in households with a washer, TV, and fridge across time

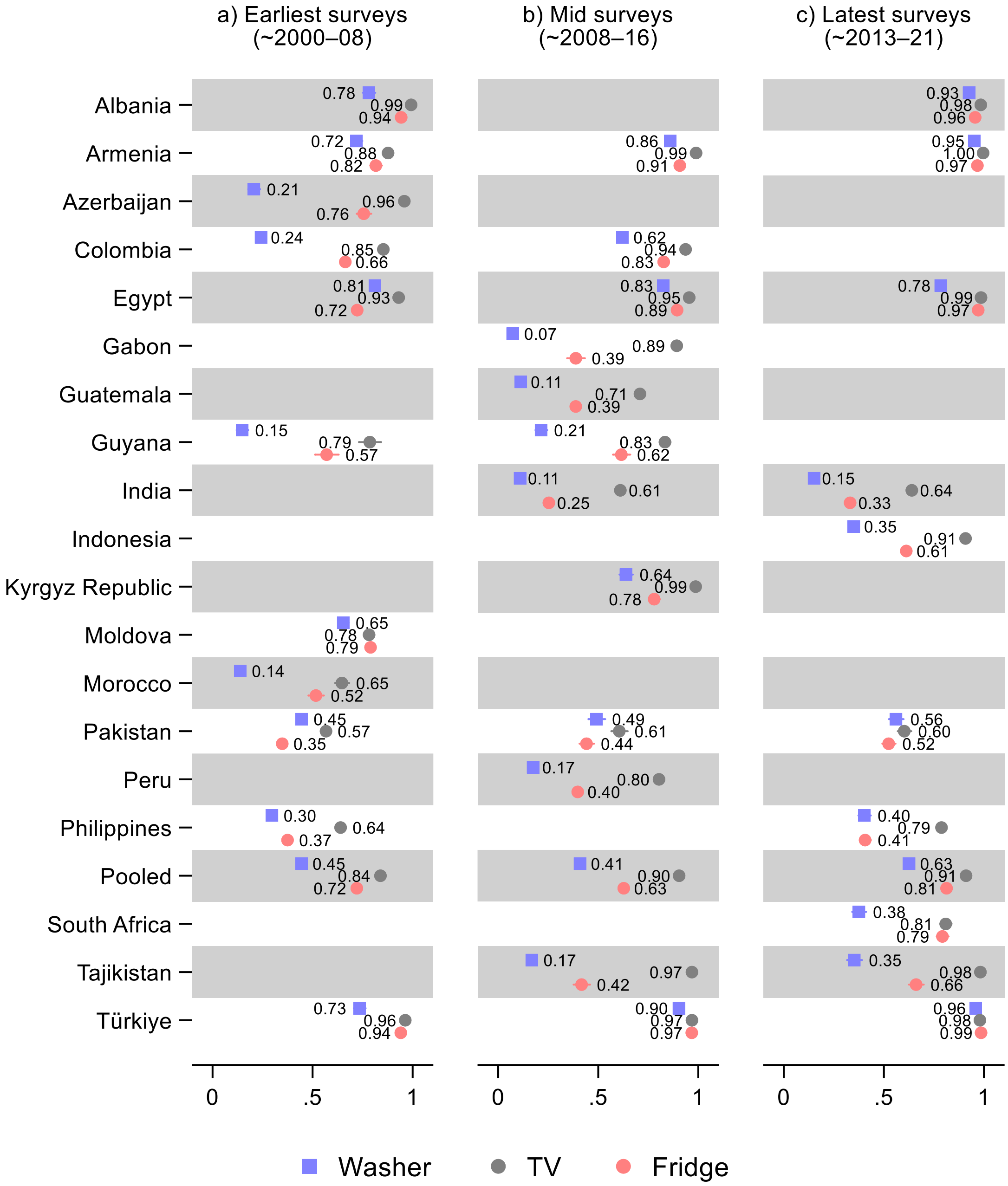

Notes: Estimates were weighted using sampling weights rescaled to sum up to one in each survey. Estimates from pooled sample were further rescaled such that each country contributed equally to the estimates. 95% confidence intervals are shown (in some cases symbols covers confidence intervals). See Supplementary Table S5 for tabulated estimates. Surveys were divided into the periods indicated in the top of each panel when possible: in some countries other periodization were used (see Supplementary Table S5 for exact periodization for each country).

Table S5. Proportion attending school by sex and proportion owning a washer, TV, and fridge across time

	School attendance		Ownership of assets		
	Males	Females	Washer	Fridge	TV
**Earliest surveys (~2000–08)**					
Albania	0.825	0.793	0.781	0.943	0.992
(2008–09)	[0.806, 0.844]	[0.772, 0.815]	[0.751, 0.811]	[0.931, 0.954]	[0.989, 0.996]
Armenia	0.808	0.839	0.720	0.816	0.877
(2005)	[0.786, 0.830]	[0.816, 0.863]	[0.693, 0.747]	[0.788, 0.845]	[0.858, 0.897]
Azerbaijan	0.915	0.827	0.207	0.756	0.959
(2006)	[0.902, 0.929]	[0.807, 0.848]	[0.178, 0.235]	[0.720, 0.791]	[0.949, 0.968]
Colombia	0.760	0.776	0.242	0.664	0.854
(2004–05)	[0.748, 0.771]	[0.766, 0.786]	[0.230, 0.255]	[0.649, 0.678]	[0.843, 0.865]
Egypt	0.772	0.705	0.812	0.723	0.930
(2000,2003,2005)	[0.765, 0.779]	[0.695, 0.715]	[0.803, 0.821]	[0.711, 0.735]	[0.925, 0.935]
Guyana	0.771	0.810	0.148	0.570	0.786
(2005)	[0.738, 0.804]	[0.783, 0.836]	[0.120, 0.176]	[0.512, 0.628]	[0.731, 0.841]
Moldova	0.850	0.900	0.653	0.790	0.782
(2005)	[0.833, 0.867]	[0.886, 0.913]	[0.627, 0.679]	[0.767, 0.813]	[0.759, 0.805]
Morocco	0.648	0.564	0.138	0.517	0.647
(2003–04)	[0.625, 0.671]	[0.538, 0.590]	[0.117, 0.160]	[0.479, 0.554]	[0.612, 0.681]
Pakistan	0.402	0.307	0.445	0.348	0.567
(2006–07)	[0.385, 0.418]	[0.288, 0.325]	[0.421, 0.469]	[0.328, 0.368]	[0.545, 0.589]
Philippines	0.738	0.791	0.297	0.375	0.640
(2003)	[0.725, 0.751]	[0.780, 0.802]	[0.275, 0.319]	[0.353, 0.396]	[0.616, 0.664]
Türkiye	0.723	0.612	0.734	0.941	0.963
(2003–04)	[0.706, 0.740]	[0.587, 0.636]	[0.706, 0.763]	[0.929, 0.952]	[0.955, 0.971]
Pooled	0.805	0.793	0.446	0.721	0.839
	[0.796, 0.814]	[0.784, 0.801]	[0.428, 0.464]	[0.705, 0.736]	[0.827, 0.852]
**Mid surveys (~2008–16)**					
Armenia	0.847	0.831	0.860	0.908	0.989
(2010)	[0.815, 0.879]	[0.805, 0.857]	[0.838, 0.882]	[0.890, 0.926]	[0.982, 0.996]
Colombia	0.823	0.839	0.621	0.827	0.937
(2015–16)	[0.812, 0.833]	[0.829, 0.849]	[0.602, 0.640]	[0.815, 0.839]	[0.930, 0.943]
Egypt	0.776	0.756	0.825	0.894	0.955
(2008)	[0.764, 0.788]	[0.743, 0.769]	[0.811, 0.839]	[0.882, 0.906]	[0.949, 0.961]
Gabon	0.898	0.905	0.072	0.388	0.893
(2012)	[0.882, 0.915]	[0.890, 0.919]	[0.051, 0.094]	[0.344, 0.431]	[0.870, 0.915]
Guatemala	0.722	0.646	0.113	0.388	0.708
(2014–15)	[0.708, 0.736]	[0.630, 0.661]	[0.099, 0.127]	[0.365, 0.411]	[0.684, 0.733]
Guyana	0.768	0.731	0.214	0.616	0.833
(2009)	[0.744, 0.792]	[0.703, 0.758]	[0.186, 0.243]	[0.573, 0.658]	[0.807, 0.860]
India	0.774	0.743	0.109	0.253	0.611
(2015–16)	[0.771, 0.777]	[0.740, 0.746]	[0.106, 0.113]	[0.248, 0.258]	[0.605, 0.616]
Kyrgyz Republic	0.906	0.913	0.638	0.780	0.987
(2012)	[0.891, 0.920]	[0.897, 0.930]	[0.606, 0.671]	[0.754, 0.805]	[0.981, 0.993]
Pakistan	0.629	0.494	0.492	0.441	0.605
(2012–13)	[0.607, 0.651]	[0.463, 0.526]	[0.451, 0.533]	[0.406, 0.477]	[0.565, 0.645]
Peru	0.794	0.798	0.174	0.397	0.804
(2009,2010,2011,2012)	[0.788, 0.800]	[0.792, 0.805]	[0.164, 0.184]	[0.385, 0.410]	[0.794, 0.813]
Tajikistan	0.880	0.777	0.168	0.417	0.968
(2012)	[0.864, 0.896]	[0.755, 0.798]	[0.145, 0.190]	[0.377, 0.457]	[0.957, 0.979]
Türkiye	0.766	0.697	0.903	0.968	0.968
(2008)	[0.746, 0.785]	[0.674, 0.720]	[0.886, 0.921]	[0.959, 0.976]	[0.961, 0.976]
Pooled	0.835	0.812	0.409	0.628	0.904
	[0.827, 0.843]	[0.803, 0.821]	[0.390, 0.427]	[0.612, 0.643]	[0.897, 0.911]
**Latest surveys (~2013–21)**					
Albania	0.855	0.873	0.925	0.956	0.984
(2017–18)	[0.837, 0.873]	[0.855, 0.891]	[0.913, 0.938]	[0.948, 0.965]	[0.979, 0.989]
Armenia	0.875	0.898	0.952	0.967	0.996
(2015–16)	[0.858, 0.893]	[0.879, 0.917]	[0.940, 0.964]	[0.957, 0.978]	[0.993, 0.999]
Egypt	0.845	0.818	0.784	0.971	0.985

	School attendance		Ownership of assets		
	Males	Females	Washer	Fridge	TV
(2014)	[0.835, 0.855]	[0.805, 0.830]	[0.771, 0.798]	[0.967, 0.976]	[0.982, 0.989]
India	0.819	0.786	0.151	0.331	0.640
(2019–21)	[0.816, 0.821]	[0.783, 0.789]	[0.148, 0.155]	[0.326, 0.336]	[0.635, 0.645]
Indonesia	0.813	0.832	0.350	0.612	0.908
(2017)	[0.804, 0.822]	[0.824, 0.841]	[0.335, 0.365]	[0.597, 0.627]	[0.899, 0.917]
Pakistan	0.691	0.570	0.561	0.524	0.602
(2017–18)	[0.668, 0.714]	[0.540, 0.600]	[0.525, 0.597]	[0.492, 0.556]	[0.569, 0.635]
Philippines	0.849	0.892	0.403	0.406	0.788
(2017)	[0.837, 0.861]	[0.882, 0.902]	[0.372, 0.433]	[0.379, 0.433]	[0.769, 0.807]
South Africa	0.919	0.903	0.375	0.793	0.809
(2016)	[0.908, 0.931]	[0.891, 0.916]	[0.341, 0.409]	[0.764, 0.821]	[0.782, 0.837]
Tajikistan	0.901	0.832	0.352	0.661	0.982
(2017)	[0.890, 0.912]	[0.816, 0.848]	[0.316, 0.389]	[0.625, 0.697]	[0.977, 0.988]
Türkiye	0.824	0.800	0.959	0.986	0.980
(2013)	[0.807, 0.841]	[0.781, 0.819]	[0.947, 0.971]	[0.980, 0.992]	[0.974, 0.986]
Pooled	0.874	0.864	0.626	0.813	0.911
	[0.868, 0.880]	[0.857, 0.871]	[0.607, 0.645]	[0.801, 0.825]	[0.902, 0.920]

Notes: Estimates were weighted using sampling weights rescaled to sum up to one in each survey. Estimates from pooled sample were further rescaled such that each country contributed equally to the estimates. 95% confidence intervals are shown in brackets. Surveys were divided into the periods indicated in the top of each panel when possible: in some countries other periodization were used (indicated below country names).

SUPPLEMENT 2: Full model outputs for main results

Table S6A. Results from Poisson regression models of school attendance on washer ownership

	Pooled	Albania	Armenia	Azerbaijan	Colombia
Female	.993	1	1.077***	.927***	1.034***
	[.985, 1.001]	[.976, 1.025]	[1.05, 1.105]	[.899, .956]	[1.019, 1.05]
Household owns washing machine	1.017***	1.017	1.016	1.016	.983*
	[1.005, 1.029]	[.967, 1.068]	[.976, 1.058]	[.981, 1.052]	[.965, 1.002]
Household owns refrigerator	1.03***	1.03	1.044	.9956	1.025**
	[1.016, 1.044]	[.963, 1.102]	[.991, 1.1]	[.956, 1.036]	[1, 1.051]
Household owns TV	1.028***	.943	1.071	.95	1.04**
	[1.009, 1.048]	[.844, 1.053]	[.976, 1.176]	[.892, 1.012]	[1.003, 1.079]
Has flush toilet	1.012	1.007	.98	.958	1.002
	[.995, 1.029]	[.925, 1.098]	[.939, 1.023]	[.884, 1.04]	[.964, 1.042]
Household wealth index z-score	1.037***	1.001	1.056***	1.04**	1.098***
	[1.028, 1.045]	[.979, 1.024]	[1.029, 1.084]	[1.006, 1.076]	[1.075, 1.121]
Household wealth index z-score squared	1.001	.993	1.013**	.988	1.018***
	[.9975, 1.005]	[.981, 1.005]	[1.001, 1.024]	[.973, 1.004]	[1.008, 1.028]
Age (years)	1.466***	1.549***	1.568***	1.453***	1.455***
	[1.435, 1.497]	[1.455, 1.65]	[1.456, 1.687]	[1.339, 1.578]	[1.399, 1.513]
Age (years) squared	.984***	.982***	.981***	.985***	.984***
	[.983, .985]	[.98, .985]	[.979, .984]	[.982, .988]	[.982, .985]
Age of household head (years)	.9978*	.982***	.9995	.9985	1.009***
	[.9956, 1]	[.973, .99]	[.992, 1.007]	[.99, 1.007]	[1.004, 1.013]
Age of household head (years) squared	1*	1***	1	1	.9999***
	[1, 1]	[1, 1]	[.9999, 1]	[.9999, 1]	[.9999, 1]
Number of household members	.991***	.967**	1.006	.9977	.985**
	[.987, .996]	[.938, .9983]	[.956, 1.059]	[.964, 1.032]	[.969, 1]
Number of household members squared	1***	1.003**	.9994	1	1
	[1, 1]	[1, 1.005]	[.9955, 1.003]	[.9977, 1.002]	[.9993, 1.001]
Number of household members under 5 years	.978***	1.021	.949	.916**	1.004
	[.969, .988]	[.938, 1.11]	[.886, 1.018]	[.85, .986]	[.981, 1.027]
Number of household members under 5 years squared	1.003***	.953	1.016	1.033**	.994
	[1.001, 1.005]	[.893, 1.016]	[.974, 1.06]	[1.004, 1.062]	[.985, 1.002]
Highest female education level in household: Primary (rc)					
None	.986	.91	1.206	.757	.978
	[.969, 1.003]	[.752, 1.101]	[.52, 2.798]	[.425, 1.349]	[.937, 1.02]
Secondary	1.014*	1.036**	1.014	1.088	1.049***
	[.9987, 1.029]	[1.006, 1.068]	[.929, 1.107]	[.869, 1.361]	[1.028, 1.071]
Higher	1.052***	1.077***	1.083*	1.077	1.059***
	[1.034, 1.071]	[1.031, 1.124]	[.995, 1.18]	[.86, 1.349]	[1.032, 1.087]
No female in household	.983	1.035	1.004	1.197	.854***
	[.951, 1.016]	[.883, 1.212]	[.764, 1.319]	[.911, 1.573]	[.802, .91]
Highest male education level in household: Primary (rc)					
None	.944***	.883	.668	1.162**	.94***
	[.924, .964]	[.716, 1.088]	[.307, 1.457]	[1.005, 1.345]	[.902, .981]
Secondary	1.02***	1.083***	.9983	.981	1.013
	[1.005, 1.036]	[1.051, 1.115]	[.937, 1.064]	[.903, 1.065]	[.994, 1.033]
Higher	1.052***	1.066***	1.044	1.019	1.024*
	[1.035, 1.069]	[1.022, 1.113]	[.977, 1.115]	[.93, 1.116]	[.9974, 1.051]
No male in household	1.008	.9984	.977	1.016	.971**
	[.989, 1.027]	[.951, 1.048]	[.906, 1.054]	[.932, 1.108]	[.945, .9986]

	Pooled	Albania	Armenia	Azerbaijan	Colombia
**Variables interacted with being Female:**					
Household owns washing machine	1.003	1.077**	1.024	.996	1.034***
	[.987, 1.019]	[1.005, 1.155]	[.968, 1.084]	[.936, 1.06]	[1.009, 1.06]
Household owns refrigerator	.987	.941	.95	.992	.998
	[.968, 1.007]	[.852, 1.039]	[.884, 1.02]	[.913, 1.077]	[.968, 1.029]
Household owns TV	1.005	1.121	1.019	1.098*	.977
	[.982, 1.029]	[.949, 1.324]	[.922, 1.127]	[.984, 1.225]	[.934, 1.021]
Has flush toilet	1.007	1.109*	1.054*	1.019	.9963
	[.989, 1.025]	[.981, 1.254]	[.9975, 1.114]	[.947, 1.096]	[.954, 1.04]
Household wealth index z-score	1.014***	1.028**	.993	1.022	.969***
	[1.005, 1.023]	[1.004, 1.053]	[.963, 1.024]	[.979, 1.066]	[.952, .985]
Household wealth index z-score squared	.9962*	1.004	.994	1.009	.987***
	[.992, 1.001]	[.991, 1.018]	[.979, 1.01]	[.986, 1.033]	[.977, .9967]
Age (years)	.989	.855***	.908**	1.173**	.966
	[.961, 1.018]	[.784, .932]	[.831, .992]	[1.03, 1.336]	[.912, 1.023]
Age (years) squared	1	1.006***	1.004**	.994***	1.002
	[.9993, 1.001]	[1.002, 1.009]	[1.001, 1.008]	[.989, .9984]	[.9993, 1.004]
Age of household head (years)	1.001	1.004	.985***	.9984	1.001
	[.9983, 1.005]	[.993, 1.016]	[.975, .995]	[.985, 1.012]	[.9956, 1.007]
Age of household head (years) squared	1	1	1***	1	1
	[1, 1]	[.9999, 1]	[1, 1]	[.9999, 1]	[.9999, 1]
Number of household members	1.004	1.046	.972	.9958	1.009
	[.9979, 1.01]	[.978, 1.119]	[.913, 1.034]	[.933, 1.063]	[.99, 1.029]
Number of household members squared	.9999	.9958	1.002	.9995	1
	[.9996, 1]	[.99, 1.002]	[.9972, 1.007]	[.995, 1.004]	[.9987, 1.001]
Number of household members under 5 years	.97***	.922	.972	1.066	.937***
	[.956, .983]	[.82, 1.036]	[.886, 1.067]	[.939, 1.209]	[.906, .969]
Number of household members under 5 years squared	1.001	1.05	1.001	.918**	1.003
	[.9977, 1.004]	[.962, 1.146]	[.945, 1.059]	[.849, .993]	[.99, 1.016]
Highest female education level in household: Primary (rc)					
None	.936***	.755	.728	.797	.981
	[.915, .957]	[.538, 1.06]	[.286, 1.853]	[.343, 1.852]	[.927, 1.039]
Secondary	1.007	1.058**	.991	1.282	.977*
	[.988, 1.025]	[1.012, 1.107]	[.892, 1.1]	[.837, 1.963]	[.951, 1.004]
Higher	.995	.989	.99	1.266	.956***
	[.973, 1.018]	[.932, 1.05]	[.894, 1.095]	[.828, 1.935]	[.924, .989]
No female in household	.898***	1.05	1.054	1.206	.817***
	[.854, .944]	[.852, 1.294]	[.775, 1.435]	[.756, 1.924]	[.75, .89]
Highest male education level in household: Primary (rc)					
None	1.007	1.028	2.501**	1.187	1.024
	[.979, 1.036]	[.731, 1.445]	[1.011, 6.189]	[.592, 2.379]	[.969, 1.081]
Secondary	.9989	.971	1.093*	1.31	.992
	[.979, 1.019]	[.93, 1.013]	[.986, 1.212]	[.668, 2.569]	[.966, 1.019]
Higher	.99	.969	1.074	1.365	.981
	[.968, 1.013]	[.912, 1.028]	[.966, 1.193]	[.694, 2.686]	[.947, 1.015]
No male in household	.9992	1.032	1.121*	1.259	1.008
	[.975, 1.024]	[.961, 1.108]	[.9972, 1.26]	[.638, 2.486]	[.974, 1.043]
Constant	.799***	.808***	.79***	.881***	.752***
	[.794, .805]	[.793, .823]	[.774, .806]	[.865, .898]	[.744, .761]
**Combinations of parameters:**					
Washer×Female-washer ownership interaction	1.019***	1.095***	1.04*	1.012	1.017*
	[1.006, 1.033]	[1.034, 1.16]	[.994, 1.088]	[.949, 1.079]	[.9975, 1.037]

	Pooled	Albania	Armenia	Azerbaijan	Colombia
Constant×Female	.794***	.808***	.851***	.817***	.778***
	[.788, .799]	[.794, .822]	[.839, .863]	[.8, .834]	[.77, .786]
Observations	1,614,264	13,749	10,925	6,203	61,245

Notes: *P<0.1; **P<0.05; ***P<0.01. Prevalence ratios are shown (ie, exponentiated coefficients): 95% confidence intervals adjusted for clustering at the level of primary sampling units are shown in brackets below. Each column of coefficients and the corresponding confidence intervals were obtained from a separate model. All models adjusted for neighborhoods by included neighborhood level means of all independent variables (including the interaction terms). Except the terms for female, all independent variables, including neighborhood level means, were centered around a country-specific weighted mean: Therefore, the constant shows school attendance for males with the country-level average on all independent variables. Estimates were weighted using sampling weights, rescaled to sum up to one for the analytical sample from each survey. Pooled models were further rescaled such that each country contributed equally to the estimates.

Table S6B. Results from Poisson regression models of school attendance on washer ownership

	Egypt	Gabon	Guatemala	Guyana	India
Female	.909***	1.001	.872***	1.019	.958***
	[.9, .919]	[.977, 1.025]	[.848, .896]	[.974, 1.066]	[.955, .961]
Household owns washing machine	1.017**	.972	.986	.978	.992**
	[1.001, 1.033]	[.905, 1.043]	[.939, 1.035]	[.926, 1.033]	[.985, .9987]
Household owns refrigerator	1.054***	1.018	1.029	.994	.998
	[1.03, 1.079]	[.974, 1.064]	[.994, 1.066]	[.935, 1.058]	[.992, 1.004]
Household owns TV	1	.987	.9956	1.015	.981***
	[.966, 1.036]	[.922, 1.057]	[.957, 1.036]	[.942, 1.094]	[.976, .987]
Has flush toilet	1.001	.9983	.992	1.053*	.993***
	[.954, 1.05]	[.949, 1.051]	[.955, 1.031]	[.9993, 1.11]	[.988, .9981]
Household wealth index z-score	1.069***	1.035*	1.117***	1.077***	1.101***
	[1.058, 1.079]	[.9994, 1.072]	[1.078, 1.156]	[1.033, 1.124]	[1.096, 1.106]
Household wealth index z-score squared	1.011***	.994	.988	1.009	.983***
	[1.006, 1.016]	[.976, 1.014]	[.971, 1.006]	[.995, 1.024]	[.981, .986]
Age (years)	1.39***	1.138***	1.397***	1.912***	1.543***
	[1.36, 1.421]	[1.059, 1.223]	[1.324, 1.473]	[1.698, 2.153]	[1.531, 1.556]
Age (years) squared	.986***	.994***	.984***	.973***	.982***
	[.985, .987]	[.991, .9968]	[.982, .986]	[.968, .977]	[.982, .982]
Age of household head (years)	1.004*	1.003	1.003	1.002	.9975***
	[.9994, 1.008]	[.992, 1.014]	[.996, 1.01]	[.993, 1.012]	[.9964, .9985]
Age of household head (years) squared	1*	1	1	1	1***
	[.9999, 1]	[.9999, 1]	[.9999, 1]	[.9999, 1]	[1, 1]
Number of household members	.9963	1.013	.994	.99	.986***
	[.99, 1.003]	[.994, 1.032]	[.973, 1.016]	[.958, 1.024]	[.983, .988]
Number of household members squared	1	.9995	1	.9999	1***
	[.9997, 1]	[.9987, 1]	[.9988, 1.002]	[.9977, 1.002]	[1, 1.001]
Number of household members under 5 years	.99*	.952**	.96**	.951*	.966***
	[.978, 1.002]	[.915, .991]	[.928, .993]	[.899, 1.006]	[.961, .972]
Number of household members under 5 years squared	1.005***	1.006*	1	1.014	1.005***
	[1.002, 1.008]	[.9998, 1.012]	[.987, 1.013]	[.9957, 1.033]	[1.003, 1.007]
Highest female education level in household: Primary (rc)					
None	.961***	.986	.979	1.13*	.956***
	[.944, .978]	[.912, 1.066]	[.942, 1.018]	[.98, 1.303]	[.951, .962]
Secondary	1.032***	1.011	1.015	1.003	1.017***
	[1.016, 1.048]	[.969, 1.054]	[.976, 1.055]	[.957, 1.051]	[1.012, 1.023]
Higher	1.007	1.043	1.005	1.04	1.002
	[.986, 1.029]	[.975, 1.117]	[.945, 1.069]	[.962, 1.124]	[.994, 1.01]
No female in household	.926**	.901*	.89**	.914	.82***
	[.859, .9973]	[.809, 1.004]	[.811, .977]	[.806, 1.036]	[.802, .839]
Highest male education level in household: Primary (rc)					
None	.931***	.924	.945**	.89	.932***
	[.912, .95]	[.832, 1.025]	[.906, .987]	[.701, 1.131]	[.925, .938]
Secondary	1.033***	.9959	1.045**	1.002	1.042***
	[1.017, 1.05]	[.95, 1.043]	[1.007, 1.085]	[.949, 1.057]	[1.036, 1.048]
Higher	1.09***	.994	1.036	1.079**	1.052***
	[1.069, 1.111]	[.938, 1.054]	[.977, 1.098]	[1.003, 1.162]	[1.044, 1.06]
No male in household	.973*	.9961	1.002	1	.952***
	[.946, 1]	[.942, 1.054]	[.967, 1.037]	[.937, 1.067]	[.944, .96]
**Variables interacted with being Female:**					
Household owns washing machine	1.024**	1.033	.99	1.041	1.003

	Egypt	Gabon	Guatemala	Guyana	India
	[1.003, 1.045]	[.952, 1.122]	[.925, 1.059]	[.964, 1.124]	[.995, 1.012]
Household owns refrigerator	1.068***	.984	.993	1.005	.992**
	[1.035, 1.102]	[.921, 1.051]	[.948, 1.041]	[.928, 1.089]	[.984, .9991]
Household owns TV	1.024	1.044	1.054*	.987	1.01***
	[.971, 1.08]	[.952, 1.144]	[.994, 1.117]	[.892, 1.091]	[1.003, 1.017]
Has flush toilet	1.289***	1.004	1.072**	.944*	1.022***
	[1.185, 1.403]	[.933, 1.081]	[1.015, 1.132]	[.885, 1.008]	[1.016, 1.029]
Household wealth index z-score	1.02***	1.007	1.022	.999	.995*
	[1.01, 1.03]	[.961, 1.055]	[.976, 1.07]	[.946, 1.055]	[.989, 1]
Household wealth index z-score squared	.988***	.994	1.016	.985*	1.004***
	[.981, .994]	[.974, 1.014]	[.993, 1.04]	[.968, 1.003]	[1.001, 1.008]
Age (years)	.991	1.083	.885***	1.218**	1.065***
	[.958, 1.024]	[.973, 1.206]	[.814, .962]	[1.041, 1.426]	[1.053, 1.078]
Age (years) squared	1	.9972	1.003**	.993**	.9972***
	[.9987, 1.001]	[.993, 1.001]	[1, 1.007]	[.987, .999]	[.9968, .9977]
Age of household head (years)	1.017***	1.001	1	1.004	1.004***
	[1.01, 1.024]	[.988, 1.014]	[.99, 1.01]	[.99, 1.019]	[1.003, 1.006]
Age of household head (years) squared	.9998***	1	1	.9999	1***
	[.9998, .9999]	[.9999, 1]	[.9999, 1]	[.9998, 1]	[.9999, 1]
Number of household members	.986***	1.003	.989	1.018	1.001
	[.977, .9953]	[.98, 1.026]	[.958, 1.021]	[.983, 1.055]	[.9984, 1.004]
Number of household members squared	1	1	1	.9996	1
	[.9999, 1.001]	[.9991, 1.001]	[.998, 1.002]	[.9977, 1.002]	[.9999, 1]
Number of household members under 5 years	.975***	1.004	.957**	1.023	.962***
	[.958, .992]	[.963, 1.048]	[.919, .9974]	[.953, 1.098]	[.955, .969]
Number of household members under 5 years squared	.9983	.994	1.012*	.981*	1.003***
	[.994, 1.003]	[.986, 1.002]	[.9998, 1.025]	[.958, 1.003]	[1.001, 1.006]
Highest female education level in household: Primary (rc)					
None	.966***	.97	.923***	.852*	.947***
	[.944, .988]	[.863, 1.09]	[.876, .973]	[.71, 1.023]	[.94, .954]
Secondary	1.007	.992	.983	1.04	1.011***
	[.987, 1.028]	[.946, 1.041]	[.932, 1.037]	[.983, 1.1]	[1.004, 1.018]
Higher	1.014	.978	.95	1.03	1.007
	[.986, 1.043]	[.904, 1.059]	[.869, 1.039]	[.923, 1.15]	[.9958, 1.018]
No female in household	.434***	.973	.638***	.968	.798***
	[.381, .494]	[.848, 1.115]	[.537, .758]	[.788, 1.19]	[.772, .825]
Highest male education level in household: Primary (rc)					
None	.969**	.939	.992	1.23	.9964
	[.942, .9969]	[.803, 1.097]	[.929, 1.059]	[.929, 1.63]	[.987, 1.006]
Secondary	.993	.991	.954*	1.024	.983***
	[.971, 1.015]	[.934, 1.051]	[.91, 1.001]	[.958, 1.095]	[.976, .991]
Higher	.964***	1.027	.98	.961	.985***
	[.939, .99]	[.951, 1.11]	[.898, 1.07]	[.864, 1.07]	[.976, .9955]
No male in household	1.001	.9982	1.008	.9999	1.011*
	[.965, 1.039]	[.939, 1.061]	[.96, 1.059]	[.92, 1.087]	[.9991, 1.022]
Constant	.758***	.89***	.666***	.691***	.756***
	[.752, .763]	[.875, .906]	[.652, .68]	[.666, .716]	[.754, .758]

Combinations of parameters:

	Egypt	Gabon	Guatemala	Guyana	India
Washer×Female-washer ownership interaction	1.041***	1.004	.976	1.018	.995
	[1.023, 1.059]	[.949, 1.062]	[.926, 1.029]	[.959, 1.08]	[.988, 1.002]
Constant×Female	.689***	.891***	.581***	.704***	.724***
	[.682, .695]	[.878, .905]	[.566, .595]	[.682, .726]	[.722, .726]
Observations	102,079	8,462	24,453	6,801	1,075,968

Notes: *P<0.1; **P<0.05; ***P<0.01. Prevalence ratios are shown (ie, exponentiated coefficients): 95% confidence intervals adjusted for clustering at the level of primary sampling units are shown in brackets below. Each column of coefficients and the corresponding confidence intervals were obtained from a separate model. All models adjusted for neighborhood fixed effects by included neighborhood level means of all independent variables (including the interaction terms). Except the terms for female, all independent variables, including fixed effects, were centered around a country-specific weighted mean: Therefore, the constant shows school attendance for males with the country-level average on all independent variables. Estimates were weighted using sampling weights, rescaled to sum up to one for the analytical sample each survey. Pooled models were further rescaled such that each country contributed equally to the estimates.

Table S6C. Results from Poisson regression models of school attendance on washer ownership

	Indonesia	Kyrgyz Republic	Moldova	Morocco	Pakistan
Female	1.03***	1.025**	1.088***	.812***	.698***
	[1.014, 1.047]	[1.004, 1.046]	[1.061, 1.115]	[.781, .844]	[.678, .719]
Household owns washing machine	.991	.982	1.047*	1.003	.982
	[.971, 1.011]	[.952, 1.012]	[.9995, 1.097]	[.945, 1.064]	[.946, 1.019]
Household owns refrigerator	1.009	1.022	1.046	1.152***	1.122***
	[.986, 1.033]	[.993, 1.051]	[.977, 1.121]	[1.082, 1.226]	[1.084, 1.162]
Household owns TV	.977	.992	1.008	1.077**	.963**
	[.937, 1.017]	[.902, 1.092]	[.953, 1.066]	[1.01, 1.148]	[.929, .9977]
Has flush toilet	.948***	.975	1.047	1.041	.9967
	[.916, .98]	[.9, 1.057]	[.98, 1.118]	[.95, 1.141]	[.95, 1.046]
Household wealth index z-score	1.069***	.985	1.089***	.988	1.108***
	[1.051, 1.088]	[.957, 1.015]	[1.043, 1.137]	[.927, 1.052]	[1.071, 1.146]
Household wealth index z-score squared	.993	1.012	.969***	.98	.983**
	[.984, 1.001]	[.9951, 1.03]	[.947, .992]	[.949, 1.013]	[.966, .9997]
Age (years)	1.843***	1.538***	1.507***	1.183***	1.319***
	[1.768, 1.92]	[1.427, 1.657]	[1.407, 1.614]	[1.099, 1.273]	[1.265, 1.376]
Age (years) squared	.975***	.983***	.984***	.99***	.987***
	[.974, .977]	[.98, .986]	[.981, .986]	[.987, .993]	[.985, .988]
Age of household head (years)	1.001	.992**	.9952	.995	1.004
	[.994, 1.007]	[.986, .9987]	[.985, 1.005]	[.984, 1.006]	[.9976, 1.011]
Age of household head (years) squared	1	1*	1	1	.9999
	[.9999, 1]	[1, 1]	[1, 1]	[.9999, 1]	[.9999, 1]
Number of household members	.978***	.987	.972	.985	.985***
	[.963, .993]	[.968, 1.007]	[.909, 1.04]	[.966, 1.005]	[.976, .995]
Number of household members squared	1.001	1	1.001	1	1*
	[.9998, 1.002]	[.9992, 1.002]	[.994, 1.008]	[.9995, 1.001]	[1, 1.001]
Number of household members under 5 years	.9994	.97	.932	.961*	1.009
	[.973, 1.026]	[.934, 1.007]	[.843, 1.029]	[.919, 1.006]	[.985, 1.033]
Number of household members under 5 years squared	.9982	1.014**	1.034	1.014	.994**
	[.986, 1.01]	[1.001, 1.026]	[.972, 1.099]	[.9973, 1.03]	[.99, .9992]
Highest female education level in household: Primary (rc)					
None	1.05*	.9999	1.122	1.009	.945***
	[.9986, 1.105]	[.851, 1.175]	[.704, 1.787]	[.964, 1.057]	[.91, .981]
Secondary	1.054***	.938	1.249*	1.002	.988
	[1.035, 1.074]	[.794, 1.107]	[.964, 1.62]	[.95, 1.057]	[.95, 1.028]
Higher	1.063***	.974	1.304**	1.058	.997
	[1.034, 1.092]	[.821, 1.154]	[1.007, 1.689]	[.975, 1.148]	[.952, 1.044]
No female in household	.918**	.979	1.22	1.05	.629***
	[.846, .995]	[.819, 1.17]	[.935, 1.59]	[.886, 1.244]	[.551, .718]
Highest male education level in household: Primary (rc)					
None	.942	.789	.793	.98	.896***
	[.873, 1.016]	[.531, 1.172]	[.417, 1.506]	[.939, 1.022]	[.854, .941]
Secondary	1.012	.928	1.025	1.078***	1.11***
	[.991, 1.034]	[.784, 1.099]	[.825, 1.274]	[1.03, 1.128]	[1.067, 1.154]
Higher	1.043***	.946	1.058	1.076**	1.219***
	[1.014, 1.073]	[.799, 1.12]	[.851, 1.316]	[1.008, 1.147]	[1.167, 1.272]
No male in household	.969	.984	.992	.914**	1.057*
	[.933, 1.008]	[.833, 1.164]	[.794, 1.24]	[.85, .982]	[.9974, 1.121]

	Indonesia	Kyrgyz Republic	Moldova	Morocco	Pakistan
**Variables interacted with being Female:**					
Household owns washing machine	1.006	1.011	.973	.943	1.04
	[.981, 1.032]	[.977, 1.047]	[.919, 1.031]	[.872, 1.019]	[.988, 1.095]
Household owns refrigerator	1	1.017	.987	.932	.929***
	[.972, 1.03]	[.972, 1.064]	[.908, 1.072]	[.85, 1.023]	[.885, .975]
Household owns TV	1.043	.991	1.025	.942	1.107***
	[.986, 1.103]	[.793, 1.237]	[.955, 1.1]	[.862, 1.03]	[1.052, 1.165]
Has flush toilet	1.049**	1.068	.963	.971	1.146***
	[1.008, 1.092]	[.984, 1.16]	[.89, 1.042]	[.866, 1.088]	[1.07, 1.228]
Household wealth index z-score	.984*	1.018	.991	1.234***	1.159***
	[.965, 1.003]	[.993, 1.044]	[.944, 1.041]	[1.155, 1.319]	[1.108, 1.212]
Household wealth index z-score squared	1.001	.995	1.009	.957**	.947***
	[.99, 1.012]	[.977, 1.013]	[.983, 1.036]	[.917, .9994]	[.926, .969]
Age (years)	.929**	.917**	.842***	.934	.892***
	[.877, .984]	[.841, .9992]	[.77, .92]	[.83, 1.05]	[.831, .956]
Age (years) squared	1.003***	1.003**	1.007***	1.002	1.003**
	[1.001, 1.005]	[1, 1.007]	[1.004, 1.01]	[.9973, 1.006]	[1, 1.005]
Age of household head (years)	1.005	1	1.017***	1.021**	1.012**
	[.9966, 1.014]	[.992, 1.009]	[1.004, 1.029]	[1.004, 1.039]	[1.003, 1.022]
Age of household head (years) squared	1	1	.9998***	.9998***	.9999***
	[.9999, 1]	[.9999, 1]	[.9997, 1]	[.9996, .9999]	[.9998, 1]
Number of household members	1.003	1.012	.971	1.018	.9954
	[.983, 1.022]	[.983, 1.043]	[.89, 1.058]	[.99, 1.047]	[.983, 1.008]
Number of household members squared	1	.9994	1.003	.9997	.9999
	[.999, 1.001]	[.9975, 1.001]	[.995, 1.012]	[.9984, 1.001]	[.9995, 1]
Number of household members under 5 years	.971	.9983	.875*	.955	.914***
	[.934, 1.008]	[.952, 1.046]	[.764, 1.001]	[.895, 1.018]	[.884, .945]
Number of household members under 5 years squared	.9998	.9953	1.053	1.005	1.008**
	[.981, 1.018]	[.978, 1.013]	[.969, 1.146]	[.984, 1.027]	[1.002, 1.014]
Highest female education level in household: Primary (rc)					
None	.891***	1.119	.658	.915***	.841***
	[.825, .962]	[.914, 1.37]	[.338, 1.281]	[.856, .977]	[.801, .882]
Secondary	.989	1.126	.796**	.988	1.029
	[.965, 1.014]	[.932, 1.36]	[.65, .974]	[.917, 1.065]	[.98, 1.081]
Higher	.98	1.114	.796**	.912	1.035
	[.947, 1.015]	[.92, 1.349]	[.647, .979]	[.81, 1.026]	[.974, 1.099]
No female in household	1.018	1.091	.766**	.681***	.607***
	[.925, 1.12]	[.881, 1.352]	[.62, .946]	[.52, .89]	[.483, .764]
Highest male education level in household: Primary (rc)					
None	1.006	1.531	1.099	1.008	1.05
	[.91, 1.111]	[.848, 2.766]	[.58, 2.083]	[.95, 1.069]	[.985, 1.12]
Secondary	.981	1.374*	.931	.938*	1.043
	[.955, 1.008]	[.965, 1.958]	[.689, 1.258]	[.872, 1.009]	[.984, 1.105]
Higher	.953**	1.36*	.908	.937	.981
	[.917, .99]	[.95, 1.948]	[.671, 1.227]	[.844, 1.04]	[.922, 1.044]
No male in household	1.023	1.288	.922	1.109**	.9983
	[.972, 1.076]	[.906, 1.831]	[.679, 1.25]	[1.001, 1.229]	[.923, 1.08]
Constant	.771***	.881***	.819***	.584***	.511***
	[.762, .78]	[.866, .897]	[.802, .836]	[.567, .601]	[.5, .523]
**Combinations of parameters:**					
Washer×Female-washer ownership interaction	.9971	.993	1.019	.945*	1.021
	[.979, 1.016]	[.965, 1.021]	[.982, 1.058]	[.886, 1.008]	[.976, 1.069]

	Indonesia	Kyrgyz Republic	Moldova	Morocco	Pakistan
Constant×Female	.794***	.903***	.891***	.474***	.357***
	[.785, .804]	[.89, .917]	[.877, .905]	[.456, .493]	[.346, .368]
Observations	35,687	6,551	5,457	14,022	70,840

Notes: *P<0.1; **P<0.05; ***P<0.01. Prevalence ratios are shown (ie, exponentiated coefficients): 95% confidence intervals adjusted for clustering at the level of primary sampling units are shown in brackets below. Each column of coefficients and the corresponding confidence intervals were obtained from a separate model. All models adjusted for neighborhood fixed effects by included neighborhood level means of all independent variables (including the interaction terms). Except the terms for female, all independent variables, including fixed effects, were centered around a country-specific weighted mean: Therefore, the constant shows school attendance for males with the country-level average on all independent variables. Estimates were weighted using sampling weights, rescaled to sum up to one for the analytical sample each survey. Pooled models were further rescaled such that each country contributed equally to the estimates.

Table S6D. Results from Poisson regression models of school attendance on washer ownership

	Peru	Philippines	South Africa	Tajikistan	Türkiye
Female	.9975	1.061***	.977**	.869***	.874***
	[.987, 1.008]	[1.044, 1.077]	[.958, .9968]	[.85, .889]	[.853, .894]
Household owns washing machine	1.002	.994	1.025	.9997	.991
	[.977, 1.027]	[.97, 1.018]	[.99, 1.061]	[.978, 1.022]	[.947, 1.037]
Household owns refrigerator	.994	1.031**	1.044*	.9978	1.117**
	[.976, 1.012]	[1.005, 1.057]	[.9997, 1.09]	[.974, 1.022]	[1.017, 1.226]
Household owns TV	.989	1.017	1.016	.986	.864***
	[.971, 1.008]	[.978, 1.057]	[.979, 1.055]	[.933, 1.043]	[.803, .929]
Has flush toilet	.985	1.04**	.961	1.009	.972
	[.965, 1.005]	[1.003, 1.079]	[.903, 1.023]	[.962, 1.058]	[.933, 1.013]
Household wealth index z-score	1.064***	1.065***	1.059***	1.015	1.039***
	[1.047, 1.082]	[1.043, 1.088]	[1.025, 1.094]	[.99, 1.041]	[1.023, 1.055]
Household wealth index z-score squared	1.01**	1.006	1.025***	1.017***	.9955
	[1.001, 1.02]	[.9958, 1.016]	[1.008, 1.042]	[1.005, 1.029]	[.985, 1.006]
Age (years)	1.605***	1.283***	1.244***	1.58***	1.568***
	[1.558, 1.653]	[1.231, 1.337]	[1.183, 1.308]	[1.507, 1.656]	[1.49, 1.65]
Age (years) squared	.98***	.989***	.991***	.982***	.981***
	[.979, .981]	[.987, .99]	[.989, .993]	[.981, .984]	[.979, .983]
Age of household head (years)	1	.9981	1.001	1	.995
	[.9971, 1.003]	[.989, 1.008]	[.9964, 1.006]	[.996, 1.005]	[.988, 1.001]
Age of household head (years) squared	1	1	1	1	1*
	[1, 1]	[.9999, 1]	[.9999, 1]	[1, 1]	[1, 1]
Number of household members	.993	.991	.984**	1.002	.979***
	[.981, 1.006]	[.974, 1.009]	[.969, .9996]	[.99, 1.015]	[.963, .995]
Number of household members squared	.9999	.9999	1.001**	.9999	1.001**
	[.999, 1.001]	[.9988, 1.001]	[1, 1.002]	[.9994, 1.001]	[1, 1.002]
Number of household members under 5 years	.978**	.961***	.984	.992	.989
	[.96, .9953]	[.942, .981]	[.958, 1.01]	[.976, 1.009]	[.962, 1.017]
Number of household members under 5 years squared	1.008**	1.01***	1.001	1.003	1.006
	[1.001, 1.016]	[1.004, 1.017]	[.993, 1.01]	[.9993, 1.006]	[.9979, 1.013]
Highest female education level in household: Primary (rc)					
None	.98*	.871***	1.007	.993	.984
	[.958, 1.002]	[.802, .946]	[.944, 1.075]	[.904, 1.089]	[.957, 1.013]
Secondary	1.016*	1.065***	1.032	.976	.992
	[.9999, 1.032]	[1.039, 1.092]	[.993, 1.074]	[.923, 1.033]	[.964, 1.02]
Higher	1.043***	1.075***	1.033	.98	.989
	[1.019, 1.067]	[1.044, 1.106]	[.982, 1.086]	[.923, 1.04]	[.938, 1.044]
No female in household	.952***	.892***	.958	1.129**	1.101
	[.921, .985]	[.84, .947]	[.896, 1.023]	[1.024, 1.244]	[.961, 1.262]
Highest male education level in household: Primary (rc)					
None	.942***	.938	.979	1.022	.947**
	[.905, .98]	[.856, 1.029]	[.917, 1.047]	[.889, 1.175]	[.899, .9967]
Secondary	1.008	1.058***	.994	1.06	1.019
	[.993, 1.024]	[1.031, 1.086]	[.956, 1.033]	[.982, 1.144]	[.9952, 1.044]
Higher	1.028**	1.079***	.985	1.087**	1.014
	[1.005, 1.05]	[1.045, 1.114]	[.922, 1.052]	[1.007, 1.174]	[.97, 1.059]
No male in household	.984	1.041*	.984	1.057	.977
	[.964, 1.005]	[.9975,	[.943, 1.026]	[.972, 1.149]	[.923, 1.033]

	Peru	Philippines	South Africa	Tajikistan	Türkiye
		1.086]			
**Variables interacted with being Female:**					
Household owns washing machine	1.006	1.004	1.01	.987	1.287***
	[.975, 1.038]	[.973, 1.035]	[.965, 1.058]	[.953, 1.023]	[1.193, 1.388]
Household owns refrigerator	1.017	1.002	1.013	1.024	.925
	[.994, 1.041]	[.97, 1.036]	[.95, 1.081]	[.984, 1.065]	[.815, 1.049]
Household owns TV	1.008	.981	1	.959	.97
	[.984, 1.032]	[.937, 1.026]	[.941, 1.063]	[.872, 1.055]	[.858, 1.097]
Has flush toilet	1.008	.947***	.988	.982	1.089***
	[.986, 1.03]	[.909, .986]	[.936, 1.043]	[.936, 1.031]	[1.04, 1.14]
Household wealth index z-score	1.016*	.979	.988	1.011	.995
	[.9975, 1.035]	[.954, 1.005]	[.949, 1.029]	[.98, 1.042]	[.977, 1.012]
Household wealth index z-score squared	.985**	.969***	.992	.997	1.022***
	[.974, .9967]	[.956, .982]	[.973, 1.012]	[.982, 1.012]	[1.009, 1.035]
Age (years)	.941***	1.042	1.051	1.291***	.874***
	[.903, .98]	[.982, 1.107]	[.973, 1.135]	[1.199, 1.389]	[.809, .944]
Age (years) squared	1.002***	.9989	.9981	.99***	1.004***
	[1.001, 1.004]	[.9967, 1.001]	[.9953, 1.001]	[.987, .992]	[1.001, 1.007]
Age of household head (years)	1.002	1.01*	.996	.9977	1.002
	[.9977, 1.007]	[.999, 1.021]	[.988, 1.004]	[.991, 1.005]	[.992, 1.012]
Age of household head (years) squared	1	.9999*	1	1	1
	[.9999, 1]	[.9998, 1]	[1, 1]	[1, 1]	[.9999, 1]
Number of household members	1.006	1.023**	1.01	.985	1.013
	[.991, 1.021]	[1.002, 1.045]	[.989, 1.032]	[.967, 1.004]	[.981, 1.046]
Number of household members squared	1	.9994	.9993	1.001	.9985
	[.999, 1.001]	[.998, 1.001]	[.9982, 1]	[.9997, 1.001]	[.9965, 1]
Number of household members under 5 years	.957***	.954***	.975	.983	.942***
	[.935, .979]	[.925, .984]	[.933, 1.018]	[.958, 1.008]	[.904, .983]
Number of household members under 5 years squared	.9977	1.002	1.003	1	1.006
	[.988, 1.007]	[.991, 1.013]	[.991, 1.015]	[.995, 1.006]	[.993, 1.019]
Highest female education level in household: Primary (rc)					
None	.967**	1.043	1.008	1.052	.869***
	[.939, .9953]	[.943, 1.154]	[.934, 1.086]	[.883, 1.254]	[.831, .909]
Secondary	.992	.96***	1.021	1.003	1.01
	[.972, 1.013]	[.931, .989]	[.969, 1.075]	[.922, 1.092]	[.971, 1.051]
Higher	.97**	.944***	1.036	1.086*	.959
	[.941, .999]	[.91, .98]	[.971, 1.105]	[.994, 1.187]	[.895, 1.028]
No female in household	.859***	.962	.895*	.904	.778**
	[.817, .905]	[.875, 1.058]	[.794, 1.009]	[.729, 1.121]	[.641, .944]
Highest male education level in household: Primary (rc)					
None	1.059**	.994	.937	.659	.904**
	[1, 1.12]	[.888, 1.113]	[.844, 1.041]	[.365, 1.191]	[.828, .986]
Secondary	1.006	.957***	.951*	.995	.979
	[.985, 1.027]	[.926, .989]	[.899, 1.007]	[.871, 1.137]	[.946, 1.013]
Higher	.9963	.962**	.98	1.012	.972
	[.967, 1.026]	[.927, .9986]	[.895, 1.073]	[.885, 1.158]	[.917, 1.029]
No male in household	1.011	.962	.97	.957	1.139***
	[.984, 1.04]	[.913, 1.013]	[.913, 1.032]	[.83, 1.103]	[1.05, 1.236]
Constant	.76***	.768***	.912***	.87***	.726***
	[.754, .767]	[.759, .777]	[.899, .924]	[.859, .88]	[.714, .737]
**Combinations of parameters:**					
Washer×Female-washer ownership interaction	1.008	.9972	1.036*	.987	1.275***
	[.984, 1.032]	[.973, 1.022]	[.9985,	[.96, 1.015]	[1.189,

	Peru	Philippines	South Africa	Tajikistan	Türkiye
			1.074]		1.368]
Constant×Female	.758***	.814***	.891***	.756***	.634***
	[.751, .765]	[.805, .824]	[.878, .904]	[.741, .771]	[.621, .647]
Observations	82,853	39,196	7,000	16,544	26,229

Notes: *P<0.1; **P<0.05; ***P<0.01. Prevalence ratios are shown (ie, exponentiated coefficients): 95% confidence intervals adjusted for clustering at the level of primary sampling units are shown in brackets below. Each column of coefficients and the corresponding confidence intervals were obtained from a separate model. All models adjusted for neighborhood fixed effects by included neighborhood level means of all independent variables (including the interaction terms). Except the terms for female, all independent variables, including fixed effects, were centered around a country-specific weighted mean: Therefore, the constant shows school attendance for males with the country-level average on all independent variables. Estimates were weighted using sampling weights, rescaled to sum up to one for the analytical sample each survey. Pooled models were further rescaled such that each country contributed equally to the estimates.

SUPPLEMENT 3: Tabulated estimates from main Figures

Table S7. Results from Poisson regression models of school attendance on washer ownership

	Constant	Washer	Female	Interaction	Constant× Female	Interaction× Washer	
Pooled	.799***	1.017***	.993	1.003	.794***	1.019***	N=1,614,264
	[.794, .805]	[1.005, 1.029]	[.985, 1.001]	[.987, 1.019]	[.788, .799]	[1.006, 1.033]	
Albania	.808***	1.017	1	1.077**	.808***	1.095***	N=13,749
	[.793, .823]	[.967, 1.068]	[.976, 1.025]	[1.005, 1.155]	[.794, .822]	[1.034, 1.16]	
Armenia	.79***	1.016	1.077***	1.024	.851***	1.04*	N=10,925
	[.774, .806]	[.976, 1.058]	[1.05, 1.105]	[.968, 1.084]	[.839, .863]	[.994, 1.088]	
Azerbaijan	.881***	1.016	.927***	.996	.817***	1.012	N=6,203
	[.865, .898]	[.981, 1.052]	[.899, .956]	[.936, 1.06]	[.8, .834]	[.949, 1.079]	
Colombia	.752***	.983*	1.034***	1.034***	.778***	1.017*	N=61,245
	[.744, .761]	[.965, 1.002]	[1.019, 1.05]	[1.009, 1.06]	[.77, .786]	[.9975, 1.037]	
Egypt	.758***	1.017**	.909***	1.024**	.689***	1.041***	N=102,079
	[.752, .763]	[1.001, 1.033]	[.9, .919]	[1.003, 1.045]	[.682, .695]	[1.023, 1.059]	
Gabon	.89***	.972	1.001	1.033	.891***	1.004	N=8,462
	[.875, .906]	[.905, 1.043]	[.977, 1.025]	[.952, 1.122]	[.878, .905]	[.949, 1.062]	
Guatemala	.666***	.986	.872***	.99	.581***	.976	N=24,453
	[.652, .68]	[.939, 1.035]	[.848, .896]	[.925, 1.059]	[.566, .595]	[.926, 1.029]	
Guyana	.691***	.978	1.019	1.041	.704***	1.018	N=6,801
	[.666, .716]	[.926, 1.033]	[.974, 1.066]	[.964, 1.124]	[.682, .726]	[.959, 1.08]	
India	.756***	.992**	.958***	1.003	.724***	.995	N=1,075,968
	[.754, .758]	[.985, .9987]	[.955, .961]	[.995, 1.012]	[.722, .726]	[.988, 1.002]	
Indonesia	.771***	.991	1.03***	1.006	.794***	.9971	N=35,687
	[.762, .78]	[.971, 1.011]	[1.014, 1.047]	[.981, 1.032]	[.785, .804]	[.979, 1.016]	
Kyrgyz Republic	.881***	.982	1.025**	1.011	.903***	.993	N=6,551
	[.866, .897]	[.952, 1.012]	[1.004, 1.046]	[.977, 1.047]	[.89, .917]	[.965, 1.021]	
Moldova	.819***	1.047*	1.088***	.973	.891***	1.019	N=5,457
	[.802, .836]	[.9995, 1.097]	[1.061, 1.115]	[.919, 1.031]	[.877, .905]	[.982, 1.058]	
Morocco	.584***	1.003	.812***	.943	.474***	.945*	N=14,022
	[.567, .601]	[.945, 1.064]	[.781, .844]	[.872, 1.019]	[.456, .493]	[.886, 1.008]	
Pakistan	.511***	.982	.698***	1.04	.357***	1.021	N=70,840
	[.5, .523]	[.946, 1.019]	[.678, .719]	[.988, 1.095]	[.346, .368]	[.976, 1.069]	
Peru	.76***	1.002	.9975	1.006	.758***	1.008	N=82,853
	[.754, .767]	[.977, 1.027]	[.987, 1.008]	[.975, 1.038]	[.751, .765]	[.984, 1.032]	
Philippines	.768***	.994	1.061***	1.004	.814***	.9972	N=39,196
	[.759, .777]	[.97, 1.018]	[1.044, 1.077]	[.973, 1.035]	[.805, .824]	[.973, 1.022]	
South Africa	.912***	1.025	.977**	1.01	.891***	1.036*	N=7,000
	[.899, .924]	[.99, 1.061]	[.958, .9968]	[.965, 1.058]	[.878, .904]	[.9985, 1.074]	

	Constant	Washer	Female	Interaction	Constant× Female	Interaction× Washer	
Tajikistan	.87***	.9997	.869***	.987	.756***	.987	N=16,544
	[.859, .88]	[.978, 1.022]	[.85, .889]	[.953, 1.023]	[.741, .771]	[.96, 1.015]	
Türkiye	.726***	.991	.874***	1.287***	.634***	1.275***	N=26,229
	[.714, .737]	[.947, 1.037]	[.853, .894]	[1.193, 1.388]	[.621, .647]	[1.189, 1.368]	

Notes: *P<0.1; **P<0.05; ***P<0.01. Rate ratios and a constant from Poisson regression models are shown, in addition to two combinations obtained using post estimation. Each row of estimates was obtained from a separate model. All models included a baseline term for being female and baseline terms and interactions with being female for washer ownership, fridge ownership, TV ownership, having flush toilet, a wealth index z-score, number of household members, number of household members under age five, age, highest education level of a male in household, and highest education level of a female in household, as well as adjusting for neighborhood. Except the baseline term for female, all independent variables were centered around a country-specific weighted mean: Therefore, the 'Constant' column shows school attendance for males with the mean on all other independent variables. The column labelled 'Washer' shows differences in school attendance for males with washer at home. The column labelled 'Interaction' shows interaction terms for females and washer. The column labelled 'Constant×Female' shows school attendance for females with the mean on all other independent variables, obtained using post estimation. The column labelled 'Interaction×Washer' shows difference in school attendance for girls with washer at home, obtained using post estimation. Estimates were weighted using sampling weights, rescaled to sum up to one for the final sample from each survey. Pooled models were further rescaled such that each country contributed equally to the estimates. 95% confidence intervals adjusted for clustering at the level of primary sampling units are shown in brackets below the point estimates.

Table S8. Results from Poisson regression models of school attendance on washer ownership: stratified by survey year

	Constant	Washer	Female	Interaction	Constant× Female	Interaction× Washer	Mean school attendance	Mean washer ownership	
**Earliest surveys (~2000–08)**									
Pooled	.764***	1.018	1.004	1.004	.768***	1.022*	.799	.446	N=179,671
	[.755, .774]	[.9961, 1.041]	[.99, 1.019]	[.976, 1.033]	[.758, .777]	[.9991, 1.046]	[.792, .806]	[.428, .464]	
Albania	.79***	1.023	.951**	1.074	.751***	1.099**	.809	.781	N=6,372
(2008–09)	[.769, .811]	[.955, 1.097]	[.915, .989]	[.976, 1.183]	[.731, .772]	[1.016, 1.19]	[.793, .825]	[.751, .811]	
Armenia	.743***	.978	1.112***	1.015	.826***	.993	.824	.72	N=4,523
(2005)	[.717, .769]	[.917, 1.043]	[1.064, 1.162]	[.941, 1.095]	[.805, .848]	[.941, 1.047]	[.806, .842]	[.693, .747]	
Azerbaijan	.881***	1.016	.927***	.996	.817***	1.012	.871	.207	N=6,203
(2006)	[.865, .898]	[.981, 1.052]	[.899, .956]	[.936, 1.06]	[.8, .834]	[.949, 1.079]	[.857, .885]	[.178, .235]	
Colombia	.717***	.975	1.025**	1.023	.735***	.9982	.768	.242	N=31,782
(2004–05)	[.705, .728]	[.946, 1.006]	[1.005, 1.046]	[.983, 1.065]	[.724, .745]	[.968, 1.029]	[.76, .776]	[.23, .255]	
Egypt	.741***	1.018	.876***	1.028*	.649***	1.047***	.739	.812	N=60,111
(2000,2003,2005)	[.734, .748]	[.9958, 1.042]	[.863, .89]	[.9967, 1.061]	[.64, .658]	[1.02, 1.075]	[.732, .747]	[.803, .821]	
Guyana	.709***	.903**	1.056*	1.16**	.749***	1.047	.791	.148	N=2,243
(2005)	[.677, .744]	[.819, .9953]	[.995, 1.121]	[1.017, 1.322]	[.722, .777]	[.953, 1.151]	[.767, .815]	[.12, .177]	
Moldova	.819***	1.047*	1.088***	.973	.891***	1.019	.875	.653	N=5,457
(2005)	[.802, .836]	[.9995, 1.097]	[1.061, 1.115]	[.919, 1.031]	[.877, .905]	[.982, 1.058]	[.863, .887]	[.627, .679]	
Morocco	.584***	1.003	.812***	.943	.474***	.945*	.605	.138	N=14,022
(2003–04)	[.567, .601]	[.945, 1.064]	[.781, .844]	[.872, 1.019]	[.456, .493]	[.886, 1.008]	[.583, .628]	[.117, .16]	
Pakistan	.334***	1.003	.643***	1.036	.215***	1.038	.355	.445	N=25,609
(2006–07)	[.32, .349]	[.919, 1.094]	[.605, .685]	[.928, 1.156]	[.202, .229]	[.938, 1.149]	[.339, .37]	[.421, .469]	
Philippines	.71***	.9987	1.049***	1.016	.745***	1.015	.764	.297	N=13,840
(2003)	[.698, .723]	[.957, 1.042]	[1.025, 1.074]	[.963, 1.072]	[.732, .758]	[.973, 1.058]	[.754, .773]	[.275, .319]	
Türkiye	.656***	1.018	.759***	1.159**	.498***	1.18***	.668	.734	N=9,509
(2003–04)	[.637, .675]	[.954, 1.086]	[.724, .796]	[1.034, 1.3]	[.477, .52]	[1.066, 1.306]	[.652, .684]	[.706, .763]	
**Mid surveys (~2008–16)**									
Pooled	.8***	1.01	.984**	1.002	.788***	1.012	.823	.409	N=776,330
	[.791, .81]	[.991, 1.03]	[.971, .9979]	[.976, 1.028]	[.778, .797]	[.99, 1.034]	[.816, .83]	[.39, .427]	
Armenia	.788***	1.061	1.053*	1.024	.83***	1.087*	.839	.86	N=3,171
(2010)	[.757, .821]	[.986, 1.142]	[.9988, 1.11]	[.914, 1.148]	[.807, .854]	[.988, 1.196]	[.818, .86]	[.838, .882]	
Colombia	.787***	.9992	1.043***	1.03*	.821***	1.029**	.831	.621	N=29,463
(2015–16)	[.776, .799]	[.974, 1.025]	[1.021, 1.065]	[.9958, 1.065]	[.81, .832]	[1.004, 1.054]	[.823, .838]	[.602, .64]	
Egypt	.743***	1.002	.949***	1.002	.705***	1.005	.766	.825	N=19,382
(2008)	[.731, .754]	[.968, 1.038]	[.929, .97]	[.957, 1.049]	[.692, .718]	[.968, 1.043]	[.756, .777]	[.811, .839]	
Gabon	.89***	.972	1.001	1.033	.891***	1.004	.902	.0722	N=8,462

	Constant	Washer	Female	Interaction	Constant× Female	Interaction× Washer	Mean school attendance	Mean washer ownership	
(2012)	[.875, .906]	[.905, 1.043]	[.977, 1.025]	[.952, 1.122]	[.878, .905]	[.949, 1.062]	[.889, .914]	[.0508, .0936]	
Guatemala	.666***	.986	.872***	.99	.581***	.976	.684	.113	N=24,453
(2014–15)	[.652, .68]	[.939, 1.035]	[.848, .896]	[.925, 1.059]	[.566, .595]	[.926, 1.029]	[.671, .696]	[.099, .127]	
Guyana	.659***	1.014	.98	.973	.646***	.987	.749	.214	N=4,558
(2009)	[.628, .691]	[.953, 1.079]	[.921, 1.044]	[.88, 1.077]	[.618, .675]	[.911, 1.069]	[.729, .77]	[.186, .243]	
India	.716***	.992	.948***	.9966	.679***	.989*	.759	.109	N=558,460
(2015–16)	[.713, .719]	[.981, 1.003]	[.943, .953]	[.983, 1.011]	[.676, .682]	[.978, 1]	[.757, .762]	[.106, .113]	
Kyrgyz Republic	.881***	.982	1.025**	1.011	.903***	.993	.91	.638	N=6,551
(2012)	[.866, .897]	[.952, 1.012]	[1.004, 1.046]	[.977, 1.047]	[.89, .917]	[.965, 1.021]	[.897, .923]	[.606, .671]	
Pakistan	.571***	.971	.692***	1.063	.395***	1.031	.564	.492	N=22,109
(2012–13)	[.554, .588]	[.912, 1.033]	[.66, .724]	[.965, 1.17]	[.377, .413]	[.945, 1.125]	[.54, .588]	[.45, .533]	
Peru	.76***	1.002	.9975	1.006	.758***	1.008	.796	.174	N=82,853
(2009,2010,2011,2012)	[.754, .767]	[.977, 1.027]	[.987, 1.008]	[.975, 1.038]	[.751, .765]	[.984, 1.032]	[.791, .801]	[.164, .184]	
Tajikistan	.85***	1.007	.842***	.977	.716***	.984	.829	.168	N=8,250
(2012)	[.834, .868]	[.969, 1.045]	[.812, .873]	[.926, 1.032]	[.696, .737]	[.942, 1.028]	[.814, .843]	[.145, .19]	
Türkiye	.718***	1.019	.883***	1.275***	.634***	1.299***	.732	.903	N=8,618
(2008)	[.697, .739]	[.925, 1.122]	[.847, .921]	[1.075, 1.511]	[.614, .655]	[1.119, 1.507]	[.715, .748]	[.886, .921]	
**Latest surveys (~2013–21)**									
Pooled	.854***	1.021**	.992	.9952	.847***	1.016	.869	.626	N=658,263
	[.847, .861]	[1.004, 1.039]	[.981, 1.004]	[.972, 1.019]	[.84, .855]	[.9965, 1.037]	[.864, .874]	[.607, .645]	
Albania	.823***	1	1.042***	1.024	.858***	1.024	.864	.925	N=7,377
(2017–18)	[.804, .843]	[.926, 1.081]	[1.011, 1.075]	[.931, 1.127]	[.841, .875]	[.944, 1.112]	[.85, .878]	[.913, .938]	
Armenia	.832***	1.05	1.067***	.938	.887***	.985	.886	.952	N=3,231
(2015–16)	[.811, .854]	[.984, 1.121]	[1.031, 1.104]	[.826, 1.066]	[.87, .905]	[.886, 1.096]	[.873, .899]	[.94, .964]	
Egypt	.819***	1.011	.966***	.988	.791***	.9994	.832	.784	N=22,586
(2014)	[.808, .83]	[.984, 1.039]	[.95, .983]	[.958, 1.02]	[.779, .803]	[.974, 1.026]	[.822, .841]	[.771, .798]	
India	.788***	.99**	.964***	1.003	.76***	.994	.803	.151	N=517,508
(2019–21)	[.785, .791]	[.982, .9991]	[.96, .968]	[.993, 1.014]	[.757, .763]	[.985, 1.003]	[.8, .805]	[.148, .155]	
Indonesia	.771***	.991	1.03***	1.006	.794***	.9971	.822	.35	N=35,687
(2017)	[.762, .78]	[.971, 1.011]	[1.014, 1.047]	[.981, 1.032]	[.785, .804]	[.979, 1.016]	[.816, .829]	[.335, .365]	
Pakistan	.646***	.983	.742***	.9985	.479***	.981	.629	.561	N=23,122
(2017–18)	[.627, .665]	[.93, 1.038]	[.711, .774]	[.927, 1.076]	[.459, .501]	[.927, 1.039]	[.606, .653]	[.525, .597]	
Philippines	.828***	.9994	1.063***	.995	.879***	.994	.869	.403	N=25,356
(2017)	[.817, .839]	[.972, 1.027]	[1.043, 1.082]	[.959, 1.031]	[.869, .89]	[.968, 1.021]	[.861, .878]	[.372, .433]	
South Africa	.912***	1.025	.977**	1.01	.891***	1.036*	.912	.375	N=7,000
(2016)	[.899, .924]	[.99, 1.061]	[.958, .9968]	[.965, 1.058]	[.878, .904]	[.9985, 1.074]	[.903, .92]	[.341, .409]	
Tajikistan	.886***	.9982	.894***	.991	.792***	.989	.868	.352	N=8,294
(2017)	[.874, .897]	[.971, 1.026]	[.87, .918]	[.944, 1.039]	[.773, .811]	[.952, 1.027]	[.857, .879]	[.316, .389]	
Türkiye	.794***	1.017	.958***	.965	.761***	.981	.812	.959	N=8,102

	Constant	Washer	Female	Interaction	Constant× Female	Interaction× Washer	Mean school attendance	Mean washer ownership
(2013)	[.778, .811]	[.904, 1.144]	[.929, .987]	[.815, 1.143]	[.742, .78]	[.822, 1.172]	[.797, .827]	[.947, .971]

Notes: *P<0.1; **P<0.05; ***P<0.01. Rate ratios and a constant from Poisson regression models are shown, in addition to two combinations obtained using post estimation. Surveys were divided into the periods indicated at the top of each panel: in some countries other periodization were used (shown in parentheses below country name). Each row of estimates was obtained from a separate model. All models included a baseline term for being female and baseline terms and interactions with being female for washer ownership, fridge ownership, TV ownership, having flush toilet, a wealth index z-score, number of household members, number of household members under age five, age, highest education level of a male in household, and highest education level of a female in household, as well as adjusting for neighborhood. Except the baseline term for female, all independent variables, including fixed effects, were centered around a country-specific weighted mean: Therefore, the 'Constant' column shows school attendance for males with the mean on all other independent variables. The column labelled 'Washer' shows differences in school attendance for males with washer at home. The column labelled 'Interaction' shows interaction terms for females and washer. The column labelled 'Constant×Female' shows school attendance for females with the mean on all other independent variables, obtained using post estimation. The column labelled 'Interaction×Washer' shows difference in school attendance for girls with washer at home, obtained using post estimation. Means for school attendance and washer ownership are also shown. Estimates were weighted using sampling weights, rescaled to sum up to one for the final sample from each survey. 95% confidence intervals adjusted for clustering at the level of primary sampling units are shown in brackets below the point estimates. Pooled models were further rescaled such that each country contributed equally to the estimates.

Table S9. Results from Poisson regression models of school attendance on washer ownership: stratified by household wealth

	Constant	Washer	Female	Interaction	Constant× Female	Interaction× Washer	Mean school attendance	Mean washer ownership	
**Poorest third**									
Pooled	.739***	1.02	.964***	1.013	.712***	1.033**	.769	.292	N=580,948
	[.729, .749]	[.9952, 1.046]	[.947, .98]	[.978, 1.049]	[.702, .722]	[1.003, 1.064]	[.762, .777]	[.276, .307]	
Albania	.719***	1.026	.955*	1.086**	.687***	1.114***	.761	.639	N=5,381
	[.694, .745]	[.97, 1.085]	[.908, 1.005]	[1.007, 1.172]	[.661, .715]	[1.038, 1.196]	[.743, .779]	[.602, .676]	
Armenia	.667***	1.02	1.135***	1.032	.757***	1.053	.788	.74	N=3,516
	[.634, .7]	[.967, 1.075]	[1.064, 1.212]	[.951, 1.121]	[.732, .783]	[.98, 1.132]	[.77, .805]	[.709, .77]	
Azerbaijan	.835***	1.13**	.881***	.822	.736***	.928	.83	.0291	N=2,332
	[.81, .861]	[1.006, 1.269]	[.832, .933]	[.642, 1.051]	[.704, .769]	[.735, 1.172]	[.81, .849]	[.0153, .0429]	
Colombia	.647***	.974	1.069***	1.017	.691***	.991	.726	.168	N=25,420
	[.632, .662]	[.935, 1.014]	[1.04, 1.099]	[.969, 1.068]	[.677, .706]	[.952, 1.03]	[.716, .737]	[.155, .181]	
Egypt	.662***	1.045**	.796***	1.099***	.527***	1.148***	.653	.751	N=36,233
	[.652, .672]	[1.009, 1.082]	[.777, .815]	[1.045, 1.157]	[.515, .539]	[1.098, 1.202]	[.644, .662]	[.739, .764]	
Gabon	.866***		.955**		.826***	NA	.866	0	N=4,812
	[.845, .887]		[.92, .991]		[.802, .851]		[.849, .883]	[., .]	
Guatemala	.525***		.747***		.392***	NA	.568	0	N=8,224
	[.499, .552]		[.696, .802]		[.363, .423]		[.55, .586]	[., .]	
Guyana	.616***	1.091	1.078	.697**	.664***	.761**	.747	.00587	N=2,652
	[.568, .667]	[.924, 1.288]	[.977, 1.19]	[.513, .949]	[.623, .707]	[.585, .989]	[.717, .776]	[-.00162, .0134]	
India	.664***	1.021	.936***	.972	.621***	.993	.701	.00111	N=379,569
	[.66, .667]	[.931, 1.12]	[.929, .943]	[.876, 1.079]	[.617, .625]	[.902, 1.093]	[.698, .704]	[.000919, .0013]	
Indonesia	.708***	.976	1.024	1.01	.725***	.987	.77	.0438	N=14,117
	[.69, .726]	[.91, 1.048]	[.992, 1.057]	[.924, 1.105]	[.706, .743]	[.929, 1.048]	[.757, .784]	[.0367, .0508]	
Kyrgyz Republic	.896***	.948**	1.005	1.046*	.9***	.991	.918	.625	N=2,315
	[.876, .915]	[.909, .989]	[.974, 1.038]	[.9951, 1.098]	[.88, .921]	[.958, 1.025]	[.902, .934]	[.573, .677]	
Moldova	.693***	1.042	1.156***	.966	.801***	1.007	.801	.334	N=1,416
	[.655, .732]	[.958, 1.134]	[1.08, 1.237]	[.864, 1.08]	[.769, .833]	[.936, 1.083]	[.776, .826]	[.296, .372]	
Morocco	.355***	.023***	.484***	2.689***	.172***	.0618***	.386	.000727	N=5,325
	[.326, .387]	[.0032, .165]	[.416, .564]	[2.153, 3.358]	[.149, .199]	[.00863, .443]	[.362, .41]	[-.000406, .00186]	
Pakistan	.382***	.988	.421***	1.091	.161***	1.078	.346	.0632	N=23,352
	[.364, .401]	[.889, 1.099]	[.388, .457]	[.946, 1.258]	[.148, .175]	[.945, 1.23]	[.328, .364]	[.0539, .0725]	
Peru	.75***	.764	.924***	1.561**	.693***	1.192***	.775	.000452	N=34,038
	[.74, .761]	[.54, 1.08]	[.908, .941]	[1.097, 2.221]	[.681, .706]	[1.115, 1.274]	[.766, .784]	[-.000155, .00106]	
Philippines	.675***	.97	1.116***	1.001	.753***	.971	.752	.0181	N=15,646
	[.659, .691]	[.889, 1.057]	[1.085, 1.148]	[.905, 1.108]	[.737, .769]	[.91, 1.036]	[.74, .763]	[.0135, .0228]	
South Africa	.903***	.963	.96**	1.014	.867***	.976	.898	.0438	N=2,379

	Constant	Washer	Female	Interaction	Constant× Female	Interaction× Washer	Mean school attendance	Mean washer ownership	
	[.881, .925]	[.861, 1.077]	[.927, .994]	[.901, 1.141]	[.844, .891]	[.911, 1.046]	[.882, .913]	[.0297, .0578]	
Tajikistan	.866***	.992	.844***	.938	.731***	.93	.842	.0268	N=4,672
	[.85, .882]	[.922, 1.067]	[.808, .882]	[.827, 1.064]	[.703, .76]	[.837, 1.034]	[.825, .859]	[.0178, .0359]	
Türkiye	.637***	.944*	.783***	1.294***	.499***	1.222***	.657	.713	N=9,549
	[.616, .66]	[.889, 1.003]	[.745, .822]	[1.188, 1.41]	[.479, .52]	[1.119, 1.334]	[.641, .672]	[.684, .743]	
**Middle third**									
Pooled	.784***	1.016	1.007	.995	.79***	1.011	.817	.441	N=540,866
	[.775, .794]	[.992, 1.04]	[.992, 1.022]	[.965, 1.026]	[.781, .799]	[.985, 1.037]	[.811, .824]	[.425, .457]	
Albania	.798***	1.018	1.013	1.027	.808***	1.046	.833	.926	N=4,188
	[.773, .824]	[.907, 1.144]	[.969, 1.058]	[.893, 1.181]	[.785, .832]	[.935, 1.17]	[.817, .85]	[.913, .94]	
Armenia	.76***	1.018	1.137***	1.008	.864***	1.026	.849	.841	N=4,046
	[.731, .791]	[.936, 1.107]	[1.086, 1.19]	[.903, 1.125]	[.846, .883]	[.948, 1.111]	[.833, .865]	[.817, .865]	
Azerbaijan	.86***	1.006	.879***	1.001	.756***	1.006	.845	.136	N=2,208
	[.828, .893]	[.93, 1.087]	[.83, .932]	[.905, 1.106]	[.722, .791]	[.899, 1.127]	[.821, .869]	[.0991, .173]	
Colombia	.753***	.975*	1.022*	1.018	.769***	.993	.8	.347	N=20,534
	[.74, .766]	[.947, 1.003]	[.9987, 1.045]	[.983, 1.055]	[.757, .782]	[.963, 1.022]	[.792, .808]	[.331, .363]	
Egypt	.745***	1.03	.928***	.972	.692***	1.001	.762	.945	N=31,733
	[.736, .755]	[.987, 1.075]	[.911, .946]	[.921, 1.025]	[.681, .703]	[.957, 1.048]	[.755, .769]	[.94, .95]	
Gabon	.87***	1.221***	1.034	.828*	.9***	1.011	.897	.00409	N=2,073
	[.839, .903]	[1.11, 1.343]	[.988, 1.081]	[.677, 1.013]	[.877, .923]	[.845, 1.21]	[.873, .921]	[-.00104, .00923]	
Guatemala	.627***	1.045	.843***	1.06	.529***	1.108	.653	.014	N=8,303
	[.604, .651]	[.883, 1.236]	[.798, .89]	[.766, 1.468]	[.506, .553]	[.816, 1.503]	[.636, .67]	[.00941, .0186]	
Guyana	.658***	1.033	1.006	.89	.662***	.92	.751	.0846	N=1,988
	[.617, .702]	[.928, 1.151]	[.925, 1.095]	[.739, 1.072]	[.626, .7]	[.78, 1.085]	[.722, .779]	[.0566, .113]	
India	.757***	.976*	.95***	1.019	.719***	.995	.781	.0179	N=367,292
	[.753, .76]	[.952, 1]	[.945, .956]	[.989, 1.05]	[.716, .723]	[.969, 1.022]	[.778, .783]	[.017, .0188]	
Indonesia	.752***	1.003	1.039***	.985	.781***	.987	.817	.26	N=10,844
	[.737, .768]	[.968, 1.038]	[1.009, 1.069]	[.945, 1.026]	[.765, .798]	[.955, 1.02]	[.807, .826]	[.243, .277]	
Kyrgyz Republic	.85***	.9959	1.035*	.9962	.879***	.992	.893	.543	N=2,176
	[.826, .874]	[.941, 1.054]	[.9963, 1.075]	[.928, 1.07]	[.857, .902]	[.94, 1.047]	[.873, .912]	[.498, .588]	
Moldova	.832***	1.05	1.086***	.956	.904***	1.004	.892	.761	N=1,682
	[.803, .862]	[.978, 1.127]	[1.041, 1.134]	[.88, 1.038]	[.882, .926]	[.95, 1.061]	[.874, .91]	[.729, .793]	
Morocco	.584***	.944	.857***	1.132	.501***	1.069	.615	.0129	N=4,577
	[.555, .615]	[.507, 1.758]	[.804, .915]	[.664, 1.928]	[.473, .53]	[.797, 1.433]	[.59, .641]	[.00642, .0194]	
Pakistan	.515***	.994	.726***	1.005	.374***	.9981	.52	.5	N=22,798
	[.499, .532]	[.947, 1.043]	[.696, .758]	[.944, 1.069]	[.358, .39]	[.944, 1.056]	[.504, .535]	[.48, .52]	
Peru	.732***	1.054*	1.006	.949	.736***	1	.78	.0354	N=28,201
	[.72, .744]	[.991, 1.12]	[.985, 1.026]	[.875, 1.03]	[.725, .748]	[.934, 1.071]	[.772, .788]	[.0305, .0403]	
Philippines	.76***	1.013	1.087***	.979	.826***	.992	.821	.229	N=12,650

	Constant	Washer	Female	Interaction	Constant× Female	Interaction× Washer	Mean school attendance	Mean washer ownership	
	[.744, .777]	[.975, 1.052]	[1.059, 1.117]	[.938, 1.023]	[.812, .84]	[.958, 1.027]	[.811, .83]	[.212, .246]	
South Africa	.894***	.9993	1.005	1.027	.898***	1.026	.908	.298	N=2,448
	[.874, .914]	[.944, 1.057]	[.973, 1.037]	[.963, 1.096]	[.875, .922]	[.972, 1.084]	[.891, .925]	[.258, .339]	
Tajikistan	.851***	1.01	.855***	1.005	.727***	1.015	.836	.183	N=4,808
	[.833, .869]	[.966, 1.056]	[.822, .888]	[.948, 1.066]	[.705, .751]	[.965, 1.067]	[.822, .85]	[.158, .208]	
Türkiye	.727***	.943	.902***	1.385***	.656***	1.307***	.745	.951	N=8,317
	[.708, .746]	[.829, 1.073]	[.867, .938]	[1.141, 1.682]	[.635, .677]	[1.109, 1.54]	[.731, .758]	[.942, .96]	
**Wealthiest third**									
Pooled	.871***	.9953	1.004	1.013	.874***	1.009	.885	.688	N=473,818
	[.864, .878]	[.977, 1.014]	[.993, 1.014]	[.991, 1.036]	[.867, .881]	[.99, 1.028]	[.88, .891]	[.673, .703]	
Albania	.889***	.913	1.034**	1.043	.919***	.952	.916	.993	N=3,911
	[.87, .908]	[.785, 1.061]	[1.003, 1.065]	[.853, 1.275]	[.9, .938]	[.799, 1.134]	[.902, .93]	[.989, .9969]	
Armenia	.885***	.931	1.048***	1.021	.927***	.95	.915	.957	N=3,123
	[.864, .906]	[.84, 1.031]	[1.016, 1.08]	[.908, 1.148]	[.91, .944]	[.876, 1.03]	[.902, .929]	[.945, .968]	
Azerbaijan	.929***	1.01	.993	1.032	.923***	1.043	.94	.452	N=1,623
	[.911, .948]	[.972, 1.05]	[.962, 1.024]	[.972, 1.097]	[.906, .939]	[.983, 1.107]	[.925, .954]	[.394, .511]	
Colombia	.853***	1.012	1.018	1.031	.868***	1.044**	.875	.777	N=13,228
	[.84, .867]	[.973, 1.053]	[.993, 1.043]	[.983, 1.082]	[.856, .881]	[1.004, 1.086]	[.866, .883]	[.759, .794]	
Egypt	.87***	.996	.987**	.98**	.859***	.976***	.88	.728	N=33,081
	[.862, .877]	[.981, 1.011]	[.976, .9986]	[.962, .9988]	[.851, .866]	[.961, .992]	[.874, .885]	[.716, .741]	
Gabon	.927***	.946	1.018	1.088	.943***	1.03	.944	.213	N=1,534
	[.906, .948]	[.856, 1.046]	[.985, 1.052]	[.982, 1.206]	[.928, .959]	[.977, 1.085]	[.927, .96]	[.159, .267]	
Guatemala	.821***	1.006	.96***	.98	.788***	.986	.83	.325	N=7,801
	[.804, .838]	[.964, 1.051]	[.932, .988]	[.924, 1.04]	[.771, .804]	[.942, 1.033]	[.819, .841]	[.297, .354]	
Guyana	.768***	1.019	.993	1.026	.762***	1.045	.818	.445	N=2,061
	[.734, .804]	[.95, 1.093]	[.934, 1.055]	[.944, 1.115]	[.73, .795]	[.978, 1.117]	[.797, .84]	[.406, .483]	
India	.847***	.988***	.983***	1	.832***	.988***	.864	.375	N=317,276
	[.844, .85]	[.981, .9958]	[.978, .988]	[.991, 1.009]	[.829, .836]	[.981, .996]	[.862, .866]	[.37, .38]	
Indonesia	.849***	.9986	1.017	.99	.864***	.989	.879	.747	N=10,239
	[.836, .863]	[.968, 1.03]	[.994, 1.041]	[.951, 1.03]	[.851, .877]	[.96, 1.018]	[.871, .887]	[.732, .763]	
Kyrgyz Republic	.89***	1.001	1.028*	.985	.916***	.986	.918	.746	N=1,999
	[.867, .915]	[.944, 1.061]	[.9977, 1.06]	[.917, 1.058]	[.896, .936]	[.921, 1.054]	[.897, .94]	[.708, .784]	
Moldova	.905***	.99	1.046***	1.083*	.946***	1.072*	.933	.863	N=2,278
	[.886, .925]	[.924, 1.06]	[1.018, 1.073]	[.993, 1.181]	[.934, .959]	[1, 1.149]	[.92, .946]	[.839, .888]	
Morocco	.81***	1.009	.955***	.985	.774***	.994	.813	.402	N=4,076
	[.791, .83]	[.951, 1.071]	[.922, .989]	[.908, 1.068]	[.754, .794]	[.934, 1.057]	[.795, .83]	[.358, .445]	
Pakistan	.655***	.963	.937***	1.059	.614***	1.02	.681	.934	N=24,495
	[.639, .671]	[.887, 1.045]	[.91, .965]	[.949, 1.183]	[.597, .631]	[.92, 1.131]	[.666, .695]	[.927, .942]	
Peru	.799***	.991	1.032***	1.017	.824***	1.008	.834	.488	N=19,764

	Constant	Washer	Female	Interaction	Constant× Female	Interaction× Washer	Mean school attendance	Mean washer ownership	
	[.788, .81]	[.961, 1.021]	[1.014, 1.051]	[.98, 1.055]	[.813, .835]	[.981, 1.035]	[.827, .842]	[.471, .505]	
Philippines	.871***	.989	.984	1.017	.857***	1.007	.877	.8	N=10,519
	[.86, .883]	[.951, 1.029]	[.964, 1.004]	[.968, 1.069]	[.844, .871]	[.968, 1.046]	[.866, .888]	[.779, .822]	
South Africa	.933***	1.079**	.977	.9964	.911***	1.075**	.931	.784	N=2,007
	[.915, .952]	[1.011, 1.152]	[.948, 1.007]	[.913, 1.088]	[.891, .932]	[1.009, 1.145]	[.917, .945]	[.743, .825]	
Tajikistan	.889***	.982	.896***	.992	.797***	.974	.868	.57	N=6,991
	[.876, .903]	[.952, 1.012]	[.873, .919]	[.956, 1.031]	[.778, .815]	[.943, 1.007]	[.856, .879]	[.542, .599]	
Türkiye	.805***	1.06	.912***	1.338***	.733***	1.419***	.811	.93	N=7,812
	[.789, .821]	[.961, 1.169]	[.883, .941]	[1.139, 1.573]	[.714, .753]	[1.203, 1.674]	[.797, .825]	[.915, .946]	

Notes: *P<0.1; **P<0.05; ***P<0.01. Rate ratios and a constant from Poisson regression models are shown, in addition to two combinations obtained using post estimation. Each row of estimates was obtained from a separate model. All models included a baseline term for being female and baseline terms and interactions with being female for washer ownership, fridge ownership, TV ownership, having flush toilet, a wealth index z-score, number of household members, number of household members under age five, age, highest education level of a male in household, and highest education level of a female in household, as well as adjusting for neighborhood. Except the baseline term for female, all independent variables, including fixed effects, were centered around a country-specific weighted mean: Therefore, the 'Constant' column shows school attendance for males with the mean on all other independent variables. The column labelled 'Washer' shows differences in school attendance for males with washer at home. The column labelled 'Interaction' shows interaction terms for females and washer. The column labelled 'Constant×Female' shows school attendance for females with the mean on all other independent variables, obtained using post estimation. The column labelled 'Interaction×Washer' shows difference in school attendance for girls with washer at home, obtained using post estimation. Means for school attendance and washer ownership are also shown. Estimates were weighted using sampling weights, rescaled to sum up to one for the final sample from each survey. 95% confidence intervals adjusted for clustering at the level of primary sampling units are shown in brackets below the point estimates. Pooled models were further rescaled such that each country contributed equally to the estimates.

Table S10. Results from Poisson regression models of school attendance on washer ownership: stratified by age

	Constant	Washer	Female	Interaction	Constant× Female	Interaction× Washer	Mean school attendance	Mean washer ownership	
**10–12 years**									
Pooled	.97***	1.009***	.991***	.9972	.961***	1.006*	.966	.459	N=670,916
	[.967, .972]	[1.003, 1.016]	[.987, .994]	[.989, 1.005]	[.958, .964]	[.9994, 1.014]	[.964, .968]	[.447, .472]	
Albania	.978***	.984	.9974	1.03	.975***	1.013	.978	.849	N=5,427
	[.971, .984]	[.958, 1.01]	[.987, 1.007]	[.993, 1.069]	[.967, .983]	[.982, 1.046]	[.973, .983]	[.828, .871]	
Armenia	.993***	1.006	1.001	.9955	.993***	1.001	.994	.852	N=4,213
	[.989, .997]	[.992, 1.021]	[.995, 1.006]	[.977, 1.014]	[.989, .9977]	[.987, 1.016]	[.991, .9966]	[.834, .869]	
Azerbaijan	.987***	1.014	.986*	1.024	.972***	1.039*	.98	.2	N=2,505
	[.978, .995]	[.994, 1.035]	[.971, 1]	[.987, 1.062]	[.96, .984]	[.997, 1.082]	[.972, .988]	[.165, .235]	
Colombia	.95***	.987*	1.018***	1.002	.967***	.988**	.959	.392	N=23,973
	[.944, .955]	[.973, 1.001]	[1.011, 1.026]	[.987, 1.016]	[.962, .972]	[.977, .9997]	[.955, .963]	[.374, .409]	
Egypt	.937***	1.021***	.953***	1.037***	.893***	1.058***	.921	.806	N=40,547
	[.933, .942]	[1.006, 1.036]	[.945, .96]	[1.015, 1.059]	[.886, .899]	[1.039, 1.078]	[.917, .926]	[.799, .813]	
Gabon	.983***	.9966	1.004	.976	.987***	.972	.986	.0692	N=3,957
	[.975, .992]	[.944, 1.052]	[.991, 1.017]	[.906, 1.051]	[.98, .995]	[.921, 1.026]	[.98, .991]	[.0514, .087]	
Guatemala	.928***	1.022	.956***	.967	.887***	.988	.912	.105	N=10,172
	[.918, .937]	[.987, 1.057]	[.941, .971]	[.92, 1.017]	[.875, .9]	[.95, 1.028]	[.903, .921]	[.0914, .119]	
Guyana	.974***	1.019	1.005	.974	.978***	.993	.977	.179	N=3,089
	[.962, .986]	[.9955, 1.044]	[.99, 1.02]	[.942, 1.007]	[.969, .987]	[.958, 1.028]	[.969, .986]	[.153, .205]	
India	.942***	1.006**	.995***	1.001	.937***	1.006**	.942	.125	N=449,586
	[.94, .943]	[1, 1.011]	[.993, .9971]	[.994, 1.008]	[.936, .939]	[1.001, 1.012]	[.941, .943]	[.122, .127]	
Indonesia	.975***	1.011*	1.003	.991	.977***	1.002	.977	.355	N=15,286
	[.97, .98]	[.999, 1.022]	[.996, 1.009]	[.977, 1.005]	[.972, .982]	[.993, 1.011]	[.973, .98]	[.339, .372]	
Kyrgyz Republic	.994***	.9985	1.001	1.007	.9953**	1.006	.995	.618	N=2,775
	[.99, .998]	[.989, 1.008]	[.995, 1.008]	[.995, 1.02]	[.991, .9998]	[.9983, 1.013]	[.991, .9981]	[.582, .653]	
Moldova	.974***	1.026*	1.02***	.972*	.993**	.9973	.985	.659	N=1,918
	[.962, .986]	[.9957, 1.057]	[1.006, 1.034]	[.944, 1.001]	[.987, .9988]	[.988, 1.007]	[.979, .992]	[.626, .692]	
Morocco	.846***	.975	.886***	.968	.75***	.943**	.818	.129	N=5,841
	[.83, .863]	[.926, 1.026]	[.86, .913]	[.906, 1.034]	[.727, .773]	[.898, .991]	[.798, .838]	[.107, .15]	
Pakistan	.67***	1.008	.801***	.9976	.537***	1.006	.659	.477	N=29,709
	[.656, .685]	[.971, 1.047]	[.78, .823]	[.944, 1.054]	[.521, .552]	[.959, 1.055]	[.643, .674]	[.457, .497]	
Peru	.966***	.9984	.9961*	.996	.963***	.994	.965	.154	N=35,061
	[.963, .97]	[.99, 1.007]	[.992, 1.001]	[.982, 1.011]	[.959, .967]	[.983, 1.006]	[.962, .968]	[.144, .165]	
Philippines	.941***	.998	1.024***	.987	.964***	.985*	.954	.322	N=16,979
	[.934, .948]	[.981, 1.016]	[1.015, 1.033]	[.967, 1.008]	[.958, .97]	[.968, 1.002]	[.948, .959]	[.299, .344]	
South Africa	.993***	1.009	.9985	1.007	.991***	1.016**	.992	.369	N=2,817

	Constant	Washer	Female	Interaction	Constant× Female	Interaction× Washer	Mean school attendance	Mean washer ownership	
	[.988, .9971]	[.9958, 1.022]	[.992, 1.006]	[.987, 1.027]	[.986, .9964]	[1.002, 1.03]	[.989, .9956]	[.33, .408]	
Tajikistan	.987***	1.009	.982***	.9984	.969***	1.008	.98	.272	N=6,635
	[.982, .992]	[.9967, 1.022]	[.971, .994]	[.983, 1.014]	[.959, .98]	[.9957, 1.02]	[.973, .986]	[.247, .298]	
Türkiye	.983***	.985	.949***	1.198***	.933***	1.18***	.962	.864	N=10,426
	[.979, .988]	[.959, 1.012]	[.939, .959]	[1.134, 1.265]	[.924, .942]	[1.117, 1.246]	[.957, .967]	[.85, .879]	
**13–15 years**									
Pooled	.9***	1.022**	.994	.988	.895***	1.01	.902	.461	N=310,074
	[.893, .908]	[1.004, 1.041]	[.984, 1.004]	[.964, 1.013]	[.888, .902]	[.991, 1.03]	[.896, .907]	[.447, .474]	
Albania	.905***	1.037	.96**	1.174**	.868***	1.217***	.897	.832	N=2,552
	[.884, .926]	[.931, 1.155]	[.924, .9969]	[1.011, 1.363]	[.843, .895]	[1.073, 1.381]	[.879, .914]	[.809, .856]	
Armenia	.937***	1.043	1.026*	.985	.962***	1.028	.953	.849	N=2,188
	[.918, .956]	[.97, 1.122]	[.9971, 1.056]	[.904, 1.074]	[.944, .98]	[.972, 1.087]	[.941, .966]	[.827, .87]	
Azerbaijan	.966***	1.016	.972*	1.011	.939***	1.027	.959	.205	N=1,396
	[.95, .982]	[.979, 1.055]	[.943, 1.002]	[.961, 1.062]	[.915, .964]	[.975, 1.082]	[.944, .974]	[.166, .243]	
Colombia	.847***	.974	1.047***	1.037	.887***	1.011	.875	.407	N=10,524
	[.833, .862]	[.938, 1.012]	[1.024, 1.072]	[.993, 1.084]	[.874, .901]	[.979, 1.043]	[.866, .885]	[.387, .426]	
Egypt	.837***	1.01	.92***	1.087***	.769***	1.098***	.822	.808	N=19,359
	[.827, .847]	[.977, 1.044]	[.903, .937]	[1.037, 1.138]	[.758, .781]	[1.058, 1.139]	[.814, .83]	[.799, .817]	
Gabon	.901***	1.032	1.062**	.904	.957***	.933	.939	.0669	N=1,600
	[.865, .939]	[.909, 1.172]	[1.006, 1.121]	[.769, 1.062]	[.936, .978]	[.831, 1.048]	[.922, .955]	[.0355, .0982]	
Guatemala	.703***	.983	.828***	1.011	.583***	.994	.678	.11	N=5,170
	[.677, .731]	[.906, 1.067]	[.783, .877]	[.905, 1.13]	[.555, .612]	[.914, 1.081]	[.655, .701]	[.0938, .126]	
Guyana	.836***	.967	1.075***	.937	.899***	.906*	.878	.161	N=1,393
	[.801, .874]	[.881, 1.061]	[1.021, 1.131]	[.812, 1.081]	[.87, .928]	[.816, 1.006]	[.854, .902]	[.133, .189]	
India	.85***	1.008	.985***	.993	.838***	1.001	.854	.127	N=205,235
	[.847, .854]	[.9968, 1.019]	[.98, .991]	[.979, 1.008]	[.834, .841]	[.989, 1.013]	[.852, .857]	[.123, .13]	
Indonesia	.898***	.999	1.034***	.98	.928***	.979	.917	.347	N=7,254
	[.884, .912]	[.967, 1.032]	[1.015, 1.054]	[.938, 1.023]	[.917, .94]	[.951, 1.008]	[.908, .927]	[.327, .367]	
Kyrgyz Republic	.988***	1.008	.994	1.002	.982***	1.01	.985	.626	N=1,385
	[.981, .9952]	[.986, 1.029]	[.98, 1.008]	[.965, 1.04]	[.97, .994]	[.979, 1.042]	[.978, .993]	[.586, .666]	
Moldova	.949***	1.083***	1.027	.931**	.975***	1.008	.964	.636	N=1,088
	[.923, .975]	[1.031, 1.138]	[.993, 1.063]	[.873, .992]	[.959, .991]	[.966, 1.051]	[.951, .978]	[.596, .676]	
Morocco	.608***	1.014	.743***	.849*	.452***	.861**	.606	.144	N=2,728
	[.577, .641]	[.908, 1.134]	[.675, .817]	[.715, 1.008]	[.413, .493]	[.757, .981]	[.573, .638]	[.119, .17]	
Pakistan	.56***	.966	.654***	1.052	.366***	1.016	.531	.494	N=14,296
	[.542, .578]	[.893, 1.045]	[.62, .689]	[.927, 1.193]	[.348, .386]	[.924, 1.117]	[.514, .548]	[.473, .516]	
Peru	.898***	.975	.99	1.034	.889***	1.008	.898	.162	N=17,111
	[.888, .907]	[.941, 1.01]	[.977, 1.004]	[.985, 1.086]	[.88, .898]	[.973, 1.044]	[.891, .905]	[.15, .175]	
Philippines	.815***	1.028	1.096***	.98	.894***	1.007	.862	.343	N=7,739

	Constant	Washer	Female	Interaction	Constant× Female	Interaction× Washer	Mean school attendance	Mean washer ownership	
	[.798, .833]	[.98, 1.077]	[1.068, 1.125]	[.924, 1.039]	[.881, .907]	[.964, 1.052]	[.85, .873]	[.32, .367]	
South Africa	.968***	1.041*	1.014	.989	.981**	1.03*	.976	.379	N=1,137
	[.95, .987]	[.993, 1.092]	[.986, 1.042]	[.934, 1.047]	[.967, .9959]	[.9951, 1.066]	[.965, .987]	[.327, .43]	
Tajikistan	.99**	1	.955***	.977	.945***	.977*	.971	.248	N=3,225
	[.981, .9988]	[.983, 1.018]	[.939, .972]	[.944, 1.01]	[.93, .961]	[.95, 1.004]	[.962, .98]	[.222, .274]	
Türkiye	.832***	.945	.801***	1.57***	.667***	1.483***	.783	.86	N=4,694
	[.812, .853]	[.848, 1.052]	[.763, .841]	[1.267, 1.946]	[.64, .695]	[1.212, 1.814]	[.765, .8]	[.842, .877]	
**16–19 years**									
Pooled	.596***	1.047**	.9965	.9978	.594***	1.045**	.628	.488	N=611,776
	[.586, .607]	[1.01, 1.085]	[.976, 1.017]	[.952, 1.046]	[.584, .604]	[1.007, 1.084]	[.62, .637]	[.476, .5]	
Albania	.604***	1.062	1.003	1.061	.606***	1.127	.66	.858	N=5,312
	[.576, .633]	[.899, 1.255]	[.939, 1.071]	[.843, 1.335]	[.578, .634]	[.943, 1.347]	[.636, .683]	[.839, .877]	
Armenia	.53***	1	1.243***	1.147	.659***	1.147**	.65	.836	N=4,173
	[.502, .56]	[.86, 1.163]	[1.159, 1.335]	[.953, 1.379]	[.636, .684]	[1.004, 1.31]	[.627, .672]	[.817, .855]	
Azerbaijan	.716***	1.078	.833***	.977	.597***	1.053	.708	.212	N=2,277
	[.679, .755]	[.964, 1.205]	[.765, .907]	[.824, 1.159]	[.564, .631]	[.908, 1.222]	[.676, .74]	[.181, .243]	
Colombia	.526***	.96	1.055**	1.092**	.555***	1.049	.582	.445	N=21,282
	[.511, .543]	[.899, 1.026]	[1.012, 1.1]	[1.004, 1.189]	[.541, .57]	[.987, 1.115]	[.569, .595]	[.424, .466]	
Egypt	.569***	.9999	.825***	.9951	.469***	.995	.573	.815	N=40,277
	[.559, .578]	[.965, 1.036]	[.805, .846]	[.949, 1.044]	[.459, .479]	[.958, 1.034]	[.565, .581]	[.808, .822]	
Gabon	.756***	.914	.988	1.147	.747***	1.049	.777	.0797	N=2,844
	[.719, .794]	[.752, 1.111]	[.922, 1.058]	[.923, 1.425]	[.713, .782]	[.911, 1.207]	[.748, .806]	[.0471, .112]	
Guatemala	.404***	.99	.752***	1.054	.304***	1.044	.429	.122	N=9,083
	[.383, .426]	[.89, 1.102]	[.694, .815]	[.9, 1.233]	[.284, .325]	[.925, 1.177]	[.409, .449]	[.106, .139]	
Guyana	.345***	.899	1.015	1.248	.35***	1.122	.431	.187	N=2,203
	[.306, .389]	[.702, 1.152]	[.871, 1.184]	[.9, 1.729]	[.313, .392]	[.899, 1.4]	[.4, .462]	[.162, .212]	
India	.534***	.981**	.892***	1.016	.476***	.9972	.57	.135	N=411,124
	[.531, .538]	[.965, .9975]	[.883, .9]	[.995, 1.038]	[.473, .48]	[.98, 1.014]	[.567, .573]	[.132, .138]	
Indonesia	.512***	.96	1.089***	1.019	.557***	.977	.59	.346	N=12,889
	[.494, .53]	[.898, 1.025]	[1.039, 1.141]	[.936, 1.109]	[.54, .576]	[.917, 1.042]	[.577, .604]	[.329, .363]	
Kyrgyz Republic	.712***	.966	1.069**	.971	.762***	.937	.776	.663	N=2,342
	[.678, .748]	[.876, 1.064]	[1.013, 1.129]	[.867, 1.086]	[.732, .793]	[.86, 1.021]	[.744, .807]	[.625, .7]	
Moldova	.621***	1.082	1.209***	.9981	.75***	1.08	.73	.65	N=2,336
	[.588, .655]	[.949, 1.234]	[1.132, 1.291]	[.851, 1.171]	[.72, .782]	[.977, 1.194]	[.705, .755]	[.622, .679]	
Morocco	.326***	1.058	.59***	1.02	.192***	1.079	.377	.145	N=5,431
	[.302, .352]	[.909, 1.231]	[.515, .676]	[.832, 1.251]	[.17, .217]	[.926, 1.258]	[.349, .405]	[.122, .169]	
Pakistan	.345***	.979	.552***	1.074	.19***	1.052	.348	.526	N=26,765
	[.332, .358]	[.897, 1.07]	[.517, .589]	[.946, 1.22]	[.179, .202]	[.95, 1.165]	[.334, .361]	[.505, .546]	
Peru	.524***	1.073*	.978	1.009	.513***	1.082**	.549	.19	N=29,686

	Constant	Washer	Female	Interaction	Constant× Female	Interaction× Washer	Mean school attendance	Mean washer ownership	
	[.513, .535]	[.988, 1.165]	[.951, 1.007]	[.91, 1.118]	[.501, .524]	[1.006, 1.164]	[.54, .558]	[.178, .202]	
Philippines	.564***	.958	1.087***	1.091*	.613***	1.045	.624	.377	N=14,138
	[.548, .581]	[.894, 1.027]	[1.04, 1.136]	[.989, 1.203]	[.594, .633]	[.974, 1.122]	[.61, .638]	[.357, .397]	
South Africa	.794***	1.07	.962	.971	.763***	1.039	.799	.361	N=2,701
	[.765, .824]	[.982, 1.166]	[.914, 1.011]	[.851, 1.108]	[.736, .791]	[.935, 1.155]	[.78, .818]	[.325, .398]	
Tajikistan	.707***	1.022	.715***	.974	.505***	.9951	.658	.252	N=6,610
	[.685, .728]	[.963, 1.085]	[.675, .757]	[.876, 1.082]	[.48, .531]	[.914, 1.083]	[.641, .676]	[.229, .276]	
Türkiye	.468***	1.105	.766***	1.232	.359***	1.361**	.485	.863	N=10,303
	[.45, .488]	[.945, 1.293]	[.718, .817]	[.935, 1.621]	[.339, .379]	[1.06, 1.748]	[.468, .502]	[.849, .878]	

Notes: *P<0.1; **P<0.05; ***P<0.01. Rate ratios and a constant from Poisson regression models are shown, in addition to two combinations obtained using post estimation. Each row of estimates was obtained from a separate model. All models included a baseline term for being female and baseline terms and interactions with being female for washer ownership, fridge ownership, TV ownership, having flush toilet, a wealth index z-score, number of household members, number of household members under age five, age, highest education level of a male in household, and highest education level of a female in household, as well as adjusting for neighborhood. Except the baseline term for female, all independent variables, including fixed effects, were centered around a country-specific weighted mean: Therefore, the 'Constant' column shows school attendance for males with the mean on all other independent variables. The column labelled 'Washer' shows differences in school attendance for males with washer at home. The column labelled 'Interaction' shows interaction terms for females and washer. The column labelled 'Constant×Female' shows school attendance for females with the mean on all other independent variables, obtained using post estimation. The column labelled 'Interaction×Washer' shows difference in school attendance for girls with washer at home, obtained using post estimation. Means for school attendance and washer ownership are also shown. Estimates were weighted using sampling weights, rescaled to sum up to one for the final sample from each survey. 95% confidence intervals adjusted for clustering at the level of primary sampling units are shown in brackets below the point estimates. Pooled models were further rescaled such that each country contributed equally to the estimates.

SUPPLEMENT 4: Supplementary and sensitivity analyses

Appendix S1. Supplementary and sensitivity analyses

Seventeen supplementary and sensitivity analyses were done. The relationship between washer ownership and school attendance was compared to that of other household assets and amenities, some of which may also be time saving to a varying extent, while others are more likely to only reflect socioeconomic status: 1) refrigerator (Supplementary Figure S3 and Table S11), 2) piped (drinking) water (Supplementary Figure S4 and Table S12), 3) electricity (Supplementary Figure S5 and Table S13), 4) car (Supplementary Figure S6 and Table S14), 5) motorbike (Supplementary Figure S7 and Table S15), 6) clean cooking fuel (Supplementary Figure S8 and Table S16), 7) flush toilet (Supplementary Figure S9 and Table S17), and 8) TV (Supplementary Figure S10 and Table S18). Refrigerator ownership may involve timesaving within the household since it could reduce the burden from collecting and cooking food as food can be bought in bulk and stored. Having piped water for drinking can reduce the time needed to fetch water. Access to electricity may reduce the time burden of household tasks in general and extend work into the evenings due to improved lighting. Having a car or a motorbike may facilitate access to school or running errands for the household. Using clean cooking fuel reduces adverse exposures to dirty cooking fuels, improving health, and may reduce the time needed for cooking meals. Gathering wood for cooking fuel can also be time consuming. A flush toilet is similarly more hygienic, reducing adverse exposures, and is generally less time-consuming to maintain. The extent to which these items have an association with school attendance through reduced burden from work may be differential between boys and girls, depending on whether the work that is reduced is traditionally done by girls or boys.

It is less clear how having a TV can relate to child schooling other than by reflecting socioeconomic status. TV ownership should be unrelated to school attendance since it uses

time rather than saving time. However, TV ownership could plausibly relate to better information access, potentially improving attendance.

The association for having a flush toilet, TV ownership, and refrigerator ownership were obtained from the same models as the main results on washing machine ownership (since these variables were used as control variables in that analysis). The estimation of the association with the use of clean cooking fuel, access to piped water, car ownership, motorbike ownership, and access to electricity was estimated by adding these items to the models in place of washing machine ownership.

Other sensitivity checks involved using different estimation methods: 9) logit regression models providing odds ratios (Supplementary Figure S11 and Table S19) and 10) linear regression models (Supplementary Figure S12 and Table S20). 11) Since a large majority of households with a washing machine also had a TV and a refrigerator, and most also had a flush toilet, which may cause problems for the interpretation of the estimates for washer ownership, the sensitivity of the results was tested to the exclusion these control variables (Supplementary Figure S13 and Table S21). Further, since washer ownership is one of many variables used to construct the household wealth index, there may be concerns that controlling for the wealth index may control away some of the positive association between washer ownership and school attendance: therefore, 12) the sensitivity of the results was tested to the exclusion of the wealth index z-scores from the set of control variables in the models (Supplementary Figure S14 and Table S22).

The main results were estimated after making sample restrictions: 13) only descendants of the household head were included since others could potentially include spouses or staff (Supplementary Figure S15 and Table S23), 14) only households with access to electricity were included since electricity access is required for automatic washing machines (Supplementary Figure S16 and Table S24). 15) The results were also stratified

according to the overall share of adolescents attending school within sub-national regions, separated into urban and rural areas, generally overlapping with the sampling strata (Supplementary Figure S17 and Table S25). 16) we present results from linear regression models estimating the association of washer ownership with number of years of education the adolescent had completed in the year of the survey, overall (Supplementary Figure S18 and Table S26) and stratified by year of survey (Supplementary Figure S19 and Table S27). 17) The results were stratified according to the extent of female disadvantage in school attendance at the regional level (Supplementary Figure S20 and Table S28). The share of boys and girls in each sub-national region (further separated into urban and rural areas) was first estimated, and then the ratio of the attendance of boys to that of girls. The sample was divided into three categories: a) no female-disadvantage (a ratio equal to or below one), b) female-disadvantage (a ratio above one but below 1.05); and c) large female-disadvantage (a ratio equal to or above 1.05).

18) Results were obtained from a household fixed effect model, estimating the difference in the school attendance of boys and girls in households with and without a washer (Supplementary Figure S21 and Table S29). Only households with at least one girl and one boy were considered for these models. A household level weighted mean of all independent variables was used to adjust for household level factors. Of the independent variables used in this paper, only sex and age vary between children in the same households: therefore, the models included baseline terms only for being female and age, as well as interaction terms for female with all other independent variables (as well as age).

Results from supplementary and sensitivity analyses

The association for refrigerator ownership showed a fairly large and statistically significantly greater school attendance in some cases: for boys in Türkiye, Pakistan, and Morocco and girls in Egypt (Supplementary Figure S3 and Table S11). Having piped water

had a substantial and statistically significant positive association for girls in Guyana, Egypt, Morocco and Guatemala (Supplementary Figure S4 and Table S12). Electricity had a large statistically significant positive association with school attendance for girls in Pakistan (Supplementary Figure S5 and Table S13). The association between owning a car (Supplementary Figure S6 and Table S14) or scooter (Supplementary Figure S7 and Table S15) showed a small and mostly non-statistically significant association with school attendance. The association between living in households using clean cooking fuel was in most cases small and not statistically significant: Egypt was an exception, although the use of clean cooking fuel was almost universal there (Supplementary Figure S8 and Table S16). The association between having a flush toilet at home and school attendance was substantial and statistically significant and positive in many cases: for girls in Albania, Egypt, Pakistan, and Guatemala and boys in Guyana (Supplementary Figure S9 and Table S17). TV ownership was mostly small but statistically significant in a few cases (Supplementary Figure S10 and Table S18): However, in Pakistan, girls in households with a TV had a statistically significant 7% greater school attendance than girls that did not have a TV while boys with a TV had 4% lower school attendance than boys that did not. In Türkiye the association with TV ownership was substantial, negative, and statistically significant, both for boys and girls. The association was further positive and statistically significant for boys in Colombia and girls in Armenia.

Using logit models (Supplementary Figure S11 and Table S19) or linear regressions (Supplementary Figure S12 and Table S20) mostly yielded similar results, although a statistically significant association between washer ownership and school attendance was additionally observed for girls in Colombia. Excluding those without electricity removed the disadvantage observed for girls without a washer in the household in Morocco (as happened when stratifying by household wealth: Supplementary Figure S15 and Table S24). A positive association was additionally observed for girls in Pakistan and boys (in addition to girls) in

Albania, when using years of education as an outcome (Supplementary Figure S18 and Table S26).

Figure S3. Results from Poisson regression models of school attendance on fridge ownership

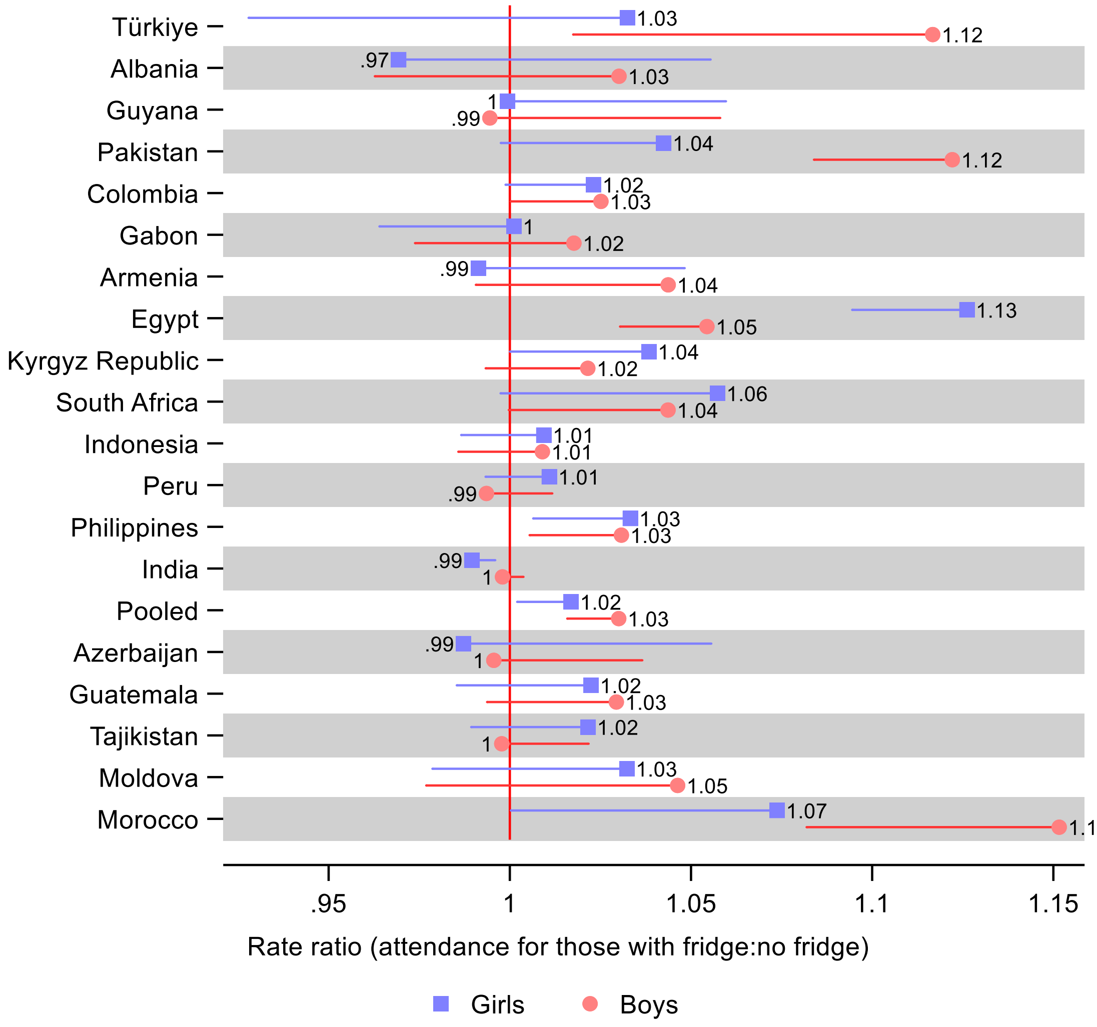

Notes: Countries were ordered according to the relative difference in rate ratio between boys and girls from Figure 1. 95% confidence intervals are shown. Estimates were weighted using sampling weights rescaled to sum up to one in each survey. Pooled models were further rescaled such that each country contributed equally to the estimates. All models included a baseline term for being female and baseline terms and interactions with being female for washer ownership, fridge ownership, TV ownership, having flush toilet, a wealth index z-score, number of household members, number of household members under age five, age, highest education level of a male in household, and highest education level of a female in household, as well as adjusting for neighborhood. Upper confidence limits were omitted for estimates above one and lower confidence limits were omitted for estimates below one, for improved readability. See Supplementary Table S11 for tabulated estimates.

Figure S4. Results from Poisson regression models of school attendance on piped water

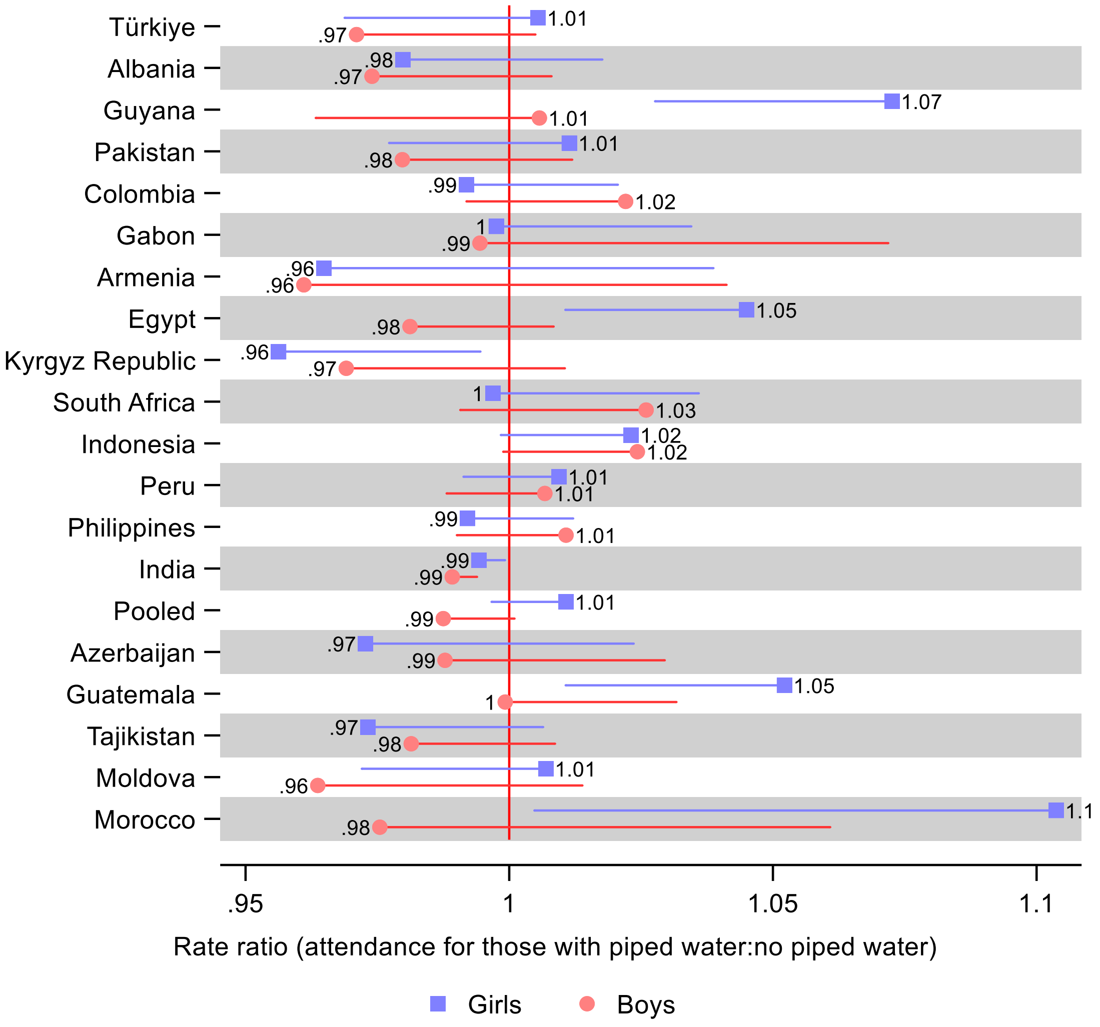

Notes: Countries were ordered according to the relative difference in rate ratio between boys and girls from Figure 1. 95% confidence intervals are shown. Estimates were weighted using sampling weights rescaled to sum up to one in each survey. All models included a baseline term for being female and baseline terms and interactions with being female for washer ownership, fridge ownership, TV ownership, having flush toilet, a wealth index z-score, number of household members, number of household members under age five, age, highest education level of a male in household, and highest education level of a female in household, as well as adjusting for neighborhood. Upper confidence limits were omitted for estimates above one and lower confidence limits were omitted for estimates below one, for improved readability. See Supplementary Table S12 for tabulated estimates. Pooled models were further rescaled such that each country contributed equally to the estimates.

Figure S5. Results from Poisson regression models of school attendance on electricity

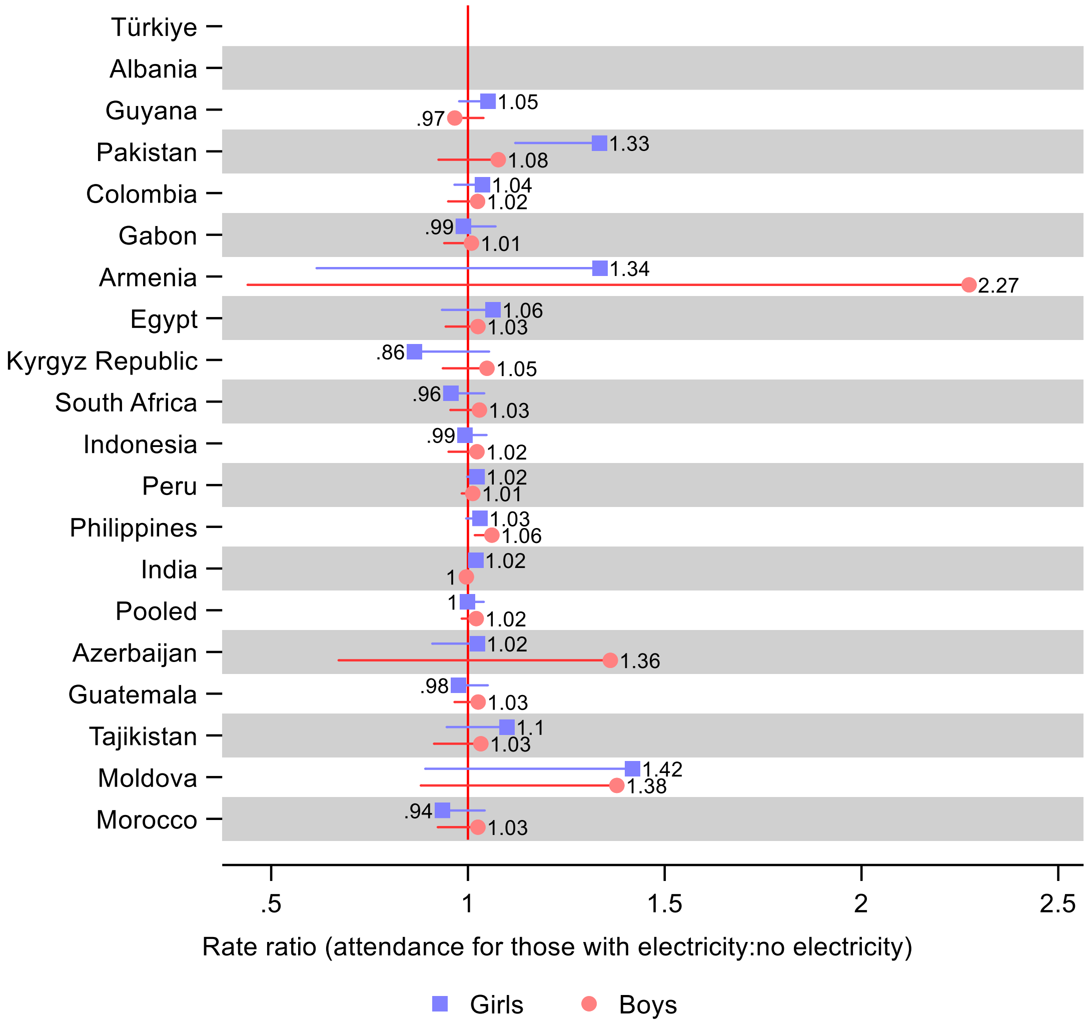

Notes: Countries were ordered according to the relative difference in rate ratio between boys and girls from Figure 1. 95% confidence intervals are shown. Estimates were weighted using sampling weights rescaled to sum up to one in each survey. All models included a baseline term for being female and baseline terms and interactions with being female for washer ownership, fridge ownership, TV ownership, having flush toilet, a wealth index z-score, number of household members, number of household members under age five, age, highest education level of a male in household, and highest education level of a female in household, as well as adjusting for neighborhood. Upper confidence limits were omitted for estimates above one and lower confidence limits were omitted for estimates below one, for improved readability. See Supplementary Table S13 for tabulated estimates. Pooled models were further rescaled such that each country contributed equally to the estimates.

Figure S6. Results from Poisson regression models of school attendance on car ownership

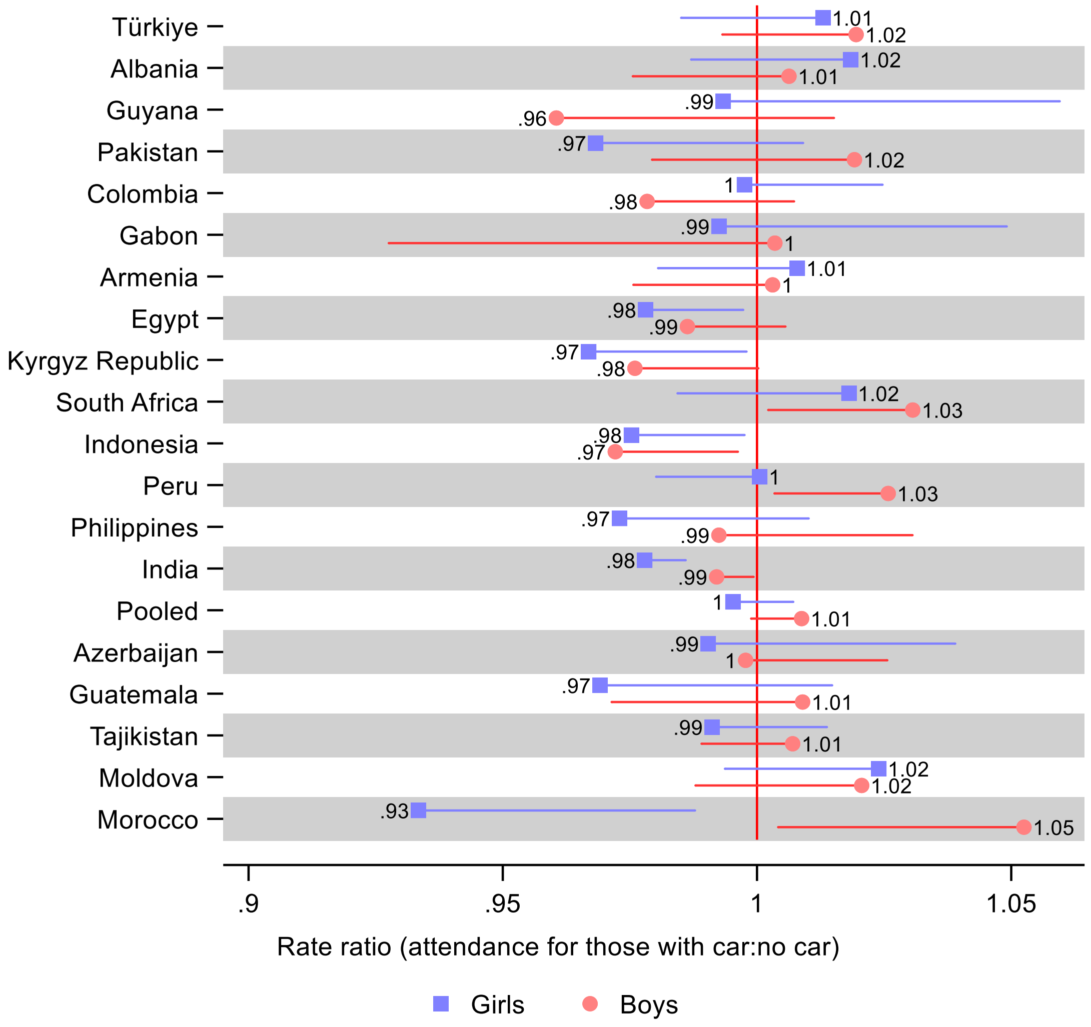

Notes: Countries were ordered according to the relative difference in rate ratio between boys and girls from Figure 1. 95% confidence intervals are shown. Estimates were weighted using sampling weights rescaled to sum up to one in each survey. All models included a baseline term for being female and baseline terms and interactions with being female for washer ownership, fridge ownership, TV ownership, having flush toilet, a wealth index z-score, number of household members, number of household members under age five, age, highest education level of a male in household, and highest education level of a female in household, as well as adjusting for neighborhood. Upper confidence limits were omitted for estimates above one and lower confidence limits were omitted for estimates below one, for improved readability. See Supplementary Table S14 for tabulated estimates. Pooled models were further rescaled such that each country contributed equally to the estimates.

Figure S7. Results from Poisson regression models of school attendance on scooter ownership

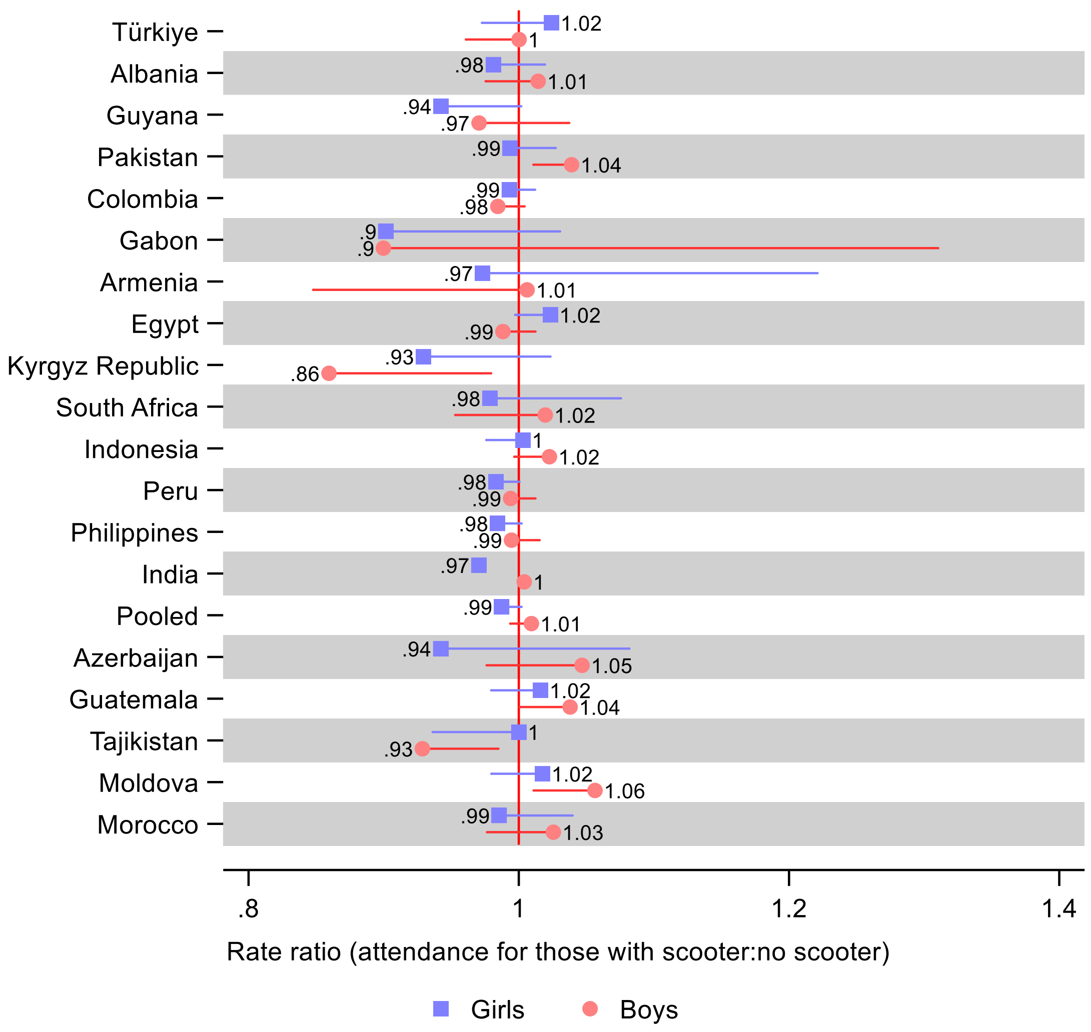

Notes: Countries were ordered according to the relative difference in rate ratio between boys and girls from Figure 1. 95% confidence intervals are shown. Estimates were weighted using sampling weights rescaled to sum up to one in each survey. All models included a baseline term for being female and baseline terms and interactions with being female for washer ownership, fridge ownership, TV ownership, having flush toilet, a wealth index z-score, number of household members, number of household members under age five, age, highest education level of a male in household, and highest education level of a female in household, as well as adjusting for neighborhood. Upper confidence limits were omitted for estimates above one and lower confidence limits were omitted for estimates below one, for improved readability. See Supplementary Table S15 for tabulated estimates. Pooled models were further rescaled such that each country contributed equally to the estimates.

Figure S8. Results from Poisson regression models of school attendance on clean cooking

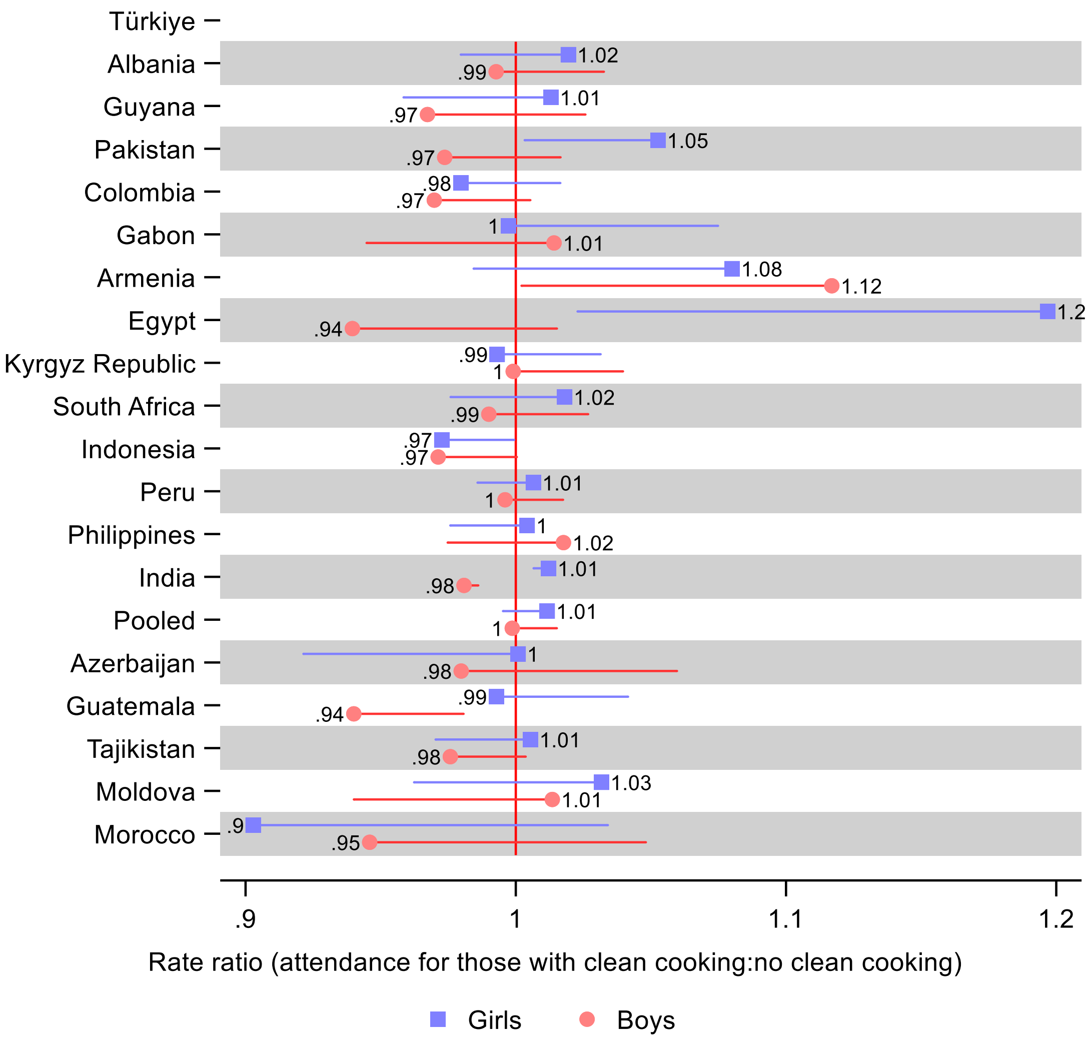

Notes: Countries were ordered according to the relative difference in rate ratio between boys and girls from Figure 1. 95% confidence intervals are shown. Estimates were weighted using sampling weights rescaled to sum up to one in each survey. All models included a baseline term for being female and baseline terms and interactions with being female for washer ownership, fridge ownership, TV ownership, having flush toilet, a wealth index z-score, number of household members, number of household members under age five, age, highest education level of a male in household, and highest education level of a female in household, as well as adjusting for neighborhood. Upper confidence limits were omitted for estimates above one and lower confidence limits were omitted for estimates below one, for improved readability. See Supplementary Table S16 for tabulated estimates. Pooled models were further rescaled such that each country contributed equally to the estimates.

Figure S9. Results from Poisson regression models of school attendance on flush toilet

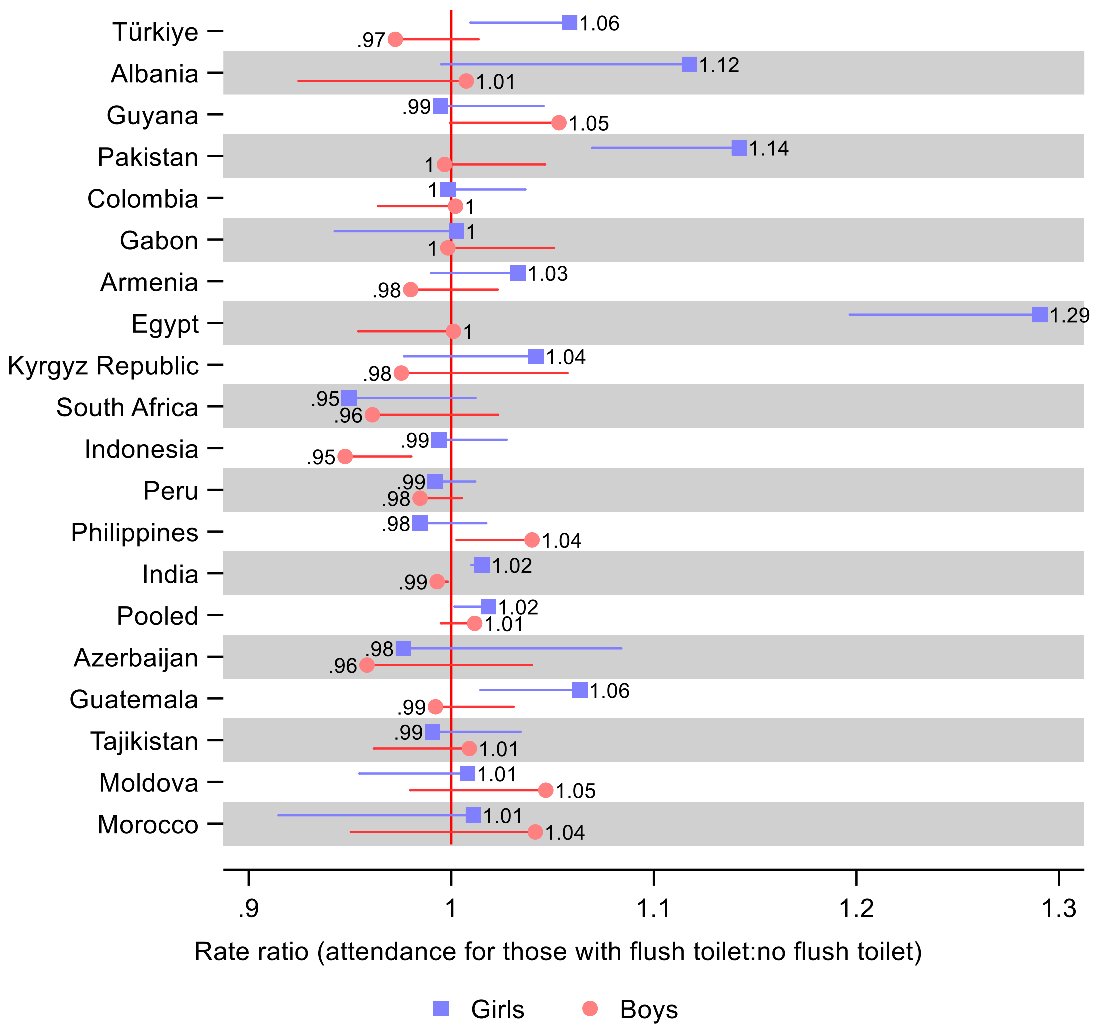

Notes: Countries were ordered according to the relative difference in rate ratio between boys and girls from Figure 1. 95% confidence intervals are shown. Estimates were weighted using sampling weights rescaled to sum up to one in each survey. All models included a baseline term for being female and baseline terms and interactions with being female for washer ownership, fridge ownership, TV ownership, having flush toilet, a wealth index z-score, number of household members, number of household members under age five, age, highest education level of a male in household, and highest education level of a female in household, as well as adjusting for neighborhood. Upper confidence limits were omitted for estimates above one and lower confidence limits were omitted for estimates below one, for improved readability. See Supplementary Table S17 for tabulated estimates. Pooled models were further rescaled such that each country contributed equally to the estimates.

Figure S10. Results from Poisson regression models of school attendance on TV ownership

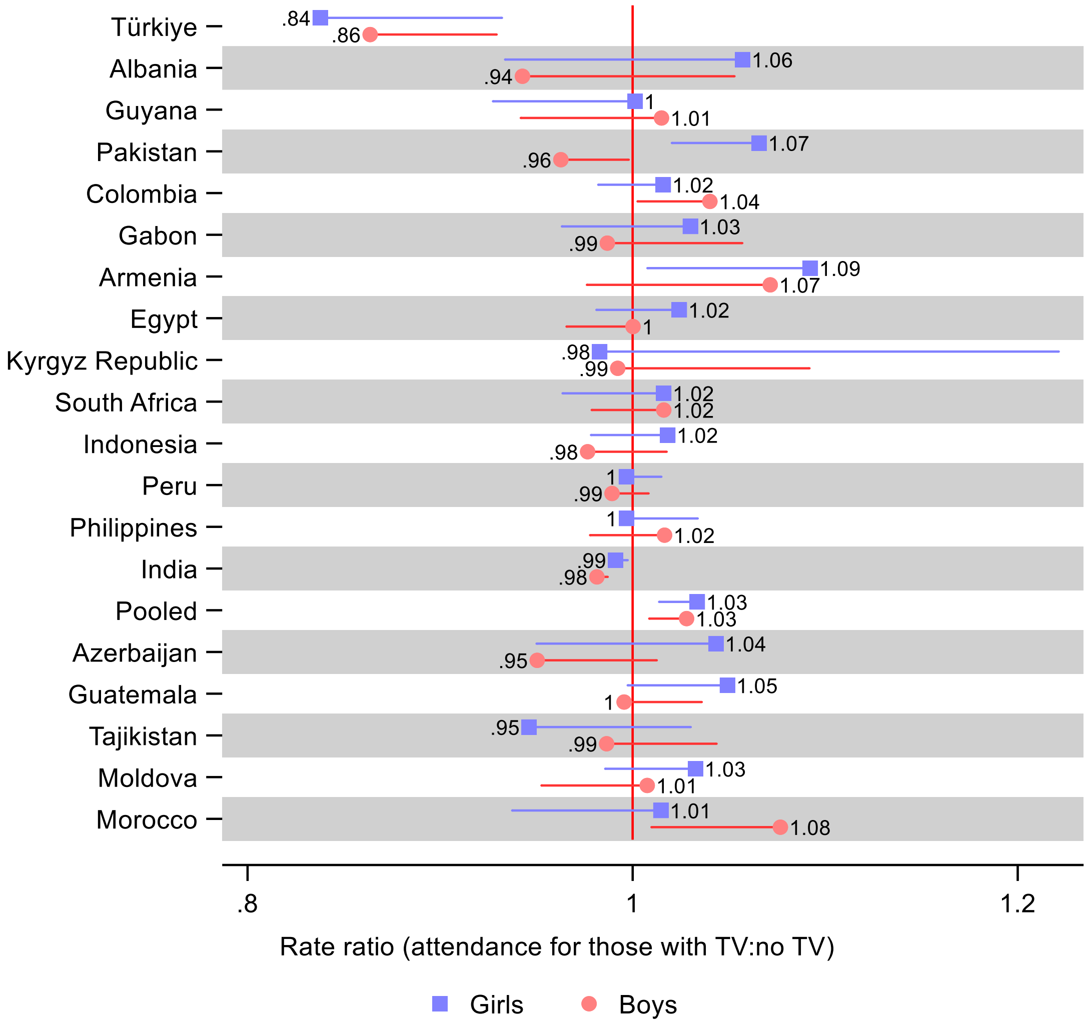

Notes: Countries were ordered according to the relative difference in rate ratio between boys and girls from Figure 1. 95% confidence intervals are shown. Estimates were weighted using sampling weights rescaled to sum up to one in each survey. All models included a baseline term for being female and baseline terms and interactions with being female for washer ownership, fridge ownership, TV ownership, having flush toilet, a wealth index z-score, number of household members, number of household members under age five, age, highest education level of a male in household, and highest education level of a female in household, as well as adjusting for neighborhood. Upper confidence limits were omitted for estimates above one and lower confidence limits were omitted for estimates below one, for improved readability. See Supplementary Table S18 for tabulated estimates. Pooled models were further rescaled such that each country contributed equally to the estimates.

Figure S11. Results from logistic regression models of school attendance on washer ownership

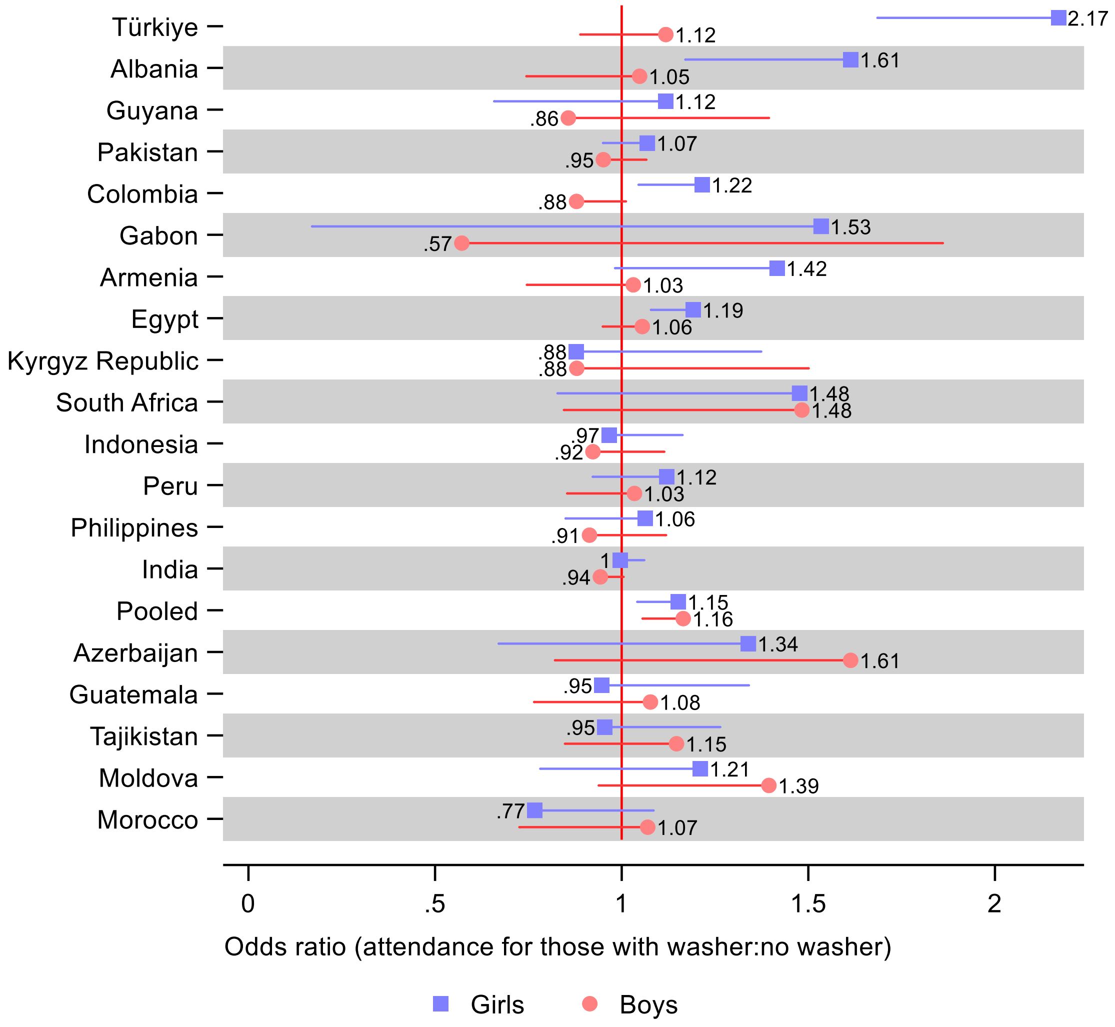

Notes: Countries were ordered according to the relative difference in rate ratio between boys and girls from Figure 1. 95% confidence intervals are shown. Estimates were weighted using sampling weights rescaled to sum up to one in each survey. All models included a baseline term for being female and baseline terms and interactions with being female for washer ownership, fridge ownership, TV ownership, having flush toilet, a wealth index z-score, number of household members, number of household members under age five, age, highest education level of a male in household, and highest education level of a female in household, as well as adjusting for neighborhood. Upper confidence limits were omitted for estimates above one and lower confidence limits were omitted for estimates below one, for improved readability. See Supplementary Table S19 for tabulated estimates. Pooled models were further rescaled such that each country contributed equally to the estimates.

Figure S12. Results from linear regression models of school attendance on washer ownership

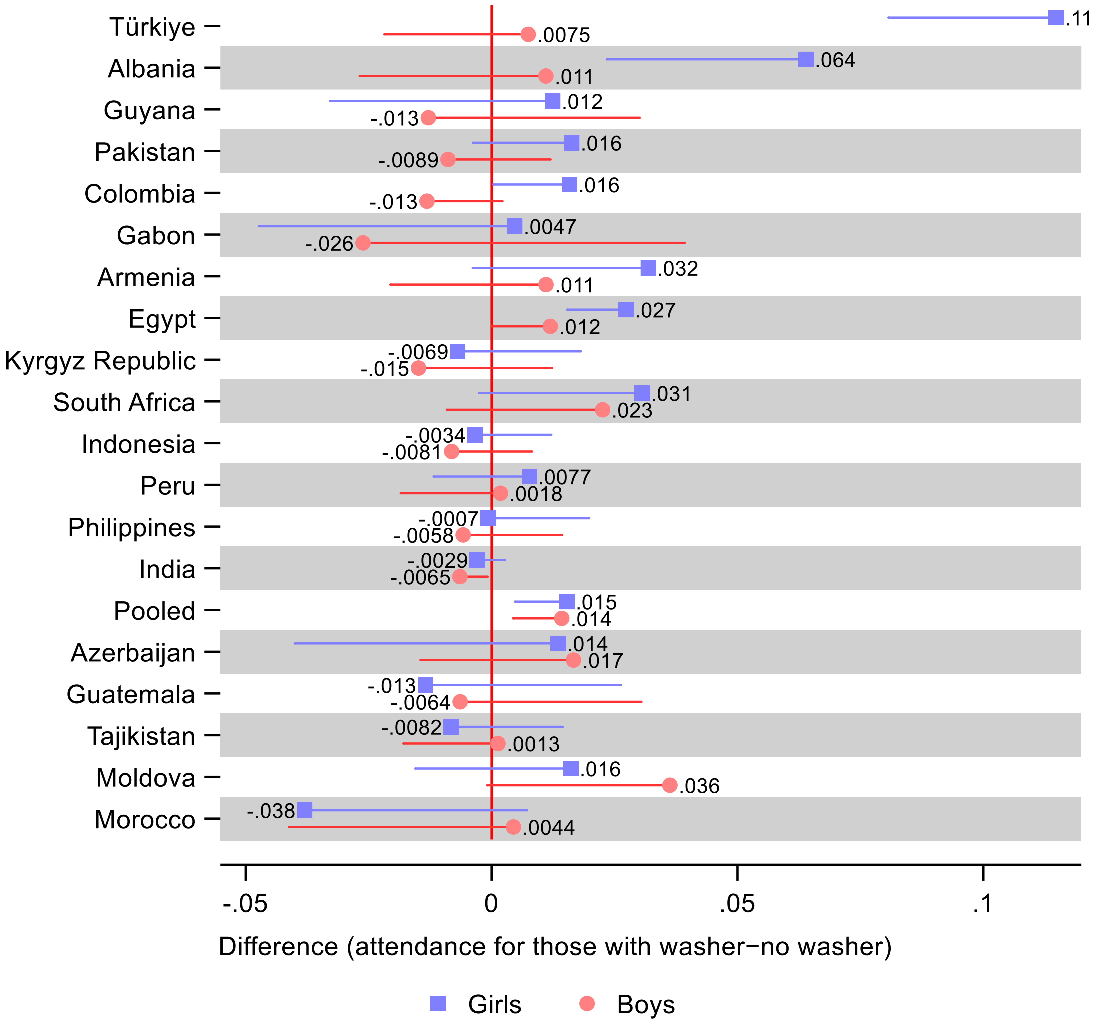

Notes: Countries were ordered according to the relative difference in rate ratio between boys and girls from Figure 1. 95% confidence intervals are shown. Estimates were weighted using sampling weights rescaled to sum up to one in each survey. All models included a baseline term for being female and baseline terms and interactions with being female for washer ownership, fridge ownership, TV ownership, having flush toilet, a wealth index z-score, number of household members, number of household members under age five, age, highest education level of a male in household, and highest education level of a female in household, as well as adjusting for neighborhood. Upper confidence limits were omitted for estimates above one and lower confidence limits were omitted for estimates below one, for improved readability. See Supplementary Table S20 for tabulated estimates. Pooled models were further rescaled such that each country contributed equally to the estimates.

Figure S13. Results from Poisson regression models of school attendance on washer ownership: excluding flush toilet, fridge, and TV from independent variables

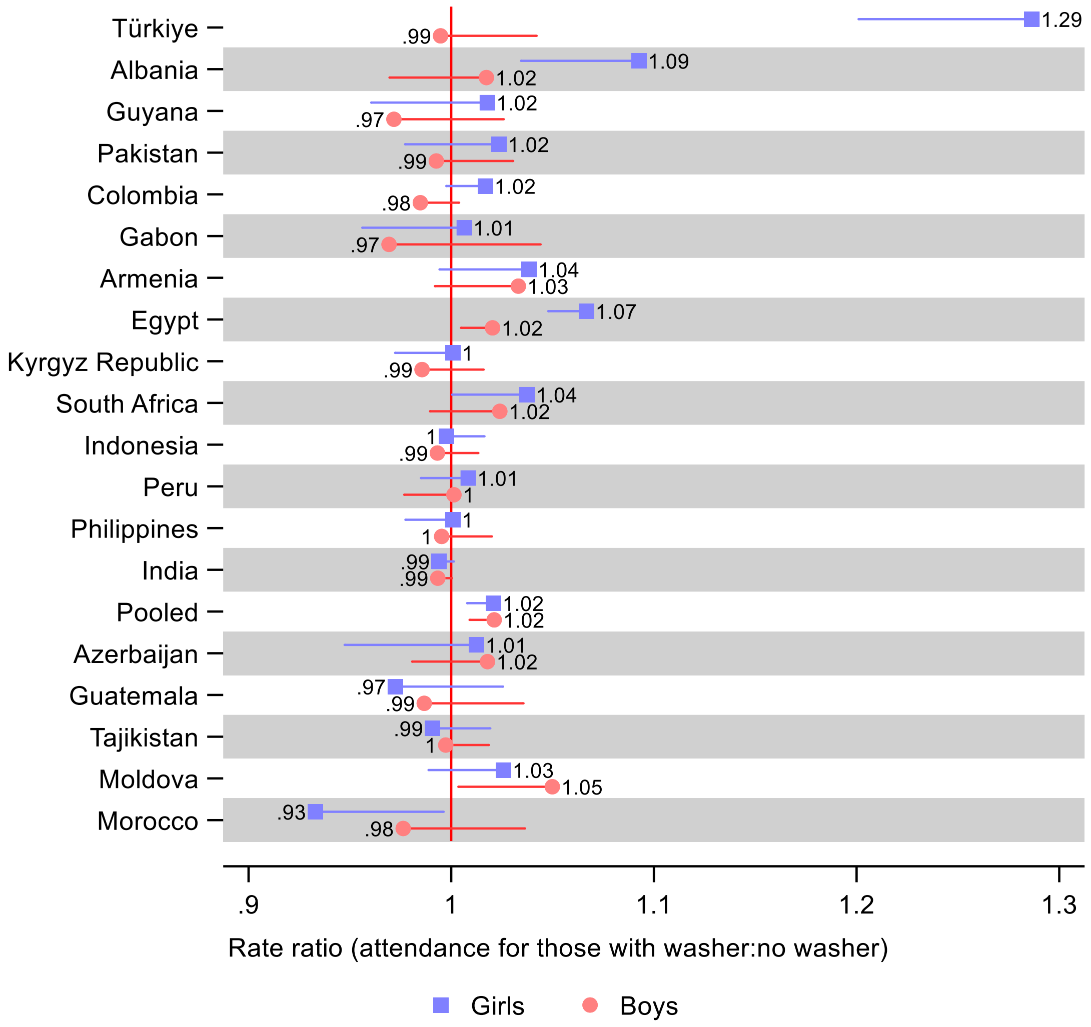

Notes: Countries were ordered according to the relative difference in rate ratio between boys and girls from Figure 1. 95% confidence intervals are shown. Estimates were weighted using sampling weights rescaled to sum up to one in each survey. All models included a baseline term for being female and baseline terms and interactions with being female for washer ownership, a wealth index z-score, number of household members, number of household members under age five, age, highest education level of a male in household, and highest education level of a female in household, as well as adjusting for neighborhood. Upper confidence limits were omitted for estimates above one and lower confidence limits were omitted for estimates below one, for improved readability. See Supplementary Table S21 for tabulated estimates. Pooled models were further rescaled such that each country contributed equally to the estimates.

Figure S14. Results from Poisson regression models of school attendance on washer ownership: excluding the wealth index from independent variables

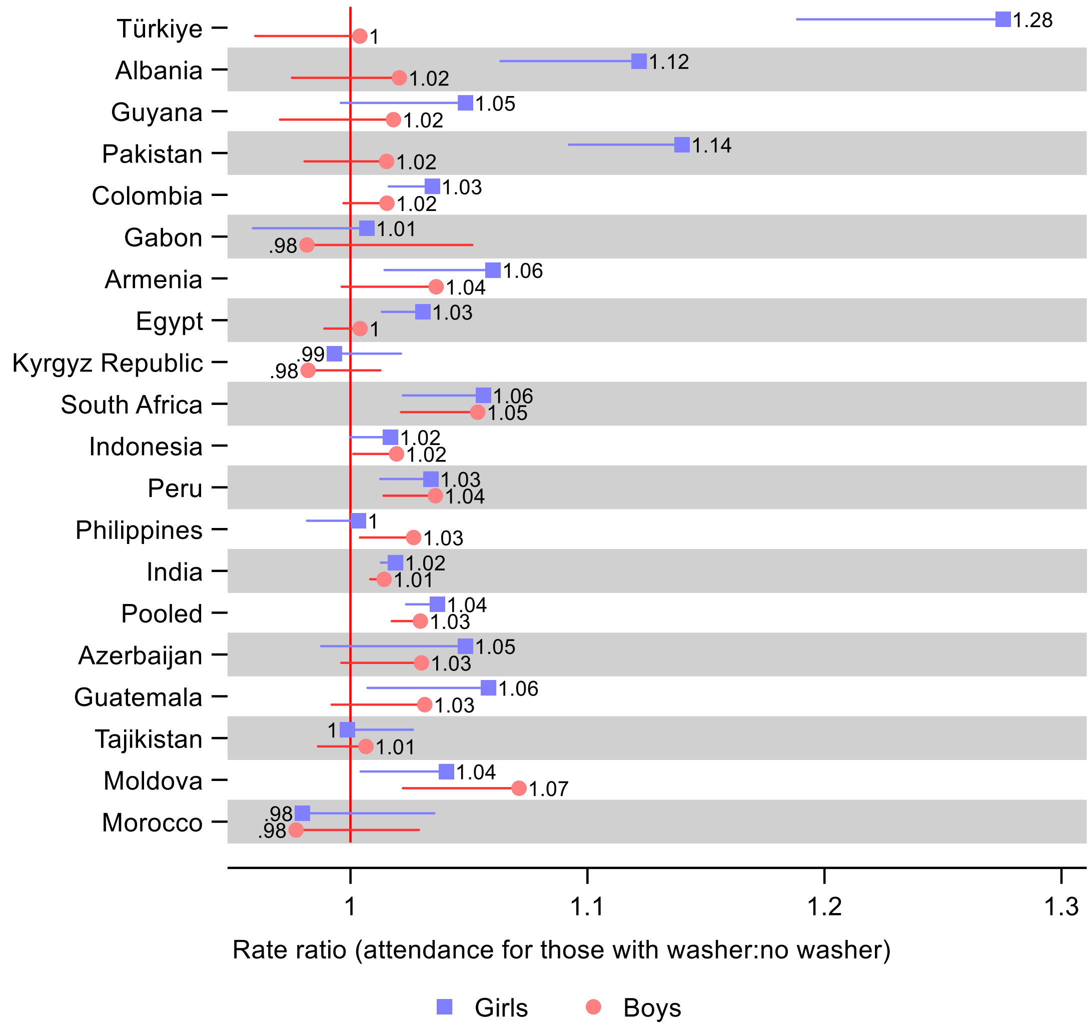

Notes: Countries were ordered according to the relative difference in rate ratio between boys and girls from Figure 1. 95% confidence intervals are shown. Estimates were weighted using sampling weights rescaled to sum up to one in each survey. Pooled models were further rescaled such that each country contributed equally to the estimates. All models included a baseline term for being female and baseline terms and interactions with being female for washer ownership, fridge ownership, TV ownership, having flush toilet, number of household members, number of household members under age five, age, highest education level of a male in household, and highest education level of a female in household, as well as adjusting for neighborhood. Upper confidence limits were omitted for estimates above one and lower confidence limits were omitted for estimates below one, for improved readability. See Supplementary Table S22 for tabulated estimates.

Figure S15. Results from Poisson regression models of school attendance on washer ownership: only including descendants of household head

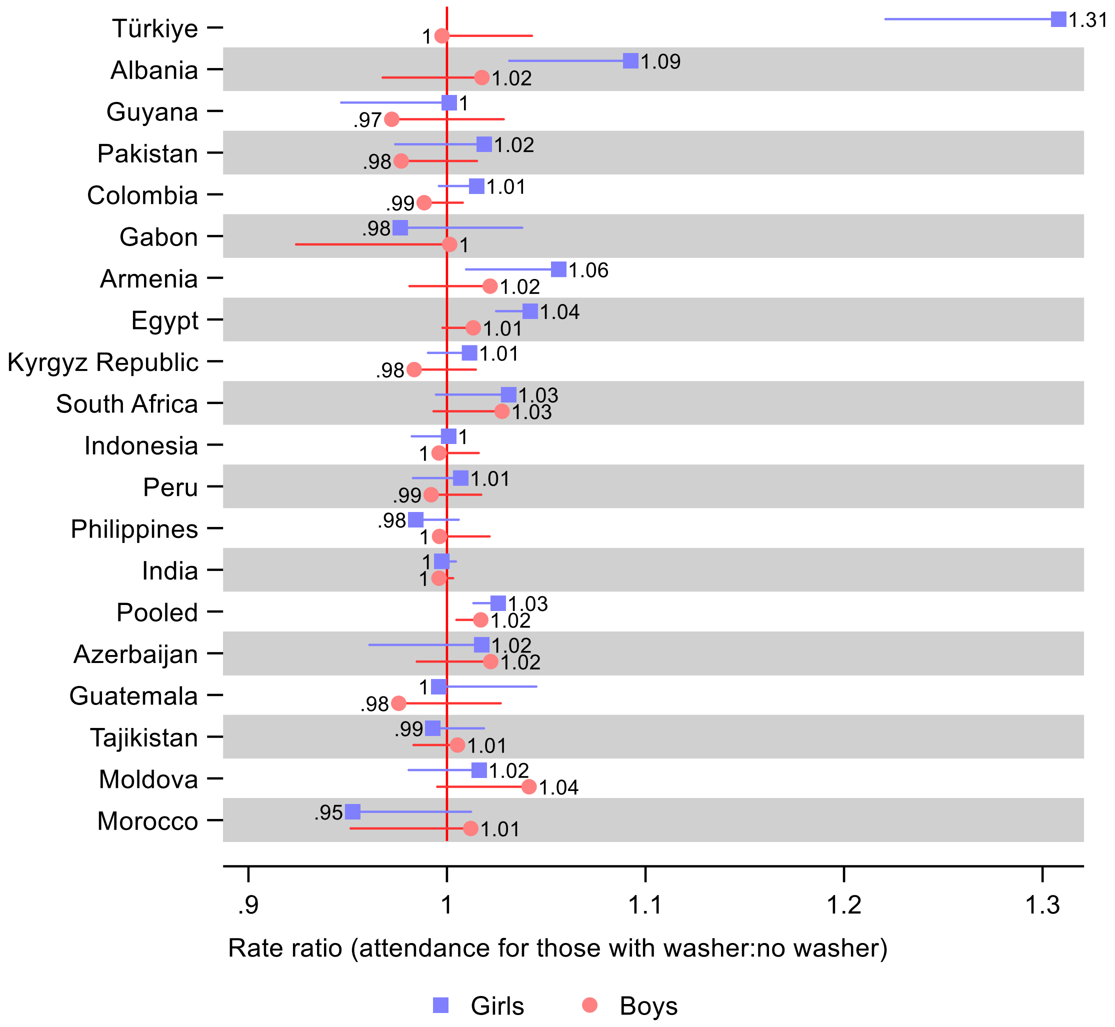

Notes: Countries were ordered according to the relative difference in rate ratio between boys and girls from Figure 1. 95% confidence intervals are shown. Estimates were weighted using sampling weights rescaled to sum up to one in each survey. All models included a baseline term for being female and baseline terms and interactions with being female for washer ownership, fridge ownership, TV ownership, having flush toilet, a wealth index z-score, number of household members, number of household members under age five, age, highest education level of a male in household, and highest education level of a female in household, as well as adjusting for neighborhood. Upper confidence limits were omitted for estimates above one and lower confidence limits were omitted for estimates below one, for improved readability. See Supplementary Table S23 for tabulated estimates. Pooled models were further rescaled such that each country contributed equally to the estimates.

Figure S16. Results from Poisson regression models of school attendance on washer ownership: only including households with electricity

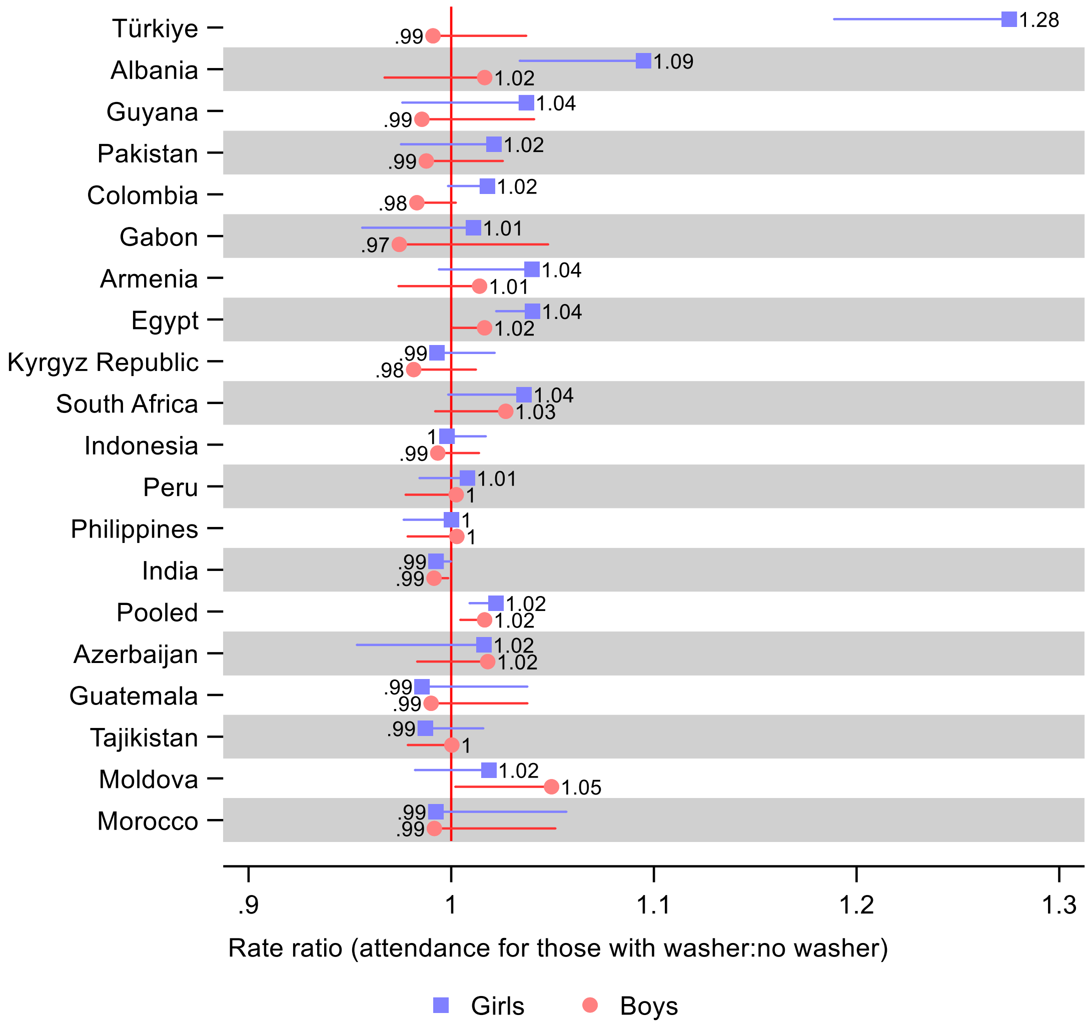

Notes: Countries were ordered according to the relative difference in rate ratio between boys and girls from Figure 1. 95% confidence intervals are shown. Estimates were weighted using sampling weights rescaled to sum up to one in each survey. All models included a baseline term for being female and baseline terms and interactions with being female for washer ownership, fridge ownership, TV ownership, having flush toilet, a wealth index z-score, number of household members, number of household members under age five, age, highest education level of a male in household, and highest education level of a female in household, as well as adjusting for neighborhood. Upper confidence limits were omitted for estimates above one and lower confidence limits were omitted for estimates below one, for improved readability. See Supplementary Table S24 for tabulated estimates. Pooled models were further rescaled such that each country contributed equally to the estimates.

Figure S17. Results from Poisson regression models of school attendance on washer ownership: stratified by overall school attendance in region

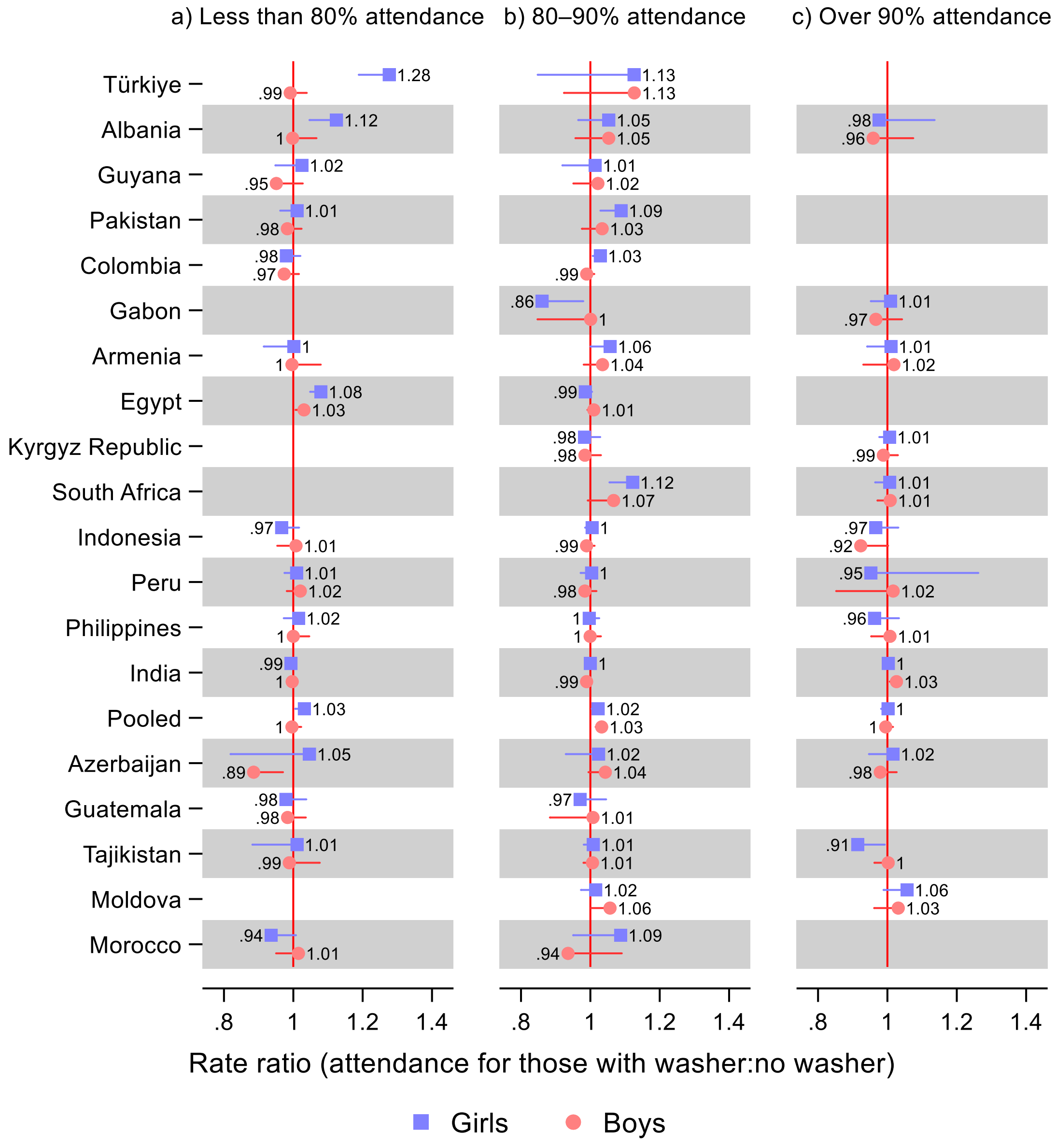

Notes: Countries were ordered according to the relative difference in rate ratio between boys and girls from Figure 1. 95% confidence intervals are shown. Estimates were weighted using sampling weights rescaled to sum up to one in each survey. All models included a baseline term for being female and baseline terms and interactions with being female for washer ownership, fridge ownership, TV ownership, having flush toilet, a wealth index z-score, number of household members, number of household members under age five, age, highest education level of a male in household, and highest education level of a female in household, as well as adjusting for neighborhood. Upper confidence limits were omitted for estimates above one and lower confidence limits were omitted for estimates below one, for improved readability. See Supplementary Table S25 for tabulated estimates. Pooled models were further rescaled such that each country contributed equally to the estimates.

Figure S18. Results from linear regression models of school attendance on washer ownership: number of complete years of education as an outcome

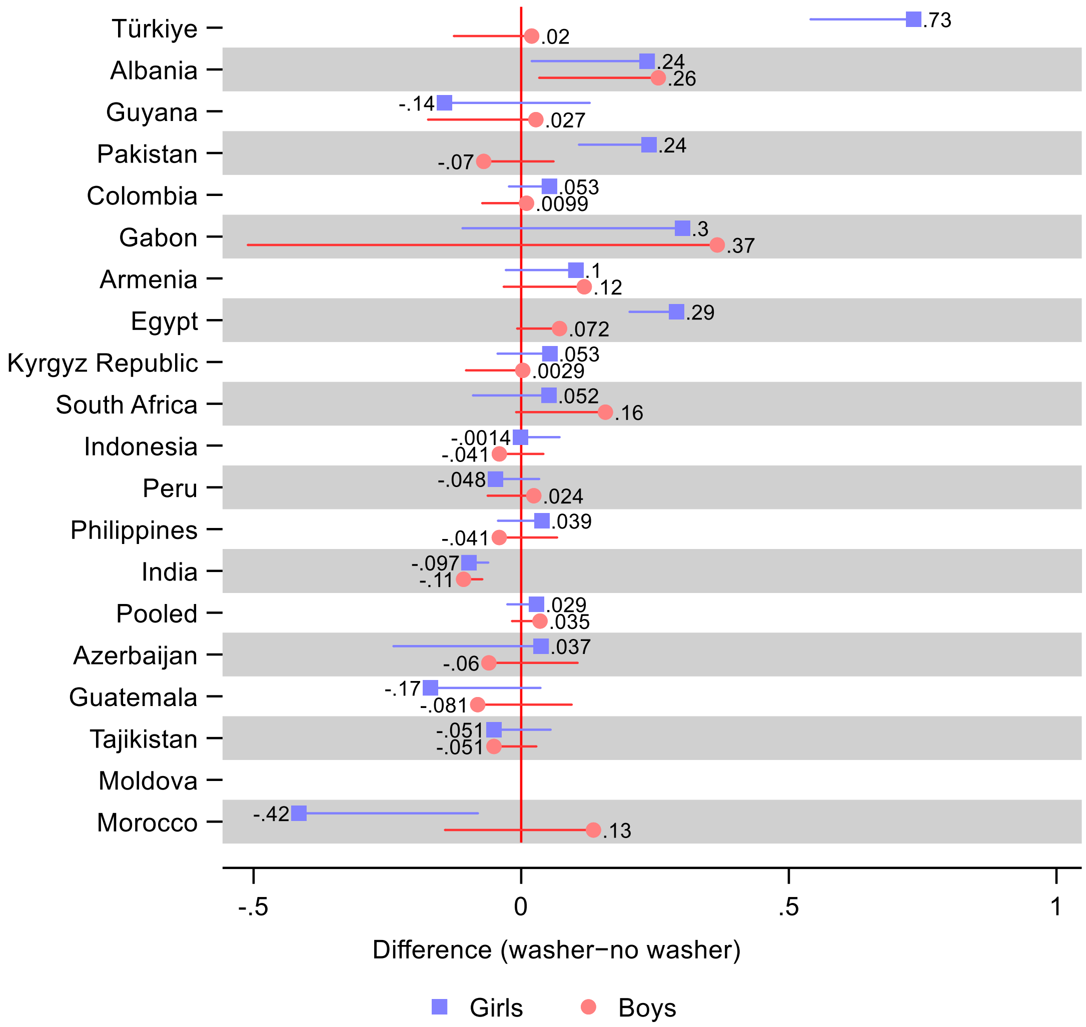

Notes: Countries were ordered according to the relative difference in rate ratio between boys and girls from Figure 1. 95% confidence intervals are shown. Estimates were weighted using sampling weights rescaled to sum up to one in each survey. All models included a baseline term for being female and baseline terms and interactions with being female for washer ownership, fridge ownership, TV ownership, having flush toilet, a wealth index z-score, number of household members, number of household members under age five, age, highest education level of a male in household, and highest education level of a female in household, as well as adjusting for neighborhood. Upper confidence limits were omitted for estimates above one and lower confidence limits were omitted for estimates below one, for improved readability. See Supplementary Table S26 for tabulated estimates. Pooled models were further rescaled such that each country contributed equally to the estimates.

Figure S19. Results from linear regression models of school attendance on washer ownership: number of complete years of education as an outcome, by survey year

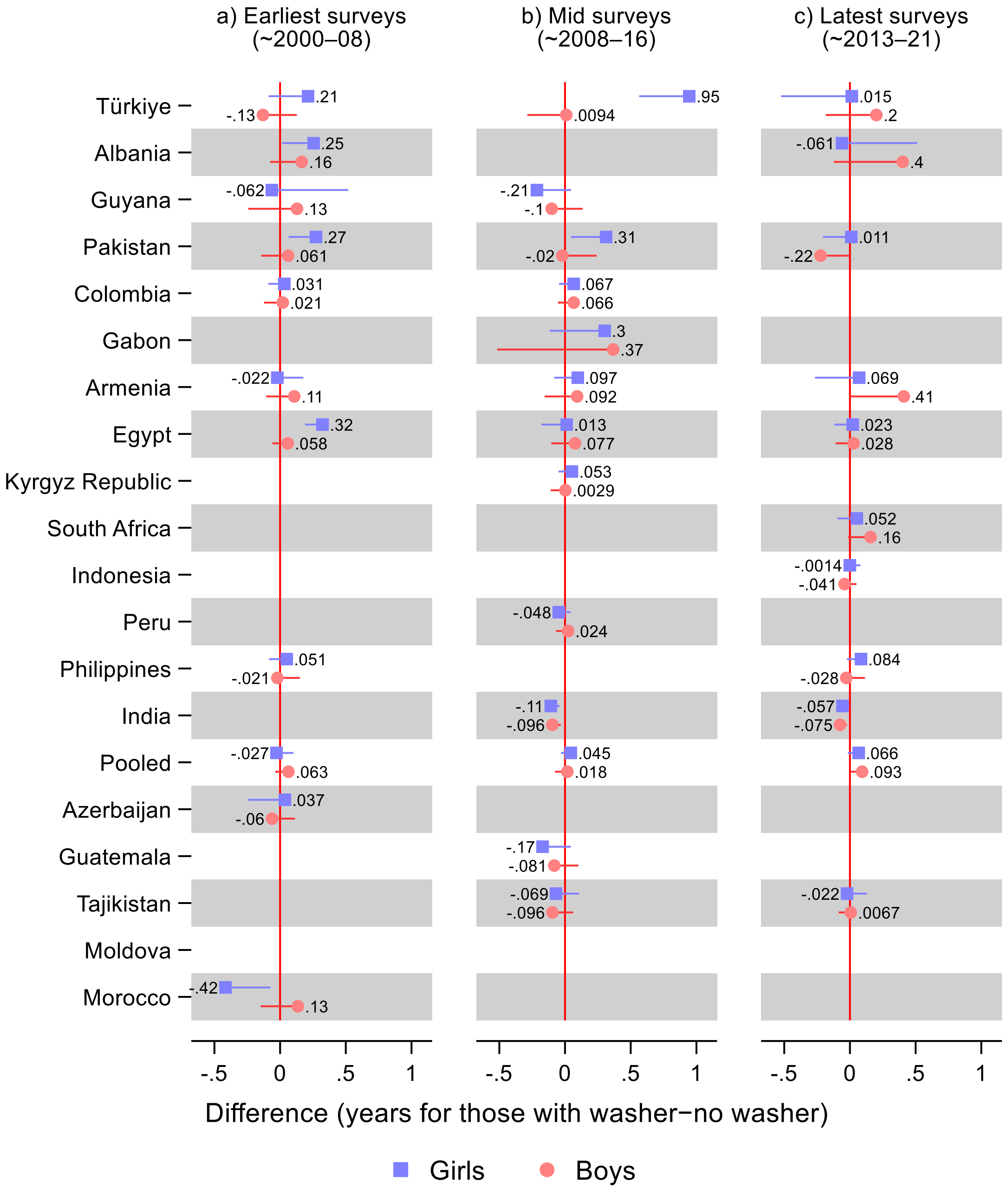

Notes: Countries were ordered according to the relative difference in rate ratio between boys and girls from Figure 1. 95% confidence intervals are shown. Estimates were weighted using sampling weights rescaled to sum up to one in each survey. Pooled models were further rescaled such that each country contributed equally to the estimates. All models included a baseline term for being female and baseline terms and interactions with being female for washer ownership, fridge ownership, TV ownership, having flush toilet, a wealth index z-score, number of household members, number of household members under age five, age, highest education level of a male in household, and highest education level of a female in household, as well as adjusting for neighborhood. Upper confidence limits were omitted for estimates above one and lower confidence limits were omitted for estimates below one, for improved readability. See Supplementary Table S27 for tabulated estimates.

Figure S20. Results from Poisson regression models of school attendance on washer ownership: stratified by the extent of female disadvantage in school attendance at the regional level

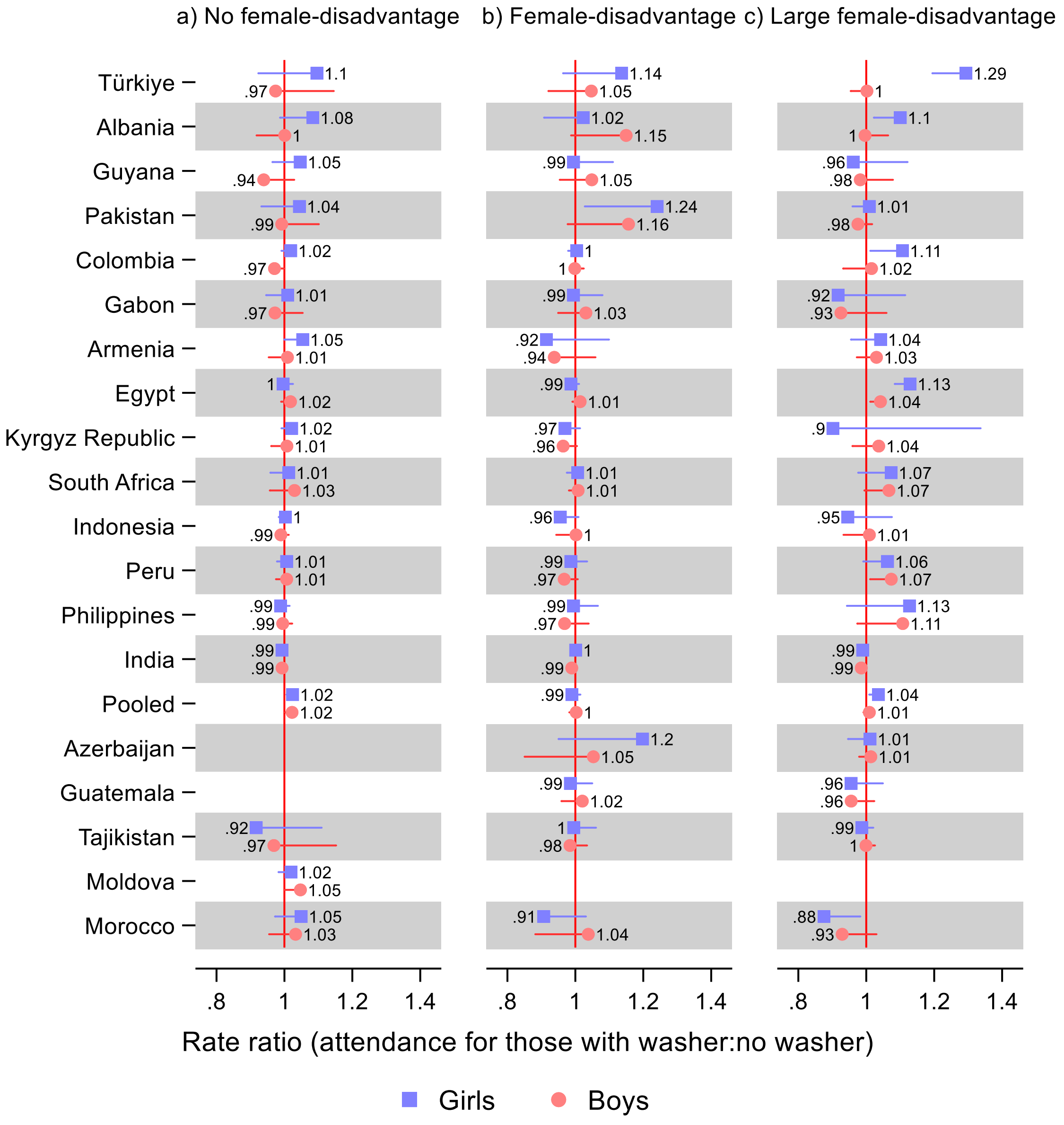

Notes: Countries were ordered according to the relative difference in the two rate ratios from Figure 1. 95% confidence intervals are shown. Estimates were weighted using sampling weights rescaled to sum up to one in each survey. Pooled models were further rescaled such that each country contributed equally to the estimates. All models included a baseline term for being female and baseline terms and interactions with being female for washer ownership, fridge ownership, TV ownership, having flush toilet, a wealth index z-score, number of household members, number of household members under age five, age, highest education level of a male in household, and highest education level of a female in household, as well as adjusting for neighborhood level factors. Upper confidence limits were omitted for estimates above one and lower confidence limits were omitted for estimates below one, for improved readability. See Supplementary Table S28 for tabulated estimates.

Figure S21. Results from Poisson regression models of school attendance on being female interacted with washer ownership adjusting for all household level factors

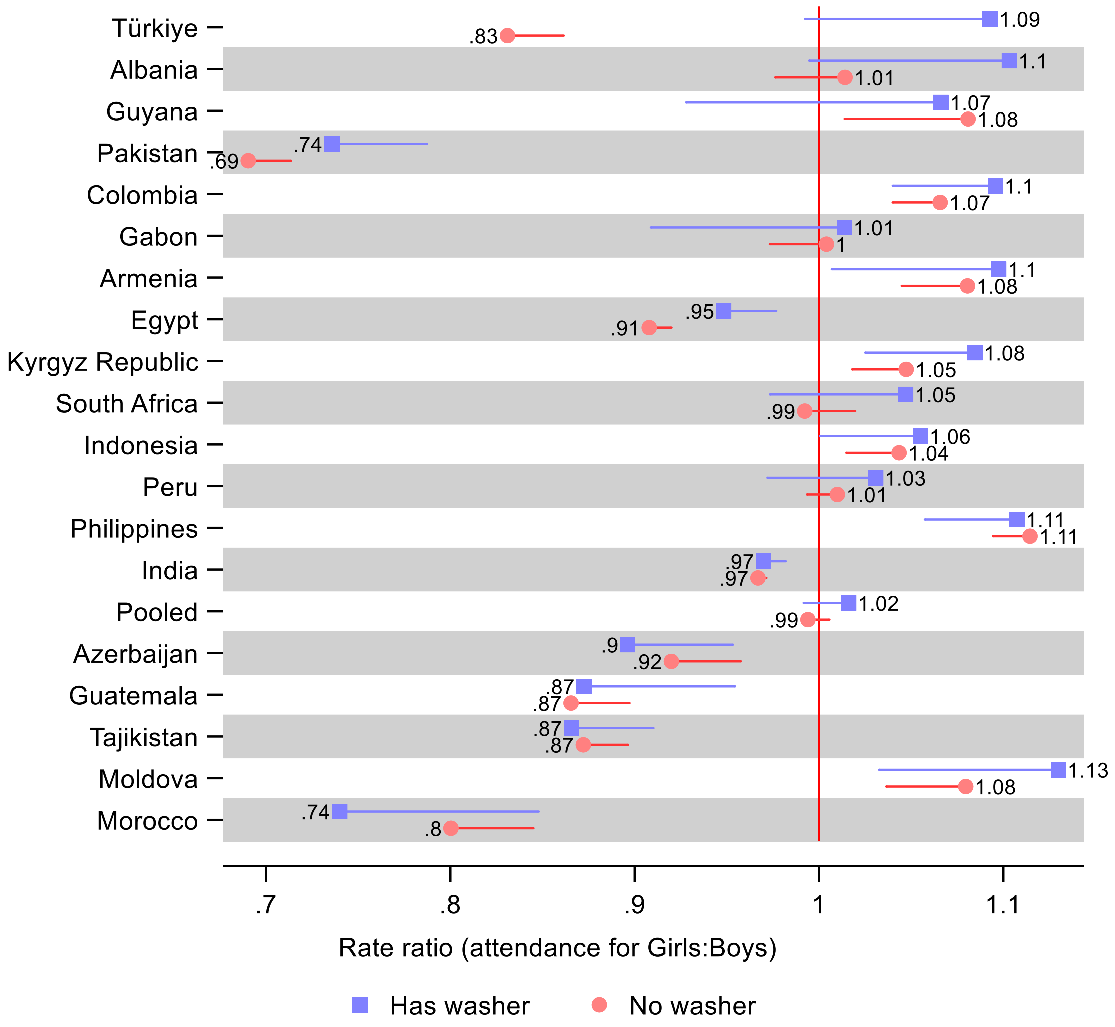

Notes: Countries were ordered according to the relative difference in the two rate ratios from Figure 1. Households without at least one girl and one boy were excluded. 95% confidence intervals are shown. Estimates were weighted using sampling weights rescaled to sum up to one in each survey. Pooled models were further rescaled such that each country contributed equally to the estimates. All models included a baseline term for being female and age and interactions with being female for washer ownership, fridge ownership, TV ownership, having flush toilet, a wealth index z-score, number of household members, number of household members under age five, age, highest education level of a male in household, and highest education level of a female in household, as well as adjusting for household level factors using household level means of all independent variables (baseline and interactions with being female). Upper confidence limits were omitted for estimates above one and lower confidence limits were omitted for estimates below one, for improved readability. See Supplementary Table S29 for tabulated estimates.

SUPPLEMENT 5: Tabulated estimates from sensitivity analyses Figures: Main model parameters and combinations

Table S11. Results from Poisson regression models of school attendance on fridge ownership

	Constant	Fridge	Female	Interaction	Constant× Female	Interaction× Fridge	
Pooled	.799***	1.03***	.993	.987	.794***	1.017**	N=1,614,264
	[.794, .805]	[1.016, 1.044]	[.985, 1.001]	[.968, 1.007]	[.788, .799]	[1.002, 1.032]	
Albania	.808***	1.03	1	.941	.808***	.969	N=13,749
	[.793, .823]	[.963, 1.102]	[.976, 1.025]	[.852, 1.039]	[.794, .822]	[.89, 1.055]	
Armenia	.79***	1.044	1.077***	.95	.851***	.991	N=10,925
	[.774, .806]	[.991, 1.1]	[1.05, 1.105]	[.884, 1.02]	[.839, .863]	[.938, 1.048]	
Azerbaijan	.881***	.9956	.927***	.992	.817***	.987	N=6,203
	[.865, .898]	[.956, 1.036]	[.899, .956]	[.913, 1.077]	[.8, .834]	[.923, 1.055]	
Colombia	.752***	1.025**	1.034***	.998	.778***	1.023*	N=61,245
	[.744, .761]	[1, 1.051]	[1.019, 1.05]	[.968, 1.029]	[.77, .786]	[.9988, 1.048]	
Egypt	.758***	1.054***	.909***	1.068***	.689***	1.126***	N=102,079
	[.752, .763]	[1.03, 1.079]	[.9, .919]	[1.035, 1.102]	[.682, .695]	[1.094, 1.159]	
Gabon	.89***	1.018	1.001	.984	.891***	1.001	N=8,462
	[.875, .906]	[.974, 1.064]	[.977, 1.025]	[.921, 1.051]	[.878, .905]	[.964, 1.04]	
Guatemala	.666***	1.029	.872***	.993	.581***	1.022	N=24,453
	[.652, .68]	[.994, 1.066]	[.848, .896]	[.948, 1.041]	[.566, .595]	[.985, 1.061]	
Guyana	.691***	.994	1.019	1.005	.704***	.9994	N=6,801
	[.666, .716]	[.935, 1.058]	[.974, 1.066]	[.928, 1.089]	[.682, .726]	[.943, 1.06]	
India	.756***	.998	.958***	.992**	.724***	.99***	N=1,075,968
	[.754, .758]	[.992, 1.004]	[.955, .961]	[.984, .9991]	[.722, .726]	[.983, .9959]	
Indonesia	.771***	1.009	1.03***	1	.794***	1.009	N=35,687
	[.762, .78]	[.986, 1.033]	[1.014, 1.047]	[.972, 1.03]	[.785, .804]	[.987, 1.033]	
Kyrgyz Republic	.881***	1.022	1.025**	1.017	.903***	1.038**	N=6,551
	[.866, .897]	[.993, 1.051]	[1.004, 1.046]	[.972, 1.064]	[.89, .917]	[1, 1.078]	
Moldova	.819***	1.046	1.088***	.987	.891***	1.032	N=5,457
	[.802, .836]	[.977, 1.121]	[1.061, 1.115]	[.908, 1.072]	[.877, .905]	[.979, 1.089]	
Morocco	.584***	1.152***	.812***	.932	.474***	1.074**	N=14,022
	[.567, .601]	[1.082, 1.226]	[.781, .844]	[.85, 1.023]	[.456, .493]	[1, 1.153]	
Pakistan	.511***	1.122***	.698***	.929***	.357***	1.042*	N=70,840
	[.5, .523]	[1.084, 1.162]	[.678, .719]	[.885, .975]	[.346, .368]	[.9975, 1.089]	
Peru	.76***	.994	.9975	1.017	.758***	1.011	N=82,853
	[.754, .767]	[.976, 1.012]	[.987, 1.008]	[.994, 1.041]	[.751, .765]	[.993, 1.029]	
Philippines	.768***	1.031**	1.061***	1.002	.814***	1.033**	N=39,196
	[.759, .777]	[1.005, 1.057]	[1.044, 1.077]	[.97, 1.036]	[.805, .824]	[1.006, 1.061]	
South Africa	.912***	1.044*	.977**	1.013	.891***	1.057*	N=7,000
	[.899, .924]	[.9997, 1.09]	[.958, .9968]	[.95, 1.081]	[.878, .904]	[.9974, 1.121]	
Tajikistan	.87***	.9978	.869***	1.024	.756***	1.022	N=16,544
	[.859, .88]	[.974, 1.022]	[.85, .889]	[.984, 1.065]	[.741, .771]	[.989, 1.055]	
Türkiye	.726***	1.117**	.874***	.925	.634***	1.032	N=26,229
	[.714, .737]	[1.017, 1.226]	[.853, .894]	[.815, 1.049]	[.621, .647]	[.928, 1.149]	

Notes: *P<0.1; **P<0.05; ***P<0.01. Rate ratios and a constant from Poisson regression models are shown, in addition to two combinations obtained using post estimation. Each row of estimates was obtained from a separate model. All models included a baseline term for being female and baseline terms and interactions with being female for washer ownership, fridge ownership, TV ownership, having flush toilet, a wealth index z-score, number of household members, number of household members under age five, age, highest education level of a male in household, and highest education level of a female in household, as well as adjusting for neighborhood. Except the baseline term for female, all independent variables, including fixed effects, were centered around a country-specific weighted mean: Therefore, the 'Constant' column shows school attendance for males with the mean on all other independent variables. The column labelled 'Fridge' shows differences in school attendance for males with fridge at home. The column labelled 'Interaction' shows interaction terms for females and fridge. The column labelled 'Constant×Female' shows school attendance for females with the mean on all other independent variables, obtained using post estimation. The column labelled 'Interaction×Fridge' shows difference in school attendance for girls with fridge at home, obtained using post estimation. Estimates were weighted using sampling weights, rescaled to sum up to one for the final sample from each survey. 95% confidence intervals adjusted for clustering at the level of primary sampling units are shown in brackets below the point estimates. Pooled models were further rescaled such that each country contributed equally to the estimates.

Table S12. Results from Poisson regression models of school attendance on piped water

	Constant	Piped water	Female	Interaction	Constant× Female	Interaction× Piped water	
Pooled	.8***	.987*	.995	1.024***	.796***	1.011	N=1,605,539
	[.795, .806]	[.974, 1.001]	[.987, 1.003]	[1.009, 1.039]	[.791, .802]	[.9966, 1.025]	
Albania	.808***	.974	1	1.006	.808***	.98	N=13,749
	[.793, .823]	[.941, 1.008]	[.976, 1.025]	[.97, 1.044]	[.794, .822]	[.943, 1.018]	
Armenia	.79***	.961	1.077***	1.004	.851***	.965	N=10,923
	[.774, .806]	[.887, 1.041]	[1.05, 1.105]	[.935, 1.079]	[.838, .863]	[.896, 1.039]	
Azerbaijan	.881***	.988	.927***	.985	.817***	.973	N=6,203
	[.865, .897]	[.948, 1.029]	[.9, .956]	[.931, 1.041]	[.8, .834]	[.925, 1.024]	
Colombia	.753***	1.022	1.034***	.97*	.778***	.992	N=61,245
	[.744, .761]	[.992, 1.053]	[1.018, 1.05]	[.941, 1.001]	[.77, .786]	[.964, 1.021]	
Egypt	.758***	.981	.909***	1.065***	.689***	1.045***	N=102,075
	[.752, .763]	[.955, 1.008]	[.9, .919]	[1.028, 1.103]	[.682, .695]	[1.011, 1.081]	
Gabon	.89***	.994	1.001	1.003	.891***	.9975	N=8,435
	[.875, .906]	[.923, 1.072]	[.977, 1.026]	[.915, 1.099]	[.878, .904]	[.962, 1.034]	
Guatemala	.666***	.9992	.872***	1.053**	.58***	1.052**	N=24,453
	[.652, .68]	[.968, 1.032]	[.848, .896]	[1.007, 1.102]	[.566, .595]	[1.011, 1.095]	
Guyana	.69***	1.006	1.019	1.066**	.704***	1.073***	N=6,797
	[.666, .716]	[.963, 1.05]	[.975, 1.066]	[1.013, 1.123]	[.682, .726]	[1.028, 1.119]	
India	.756***	.989***	.958***	1.005*	.724***	.994**	N=1,075,968
	[.754, .758]	[.985, .994]	[.955, .961]	[.9997, 1.01]	[.722, .726]	[.989, .9991]	
Indonesia	.771***	1.024*	1.031***	.9989	.794***	1.023*	N=35,684
	[.761, .78]	[.9989, 1.05]	[1.014, 1.047]	[.97, 1.028]	[.785, .804]	[.9984, 1.048]	
Kyrgyz Republic	.881***	.969	1.025**	.987	.904***	.956**	N=6,545
	[.866, .897]	[.929, 1.01]	[1.005, 1.047]	[.951, 1.024]	[.891, .917]	[.92, .994]	
Moldova	.819***	.964	1.088***	1.045*	.891***	1.007	N=5,453
	[.802, .836]	[.916, 1.014]	[1.061, 1.116]	[.992, 1.101]	[.877, .905]	[.972, 1.043]	
Morocco	.584***	.975	.811***	1.131***	.473***	1.104**	N=14,022
	[.567, .601]	[.897, 1.061]	[.78, .843]	[1.035, 1.237]	[.455, .492]	[1.005, 1.212]	
Pakistan	.512***	.98	.698***	1.032*	.357***	1.011	N=70,810
	[.5, .523]	[.949, 1.012]	[.678, .718]	[.994, 1.072]	[.346, .368]	[.977, 1.047]	
Peru	.76***	1.007	.9975	1.003	.758***	1.009	N=82,853
	[.753, .767]	[.988, 1.026]	[.987, 1.008]	[.981, 1.024]	[.751, .765]	[.991, 1.028]	
Philippines	.768***	1.011	1.061***	.981*	.814***	.992	N=39,192
	[.759, .776]	[.99, 1.032]	[1.044, 1.077]	[.96, 1.004]	[.805, .824]	[.972, 1.012]	
South Africa	.912***	1.026	.977**	.972	.891***	.9969	N=7,000
	[.899, .924]	[.991, 1.062]	[.958, .9967]	[.931, 1.014]	[.878, .904]	[.959, 1.036]	
Tajikistan	.87***	.981	.869***	.992	.756***	.973	N=16,528
	[.859, .88]	[.955, 1.009]	[.85, .889]	[.96, 1.024]	[.741, .771]	[.941, 1.006]	
Türkiye	.729***	.971*	.867***	1.035*	.632***	1.005	N=17,604
	[.716, .743]	[.938, 1.005]	[.844, .891]	[.9953, 1.077]	[.617, .648]	[.969, 1.044]	

Notes: *P<0.1; **P<0.05; ***P<0.01. Rate ratios and a constant from Poisson regression models are shown, in addition to two combinations obtained using post estimation. Each row of estimates was obtained from a separate model. All models included a baseline term for being female and baseline terms and interactions with being female for washer ownership, fridge ownership, TV ownership, having flush toilet, a wealth index z-score, number of household members, number of household members under age five, age, highest education level of a male in household, and highest education level of a female in household, as well as adjusting for neighborhood. Except the baseline term for female, all independent variables, including fixed effects, were centered around a country-specific weighted mean: Therefore, the 'Constant' column shows school attendance for males with the mean on all other independent variables. The column labelled 'Piped water' shows differences in school attendance for males with piped water at home. The column labelled 'Interaction' shows interaction terms for females and piped water. The column labelled 'Constant×Female' shows school attendance for females with the mean on all other independent variables, obtained using post estimation. The column labelled 'Interaction×Piped water' shows difference in school attendance for girls with piped water at home, obtained using post estimation. Estimates were weighted using sampling weights, rescaled to sum up to one for the final sample from each survey. 95% confidence intervals adjusted for clustering at the level of primary sampling units are shown in brackets below the point estimates. Pooled models were further rescaled such that each country contributed equally to the estimates.

Table S13. Results from Poisson regression models of school attendance on electricity

	Constant	Electricity	Female	Interaction	Constant× Female	Interaction× Electricity	
Pooled	.799***	1.021	.993	.978	.794***	.999	N=1,614,215
	[.794, .805]	[.984, 1.059]	[.985, 1.002]	[.945, 1.014]	[.788, .799]	[.961, 1.039]	
Albania	.808***		1		.808***	NA	N=13,749
	[.793, .823]		[.976, 1.025]		[.794, .822]		
Armenia	.79***	2.274	1.077***	.587	.851***	1.336	N=10,925
	[.774, .806]	[.44, 11.75]	[1.05, 1.105]	[.0742, 4.649]	[.838, .863]	[.615, 2.899]	
Azerbaijan	.881***	1.362	.928***	.752	.817***	1.024	N=6,203
	[.864, .897]	[.671, 2.763]	[.9, .956]	[.335, 1.688]	[.8, .835]	[.909, 1.153]	
Colombia	.752***	1.025	1.034***	1.012	.778***	1.037	N=61,245
	[.744, .761]	[.949, 1.106]	[1.019, 1.05]	[.93, 1.101]	[.77, .786]	[.966, 1.113]	
Egypt	.758***	1.025	.909***	1.038	.689***	1.064	N=102,079
	[.752, .763]	[.943, 1.115]	[.9, .919]	[.901, 1.195]	[.682, .695]	[.934, 1.212]	
Gabon	.89***	1.009	1.001	.979	.891***	.988	N=8,461
	[.875, .906]	[.94, 1.083]	[.977, 1.026]	[.901, 1.065]	[.878, .904]	[.913, 1.069]	
Guatemala	.666***	1.026	.872***	.951	.581***	.976	N=24,445
	[.652, .68]	[.966, 1.09]	[.849, .896]	[.875, 1.034]	[.567, .596]	[.908, 1.049]	
Guyana	.69***	.967	1.019	1.088**	.704***	1.051	N=6,787
	[.666, .716]	[.9, 1.038]	[.975, 1.066]	[1.004, 1.179]	[.682, .726]	[.978, 1.131]	
India	.756***	.9959	.958***	1.024***	.724***	1.02***	N=1,075,968
	[.754, .758]	[.987, 1.005]	[.955, .961]	[1.013, 1.035]	[.722, .726]	[1.01, 1.03]	
Indonesia	.771***	1.023	1.031***	.97	.794***	.992	N=35,679
	[.762, .78]	[.951, 1.101]	[1.014, 1.047]	[.889, 1.058]	[.785, .804]	[.941, 1.046]	
Kyrgyz Republic	.881***	1.048	1.025**	.824*	.903***	.864	N=6,551
	[.866, .897]	[.936, 1.174]	[1.004, 1.046]	[.663, 1.025]	[.89, .917]	[.709, 1.053]	
Moldova	.819***	1.378	1.087***	1.029	.89***	1.418	N=5,455
	[.802, .836]	[.88, 2.159]	[1.06, 1.115]	[.532, 1.988]	[.877, .904]	[.891, 2.257]	
Morocco	.584***	1.025	.811***	.912	.474***	.935	N=14,014
	[.567, .601]	[.923, 1.139]	[.781, .843]	[.799, 1.041]	[.456, .492]	[.84, 1.041]	
Pakistan	.509***	1.077	.692***	1.239**	.352***	1.334***	N=70,834
	[.497, .521]	[.925, 1.254]	[.672, .713]	[1.003, 1.53]	[.342, .363]	[1.12, 1.59]	
Peru	.76***	1.012	.9974	1.011	.758***	1.023*	N=82,853
	[.753, .767]	[.984, 1.041]	[.987, 1.008]	[.98, 1.043]	[.751, .765]	[.9979, 1.05]	
Philippines	.767***	1.061***	1.061***	.972	.814***	1.031*	N=39,196
	[.759, .776]	[1.017, 1.106]	[1.045, 1.078]	[.924, 1.021]	[.805, .824]	[.9954, 1.067]	
South Africa	.911***	1.029	.977**	.929	.891***	.956	N=7,000
	[.899, .924]	[.955, 1.109]	[.958, .9969]	[.845, 1.022]	[.878, .904]	[.879, 1.04]	
Tajikistan	.87***	1.033	.869***	1.064	.756***	1.099	N=16,542
	[.859, .88]	[.914, 1.167]	[.85, .889]	[.867, 1.306]	[.741, .771]	[.946, 1.277]	
Türkiye	.726***		.874***		.634***		N=26,229
	[.714, .737]		[.853, .894]		[.621, .647]		

Notes: *P<0.1; **P<0.05; ***P<0.01. Rate ratios and a constant from Poisson regression models are shown, in addition to two combinations obtained using post estimation. Each row of estimates was obtained from a separate model. All models included a baseline term for being female and baseline terms and interactions with being female for washer ownership, fridge ownership, TV ownership, having flush toilet, a wealth index z-score, number of household members, number of household members under age five, age, highest education level of a male in household, and highest education level of a female in household, as well as adjusting for neighborhood. Except the baseline term for female, all independent variables, including fixed effects, were centered around a country-specific weighted mean: Therefore, the 'Constant' column shows school attendance for males with the mean on all other independent variables. The column labelled 'Electricity' shows differences in school attendance for males with electricity at home. The column labelled 'Interaction' shows interaction terms for females and electricity. The column labelled 'Constant×Female' shows school attendance for females with the mean on all other independent variables, obtained using post estimation. The column labelled 'Interaction×Electricity' shows difference in school attendance for girls with electricity at home, obtained using post estimation. Estimates were weighted using sampling weights, rescaled to sum up to one for the final sample from each survey. 95% confidence intervals adjusted for clustering at the level of primary sampling units are shown in brackets below the point estimates. Pooled models were further rescaled such that each country contributed equally to the estimates.

Table S14. Results from Poisson regression models of school attendance on car ownership

	Constant	Car	Female	Interaction	Constant× Female	Interaction× Car	
Pooled	.799***	1.009*	.994	.987*	.794***	.9953	N=1,613,855
	[.793, .805]	[.9988, 1.019]	[.986, 1.002]	[.973, 1.001]	[.788, .799]	[.984, 1.007]	
Albania	.808***	1.006	1	1.012	.808***	1.018	N=13,749
	[.793, .823]	[.976, 1.038]	[.976, 1.025]	[.973, 1.053]	[.794, .822]	[.987, 1.051]	
Armenia	.789***	1.003	1.078***	1.005	.851***	1.008	N=10,892
	[.773, .805]	[.976, 1.031]	[1.051, 1.106]	[.967, 1.044]	[.838, .863]	[.981, 1.036]	
Azerbaijan	.881***	.9978	.927***	.993	.817***	.99	N=6,202
	[.865, .898]	[.971, 1.026]	[.899, .956]	[.942, 1.046]	[.8, .834]	[.944, 1.039]	
Colombia	.752***	.978	1.034***	1.02	.778***	.9975	N=61,245
	[.744, .761]	[.95, 1.007]	[1.019, 1.05]	[.98, 1.061]	[.77, .786]	[.971, 1.025]	
Egypt	.757***	.986	.91***	.992	.689***	.978**	N=101,994
	[.752, .763]	[.967, 1.005]	[.9, .919]	[.967, 1.016]	[.682, .696]	[.959, .9972]	
Gabon	.89***	1.004	1.001	.989	.891***	.993	N=8,422
	[.875, .905]	[.928, 1.086]	[.977, 1.026]	[.922, 1.061]	[.877, .905]	[.939, 1.049]	
Guatemala	.666***	1.009	.872***	.961	.581***	.969	N=24,450
	[.652, .68]	[.971, 1.048]	[.848, .896]	[.915, 1.009]	[.566, .595]	[.926, 1.015]	
Guyana	.691***	.961	1.02	1.034	.704***	.993	N=6,771
	[.666, .716]	[.909, 1.015]	[.975, 1.067]	[.952, 1.124]	[.683, .727]	[.931, 1.059]	
India	.756***	.992**	.958***	.986***	.724***	.978***	N=1,075,968
	[.754, .758]	[.985, .9992]	[.955, .961]	[.976, .9955]	[.722, .726]	[.97, .986]	
Indonesia	.771***	.972**	1.03***	1.003	.794***	.975**	N=35,675
	[.762, .78]	[.949, .9962]	[1.014, 1.047]	[.971, 1.036]	[.785, .804]	[.954, .9975]	
Kyrgyz Republic	.881***	.976*	1.025**	.991	.903***	.967**	N=6,533
	[.866, .897]	[.952, 1]	[1.004, 1.046]	[.953, 1.03]	[.89, .916]	[.937, .9978]	
Moldova	.819***	1.021	1.088***	1.003	.891***	1.024	N=5,441
	[.802, .836]	[.988, 1.054]	[1.061, 1.116]	[.962, 1.046]	[.877, .905]	[.994, 1.055]	
Morocco	.584***	1.053**	.812***	.887***	.474***	.933**	N=14,019
	[.567, .601]	[1.004, 1.103]	[.781, .843]	[.826, .953]	[.456, .492]	[.882, .988]	
Pakistan	.511***	1.019	.698***	.95**	.357***	.968	N=70,741
	[.5, .523]	[.979, 1.061]	[.678, .719]	[.907, .9957]	[.346, .368]	[.929, 1.009]	
Peru	.76***	1.026**	.9976	.975*	.758***	1.001	N=82,853
	[.753, .767]	[1.003, 1.049]	[.987, 1.008]	[.947, 1.005]	[.751, .765]	[.98, 1.021]	
Philippines	.768***	.993	1.061***	.98	.814***	.973	N=39,179
	[.759, .776]	[.956, 1.03]	[1.044, 1.077]	[.936, 1.026]	[.805, .824]	[.937, 1.01]	
South Africa	.911***	1.031**	.978**	.988	.891***	1.018	N=7,000
	[.899, .924]	[1.002, 1.06]	[.959, .9974]	[.947, 1.031]	[.878, .904]	[.984, 1.053]	
Tajikistan	.87***	1.007	.869***	.984	.756***	.991	N=16,532
	[.86, .88]	[.989, 1.025]	[.85, .889]	[.958, 1.011]	[.741, .772]	[.969, 1.014]	
Türkiye	.726***	1.02	.873***	.994	.634***	1.013	N=26,189
	[.715, .738]	[.993, 1.047]	[.853, .894]	[.959, 1.029]	[.621, .647]	[.985, 1.042]	

Notes: *P<0.1; **P<0.05; ***P<0.01. Rate ratios and a constant from Poisson regression models are shown, in addition to two combinations obtained using post estimation. Each row of estimates was obtained from a separate model. All models included a baseline term for being female and baseline terms and interactions with being female for washer ownership, fridge ownership, TV ownership, having flush toilet, a wealth index z-score, number of household members, number of household members under age five, age, highest education level of a male in household, and highest education level of a female in household, as well as adjusting for neighborhood. Except the baseline term for female, all independent variables, including fixed effects, were centered around a country-specific weighted mean: Therefore, the 'Constant' column shows school attendance for males with the mean on all other independent variables. The column labelled 'Car' shows differences in school attendance for males with car at home. The column labelled 'Interaction' shows interaction terms for females and car. The column labelled 'Constant×Female' shows school attendance for females with the mean on all other independent variables, obtained using post estimation. The column labelled 'Interaction×Car' shows difference in school attendance for girls with car at home, obtained using post estimation. Estimates were weighted using sampling weights, rescaled to sum up to one for the final sample from each survey. 95% confidence intervals adjusted for clustering at the level of primary sampling units are shown in brackets below the point estimates. Pooled models were further rescaled such that each country contributed equally to the estimates.

Table S15. Results from Poisson regression models of school attendance on scooter ownership

	Constant	Scooter	Female	Interaction	Constant× Female	Interaction× Scooter	
Pooled	.799***	1.009	.994	.978**	.794***	.987*	N=1,613,853
	[.793, .805]	[.994, 1.025]	[.986, 1.002]	[.959, .9976]	[.788, .799]	[.973, 1.002]	
Albania	.808***	1.014	1	.967	.808***	.981	N=13,749
	[.793, .823]	[.975, 1.055]	[.976, 1.025]	[.92, 1.017]	[.794, .822]	[.945, 1.019]	
Armenia	.789***	1.006	1.078***	.967	.851***	.973	N=10,889
	[.773, .806]	[.848, 1.194]	[1.051, 1.106]	[.739, 1.266]	[.839, .864]	[.776, 1.221]	
Azerbaijan	.881***	1.047	.927***	.9	.817***	.942	N=6,198
	[.865, .898]	[.976, 1.123]	[.899, .956]	[.779, 1.04]	[.8, .834]	[.821, 1.082]	
Colombia	.753***	.984	1.034***	1.009	.778***	.993	N=61,245
	[.744, .761]	[.965, 1.004]	[1.018, 1.05]	[.983, 1.035]	[.77, .786]	[.975, 1.012]	
Egypt	.758***	.988	.91***	1.036**	.689***	1.023*	N=102,016
	[.752, .763]	[.965, 1.012]	[.9, .919]	[1.002, 1.071]	[.682, .696]	[.9972, 1.05]	
Gabon	.89***	.9	1.001	1.002	.891***	.902	N=8,414
	[.874, .905]	[.618, 1.31]	[.977, 1.026]	[.689, 1.456]	[.877, .904]	[.789, 1.03]	
Guatemala	.666***	1.038**	.872***	.979	.581***	1.016	N=24,451
	[.652, .68]	[1, 1.077]	[.849, .896]	[.934, 1.026]	[.566, .595]	[.98, 1.054]	
Guyana	.691***	.971	1.021	.971	.705***	.943*	N=6,778
	[.666, .716]	[.908, 1.037]	[.976, 1.067]	[.891, 1.058]	[.683, .727]	[.887, 1.002]	
India	.756***	1.004*	.958***	.967***	.724***	.97***	N=1,075,968
	[.754, .758]	[.9996, 1.009]	[.955, .961]	[.961, .972]	[.722, .726]	[.966, .975]	
Indonesia	.771***	1.023*	1.031***	.981	.795***	1.003	N=35,670
	[.762, .78]	[.9966, 1.049]	[1.014, 1.047]	[.947, 1.016]	[.785, .804]	[.976, 1.031]	
Kyrgyz Republic	.881***	.86**	1.025**	1.081	.903***	.929	N=6,525
	[.866, .897]	[.754, .979]	[1.004, 1.046]	[.923, 1.267]	[.89, .916]	[.844, 1.023]	
Moldova	.818***	1.056**	1.089***	.963	.891***	1.018	N=5,447
	[.801, .836]	[1.011, 1.104]	[1.062, 1.117]	[.909, 1.021]	[.877, .905]	[.98, 1.057]	
Morocco	.584***	1.026	.812***	.961	.474***	.985	N=14,019
	[.567, .601]	[.976, 1.077]	[.781, .844]	[.894, 1.032]	[.456, .493]	[.934, 1.039]	
Pakistan	.511***	1.039***	.699***	.956**	.357***	.994	N=70,737
	[.5, .523]	[1.011, 1.068]	[.679, .72]	[.923, .99]	[.346, .369]	[.961, 1.027]	
Peru	.76***	.994	.9974	.989	.758***	.983*	N=82,853
	[.754, .767]	[.976, 1.012]	[.987, 1.008]	[.967, 1.012]	[.751, .765]	[.966, 1]	
Philippines	.767***	.995	1.061***	.99	.814***	.984*	N=39,177
	[.758, .776]	[.974, 1.015]	[1.044, 1.077]	[.967, 1.013]	[.804, .824]	[.967, 1.002]	
South Africa	.912***	1.02	.977**	.96	.891***	.979	N=7,000
	[.899, .924]	[.953, 1.091]	[.958, .9968]	[.852, 1.081]	[.878, .904]	[.891, 1.075]	
Tajikistan	.87***	.929**	.869***	1.077**	.756***	1	N=16,522
	[.86, .881]	[.876, .985]	[.849, .889]	[1.003, 1.156]	[.741, .771]	[.936, 1.068]	
Türkiye	.726***	1	.873***	1.024	.634***	1.024	N=26,195
	[.714, .737]	[.961, 1.041]	[.853, .894]	[.963, 1.089]	[.621, .647]	[.973, 1.079]	

Notes: *P<0.1; **P<0.05; ***P<0.01. Rate ratios and a constant from Poisson regression models are shown, in addition to two combinations obtained using post estimation. Each row of estimates was obtained from a separate model. All models included a baseline term for being female and baseline terms and interactions with being female for washer ownership, fridge ownership, TV ownership, having flush toilet, a wealth index z-score, number of household members, number of household members under age five, age, highest education level of a male in household, and highest education level of a female in household, as well as adjusting for neighborhood. Except the baseline term for female, all independent variables, including fixed effects, were centered around a country-specific weighted mean: Therefore, the 'Constant' column shows school attendance for males with the mean on all other independent variables. The column labelled 'Scooter' shows differences in school attendance for males with scooter at home. The column labelled 'Interaction' shows interaction terms for females and scooter. The column labelled 'Constant×Female' shows school attendance for females with the mean on all other independent variables, obtained using post estimation. The column labelled 'Interaction×Scooter' shows difference in school attendance for girls with scooter at home, obtained using post estimation. Estimates were weighted using sampling weights, rescaled to sum up to one for the final sample from each survey. 95% confidence intervals adjusted for clustering at the level of primary sampling units are shown in brackets below the point estimates. Pooled models were further rescaled such that each country contributed equally to the estimates.

Table S16. Results from Poisson regression models of school attendance on clean cooking

	Constant	Clean cooking	Female	Interaction	Constant×Female	Interaction×Clean cooking	
Pooled	.805***	.9987	.9975	1.013	.803***	1.012	N=1,532,127
	[.799, .811]	[.983, 1.015]	[.989, 1.006]	[.995, 1.032]	[.797, .808]	[.9952, 1.028]	
Albania	.808***	.993	1	1.027	.808***	1.019	N=13,749
	[.793, .823]	[.955, 1.032]	[.976, 1.025]	[.982, 1.073]	[.794, .822]	[.98, 1.061]	
Armenia	.79***	1.117**	1.077***	.967	.851***	1.08	N=10,924
	[.774, .806]	[1.002, 1.245]	[1.05, 1.105]	[.855, 1.094]	[.838, .863]	[.984, 1.185]	
Azerbaijan	.881***	.98	.927***	1.022	.817***	1.001	N=6,203
	[.865, .898]	[.906, 1.059]	[.899, .956]	[.938, 1.112]	[.8, .834]	[.921, 1.087]	
Colombia	.752***	.97*	1.034***	1.01	.778***	.98	N=61,245
	[.744, .761]	[.936, 1.005]	[1.019, 1.05]	[.966, 1.056]	[.77, .786]	[.944, 1.016]	
Egypt	.741***	.94	.876***	1.274***	.649***	1.197**	N=60,107
	[.734, .748]	[.87, 1.015]	[.863, .889]	[1.077, 1.507]	[.64, .658]	[1.023, 1.4]	
Gabon	.89***	1.014	1.001	.983	.891***	.9973	N=8,455
	[.875, .906]	[.945, 1.089]	[.977, 1.025]	[.894, 1.082]	[.878, .904]	[.926, 1.075]	
Guatemala	.666***	.94***	.872***	1.056*	.581***	.993	N=24,450
	[.652, .68]	[.901, .98]	[.848, .896]	[.9988, 1.117]	[.567, .595]	[.947, 1.041]	
Guyana	.69***	.967	1.018	1.047	.702***	1.013	N=6,796
	[.666, .716]	[.912, 1.026]	[.973, 1.064]	[.981, 1.118]	[.681, .725]	[.958, 1.071]	
India	.756***	.981***	.958***	1.032***	.724***	1.012***	N=1,075,968
	[.754, .758]	[.976, .986]	[.955, .961]	[1.025, 1.039]	[.722, .726]	[1.007, 1.018]	
Indonesia	.771***	.971*	1.031***	1.001	.794***	.973**	N=35,685
	[.762, .78]	[.943, 1]	[1.014, 1.047]	[.968, 1.036]	[.785, .804]	[.947, .9992]	
Kyrgyz Republic	.881***	.999	1.025**	.994	.904***	.993	N=6,549
	[.866, .897]	[.96, 1.04]	[1.004, 1.046]	[.957, 1.032]	[.891, .916]	[.956, 1.031]	
Moldova	.819***	1.014	1.087***	1.018	.891***	1.032	N=5,457
	[.802, .836]	[.94, 1.093]	[1.06, 1.115]	[.929, 1.116]	[.877, .905]	[.962, 1.106]	
Morocco	.583***	.946	.811***	.954	.473***	.903	N=13,962
	[.566, .6]	[.854, 1.048]	[.78, .843]	[.827, 1.101]	[.455, .491]	[.788, 1.034]	
Pakistan	.511***	.974	.699***	1.081***	.357***	1.053**	N=70,824
	[.5, .523]	[.933, 1.016]	[.679, .719]	[1.032, 1.133]	[.346, .368]	[1.003, 1.105]	
Peru	.76***	.996	.9976	1.011	.758***	1.006	N=82,853
	[.753, .767]	[.975, 1.017]	[.987, 1.008]	[.984, 1.037]	[.751, .765]	[.986, 1.028]	
Philippines	.828***	1.018	1.062***	.987	.879***	1.004	N=25,356
	[.817, .839]	[.975, 1.062]	[1.043, 1.082]	[.941, 1.035]	[.869, .89]	[.976, 1.034]	
South Africa	.911***	.99	.977**	1.028	.891***	1.018	N=7,000
	[.899, .924]	[.955, 1.027]	[.958, .9968]	[.98, 1.079]	[.878, .904]	[.976, 1.062]	
Tajikistan	.87***	.976*	.869***	1.03	.756***	1.005	N=16,544
	[.86, .88]	[.949, 1.003]	[.85, .889]	[.987, 1.075]	[.741, .771]	[.97, 1.042]	

Notes: *P<0.1; **P<0.05; ***P<0.01. Rate ratios and a constant from Poisson regression models are shown, in addition to two combinations obtained using post estimation. Each row of estimates was obtained from a separate model. All models included a baseline term for being female and baseline terms and interactions with being female for washer ownership, fridge ownership, TV ownership, having flush toilet, a wealth index z-score, number of household members, number of household members under age five, age, highest education level of a male in household, and highest education level of a female in household, as well as adjusting for neighborhood. Except the baseline term for female, all independent variables, including fixed effects, were centered around a country-specific weighted mean: Therefore, the 'Constant' column shows school attendance for males with the mean on all other independent variables. The column labelled 'Clean cooking' shows differences in school attendance for males with clean cooking at home. The column labelled 'Interaction' shows interaction terms for females and clean cooking. The column labelled 'Constant×Female' shows school attendance for females with the mean on all other independent variables, obtained using post estimation. The column labelled 'Interaction×Clean cooking' shows difference in school attendance for girls with clean cooking at home, obtained using post estimation. Estimates were weighted using sampling weights, rescaled to sum up to one for the final sample from each survey. 95% confidence intervals adjusted for clustering at the level of primary sampling units are shown in brackets below the point estimates. Pooled models were further rescaled such that each country contributed equally to the estimates.

Table S17. Results from Poisson regression models of school attendance on flush toilet

	Constant	Flush toilet	Female	Interaction	Constant× Female	Interaction× Flush toilet	
Pooled	.799***	1.012	.993	1.007	.794***	1.018**	N=1,614,264
	[.794, .805]	[.995, 1.029]	[.985, 1.001]	[.989, 1.025]	[.788, .799]	[1.002, 1.035]	
Albania	.808***	1.007	1	1.109*	.808***	1.118*	N=13,749
	[.793, .823]	[.925, 1.098]	[.976, 1.025]	[.981, 1.254]	[.794, .822]	[.995, 1.255]	
Armenia	.79***	.98	1.077***	1.054*	.851***	1.033	N=10,925
	[.774, .806]	[.939, 1.023]	[1.05, 1.105]	[.9975, 1.114]	[.839, .863]	[.99, 1.078]	
Azerbaijan	.881***	.958	.927***	1.019	.817***	.976	N=6,203
	[.865, .898]	[.884, 1.04]	[.899, .956]	[.947, 1.096]	[.8, .834]	[.88, 1.084]	
Colombia	.752***	1.002	1.034***	.9963	.778***	.9984	N=61,245
	[.744, .761]	[.964, 1.042]	[1.019, 1.05]	[.954, 1.04]	[.77, .786]	[.962, 1.036]	
Egypt	.758***	1.001	.909***	1.289***	.689***	1.291***	N=102,079
	[.752, .763]	[.954, 1.05]	[.9, .919]	[1.185, 1.403]	[.682, .695]	[1.197, 1.392]	
Gabon	.89***	.9983	1.001	1.004	.891***	1.003	N=8,462
	[.875, .906]	[.949, 1.051]	[.977, 1.025]	[.933, 1.081]	[.878, .905]	[.942, 1.067]	
Guatemala	.666***	.992	.872***	1.072**	.581***	1.063**	N=24,453
	[.652, .68]	[.955, 1.031]	[.848, .896]	[1.015, 1.132]	[.566, .595]	[1.014, 1.115]	
Guyana	.691***	1.053*	1.019	.944*	.704***	.995	N=6,801
	[.666, .716]	[.9993, 1.11]	[.974, 1.066]	[.885, 1.008]	[.682, .726]	[.947, 1.045]	
India	.756***	.993***	.958***	1.022***	.724***	1.015***	N=1,075,968
	[.754, .758]	[.988, .9981]	[.955, .961]	[1.016, 1.029]	[.722, .726]	[1.01, 1.021]	
Indonesia	.771***	.948***	1.03***	1.049**	.794***	.994	N=35,687
	[.762, .78]	[.916, .98]	[1.014, 1.047]	[1.008, 1.092]	[.785, .804]	[.962, 1.027]	
Kyrgyz Republic	.881***	.975	1.025**	1.068	.903***	1.042	N=6,551
	[.866, .897]	[.9, 1.057]	[1.004, 1.046]	[.984, 1.16]	[.89, .917]	[.977, 1.112]	
Moldova	.819***	1.047	1.088***	.963	.891***	1.008	N=5,457
	[.802, .836]	[.98, 1.118]	[1.061, 1.115]	[.89, 1.042]	[.877, .905]	[.955, 1.065]	
Morocco	.584***	1.041	.812***	.971	.474***	1.011	N=14,022
	[.567, .601]	[.95, 1.141]	[.781, .844]	[.866, 1.088]	[.456, .493]	[.915, 1.117]	
Pakistan	.511***	.9967	.698***	1.146***	.357***	1.142***	N=70,840
	[.5, .523]	[.95, 1.046]	[.678, .719]	[1.07, 1.228]	[.346, .368]	[1.069, 1.22]	
Peru	.76***	.985	.9975	1.008	.758***	.992	N=82,853
	[.754, .767]	[.965, 1.005]	[.987, 1.008]	[.986, 1.03]	[.751, .765]	[.973, 1.012]	
Philippines	.768***	1.04**	1.061***	.947***	.814***	.985	N=39,196
	[.759, .777]	[1.003, 1.079]	[1.044, 1.077]	[.909, .986]	[.805, .824]	[.953, 1.017]	
South Africa	.912***	.961	.977**	.988	.891***	.95	N=7,000
	[.899, .924]	[.903, 1.023]	[.958, .9968]	[.936, 1.043]	[.878, .904]	[.891, 1.012]	
Tajikistan	.87***	1.009	.869***	.982	.756***	.991	N=16,544
	[.859, .88]	[.962, 1.058]	[.85, .889]	[.936, 1.031]	[.741, .771]	[.949, 1.034]	
Türkiye	.726***	.972	.874***	1.089***	.634***	1.058**	N=26,229
	[.714, .737]	[.933, 1.013]	[.853, .894]	[1.04, 1.14]	[.621, .647]	[1.009, 1.11]	

Notes: *P<0.1; **P<0.05; ***P<0.01. Rate ratios and a constant from Poisson regression models are shown, in addition to two combinations obtained using post estimation. Each row of estimates was obtained from a separate model. All models included a baseline term for being female and baseline terms and interactions with being female for washer ownership, fridge ownership, TV ownership, having flush toilet, a wealth index z-score, number of household members, number of household members under age five, age, highest education level of a male in household, and highest education level of a female in household, as well as adjusting for neighborhood. Except the baseline term for female, all independent variables, including fixed effects, were centered around a country-specific weighted mean: Therefore, the 'Constant' column shows school attendance for males with the mean on all other independent variables. The column labelled 'Flush toilet' shows differences in school attendance for males with flush toilet at home. The column labelled 'Interaction' shows interaction terms for females and flush toilet. The column labelled 'Constant×Female' shows school attendance for females with the mean on all other independent variables, obtained using post estimation. The column labelled 'Interaction×Flush toilet' shows difference in school attendance for girls with flush toilet at home, obtained using post estimation. Estimates were weighted using sampling weights, rescaled to sum up to one for the final sample from each survey. 95% confidence intervals adjusted for clustering at the level of primary sampling units are shown in brackets below the point estimates. Pooled models were further rescaled such that each country contributed equally to the estimates.

Table S18. Results from Poisson regression models of school attendance on TV ownership

	Constant	TV	Female	Interaction	Constant× Female	Interaction× TV	
Pooled	.799***	1.028***	.993	1.005	.794***	1.034***	N=1,614,264
	[.794, .805]	[1.009, 1.048]	[.985, 1.001]	[.982, 1.029]	[.788, .799]	[1.014, 1.054]	
Albania	.808***	.943	1	1.121	.808***	1.057	N=13,749
	[.793, .823]	[.844, 1.053]	[.976, 1.025]	[.949, 1.324]	[.794, .822]	[.934, 1.197]	
Armenia	.79***	1.071	1.077***	1.019	.851***	1.092**	N=10,925
	[.774, .806]	[.976, 1.176]	[1.05, 1.105]	[.922, 1.127]	[.839, .863]	[1.008, 1.184]	
Azerbaijan	.881***	.95	.927***	1.098*	.817***	1.043	N=6,203
	[.865, .898]	[.892, 1.012]	[.899, .956]	[.984, 1.225]	[.8, .834]	[.95, 1.146]	
Colombia	.752***	1.04**	1.034***	.977	.778***	1.016	N=61,245
	[.744, .761]	[1.003, 1.079]	[1.019, 1.05]	[.934, 1.021]	[.77, .786]	[.982, 1.051]	
Egypt	.758***	1	.909***	1.024	.689***	1.024	N=102,079
	[.752, .763]	[.966, 1.036]	[.9, .919]	[.971, 1.08]	[.682, .695]	[.981, 1.069]	
Gabon	.89***	.987	1.001	1.044	.891***	1.03	N=8,462
	[.875, .906]	[.922, 1.057]	[.977, 1.025]	[.952, 1.144]	[.878, .905]	[.963, 1.101]	
Guatemala	.666***	.9956	.872***	1.054*	.581***	1.049*	N=24,453
	[.652, .68]	[.957, 1.036]	[.848, .896]	[.994, 1.117]	[.566, .595]	[.9974, 1.104]	
Guyana	.691***	1.015	1.019	.987	.704***	1.001	N=6,801
	[.666, .716]	[.942, 1.094]	[.974, 1.066]	[.892, 1.091]	[.682, .726]	[.927, 1.081]	
India	.756***	.981***	.958***	1.01***	.724***	.991***	N=1,075,968
	[.754, .758]	[.976, .987]	[.955, .961]	[1.003, 1.017]	[.722, .726]	[.985, .9972]	
Indonesia	.771***	.977	1.03***	1.043	.794***	1.018	N=35,687
	[.762, .78]	[.937, 1.017]	[1.014, 1.047]	[.986, 1.103]	[.785, .804]	[.978, 1.06]	
Kyrgyz Republic	.881***	.992	1.025**	.991	.903***	.983	N=6,551
	[.866, .897]	[.902, 1.092]	[1.004, 1.046]	[.793, 1.237]	[.89, .917]	[.791, 1.221]	
Moldova	.819***	1.008	1.088***	1.025	.891***	1.033	N=5,457
	[.802, .836]	[.953, 1.066]	[1.061, 1.115]	[.955, 1.1]	[.877, .905]	[.986, 1.082]	
Morocco	.584***	1.077**	.812***	.942	.474***	1.015	N=14,022
	[.567, .601]	[1.01, 1.148]	[.781, .844]	[.862, 1.03]	[.456, .493]	[.937, 1.098]	
Pakistan	.511***	.963**	.698***	1.107***	.357***	1.066***	N=70,840
	[.5, .523]	[.929, .9977]	[.678, .719]	[1.052, 1.165]	[.346, .368]	[1.02, 1.113]	
Peru	.76***	.989	.9975	1.008	.758***	.9967	N=82,853
	[.754, .767]	[.971, 1.008]	[.987, 1.008]	[.984, 1.032]	[.751, .765]	[.979, 1.015]	
Philippines	.768***	1.017	1.061***	.981	.814***	.9969	N=39,196
	[.759, .777]	[.978, 1.057]	[1.044, 1.077]	[.937, 1.026]	[.805, .824]	[.961, 1.034]	
South Africa	.912***	1.016	.977**	1	.891***	1.016	N=7,000
	[.899, .924]	[.979, 1.055]	[.958, .9968]	[.941, 1.063]	[.878, .904]	[.964, 1.072]	
Tajikistan	.87***	.986	.869***	.959	.756***	.946	N=16,544
	[.859, .88]	[.933, 1.043]	[.85, .889]	[.872, 1.055]	[.741, .771]	[.869, 1.03]	
Türkiye	.726***	.864***	.874***	.97	.634***	.838***	N=26,229
	[.714, .737]	[.803, .929]	[.853, .894]	[.858, 1.097]	[.621, .647]	[.753, .932]	

Notes: *P<0.1; **P<0.05; ***P<0.01. Rate ratios and a constant from Poisson regression models are shown, in addition to two combinations obtained using post estimation. Each row of estimates was obtained from a separate model. All models included a baseline term for being female and baseline terms and interactions with being female for washer ownership, fridge ownership, TV ownership, having flush toilet, a wealth index z-score, number of household members, number of household members under age five, age, highest education level of a male in household, and highest education level of a female in household, as well as adjusting for neighborhood. Except the baseline term for female, all independent variables, including fixed effects, were centered around a country-specific weighted mean: Therefore, the 'Constant' column shows school attendance for males with the mean on all other independent variables. The column labelled 'TV' shows differences in school attendance for males with TV at home. The column labelled 'Interaction' shows interaction terms for females and TV. The column labelled 'Constant×Female' shows school attendance for females with the mean on all other independent variables, obtained using post estimation. The column labelled 'Interaction×TV' shows difference in school attendance for girls with TV at home, obtained using post estimation. Estimates were weighted using sampling weights, rescaled to sum up to one for the final sample from each survey. 95% confidence intervals adjusted for clustering at the level of primary sampling units are shown in brackets below the point estimates. Pooled models were further rescaled such that each country contributed equally to the estimates.

Table S19. Results from logistic regression models of school attendance on washer ownership

	Constant	Washer	Female	Interaction	Constant× Female	Interaction× Washer	
Pooled	10.62***	1.165***	.948*	.989	10.07***	1.152***	N=1,614,264
	[10.07, 11.21]	[1.056, 1.285]	[.89, 1.009]	[.872, 1.121]	[9.586, 10.58]	[1.041, 1.274]	R sq 0.302
Albania	14.81***	1.048	1.044	1.54**	15.47***	1.614***	N=13,749
	[12.03, 18.25]	[.744, 1.476]	[.798, 1.368]	[1.024, 2.315]	[12.66, 18.91]	[1.17, 2.226]	R sq 0.361
Armenia	44.52***	1.031	.937	1.373	41.73***	1.416*	N=10,925
	[21.36, 92.78]	[.746, 1.426]	[.423, 2.075]	[.876, 2.154]	[24.84, 70.11]	[.982, 2.042]	R sq 0.415
Azerbaijan	33.68***	1.614	.543***	.83	18.29***	1.34	N=6,203
	[23.28, 48.72]	[.821, 3.171]	[.346, .852]	[.322, 2.141]	[13.87, 24.13]	[.67, 2.678]	R sq 0.422
Colombia	7.268***	.879*	1.341***	1.383***	9.749***	1.216**	N=61,245
	[6.777, 7.794]	[.765, 1.01]	[1.208, 1.489]	[1.142, 1.675]	[8.986, 10.58]	[1.045, 1.415]	R sq 0.315
Egypt	7.601***	1.055	.756***	1.129*	5.749***	1.192***	N=102,079
	[7.254, 7.965]	[.949, 1.173]	[.712, .803]	[.992, 1.286]	[5.485, 6.026]	[1.078, 1.317]	R sq 0.324
Gabon	21.12***	.572	1.756*	2.684	37.08***	1.534	N=8,462
	[14.89, 29.95]	[.176, 1.859]	[.968, 3.185]	[.252, 28.56]	[23.43, 58.68]	[.17, 13.82]	R sq 0.299
Guatemala	4.493***	1.077	.683***	.879	3.07***	.947	N=24,453
	[4.103, 4.92]	[.765, 1.516]	[.612, .763]	[.565, 1.367]	[2.809, 3.357]	[.669, 1.34]	R sq 0.337
Guyana	8.948***	.857	1.334	1.304	11.94***	1.118	N=6,801
	[6.35, 12.61]	[.527, 1.393]	[.905, 1.968]	[.657, 2.589]	[8.894, 16.03]	[.658, 1.899]	R sq 0.460
India	7.532***	.943*	.944***	1.057	7.106***	.9962	N=1,075,968
	[7.405, 7.66]	[.886, 1.004]	[.924, .964]	[.978, 1.141]	[6.984, 7.231]	[.937, 1.059]	R sq 0.338
Indonesia	11.23***	.923	1.152**	1.047	12.93***	.966	N=35,687
	[10.18, 12.39]	[.765, 1.113]	[1.002, 1.324]	[.82, 1.336]	[11.54, 14.5]	[.804, 1.162]	R sq 0.383
Kyrgyz Republic	54.78***	.88	1.32	.9986	72.33***	.878	N=6,551
	[34.91, 85.96]	[.516, 1.5]	[.712, 2.447]	[.566, 1.763]	[47.06, 111.2]	[.562, 1.373]	R sq 0.458
Moldova	20.18***	1.395	3.586***	.868	72.37***	1.211	N=5,457
	[14.94, 27.26]	[.938, 2.073]	[2.068, 6.22]	[.503, 1.498]	[44.72, 117.1]	[.782, 1.876]	R sq 0.417
Morocco	2.723***	1.07	.59***	.717	1.607***	.767	N=14,022
	[2.48, 2.99]	[.726, 1.577]	[.529, .659]	[.459, 1.12]	[1.465, 1.764]	[.543, 1.084]	R sq 0.345
Pakistan	1.479***	.951	.52***	1.124	.769***	1.069	N=70,840
	[1.403, 1.56]	[.849, 1.065]	[.491, .551]	[.973, 1.299]	[.728, .813]	[.95, 1.203]	R sq 0.280
Peru	7.911***	1.034	1.009	1.084	7.983***	1.121	N=82,853
	[7.357, 8.507]	[.854, 1.253]	[.932, 1.092]	[.843, 1.393]	[7.411, 8.6]	[.922, 1.362]	R sq 0.303
Philippines	8.178***	.914	1.366***	1.163	11.17***	1.063	N=39,196
	[7.533, 8.878]	[.747, 1.118]	[1.219, 1.531]	[.881, 1.536]	[10.11, 12.34]	[.85, 1.33]	R sq 0.298
South Africa	38.94***	1.483	.958	.9957	37.31***	1.476	N=7,000
	[28.98, 52.32]	[.845, 2.602]	[.613, 1.497]	[.465, 2.131]	[26.49, 52.56]	[.828, 2.633]	R sq 0.336
Tajikistan	29.43***	1.147	.371***	.832	10.91***	.954	N=16,544
	[24.4, 35.51]	[.848, 1.552]	[.288, .477]	[.55, 1.258]	[9.094, 13.08]	[.721, 1.263]	R sq 0.420
Türkiye	11.27***	1.118	.466***	1.941***	5.249***	2.171***	N=26,229
	[9.757, 13.03]	[.889, 1.407]	[.394, .551]	[1.441, 2.616]	[4.766, 5.782]	[1.685, 2.797]	R sq 0.394

Notes: *P<0.1; **P<0.05; ***P<0.01. Odds ratios and a constant from logistic regression models are shown, in addition to two combinations obtained using post estimation. Each row of estimates was obtained from a separate model. All models included a baseline term for being female and baseline terms and interactions with being female for washer ownership, fridge ownership, TV ownership, having flush toilet, a wealth index z-score, number of household members, number of household members under age five, age, highest education level of a male in household, and highest education level of a female in household, as well as adjusting for neighborhood. Except the baseline term for female, all independent variables, including fixed effects, were centered around a country-specific weighted mean: Therefore, the 'Constant' column shows school attendance for males with the mean on all other independent variables. The column labelled 'Washer' shows differences in school attendance for males with washer at home. The column labelled 'Interaction' shows interaction terms for females and washer. The column labelled 'Constant×Female' shows school attendance for females with the mean on all other independent variables, obtained using post estimation. The column labelled 'Interaction×Washer' shows difference in school attendance for girls with washer at home, obtained using post estimation. Estimates were weighted using sampling weights, rescaled to sum up to one for the final sample from each survey. 95% confidence intervals adjusted for clustering at the level of primary sampling units are shown in brackets below the point estimates. Pooled models were further rescaled such that each country contributed equally to the estimates.

Table S20. Results from linear regression models of school attendance on washer ownership

	Constant	Washer	Female	Interaction	Constant+ Female	Interaction+ Washer	
Pooled	.826***	.0143***	-.00323	.00104	.823***	.0153***	N=1,614,264
	[.822, .83]	[.00433, .0242]	[-.00816, .00169]	[-.0118, .0139]	[.818, .827]	[.00473, .0259]	R sq 0.273
Albania	.837***	.011	-.000522	.0529**	.836***	.0639***	N=13,749
	[.825, .848]	[-.0268, .0489]	[-.0161, .015]	[.00299, .103]	[.825, .847]	[.0234, .104]	R sq 0.302
Armenia	.823***	.0111	.0508***	.0208	.874***	.0319*	N=10,925
	[.812, .834]	[-.0206, .0427]	[.0356, .0659]	[-.0235, .0651]	[.864, .884]	[-.00385, .0676]	R sq 0.320
Azerbaijan	.896***	.0167	-.0437***	-.00312	.853***	.0135	N=6,203
	[.884, .909]	[-.0145, .0478]	[-.0633, -.0242]	[-.0571, .0508]	[.84, .866]	[-.04, .0671]	R sq 0.346
Colombia	.788***	-.0131*	.0255***	.029***	.813***	.0159**	N=61,245
	[.781, .794]	[-.0284, .00212]	[.0166, .0344]	[.00869, .0493]	[.807, .819]	[.000283, .0314]	R sq 0.300
Egypt	.789***	.0119*	-.048***	.0154**	.741***	.0273***	N=102,079
	[.785, .793]	[-.0000167, .0239]	[-.0537, -.0422]	[.00041, .0304]	[.736, .746]	[.0153, .0394]	R sq 0.312
Gabon	.899***	-.0261	.00455	.0309	.904***	.00471	N=8,462
	[.886, .912]	[-.0915, .0392]	[-.0144, .0235]	[-.0454, .107]	[.893, .914]	[-.0474, .0568]	R sq 0.199
Guatemala	.716***	-.0064	-.0643***	-.00704	.652***	-.0134	N=24,453
	[.706, .727]	[-.0432, .0304]	[-.0775, -.0511]	[-.057, .0429]	[.641, .663]	[-.0531, .0262]	R sq 0.350
Guyana	.759***	-.0129	.025***	.0253	.784***	.0124	N=6,801
	[.744, .774]	[-.0558, .03]	[.0065, .0435]	[-.0333, .0839]	[.771, .797]	[-.0329, .0577]	R sq 0.459
India	.791***	-.00645**	-.0191***	.00351	.772***	-.00294	N=1,075,968
	[.79, .793]	[-.012, -.000868]	[-.021, -.0171]	[-.00334, .0104]	[.771, .774]	[-.00855, .00267]	R sq 0.328
Indonesia	.815***	-.00812	.0163***	.00477	.831***	-.00335	N=35,687
	[.808, .821]	[-.0244, .00812]	[.00768, .025]	[-.0159, .0254]	[.824, .838]	[-.0188, .0121]	R sq 0.360
Kyrgyz Republic	.899***	-.0149	.0173**	.00796	.917***	-.00691	N=6,551
	[.888, .911]	[-.0419, .0122]	[.00325, .0314]	[-.0225, .0384]	[.907, .927]	[-.0319, .0181]	R sq 0.303
Moldova	.846***	.0363*	.0599***	-.0202	.906***	.0161	N=5,457
	[.833, .859]	[-.000894, .0734]	[.0432, .0766]	[-.0666, .0263]	[.894, .917]	[-.0155, .0477]	R sq 0.300
Morocco	.647***	.00443	-.0812***	-.0424	.566***	-.038*	N=14,022
	[.634, .66]	[-.0412, .0501]	[-.0961, -.0664]	[-.0972, .0124]	[.552, .579]	[-.0831, .00712]	R sq 0.375
Pakistan	.572***	-.00888	-.114***	.0252*	.458***	.0163	N=70,840
	[.562, .581]	[-.0297, .0119]	[-.124, -.104]	[-.000431, .0508]	[.45, .466]	[-.00383, .0364]	R sq 0.323
Peru	.797***	.0018	-.0014	.00588	.796***	.00769	N=82,853
	[.792, .802]	[-.0185, .0221]	[-.00738, .00459]	[-.0199, .0316]	[.79, .801]	[-.0118, .0272]	R sq 0.298
Philippines	.798***	-.00578	.0403***	.00509	.838***	-.000698	N=39,196
	[.791, .805]	[-.0258, .0142]	[.0311, .0495]	[-.021, .0312]	[.831, .845]	[-.0211, .0198]	R sq 0.267
South Africa	.92***	.0226	-.0159**	.00804	.904***	.0306*	N=7,000
	[.909, .93]	[-.00912, .0543]	[-.0308, -.000979]	[-.0344, .0505]	[.893, .914]	[-.00255, .0638]	R sq 0.215
Tajikistan	.889***	.00129	-.0827***	-.00952	.807***	-.00823	N=16,544
	[.882, .897]	[-.0179, .0205]	[-.096, -.0694]	[-.0389, .0199]	[.796, .817]	[-.0309, .0144]	R sq 0.378
Türkiye	.774***	.00745	-.0721***	.107***	.702***	.115***	N=26,229
	[.766, .782]	[-.0218, .0367]	[-.0834, -.0609]	[.0692, .145]	[.693, .711]	[.0807, .149]	R sq 0.380

Notes: *P<0.1; **P<0.05; ***P<0.01. Regression coefficients and a constant from linear regression models are shown, in addition to two combinations obtained using post estimation. Each row of estimates was obtained from a separate model. All models included a baseline term for being female and baseline terms and interactions with being female for washer ownership, fridge ownership, TV ownership, having flush toilet, a wealth index z-score, number of household members, number of household members under age five, age, highest education level of a male in household, and highest education level of a female in household, as well as adjusting for neighborhood. Except the baseline term for female, all independent variables, including fixed effects, were centered around a country-specific weighted mean: Therefore, the 'Constant' column shows school attendance for males with the mean on all other independent variables. The column labelled 'Washer' shows differences in school attendance for males with washer at home. The column labelled 'Interaction' shows interaction terms for females and washer. The column labelled 'Constant+Female' shows school attendance for females with the mean on all other independent variables, obtained using post estimation. The column labelled 'Interaction+Washer' shows difference in school attendance for girls with washer at home, obtained using post estimation. Estimates were weighted using sampling weights, rescaled to sum up to one for the final sample from each survey. 95% confidence intervals adjusted for clustering at the level of primary sampling units are shown in brackets below the point estimates. Pooled models were further rescaled such that each country contributed equally to the estimates.

Table S21. Results from Poisson regression models of school attendance on washer ownership: excluding flush toilet, fridge ownership, and TV ownership from independent variables

	Constant	Washer	Female	Interaction	Constant× Female	Interaction× Washer	
Pooled	.799***	1.021***	.993	.9997	.794***	1.021***	N=1,614,264
	[.794, .805]	[1.009, 1.034]	[.985, 1.002]	[.984, 1.015]	[.788, .799]	[1.008, 1.034]	
Albania	.808***	1.017	1.001	1.074**	.808***	1.093***	N=13,749
	[.793, .823]	[.97, 1.068]	[.976, 1.026]	[1.005, 1.147]	[.794, .823]	[1.034, 1.154]	
Armenia	.79***	1.033	1.077***	1.005	.851***	1.038*	N=10,925
	[.774, .806]	[.992, 1.076]	[1.05, 1.105]	[.95, 1.063]	[.839, .864]	[.994, 1.085]	
Azerbaijan	.881***	1.018	.927***	.995	.817***	1.012	N=6,203
	[.865, .898]	[.981, 1.056]	[.899, .956]	[.932, 1.062]	[.8, .835]	[.947, 1.082]	
Colombia	.753***	.985	1.034***	1.033**	.778***	1.017*	N=61,245
	[.744, .761]	[.966, 1.004]	[1.018, 1.05]	[1.007, 1.059]	[.77, .787]	[.9975, 1.037]	
Egypt	.758***	1.02**	.911***	1.045***	.691***	1.067***	N=102,079
	[.752, .763]	[1.005, 1.036]	[.902, .921]	[1.024, 1.068]	[.684, .697]	[1.048, 1.086]	
Gabon	.89***	.969	1.001	1.038	.891***	1.006	N=8,462
	[.875, .906]	[.9, 1.044]	[.977, 1.025]	[.956, 1.127]	[.877, .905]	[.956, 1.059]	
Guatemala	.666***	.987	.872***	.986	.581***	.972	N=24,453
	[.652, .68]	[.94, 1.035]	[.849, .897]	[.921, 1.054]	[.567, .596]	[.922, 1.025]	
Guyana	.691***	.972	1.019	1.047	.704***	1.018	N=6,801
	[.666, .717]	[.921, 1.026]	[.974, 1.065]	[.972, 1.129]	[.682, .726]	[.961, 1.079]	
India	.756***	.993*	.958***	1.001	.724***	.994*	N=1,075,968
	[.754, .758]	[.987, 1]	[.955, .961]	[.992, 1.009]	[.722, .726]	[.987, 1.001]	
Indonesia	.771***	.993	1.03***	1.004	.794***	.9976	N=35,687
	[.762, .78]	[.974, 1.013]	[1.014, 1.047]	[.98, 1.03]	[.785, .804]	[.979, 1.016]	
Kyrgyz Republic	.882***	.986	1.025**	1.015	.904***	1.001	N=6,551
	[.866, .897]	[.956, 1.016]	[1.005, 1.046]	[.981, 1.052]	[.891, .917]	[.972, 1.03]	
Moldova	.819***	1.05**	1.088***	.977	.891***	1.026	N=5,457
	[.802, .837]	[1.003, 1.098]	[1.061, 1.115]	[.925, 1.033]	[.877, .905]	[.989, 1.064]	
Morocco	.584***	.976	.812***	.956	.474***	.933**	N=14,022
	[.567, .602]	[.92, 1.036]	[.781, .844]	[.884, 1.033]	[.456, .493]	[.874, .996]	
Pakistan	.514***	.993	.702***	1.031	.361***	1.023	N=70,840
	[.503, .526]	[.956, 1.03]	[.681, .722]	[.978, 1.087]	[.35, .372]	[.977, 1.072]	
Peru	.76***	1.001	.9975	1.007	.758***	1.008	N=82,853
	[.754, .767]	[.977, 1.026]	[.987, 1.008]	[.976, 1.039]	[.751, .765]	[.985, 1.032]	
Philippines	.768***	.9953	1.061***	1.006	.814***	1.001	N=39,196
	[.759, .777]	[.971, 1.02]	[1.044, 1.077]	[.975, 1.037]	[.805, .824]	[.977, 1.025]	
South Africa	.912***	1.024	.977**	1.013	.891***	1.037**	N=7,000

	Constant	Washer	Female	Interaction	Constant× Female	Interaction× Washer	
	[.9, .924]	[.989, 1.06]	[.958, .9963]	[.968, 1.061]	[.878, .904]	[1, 1.076]	
Tajikistan	.87***	.9973	.869***	.993	.756***	.991	N=16,544
	[.859, .88]	[.977, 1.018]	[.85, .889]	[.96, 1.028]	[.741, .772]	[.963, 1.019]	
Türkiye	.726***	.995	.875***	1.293***	.635***	1.287***	N=26,229
	[.714, .737]	[.95, 1.042]	[.855, .896]	[1.202, 1.392]	[.622, .648]	[1.201, 1.378]	

Notes: *P<0.1; **P<0.05; ***P<0.01. Rate ratios and a constant from Poisson regression models are shown, in addition to two combinations obtained using post estimation. Each row of estimates was obtained from a separate model. All models included a baseline term for being female and baseline terms and interactions with being female for washer ownership, a wealth index z-score, number of household members, number of household members under age five, age, highest education level of a male in household, and highest education level of a female in household, as well as adjusting for neighborhood. Except the baseline term for female, all independent variables, including fixed effects, were centered around a country-specific weighted mean: Therefore, the 'Constant' column shows school attendance for males with the mean on all other independent variables. The column labelled 'Washer' shows differences in school attendance for males with washer at home. The column labelled 'Interaction' shows interaction terms for females and washer. The column labelled 'Constant×Female' shows school attendance for females with the mean on all other independent variables, obtained using post estimation. The column labelled 'Interaction×Washer' shows difference in school attendance for girls with washer at home, obtained using post estimation. Estimates were weighted using sampling weights, rescaled to sum up to one for the final sample from each survey. 95% confidence intervals adjusted for clustering at the level of primary sampling units are shown in brackets below the point estimates. Pooled models were further rescaled such that each country contributed equally to the estimates.

Table S22. Results from Poisson regression models of school attendance on washer ownership: excluding the wealth index from independent variables

	Constant	Washer	Female	Interaction	Constant× Female	Interaction× Washer	
Pooled	.799***	1.029***	.994	1.007	.794***	1.037***	N=1,614,264
	[.794, .805]	[1.017, 1.042]	[.986, 1.002]	[.991, 1.023]	[.789, .8]	[1.023, 1.05]	
Albania	.808***	1.021	1.001	1.099***	.809***	1.122***	N=13,749
	[.794, .823]	[.975, 1.068]	[.976, 1.026]	[1.028, 1.176]	[.795, .823]	[1.063, 1.184]	
Armenia	.79***	1.036*	1.077***	1.023	.851***	1.06***	N=10,925
	[.774, .806]	[.9963, 1.078]	[1.05, 1.105]	[.968, 1.081]	[.839, .864]	[1.014, 1.108]	
Azerbaijan	.881***	1.03*	.928***	1.018	.818***	1.049	N=6,203
	[.865, .898]	[.9961, 1.065]	[.9, .956]	[.962, 1.077]	[.8, .836]	[.988, 1.113]	
Colombia	.752***	1.015	1.035***	1.019	.779***	1.035***	N=61,245
	[.744, .761]	[.997, 1.034]	[1.019, 1.051]	[.994, 1.044]	[.771, .787]	[1.016, 1.053]	
Egypt	.758***	1.004	.911***	1.026***	.69***	1.031***	N=102,079
	[.752, .763]	[.989, 1.019]	[.901, .92]	[1.007, 1.047]	[.683, .696]	[1.013, 1.048]	
Gabon	.89***	.982	1.002	1.026	.892***	1.007	N=8,462
	[.875, .906]	[.917, 1.051]	[.978, 1.026]	[.947, 1.111]	[.878, .905]	[.959, 1.058]	
Guatemala	.666***	1.031	.873***	1.026	.582***	1.058**	N=24,453
	[.652, .68]	[.992, 1.072]	[.85, .898]	[.965, 1.091]	[.567, .596]	[1.007, 1.112]	
Guyana	.691***	1.018	1.019	1.03	.704***	1.049*	N=6,801
	[.666, .717]	[.97, 1.068]	[.974, 1.066]	[.962, 1.103]	[.682, .727]	[.9959, 1.104]	
India	.757***	1.014***	.958***	1.005	.725***	1.019***	N=1,075,968
	[.754, .759]	[1.008, 1.02]	[.954, .961]	[.9975, 1.012]	[.722, .727]	[1.013, 1.025]	
Indonesia	.771***	1.019**	1.031***	.9975	.795***	1.017*	N=35,687
	[.762, .781]	[1.001, 1.038]	[1.014, 1.047]	[.975, 1.021]	[.785, .804]	[.9999, 1.034]	
Kyrgyz Republic	.882***	.982	1.025**	1.011	.903***	.993	N=6,551
	[.866, .898]	[.953, 1.012]	[1.004, 1.046]	[.977, 1.047]	[.89, .917]	[.966, 1.021]	
Moldova	.819***	1.071***	1.09***	.971	.892***	1.04**	N=5,457
	[.802, .836]	[1.022, 1.122]	[1.062, 1.118]	[.918, 1.028]	[.878, .906]	[1.004, 1.078]	
Morocco	.583***	.977	.822***	1.003	.479***	.98	N=14,022
	[.565, .6]	[.928, 1.029]	[.791, .853]	[.941, 1.069]	[.46, .498]	[.927, 1.035]	
Pakistan	.513***	1.015	.701***	1.123***	.359***	1.14***	N=70,840
	[.501, .525]	[.981, 1.051]	[.681, .722]	[1.07, 1.178]	[.349, .371]	[1.092, 1.19]	
Peru	.76***	1.036***	.9982	.9982	.759***	1.034***	N=82,853
	[.753, .767]	[1.014, 1.058]	[.988, 1.009]	[.972, 1.025]	[.752, .766]	[1.013, 1.056]	
Philippines	.768***	1.027**	1.061***	.977	.815***	1.003	N=39,196
	[.759, .777]	[1.004, 1.05]	[1.045, 1.078]	[.95, 1.006]	[.805, .825]	[.982, 1.026]	
South Africa	.912***	1.054***	.977**	1.002	.891***	1.056***	N=7,000
	[.9, .924]	[1.021, 1.087]	[.958, .9964]	[.963, 1.044]	[.878, .904]	[1.022, 1.091]	

	Constant	Washer	Female	Interaction	Constant× Female	Interaction× Washer	
Tajikistan	.87***	1.007	.869***	.992	.756***	.9987	N=16,544
	[.859, .88]	[.986, 1.027]	[.85, .889]	[.96, 1.025]	[.741, .772]	[.972, 1.026]	
Türkiye	.726***	1.004	.874***	1.27***	.634***	1.275***	N=26,229
	[.714, .737]	[.96, 1.05]	[.853, .894]	[1.177, 1.371]	[.621, .647]	[1.188, 1.369]	

Notes: *P<0.1; **P<0.05; ***P<0.01. Rate ratios and a constant from Poisson regression models are shown, in addition to two combinations obtained using post estimation. Each row of estimates was obtained from a separate model. All models included a baseline term for being female and baseline terms and interactions with being female for washer ownership, fridge ownership, TV ownership, having flush toilet, number of household members, number of household members under age five, age, highest education level of a male in household, and highest education level of a female in household, as well as adjusting for neighborhood. Except the baseline term for female, all independent variables were centered around a country-specific weighted mean: Therefore, the 'Constant' column shows school attendance for males with the mean on all other independent variables. The column labelled 'Washer' shows differences in school attendance for males with washer at home. The column labelled 'Interaction' shows interaction terms for females and washer. The column labelled 'Constant×Female' shows school attendance for females with the mean on all other independent variables, obtained using post estimation. The column labelled 'Interaction×Washer' shows difference in school attendance for girls with washer at home, obtained using post estimation. Estimates were weighted using sampling weights, rescaled to sum up to one for the final sample from each survey. Pooled models were further rescaled such that each country contributed equally to the estimates.95% confidence intervals adjusted for clustering at the level of primary sampling units are shown in brackets below the point estimates.

Table S23. Results from Poisson regression models of school attendance on washer ownership: only including descendants of household head

	Constant	Washer	Female	Interaction	Constant× Female	Interaction× Washer	
Pooled	.814***	1.017***	1.018***	1.009	.828***	1.026***	N=1,472,879
	[.808, .819]	[1.005, 1.029]	[1.01, 1.026]	[.993, 1.024]	[.823, .833]	[1.013, 1.038]	
Albania	.815***	1.018	1.019	1.074*	.83***	1.093***	N=13,406
	[.8, .829]	[.968, 1.07]	[.9958, 1.044]	[.9984, 1.155]	[.817, .844]	[1.031, 1.157]	
Armenia	.799***	1.022	1.087***	1.034	.868***	1.056**	N=10,555
	[.783, .814]	[.981, 1.064]	[1.061, 1.113]	[.976, 1.095]	[.856, .88]	[1.01, 1.105]	
Azerbaijan	.889***	1.022	.959***	.9955	.853***	1.017	N=5,905
	[.873, .904]	[.985, 1.061]	[.932, .988]	[.936, 1.059]	[.835, .87]	[.961, 1.077]	
Colombia	.782***	.989	1.062***	1.027**	.83***	1.015	N=52,196
	[.773, .79]	[.97, 1.008]	[1.046, 1.078]	[1.001, 1.053]	[.822, .838]	[.9959, 1.034]	
Egypt	.772***	1.013*	.959***	1.028***	.74***	1.042***	N=95,168
	[.767, .777]	[.9977, 1.029]	[.95, .968]	[1.008, 1.049]	[.734, .747]	[1.025, 1.059]	
Gabon	.922***	1.001	1.011	.975	.932***	.977	N=5,671
	[.908, .937]	[.924, 1.085]	[.99, 1.032]	[.886, 1.073]	[.92, .944]	[.919, 1.038]	
Guatemala	.697***	.976	.924***	1.021	.644***	.996	N=21,376
	[.683, .711]	[.927, 1.027]	[.899, .949]	[.956, 1.09]	[.629, .66]	[.949, 1.045]	
Guyana	.726***	.972	1.035	1.03	.752***	1.001	N=5,648
	[.701, .752]	[.919, 1.028]	[.992, 1.081]	[.956, 1.109]	[.728, .776]	[.947, 1.059]	
India	.774***	.996	1.001	1.001	.775***	.9975	N=996,121
	[.772, .776]	[.989, 1.003]	[.9978, 1.004]	[.993, 1.01]	[.772, .777]	[.991, 1.004]	
Indonesia	.792***	.996	1.043***	1.005	.827***	1.001	N=31,721
	[.784, .801]	[.977, 1.016]	[1.028, 1.059]	[.98, 1.031]	[.818, .836]	[.982, 1.02]	
Kyrgyz Republic	.891***	.983	1.049***	1.028	.935***	1.011	N=6,073
	[.876, .905]	[.954, 1.014]	[1.029, 1.071]	[.994, 1.064]	[.923, .946]	[.99, 1.033]	
Moldova	.829***	1.041*	1.106***	.976	.917***	1.016	N=5,015
	[.812, .846]	[.9951, 1.09]	[1.079, 1.133]	[.922, 1.033]	[.903, .93]	[.981, 1.053]	
Morocco	.601***	1.012	.846***	.941	.508***	.952	N=12,590
	[.584, .619]	[.951, 1.076]	[.813, .88]	[.869, 1.019]	[.489, .529]	[.896, 1.012]	
Pakistan	.538***	.977	.75***	1.043	.403***	1.019	N=60,609
	[.526, .55]	[.94, 1.015]	[.729, .772]	[.989, 1.099]	[.392, .415]	[.974, 1.066]	
Peru	.787***	.992	1.025***	1.015	.807***	1.007	N=70,592
	[.781, .794]	[.968, 1.017]	[1.015, 1.036]	[.983, 1.048]	[.8, .814]	[.983, 1.032]	
Philippines	.788***	.9963	1.1***	.988	.867***	.984	N=34,446
	[.78, .797]	[.972, 1.021]	[1.086, 1.115]	[.96, 1.017]	[.86, .875]	[.963, 1.006]	
South Africa	.926***	1.028	.984*	1.003	.912***	1.031*	N=5,698
	[.914, .938]	[.993, 1.063]	[.966, 1.003]	[.958, 1.05]	[.899, .924]	[.994, 1.069]	

	Constant	Washer	Female	Interaction	Constant× Female	Interaction× Washer	
Tajikistan	.881***	1.005	.915***	.988	.806***	.993	N=15,463
	[.871, .891]	[.983, 1.028]	[.895, .935]	[.955, 1.021]	[.791, .821]	[.968, 1.018]	
Türkiye	.74***	.9975	.906***	1.311***	.67***	1.308***	N=24,626
	[.729, .751]	[.954, 1.043]	[.886, .926]	[1.218, 1.412]	[.658, .683]	[1.221, 1.402]	

Notes: *P<0.1; **P<0.05; ***P<0.01. Rate ratios and a constant from Poisson regression models are shown, in addition to two combinations obtained using post estimation. Each row of estimates was obtained from a separate model. All models included a baseline term for being female and baseline terms and interactions with being female for washer ownership, fridge ownership, TV ownership, having flush toilet, a wealth index z-score, number of household members, number of household members under age five, age, highest education level of a male in household, and highest education level of a female in household, as well as adjusting for neighborhood. Except the baseline term for female, all independent variables, including fixed effects, were centered around a country-specific weighted mean: Therefore, the 'Constant' column shows school attendance for males with the mean on all other independent variables. The column labelled 'Washer' shows differences in school attendance for males with washer at home. The column labelled 'Interaction' shows interaction terms for females and washer. The column labelled 'Constant×Female' shows school attendance for females with the mean on all other independent variables, obtained using post estimation. The column labelled 'Interaction×Washer' shows difference in school attendance for girls with washer at home, obtained using post estimation. Estimates were weighted using sampling weights, rescaled to sum up to one for the final sample from each survey. 95% confidence intervals adjusted for clustering at the level of primary sampling units are shown in brackets below the point estimates. Pooled models were further rescaled such that each country contributed equally to the estimates.

Table S24. Results from Poisson regression models of school attendance on washer ownership: only including households with electricity

	Constant	Washer	Female	Interaction	Constant× Female	Interaction× Washer	
Pooled	.81***	1.016***	.995	1.006	.806***	1.022***	N=1,474,035
	[.804, .815]	[1.004, 1.029]	[.987, 1.003]	[.99, 1.021]	[.8, .811]	[1.009, 1.035]	
Albania	.808***	1.017	1	1.077**	.808***	1.095***	N=13,749
	[.793, .823]	[.967, 1.068]	[.976, 1.025]	[1.005, 1.155]	[.794, .822]	[1.034, 1.16]	
Armenia	.791***	1.014	1.077***	1.025	.851***	1.04*	N=10,916
	[.775, .807]	[.974, 1.056]	[1.05, 1.104]	[.97, 1.085]	[.839, .864]	[.994, 1.088]	
Azerbaijan	.883***	1.018	.926***	.9982	.818***	1.016	N=6,173
	[.867, .899]	[.983, 1.054]	[.899, .954]	[.938, 1.062]	[.801, .836]	[.953, 1.083]	
Colombia	.758***	.983*	1.034***	1.035***	.784***	1.018*	N=58,312
	[.749, .766]	[.964, 1.002]	[1.019, 1.05]	[1.01, 1.062]	[.776, .792]	[.9984, 1.038]	
Egypt	.76***	1.016**	.913***	1.023**	.694***	1.04***	N=100,806
	[.755, .766]	[1.001, 1.032]	[.904, .923]	[1.002, 1.045]	[.688, .701]	[1.022, 1.058]	
Gabon	.894***	.974	1.005	1.038	.898***	1.011	N=6,755
	[.878, .91]	[.906, 1.048]	[.979, 1.032]	[.955, 1.127]	[.884, .912]	[.956, 1.069]	
Guatemala	.684***	.99	.884***	.9955	.605***	.986	N=21,207
	[.67, .699]	[.945, 1.037]	[.859, .909]	[.933, 1.062]	[.59, .62]	[.937, 1.037]	
Guyana	.7***	.986	1.028	1.052	.72***	1.037	N=4,708
	[.673, .728]	[.933, 1.041]	[.98, 1.079]	[.975, 1.135]	[.696, .745]	[.976, 1.102]	
India	.767***	.992**	.962***	1.001	.738***	.993**	N=979,809
	[.765, .769]	[.985, .9983]	[.959, .966]	[.993, 1.01]	[.736, .74]	[.986, .9996]	
Indonesia	.773***	.993	1.031***	1.005	.797***	.998	N=34,245
	[.764, .783]	[.974, 1.013]	[1.014, 1.048]	[.98, 1.03]	[.788, .806]	[.98, 1.017]	
Kyrgyz Republic	.881***	.981	1.025**	1.012	.903***	.993	N=6,532
	[.866, .897]	[.952, 1.012]	[1.004, 1.046]	[.977, 1.047]	[.89, .916]	[.966, 1.021]	
Moldova	.823***	1.049**	1.087***	.971	.894***	1.019	N=5,417
	[.806, .84]	[1.002, 1.099]	[1.06, 1.115]	[.917, 1.027]	[.88, .908]	[.982, 1.056]	
Morocco	.657***	.992	.876***	1.001	.575***	.992	N=10,033
	[.638, .676]	[.936, 1.051]	[.846, .906]	[.927, 1.08]	[.557, .594]	[.932, 1.057]	
Pakistan	.538***	.988	.731***	1.034	.393***	1.021	N=65,330
	[.527, .549]	[.952, 1.025]	[.71, .752]	[.982, 1.089]	[.382, .405]	[.975, 1.069]	
Peru	.767***	1.002	1.012**	1.006	.776***	1.008	N=68,117
	[.76, .774]	[.977, 1.028]	[1.001, 1.023]	[.974, 1.038]	[.769, .783]	[.984, 1.032]	
Philippines	.799***	1.003	1.046***	.9974	.835***	1	N=33,073
	[.79, .808]	[.978, 1.028]	[1.03, 1.063]	[.967, 1.029]	[.825, .846]	[.977, 1.024]	
South Africa	.917***	1.027	.976**	1.009	.894***	1.036*	N=6,275
	[.905, .929]	[.992, 1.063]	[.956, .9952]	[.963, 1.057]	[.881, .908]	[.9985, 1.075]	
Tajikistan	.871***	1	.869***	.987	.757***	.987	N=16,349
	[.861, .881]	[.979, 1.022]	[.85, .889]	[.953, 1.022]	[.742, .772]	[.96, 1.016]	
Türkiye	.726***	.991	.874***	1.287***	.634***	1.275***	N=26,229
	[.714, .737]	[.947, 1.037]	[.853, .894]	[1.193, 1.388]	[.621, .647]	[1.189, 1.368]	

Notes: *P<0.1; **P<0.05; ***P<0.01. Rate ratios and a constant from Poisson regression models are shown, in addition to two combinations obtained using post estimation. Each row of estimates was obtained from a separate model. All models included a baseline term for being female and baseline terms and interactions with being female for washer ownership, fridge ownership, TV ownership, having flush toilet, a wealth index z-score, number of household members, number of household members under age five, age, highest education level of a male in household, and highest education level of a female in household, as well as adjusting for neighborhood. Except the baseline term for female, all independent variables, including fixed effects, were centered around a country-specific weighted mean: Therefore, the 'Constant' column shows school attendance for males with the mean on all other independent variables. The column labelled 'Washer' shows differences in school attendance for males with washer at home. The column labelled 'Interaction' shows interaction terms for females and washer. The column labelled 'Constant×Female' shows school attendance for females with the mean on all other independent variables, obtained using post estimation. The column labelled 'Interaction×Washer' shows difference in school attendance for girls with washer at home, obtained using post estimation. Estimates were weighted using sampling weights, rescaled to sum up to one for the final sample from each survey. 95% confidence intervals adjusted for clustering at the level of primary sampling units are shown in brackets below the point estimates. Pooled models were further rescaled such that each country contributed equally to the estimates.

Table S25. Results from Poisson regression models of school attendance on washer ownership: stratified by overall school attendance in region

	Constant	Washer	Female	Interaction	Constant× Female	Interaction× Washer	Mean school attendance	Mean washer ownership	
**Less than 80% school attendance**									
Pooled	.664***	.9962	.942***	1.036**	.626***	1.032**	.708	.393	N=990,876
	[.655, .674]	[.972, 1.021]	[.923, .961]	[1.003, 1.07]	[.616, .635]	[1.005, 1.059]	[.701, .716]	[.376, .41]	
Albania	.726***	.998	.912***	1.127***	.662***	1.125***	.754	.714	N=5,575
	[.698, .754]	[.935, 1.065]	[.862, .965]	[1.032, 1.23]	[.637, .688]	[1.046, 1.208]	[.736, .773]	[.676, .751]	
Armenia	.655***	.9963	1.123***	1.006	.735***	1.002	.767	.767	N=2,506
	[.619, .693]	[.921, 1.078]	[1.047, 1.206]	[.891, 1.135]	[.707, .766]	[.915, 1.097]	[.744, .789]	[.735, .799]	
Azerbaijan	.824***	.886***	.734***	1.182	.605***	1.047	.784	.0861	N=601
	[.792, .857]	[.809, .969]	[.673, .801]	[.911, 1.533]	[.571, .642]	[.819, 1.338]	[.728, .84]	[.0406, .132]	
Colombia	.655***	.974	1.062***	1.006	.696***	.98	.729	.223	N=24,784
	[.64, .671]	[.934, 1.015]	[1.033, 1.093]	[.958, 1.057]	[.683, .71]	[.942, 1.019]	[.719, .74]	[.208, .238]	
Egypt	.724***	1.031**	.865***	1.047***	.626***	1.08***	.724	.836	N=68,892
	[.717, .731]	[1.006, 1.056]	[.852, .877]	[1.012, 1.084]	[.618, .635]	[1.048, 1.112]	[.717, .73]	[.828, .843]	
Guatemala	.663***	.984	.869***	.995	.576***	.979	.681	.108	N=23,406
	[.649, .677]	[.935, 1.035]	[.845, .893]	[.926, 1.069]	[.561, .591]	[.924, 1.036]	[.668, .693]	[.0938, .122]	
Guyana	.651***	.951	1.025	1.078	.667***	1.025	.745	.161	N=4,807
	[.62, .682]	[.882, 1.026]	[.965, 1.088]	[.971, 1.196]	[.641, .693]	[.948, 1.108]	[.726, .765]	[.139, .184]	
India	.72***	.9968	.938***	.9966	.676***	.993	.749	.0743	N=696,884
	[.718, .723]	[.986, 1.008]	[.934, .943]	[.982, 1.011]	[.673, .679]	[.982, 1.005]	[.747, .751]	[.072, .0767]	
Indonesia	.702***	1.008	1.03	.959	.722***	.966	.769	.363	N=6,093
	[.679, .724]	[.954, 1.065]	[.982, 1.08]	[.896, 1.026]	[.699, .747]	[.92, 1.015]	[.754, .784]	[.324, .401]	
Morocco	.567***	1.015	.803***	.922*	.455***	.936*	.589	.125	N=13,077
	[.549, .585]	[.95, 1.084]	[.77, .837]	[.847, 1.005]	[.437, .475]	[.871, 1.006]	[.566, .613]	[.103, .146]	
Pakistan	.494***	.983	.677***	1.028	.335***	1.011	.499	.499	N=65,234
	[.483, .506]	[.945, 1.023]	[.656, .698]	[.973, 1.087]	[.325, .345]	[.962, 1.062]	[.485, .512]	[.478, .519]	
Peru	.716***	1.02	.9954	.99	.713***	1.01	.759	.171	N=42,829
	[.707, .726]	[.981, 1.061]	[.979, 1.012]	[.943, 1.038]	[.703, .724]	[.974, 1.046]	[.751, .766]	[.157, .185]	
Philippines	.705***	1	1.05***	1.015	.74***	1.015	.761	.289	N=14,858
	[.693, .718]	[.957, 1.045]	[1.025, 1.075]	[.961, 1.073]	[.728, .754]	[.972, 1.061]	[.751, .77]	[.267, .312]	
Tajikistan	.799***	.989	.785***	1.022	.628***	1.011	.792	.165	N=1,797
	[.769, .83]	[.909, 1.075]	[.716, .861]	[.873, 1.198]	[.583, .676]	[.882, 1.159]	[.768, .816]	[.122, .207]	
Türkiye	.679***	.991	.832***	1.288***	.565***	1.277***	.695	.818	N=19,533
	[.665, .693]	[.947, 1.038]	[.806, .859]	[1.191, 1.394]	[.55, .581]	[1.189, 1.371]	[.684, .706]	[.8, .835]	
**80–90% school attendance**									
Pooled	.822***	1.033***	1.015**	.99	.835***	1.023**	.854	.528	N=577,695

	Constant	Washer	Female	Interaction	Constant× Female	Interaction× Washer	Mean school attendance	Mean washer ownership	
	[.815, .829]	[1.015, 1.051]	[1.004, 1.027]	[.968, 1.014]	[.828, .842]	[1.004, 1.042]	[.849, .858]	[.512, .544]	
Albania	.838***	1.053	1.036**	1	.868***	1.053	.874	.933	N=5,393
	[.818, .859]	[.957, 1.159]	[1.002, 1.071]	[.894, 1.119]	[.85, .886]	[.965, 1.15]	[.859, .888]	[.921, .945]	
Armenia	.797***	1.035	1.081***	1.021	.862***	1.057**	.857	.841	N=7,048
	[.777, .819]	[.981, 1.093]	[1.046, 1.118]	[.953, 1.094]	[.847, .877]	[1.001, 1.117]	[.845, .87]	[.822, .86]	
Azerbaijan	.848***	1.043*	.906***	.982	.768***	1.024	.842	.14	N=3,931
	[.828, .868]	[.994, 1.094]	[.874, .94]	[.891, 1.081]	[.748, .79]	[.929, 1.129]	[.827, .858]	[.111, .17]	
Colombia	.802***	.989	1.019**	1.04***	.817***	1.029***	.835	.538	N=36,451
	[.793, .812]	[.969, 1.01]	[1.001, 1.037]	[1.011, 1.069]	[.808, .827]	[1.007, 1.051]	[.829, .842]	[.52, .556]	
Egypt	.817***	1.01	.988*	.976**	.807***	.985	.838	.759	N=33,187
	[.808, .825]	[.992, 1.029]	[.975, 1.002]	[.954, .9976]	[.798, .817]	[.968, 1.004]	[.83, .845]	[.747, .771]	
Gabon	.863***	1.001	.971	.86	.838***	.861**	.866	.0149	N=3,605
	[.843, .883]	[.847, 1.182]	[.938, 1.006]	[.703, 1.053]	[.817, .859]	[.758, .978]	[.849, .884]	[.00369, .0261]	
Guatemala	.768***	1.008	1.014	.963	.779***	.97	.822	.327	N=1,047
	[.725, .813]	[.883, 1.149]	[.927, 1.11]	[.817, 1.135]	[.746, .813]	[.902, 1.044]	[.775, .868]	[.275, .38]	
Guyana	.777***	1.022	1.013	.992	.787***	1.014	.831	.242	N=1,909
	[.747, .808]	[.951, 1.098]	[.956, 1.072]	[.878, 1.122]	[.756, .819]	[.919, 1.119]	[.812, .85]	[.193, .291]	
India	.82***	.989**	.994**	1.011**	.815***	1	.844	.237	N=361,870
	[.816, .823]	[.98, .9981]	[.989, .9992]	[1, 1.022]	[.811, .818]	[.991, 1.009]	[.841, .846]	[.232, .243]	
Indonesia	.786***	.989	1.03***	1.016	.809***	1.005	.834	.34	N=28,524
	[.775, .796]	[.968, 1.011]	[1.013, 1.048]	[.989, 1.044]	[.799, .819]	[.985, 1.026]	[.827, .842]	[.323, .356]	
Kyrgyz Republic	.838***	.984	1.027	.9989	.861***	.983	.88	.522	N=2,664
	[.816, .861]	[.941, 1.029]	[.993, 1.062]	[.947, 1.054]	[.841, .881]	[.941, 1.027]	[.859, .902]	[.483, .562]	
Moldova	.786***	1.057**	1.114***	.961	.875***	1.016	.858	.603	N=3,740
	[.765, .807]	[1.002, 1.115]	[1.078, 1.151]	[.899, 1.027]	[.858, .893]	[.973, 1.06]	[.843, .872]	[.573, .633]	
Morocco	.799***	.936	.932*	1.162	.745***	1.087	.814	.317	N=945
	[.772, .827]	[.804, 1.089]	[.865, 1.005]	[.917, 1.474]	[.71, .783]	[.95, 1.244]	[.772, .856]	[.215, .42]	
Pakistan	.866***	1.034	.92***	1.053	.797***	1.089***	.852	.505	N=5,606
	[.848, .885]	[.975, 1.096]	[.882, .959]	[.987, 1.123]	[.769, .826]	[1.029, 1.152]	[.828, .877]	[.429, .58]	
Peru	.803***	.984	1	1.02	.803***	1.004	.833	.179	N=39,366
	[.794, .811]	[.953, 1.016]	[.987, 1.014]	[.978, 1.064]	[.794, .812]	[.972, 1.036]	[.828, .839]	[.165, .194]	
Philippines	.826***	.9994	1.063***	.9972	.878***	.9967	.868	.416	N=22,312
	[.815, .838]	[.971, 1.029]	[1.043, 1.084]	[.961, 1.035]	[.867, .89]	[.97, 1.024]	[.859, .877]	[.385, .448]	
South Africa	.847***	1.067*	1.02	1.051	.864***	1.122***	.876	.518	N=1,763
	[.821, .873]	[.992, 1.148]	[.976, 1.067]	[.964, 1.146]	[.841, .888]	[1.055, 1.193]	[.858, .894]	[.46, .577]	
Tajikistan	.863***	1.006	.866***	1.002	.747***	1.009	.842	.245	N=11,638
	[.85, .876]	[.98, 1.033]	[.843, .889]	[.967, 1.04]	[.73, .765]	[.981, 1.037]	[.832, .853]	[.219, .27]	
Türkiye	.828***	1.127	.961***	.9993	.795***	1.126	.838	.979	N=6,696
	[.811, .845]	[.924, 1.374]	[.933, .989]	[.788, 1.267]	[.777, .814]	[.848, 1.495]	[.824, .853]	[.973, .986]	

	Constant	Washer	Female	Interaction	Constant× Female	Interaction× Washer	Mean school attendance	Mean washer ownership	
**Over 90% school attendance**									
Pooled	.917***	.9951	1.001	1.007	.918***	1.002	.924	.483	N=45,693
	[.909, .925]	[.976, 1.015]	[.99, 1.012]	[.983, 1.031]	[.91, .925]	[.982, 1.022]	[.919, .93]	[.455, .51]	
Albania	.901***	.959	1.039***	1.018	.936***	.977	.928	.95	N=2,781
	[.882, .92]	[.857, 1.073]	[1.011, 1.068]	[.855, 1.213]	[.921, .951]	[.841, 1.135]	[.911, .944]	[.935, .966]	
Armenia	.888***	1.019	1.06***	.992	.941***	1.01	.928	.955	N=1,371
	[.864, .914]	[.93, 1.116]	[1.022, 1.098]	[.88, 1.117]	[.925, .958]	[.941, 1.084]	[.915, .942]	[.936, .974]	
Azerbaijan	.937***	.979	.956*	1.038	.896***	1.016	.927	.329	N=1,671
	[.914, .96]	[.936, 1.025]	[.912, 1.002]	[.965, 1.116]	[.869, .923]	[.947, 1.091]	[.908, .946]	[.276, .383]	
Colombia	.000383	9.03e-29	78274		29.97	9.03e-29	.9	.8	N=10
	[., .]	[., .]	[., .]		[., .]	[., .]	[., .]	[., .]	
Gabon	.895***	.967	1.007	1.044	.901***	1.009	.909	.0837	N=4,857
	[.878, .912]	[.898, 1.041]	[.978, 1.036]	[.958, 1.137]	[.886, .916]	[.952, 1.07]	[.894, .923]	[.0584, .109]	
Guyana	.9992		.832***		.831***	NA	.932	0	N=85
	[.958, 1.042]		[.776, .892]		[.752, .918]		[.846, 1.018]	[., .]	
India	.933***	1.026***	1.015***	.977**	.948***	1.003	.945	.427	N=17,214
	[.925, .942]	[1.007, 1.046]	[1.004, 1.027]	[.955, .9995]	[.941, .954]	[.986, 1.019]	[.94, .951]	[.408, .447]	
Indonesia	.868***	.923*	1.055**	1.047	.916***	.967	.908	.546	N=1,070
	[.838, .899]	[.852, 1.001]	[1.007, 1.106]	[.953, 1.151]	[.897, .935]	[.907, 1.03]	[.888, .928]	[.487, .606]	
Kyrgyz Republic	.916***	.988	1.022*	1.018	.936***	1.006	.936	.743	N=3,887
	[.898, .934]	[.949, 1.029]	[.9995, 1.046]	[.976, 1.063]	[.924, .949]	[.977, 1.037]	[.923, .95]	[.707, .778]	
Moldova	.907***	1.031	1.038**	1.025	.941***	1.057	.93	.822	N=1,717
	[.886, .928]	[.962, 1.106]	[1.007, 1.07]	[.936, 1.122]	[.926, .957]	[.989, 1.13]	[.916, .945]	[.791, .852]	
Peru	.907***	1.017	.95	.937	.862***	.952	.917	.0264	N=658
	[.875, .94]	[.852, 1.213]	[.88, 1.027]	[.641, 1.37]	[.821, .905]	[.719, 1.261]	[.876, .958]	[-.01, .0628]	
Philippines	.892***	1.007	1.033*	.956	.922***	.963	.915	.324	N=2,026
	[.874, .911]	[.953, 1.065]	[.995, 1.073]	[.873, 1.047]	[.901, .943]	[.898, 1.032]	[.899, .932]	[.257, .392]	
South Africa	.932***	1.008	.967***	.9985	.901***	1.007	.925	.323	N=5,237
	[.919, .945]	[.971, 1.047]	[.946, .987]	[.947, 1.053]	[.886, .916]	[.965, 1.05]	[.915, .934]	[.282, .363]	
Tajikistan	.932***	1.002	.93***	.912*	.866***	.914**	.914	.399	N=3,109
	[.917, .946]	[.962, 1.044]	[.889, .972]	[.832, 1]	[.835, .898]	[.844, .991]	[.896, .933]	[.334, .465]	

Notes: *P<0.1; **P<0.05; ***P<0.01. Rate ratios and a constant from Poisson regression models are shown, in addition to two combinations obtained using post estimation. Each row of estimates was obtained from a separate model. All models included a baseline term for being female and baseline terms and interactions with being female for washer ownership, fridge ownership, TV ownership, having flush toilet, a wealth index z-score, number of household members, number of household members under age five, age, highest education level of a male in household, and highest education level of a female in household, as well as adjusting for neighborhood. Except the baseline term for female, all independent variables, including fixed effects, were centered around a country-specific weighted mean: Therefore, the 'Constant' column shows school attendance for males with the mean on all other independent variables. The column labelled 'Washer' shows differences in school attendance for males with washer at home. The column labelled 'Interaction' shows interaction terms for females and washer. The column labelled 'Constant×Female' shows school attendance for females with the mean on all other independent variables, obtained using post estimation. The column labelled 'Interaction×Washer' shows difference in school attendance for girls with washer at home, obtained using post estimation. Means for school attendance and washer ownership are also shown. Estimates were weighted

using sampling weights, rescaled to sum up to one for the final sample from each survey. 95% confidence intervals adjusted for clustering at the level of primary sampling units are shown in brackets below the point estimates. Pooled models were further rescaled such that each country contributed equally to the estimates.

Table S26. Results from linear regression models of school attendance on washer ownership: number of complete years of education as an outcome

	Constant	Washer	Female	Interaction	Constant+ Female	Interaction+ Washer	
Pooled	7.11***	.0351	.0244*	-.00632	7.134***	.0288	N=1,602,595
	[7.076, 7.143]	[-.0172, .0874]	[-.00182, .0505]	[-.074, .0613]	[7.098, 7.17]	[-.0259, .0835]	R sq 0.576
Albania	7.295***	.256**	.0717*	-.0207	7.367***	.236**	N=11,718
	[7.231, 7.359]	[.0336, .479]	[-.00794, .151]	[-.277, .236]	[7.304, 7.429]	[.0196, .451]	R sq 0.655
Armenia	7.591***	.118	.00744	-.0159	7.599***	.102	N=10,913
	[7.54, 7.643]	[-.0327, .268]	[-.0484, .0632]	[-.193, .161]	[7.55, 7.647]	[-.0287, .233]	R sq 0.796
Azerbaijan	7.816***	-.0604	-.115***	.0978	7.701***	.0374	N=6,152
	[7.761, 7.871]	[-.225, .104]	[-.199, -.0317]	[-.202, .398]	[7.63, 7.771]	[-.239, .313]	R sq 0.724
Colombia	6.547***	.00994	.486***	.0426	7.033***	.0525	N=61,114
	[6.513, 6.582]	[-.0729, .0928]	[.446, .526]	[-.0527, .138]	[7.002, 7.064]	[-.0229, .128]	R sq 0.625
Egypt	6.7***	.0716*	-.284***	.219***	6.416***	.29***	N=102,010
	[6.673, 6.727]	[-.00757, .151]	[-.322, -.246]	[.118, .319]	[6.381, 6.451]	[.202, .378]	R sq 0.496
Gabon	5.638***	.366	.244***	-.0648	5.882***	.302	N=8,424
	[5.558, 5.719]	[-.511, 1.243]	[.128, .36]	[-.892, .762]	[5.813, 5.952]	[-.11, .713]	R sq 0.590
Guatemala	5.628***	-.0813	-.0612*	-.0883	5.567***	-.17	N=24,429
	[5.576, 5.68]	[-.256, .0934]	[-.124, .0017]	[-.341, .164]	[5.507, 5.627]	[-.374, .0351]	R sq 0.525
Guyana	7.622***	.0274	.257***	-.171	7.88***	-.143	N=6,263
	[7.521, 7.724]	[-.174, .229]	[.149, .366]	[-.507, .166]	[7.781, 7.978]	[-.413, .127]	R sq 0.657
India	7.153***	-.108***	.0926***	.0102	7.246***	-.0974***	N=1,073,334
	[7.142, 7.164]	[-.142, -.0736]	[.0806, .105]	[-.0313, .0517]	[7.235, 7.257]	[-.132, -.0625]	R sq 0.556
Indonesia	7.515***	-.0409	.289***	.0394	7.804***	-.00145	N=35,501
	[7.478, 7.551]	[-.122, .0405]	[.245, .333]	[-.0625, .141]	[7.771, 7.837]	[-.0737, .0708]	R sq 0.703
Kyrgyz Republic	7.547***	.00293	.136***	.0504	7.682***	.0534	N=6,548
	[7.489, 7.604]	[-.103, .109]	[.0615, .21]	[-.0769, .178]	[7.627, 7.738]	[-.0442, .151]	R sq 0.844
Morocco	5.219***	.135	-.6***	-.55***	4.619***	-.415**	N=13,961
	[5.125, 5.314]	[-.142, .412]	[-.709, -.492]	[-.921, -.179]	[4.53, 4.709]	[-.748, -.0818]	R sq 0.387
Pakistan	4.779***	-.0701	-.732***	.309***	4.047***	.239***	N=70,688
	[4.726, 4.833]	[-.2, .0595]	[-.798, -.666]	[.141, .476]	[3.986, 4.108]	[.108, .369]	R sq 0.475
Peru	7.351***	.0236	.0398***	-.0718	7.39***	-.0482	N=82,743
	[7.329, 7.372]	[-.0628, .11]	[.0126, .067]	[-.183, .0397]	[7.367, 7.414]	[-.129, .0327]	R sq 0.692
Philippines	6.57***	-.041	.576***	.0796	7.146***	.0386	N=39,126
	[6.531, 6.609]	[-.148, .0661]	[.533, .62]	[-.0427, .202]	[7.11, 7.182]	[-.0434, .121]	R sq 0.682
South Africa	6.782***	.157*	.514***	-.105	7.296***	.0519	N=6,960
	[6.709, 6.854]	[-.00954, .324]	[.426, .603]	[-.315, .104]	[7.24, 7.351]	[-.0901, .194]	R sq 0.765
Tajikistan	7.605***	-.0509	-.31***	-.00021	7.295***	-.0511	N=16,501
	[7.564, 7.645]	[-.129, .0274]	[-.373, -.246]	[-.118, .117]	[7.24, 7.35]	[-.156, .054]	R sq 0.783

	Constant	Washer	Female	Interaction	Constant+ Female	Interaction+ Washer	
Türkiye	7.262***	.0196	-.349***	.714***	6.913***	.733***	N=26,210
	[7.221, 7.302]	[-.126, .165]	[-.403, -.295]	[.505, .922]	[6.862, 6.963]	[.541, .926]	R sq 0.587

Notes: *P<0.1; **P<0.05; ***P<0.01. Regression coefficients and a constant from linear regression models are shown, in addition to two combinations obtained using post estimation. Each row of estimates was obtained from a separate model. All models included a baseline term for being female and baseline terms and interactions with being female for washer ownership, fridge ownership, TV ownership, having flush toilet, a wealth index z-score, number of household members, number of household members under age five, age, highest education level of a male in household, and highest education level of a female in household, as well as adjusting for neighborhood. Except the baseline term for female, all independent variables, including fixed effects, were centered around a country-specific weighted mean: Therefore, the 'Constant' column shows school attendance for males with the mean on all other independent variables. The column labelled 'Washer' shows differences in school attendance for males with washer at home. The column labelled 'Interaction' shows interaction terms for females and washer. The column labelled 'Constant+Female' shows school attendance for females with the mean on all other independent variables, obtained using post estimation. The column labelled 'Interaction+Washer' shows difference in school attendance for girls with washer at home, obtained using post estimation. Estimates were weighted using sampling weights, rescaled to sum up to one for the final sample from each survey. 95% confidence intervals adjusted for clustering at the level of primary sampling units are shown in brackets below the point estimates. Pooled models were further rescaled such that each country contributed equally to the estimates.

Table S27. Results from linear regression models of school attendance on washer ownership: number of complete years of education as an outcome, by survey years

	Constant	Washer	Female	Interaction	Constant+ Female	Interaction+ Washer	Mean school attendance	Mean washer ownership	
**Earliest surveys (~2000–08)**									
Pooled	7.027***	.0631	-.0861***	-.0896	6.941***	-.0265	.77	.395	N=173,426
	[6.96, 7.094]	[-.0317, .158]	[-.139, -.0335]	[-.225, .046]	[6.861, 7.021]	[-.146, .0932]	[.762, .779]	[.376, .414]	R sq 0.495
Albania	7.322***	.165	.091**	.0896	7.413***	.254**	.809	.781	N=6,363
(2008–09)	[7.252, 7.392]	[-.0692, .399]	[.000215, .182]	[-.202, .381]	[7.35, 7.476]	[.0217, .487]	[.793, .825]	[.751, .812]	R sq 0.717
Armenia	6.65***	.108	-.096*	-.129	6.554***	-.0218	.825	.72	N=4,514
(2005)	[6.571, 6.729]	[-.101, .316]	[-.205, .0127]	[-.371, .112]	[6.487, 6.622]	[-.211, .167]	[.807, .843]	[.693, .747]	R sq 0.679
Azerbaijan	7.816***	-.0604	-.115***	.0978	7.701***	.0374	.871	.206	N=6,152
(2006)	[7.761, 7.871]	[-.225, .104]	[-.199, -.0317]	[-.202, .398]	[7.63, 7.771]	[-.239, .313]	[.857, .884]	[.177, .234]	R sq 0.724
Colombia	6.172***	.0205	.48***	.0107	6.652***	.0313	.769	.242	N=31,703
(2004–05)	[6.126, 6.218]	[-.117, .158]	[.427, .534]	[-.148, .17]	[6.609, 6.695]	[-.0847, .147]	[.761, .777]	[.229, .255]	R sq 0.594
Egypt	6.569***	.0579	-.462***	.266***	6.106***	.324***	.739	.812	N=60,097
(2000,2003,2005)	[6.532, 6.606]	[-.0539, .17]	[-.516, -.409]	[.12, .413]	[6.057, 6.156]	[.196, .453]	[.732, .747]	[.803, .821]	R sq 0.467
Guyana	8.06***	.129	.315***	-.191	8.375***	-.0617	.756	.139	N=1,770
(2005)	[7.924, 8.197]	[-.237, .494]	[.134, .496]	[-.893, .512]	[8.269, 8.482]	[-.633, .51]	[.73, .783]	[.111, .167]	R sq 0.727
Morocco	5.219***	.135	-.6***	-.55***	4.619***	-.415**	.604	.138	N=13,961
(2003–04)	[5.125, 5.314]	[-.142, .412]	[-.709, -.492]	[-.921, -.179]	[4.53, 4.709]	[-.748, -.0818]	[.582, .626]	[.116, .16]	R sq 0.387
Pakistan	4.281***	.0613	-.973***	.213*	3.308***	.275***	.355	.445	N=25,546
(2006–07)	[4.209, 4.352]	[-.138, .261]	[-1.06, -.881]	[-.0308, .457]	[3.231, 3.385]	[.0708, .478]	[.339, .37]	[.421, .469]	R sq 0.459
Philippines	6.111***	-.0209	.613***	.0721	6.723***	.0512	.765	.296	N=13,812
(2003)	[6.061, 6.161]	[-.183, .142]	[.554, .671]	[-.126, .27]	[6.676, 6.771]	[-.0781, .181]	[.755, .774]	[.274, .318]	R sq 0.656
Türkiye	6.797***	-.131	-.673***	.343*	6.124***	.213	.668	.734	N=9,508
(2003–04)	[6.731, 6.862]	[-.38, .119]	[-.767, -.579]	[-.021, .708]	[6.038, 6.209]	[-.0816, .507]	[.652, .684]	[.706, .763]	R sq 0.557
**Mid surveys (~2008–16)**									
Pooled	7.068***	.0177	.0527***	.0273	7.121***	.0449	.824	.41	N=774,212
	[7.012, 7.124]	[-.0711, .106]	[.0148, .0905]	[-.0747, .129]	[7.068, 7.174]	[-.025, .115]	[.818, .831]	[.391, .428]	R sq 0.589
Armenia	8.197***	.0921	.0828**	.00484	8.28***	.0969	.839	.86	N=3,168
(2010)	[8.13, 8.264]	[-.149, .334]	[3.47e-06, .166]	[-.259, .269]	[8.24, 8.32]	[-.077, .271]	[.818, .861]	[.838, .882]	R sq 0.855
Colombia	6.925***	.0664	.493***	.00106	7.418***	.0674	.831	.621	N=29,411
(2015–16)	[6.877, 6.973]	[-.0483, .181]	[.436, .55]	[-.144, .146]	[7.376, 7.46]	[-.0401, .175]	[.824, .839]	[.602, .64]	R sq 0.658
Egypt	6.791***	.0766	-.101***	-.0637	6.689***	.0129	.766	.825	N=19,367
(2008)	[6.736, 6.845]	[-.0992, .252]	[-.173, -.0296]	[-.289, .162]	[6.626, 6.753]	[-.173, .199]	[.756, .777]	[.811, .839]	R sq 0.515
Gabon	5.638***	.366	.244***	-.0648	5.882***	.302	.905	.0715	N=8,424
(2012)	[5.558, 5.719]	[-.511, 1.243]	[.128, .36]	[-.892, .762]	[5.813, 5.952]	[-.11, .713]	[.894, .916]	[.0502, .0929]	R sq 0.590
Guatemala	5.628***	-.0813	-.0612*	-.0883	5.567***	-.17	.684	.113	N=24,429

	Constant	Washer	Female	Interaction	Constant+ Female	Interaction+ Washer	Mean school attendance	Mean washer ownership	
(2014–15)	[5.576, 5.68]	[-.256, .0934]	[-.124, .0017]	[-.341, .164]	[5.507, 5.627]	[-.374, .0351]	[.671, .696]	[.099, .127]	R sq 0.525
Guyana	7.207***	-.102	.226***	-.113	7.433***	-.214*	.751	.215	N=4,493
(2009)	[7.118, 7.296]	[-.329, .125]	[.112, .341]	[-.45, .225]	[7.344, 7.522]	[-.466, .0375]	[.731, .772]	[.187, .244]	R sq 0.637
India	7.082***	-.0963***	.0349***	-.0121	7.116***	-.108***	.76	.109	N=556,739
(2015–16)	[7.066, 7.098]	[-.151, -.0413]	[.0174, .0524]	[-.0806, .0563]	[7.1, 7.133]	[-.166, -.0506]	[.757, .762]	[.106, .113]	R sq 0.531
Kyrgyz Republic	7.547***	.00293	.136***	.0504	7.682***	.0534	.91	.638	N=6,548
(2012)	[7.489, 7.604]	[-.103, .109]	[.0615, .21]	[-.0769, .178]	[7.627, 7.738]	[-.0442, .151]	[.897, .923]	[.606, .671]	R sq 0.844
Pakistan	4.878***	-.0201	-.641***	.333**	4.237***	.313**	.564	.492	N=22,042
(2012–13)	[4.789, 4.968]	[-.272, .232]	[-.765, -.517]	[.00434, .662]	[4.126, 4.349]	[.0528, .573]	[.54, .588]	[.45, .533]	R sq 0.467
Peru	7.351***	.0236	.0398***	-.0718	7.39***	-.0482	.796	.174	N=82,743
(2009,2010,2011,2012)	[7.329, 7.372]	[-.0628, .11]	[.0126, .067]	[-.183, .0397]	[7.367, 7.414]	[-.129, .0327]	[.791, .801]	[.164, .184]	R sq 0.692
Tajikistan	7.563***	-.0958	-.375***	.0266	7.189***	-.0692	.829	.168	N=8,236
(2012)	[7.496, 7.631]	[-.245, .053]	[-.474, -.276]	[-.169, .223]	[7.121, 7.256]	[-.237, .0986]	[.815, .844]	[.145, .19]	R sq 0.749
Türkiye	7.335***	.00937	-.339***	.936***	6.997***	.946***	.732	.903	N=8,612
(2008)	[7.263, 7.408]	[-.282, .3]	[-.44, -.237]	[.464, 1.409]	[6.918, 7.075]	[.57, 1.322]	[.715, .748]	[.886, .921]	R sq 0.601
**Latest surveys (~2013–21)**									
Pooled	7.28***	.0932**	.148***	-.0272	7.428***	.066*	.87	.613	N=654,957
	[7.242, 7.319]	[.0142, .172]	[.108, .187]	[-.129, .0743]	[7.394, 7.462]	[-.00982, .142]	[.865, .875]	[.594, .632]	R sq 0.706
Albania	7.266***	.402	.0442	-.463	7.31***	-.0607	.868	.92	N=5,355
(2017–18)	[7.163, 7.369]	[-.117, .921]	[-.0893, .178]	[-1.15, .222]	[7.21, 7.411]	[-.624, .502]	[.852, .883]	[.905, .935]	R sq 0.647
Armenia	7.882***	.41**	.067*	-.341	7.949***	.0693	.886	.952	N=3,231
(2015–16)	[7.824, 7.94]	[.00476, .816]	[-.00771, .142]	[-.928, .247]	[7.894, 8.004]	[-.26, .399]	[.873, .899]	[.94, .964]	R sq 0.905
Egypt	7.006***	.0279	.0623*	-.00519	7.068***	.0227	.831	.784	N=22,546
(2014)	[6.955, 7.056]	[-.103, .159]	[-.000768, .125]	[-.153, .143]	[7.016, 7.12]	[-.114, .159]	[.822, .841]	[.771, .798]	R sq 0.608
India	7.223***	-.0747***	.144***	.0175	7.368***	-.0572**	.803	.151	N=516,595
(2019–21)	[7.208, 7.238]	[-.118, -.0314]	[.128, .161]	[-.0355, .0705]	[7.354, 7.382]	[-.101, -.0134]	[.8, .805]	[.147, .155]	R sq 0.584
Indonesia	7.515***	-.0409	.289***	.0394	7.804***	-.00145	.822	.349	N=35,501
(2017)	[7.478, 7.551]	[-.122, .0405]	[.245, .333]	[-.0625, .141]	[7.771, 7.837]	[-.0737, .0708]	[.816, .829]	[.334, .364]	R sq 0.703
Pakistan	5.178***	-.223**	-.562***	.234	4.616***	.0111	.63	.561	N=23,100
(2017–18)	[5.083, 5.273]	[-.435, -.0107]	[-.68, -.445]	[-.0646, .533]	[4.518, 4.714]	[-.199, .222]	[.606, .653]	[.525, .597]	R sq 0.511
Philippines	7.059***	-.0278	.515***	.112	7.574***	.0839	.869	.402	N=25,314
(2017)	[7.01, 7.108]	[-.161, .106]	[.454, .576]	[-.0318, .255]	[7.53, 7.617]	[-.0175, .185]	[.861, .878]	[.372, .433]	R sq 0.717
South Africa	6.782***	.157*	.514***	-.105	7.296***	.0519	.913	.374	N=6,960
(2016)	[6.709, 6.854]	[-.00954, .324]	[.426, .603]	[-.315, .104]	[7.24, 7.351]	[-.0901, .194]	[.904, .921]	[.34, .408]	R sq 0.765
Tajikistan	7.643***	.00669	-.236***	-.0291	7.407***	-.0224	.868	.351	N=8,265
(2017)	[7.608, 7.678]	[-.0803, .0937]	[-.309, -.163]	[-.187, .129]	[7.337, 7.478]	[-.164, .119]	[.857, .879]	[.315, .388]	R sq 0.831
Türkiye	7.658***	.201	-.052	-.186	7.606***	.0153	.812	.959	N=8,090
(2013)	[7.605, 7.712]	[-.178, .581]	[-.124, .02]	[-.925, .553]	[7.541, 7.671]	[-.519, .549]	[.798, .827]	[.947, .971]	R sq 0.657

Notes: *P<0.1; **P<0.05; ***P<0.01. Regression coefficients and a constant from linear regression models are shown, in addition to two combinations obtained using post estimation. Surveys were divided into the periods indicated at the top of each panel: in some countries other periodization were used (shown in parentheses below country name). Each row of estimates was obtained from a separate model. All models included a baseline term for being female and baseline terms and interactions with being female for washer ownership, fridge ownership, TV ownership, having flush toilet, a wealth index z-score, number of household members, number of household members under age five, age, highest education level of a male in household, and highest education level of a female in household, as well as adjusting for neighborhood. Except the baseline term for female, all independent variables were centered around a country-specific weighted mean: Therefore, the 'Constant' column shows school attendance for males with the mean on all other independent variables. The column labelled 'Washer' shows differences in school attendance for males with washer at home. The column labelled 'Interaction' shows interaction terms for females and washer. The column labelled 'Constant+Female' shows school attendance for females with the mean on all other independent variables, obtained using post estimation. The column labelled 'Interaction+Washer' shows difference in school attendance for girls with washer at home, obtained using post estimation. Means for school attendance and washer ownership are also shown. Estimates were weighted using sampling weights, rescaled to sum up to one for the final sample from each survey. Pooled models were further rescaled such that each country contributed equally to the estimates.95% confidence intervals adjusted for clustering at the level of primary sampling units are shown in brackets below the point estimates.

Table S28. Results from Poisson regression models of school attendance on washer ownership: stratified by the extent of female disadvantage in school attendance at the regional level

	Constant	Washer	Female	Interaction	Constant× Female	Interaction× Washer	Mean school attendance	Mean washer ownership	
**No female-disadvantage**									
Pooled	.8***	1.023**	1.074***	1.002	.859***	1.024***	.851	.537	N=558,151
	[.791, .808]	[1.002, 1.044]	[1.061, 1.086]	[.978, 1.026]	[.851, .866]	[1.006, 1.043]	[.845, .857]	[.518, .556]	
Albania	.844***	1.001	1.059***	1.083	.894***	1.084*	.886	.945	N=6,627
	[.826, .863]	[.918, 1.092]	[1.028, 1.091]	[.968, 1.211]	[.878, .91]	[.987, 1.19]	[.872, .899]	[.935, .956]	
Armenia	.794***	1.009	1.115***	1.044	.885***	1.054*	.864	.849	N=6,732
	[.775, .813]	[.954, 1.067]	[1.083, 1.148]	[.975, 1.119]	[.871, .898]	[.9993, 1.111]	[.851, .877]	[.832, .867]	
Colombia	.722***	.971**	1.065***	1.049***	.769***	1.019	.782	.417	N=33,778
	[.711, .734]	[.944, .9986]	[1.042, 1.088]	[1.012, 1.088]	[.759, .78]	[.991, 1.047]	[.774, .79]	[.395, .439]	
Egypt	.78***	1.018	1.016*	.979	.792***	.9963	.815	.796	N=20,711
	[.769, .79]	[.99, 1.047]	[.9982, 1.033]	[.947, 1.012]	[.781, .802]	[.969, 1.024]	[.806, .824]	[.782, .81]	
Gabon	.874***	.972	1.031	1.039	.901***	1.01	.903	.101	N=2,886
	[.852, .896]	[.898, 1.053]	[.993, 1.071]	[.946, 1.14]	[.884, .919]	[.946, 1.077]	[.885, .921]	[.0696, .133]	
Guyana	.677***	.939	1.088***	1.115*	.737***	1.047	.775	.17	N=2,870
	[.644, .712]	[.858, 1.028]	[1.023, 1.158]	[.9998, 1.243]	[.709, .765]	[.964, 1.136]	[.752, .797]	[.141, .199]	
India	.755***	.993	1.031***	.9998	.779***	.993	.805	.225	N=349,054
	[.751, .759]	[.983, 1.003]	[1.025, 1.038]	[.987, 1.012]	[.775, .783]	[.983, 1.003]	[.802, .809]	[.219, .23]	
Indonesia	.775***	.99	1.043***	1.014	.808***	1.003	.829	.349	N=29,765
	[.765, .785]	[.968, 1.011]	[1.025, 1.06]	[.986, 1.042]	[.799, .818]	[.983, 1.023]	[.822, .836]	[.333, .365]	
Kyrgyz Republic	.87***	1.007	1.034**	1.014	.9***	1.022	.904	.668	N=3,315
	[.85, .89]	[.961, 1.056]	[1.003, 1.067]	[.963, 1.068]	[.883, .917]	[.991, 1.053]	[.888, .92]	[.623, .712]	
Moldova	.819***	1.047*	1.088***	.973	.891***	1.019	.875	.653	N=5,457
	[.802, .836]	[.9995, 1.097]	[1.061, 1.115]	[.919, 1.031]	[.877, .905]	[.982, 1.058]	[.863, .887]	[.627, .679]	
Morocco	.715***	1.033	1.015	1.016	.726***	1.05	.752	.246	N=3,455
	[.692, .739]	[.955, 1.118]	[.97, 1.062]	[.923, 1.119]	[.7, .752]	[.972, 1.133]	[.726, .778]	[.197, .296]	
Pakistan	.596***	.992	1.056**	1.052	.629***	1.044	.668	.808	N=8,969
	[.573, .619]	[.895, 1.1]	[1.011, 1.103]	[.913, 1.212]	[.606, .653]	[.932, 1.17]	[.645, .692]	[.783, .833]	
Peru	.743***	1.007	1.048***	.9998	.779***	1.006	.797	.242	N=41,069
	[.734, .753]	[.975, 1.039]	[1.032, 1.064]	[.962, 1.04]	[.769, .788]	[.978, 1.036]	[.79, .803]	[.227, .256]	
Philippines	.763***	.995	1.073***	.994	.819***	.989	.816	.34	N=36,225
	[.754, .773]	[.969, 1.022]	[1.056, 1.09]	[.961, 1.027]	[.809, .829]	[.964, 1.014]	[.808, .823]	[.319, .361]	
South Africa	.877***	1.03	1.025*	.984	.899***	1.013	.901	.332	N=3,073
	[.858, .896]	[.957, 1.108]	[.9958, 1.056]	[.904, 1.07]	[.882, .915]	[.958, 1.071]	[.888, .913]	[.282, .383]	
Tajikistan	.766***	.969		.946	.766***	.917	.857	.441	N=400
	[.727, .807]	[.816, 1.151]		[.744, 1.203]	[.727, .807]	[.759, 1.108]	[.823, .891]	[.346, .535]	

	Constant	Washer	Female	Interaction	Constant× Female	Interaction× Washer	Mean school attendance	Mean washer ownership	
Türkiye	.8***	.974	1.019	1.125	.816***	1.096	.84	.97	N=3,765
	[.78, .822]	[.829, 1.144]	[.981, 1.059]	[.933, 1.357]	[.795, .838]	[.923, 1.3]	[.823, .857]	[.959, .981]	
**Female-disadvantage**									
Pooled	.836***	1.003	.983**	.987	.822***	.99	.852	.509	N=377,107
	[.826, .846]	[.982, 1.023]	[.97, .9962]	[.961, 1.014]	[.812, .832]	[.966, 1.014]	[.845, .86]	[.488, .53]	
Albania	.819***	1.15*	.987	.89	.809***	1.023	.844	.896	N=2,434
	[.794, .846]	[.987, 1.339]	[.947, 1.03]	[.763, 1.039]	[.783, .836]	[.908, 1.154]	[.821, .868]	[.869, .923]	
Armenia	.811***	.938		.975	.811***	.915	.866	.935	N=1,044
	[.768, .856]	[.832, 1.058]		[.786, 1.21]	[.768, .856]	[.763, 1.098]	[.839, .893]	[.902, .967]	
Azerbaijan	.793***	1.053	.973	1.138	.772***	1.198	.849	.0468	N=870
	[.755, .834]	[.85, 1.304]	[.903, 1.049]	[.818, 1.583]	[.742, .804]	[.95, 1.51]	[.817, .88]	[.0182, .0755]	
Colombia	.8***	.9984	.994	1.006	.795***	1.004	.828	.461	N=25,792
	[.789, .811]	[.974, 1.023]	[.976, 1.012]	[.975, 1.038]	[.784, .807]	[.979, 1.03]	[.819, .836]	[.443, .48]	
Egypt	.785***	1.014	.968***	.973*	.76***	.987	.803	.833	N=36,018
	[.776, .793]	[.991, 1.038]	[.954, .983]	[.947, 1.001]	[.75, .769]	[.965, 1.01]	[.795, .811]	[.823, .843]	
Gabon	.91***	1.031	.963***	.965	.876***	.995	.902	.0194	N=4,066
	[.896, .923]	[.949, 1.12]	[.94, .987]	[.836, 1.114]	[.86, .893]	[.917, 1.079]	[.888, .915]	[.0102, .0286]	
Guatemala	.761***	1.021	.969	.966	.737***	.985	.788	.258	N=5,959
	[.738, .785]	[.959, 1.086]	[.923, 1.017]	[.892, 1.045]	[.714, .761]	[.926, 1.049]	[.77, .806]	[.219, .296]	
Guyana	.716***	1.048	1.009	.949	.723***	.995	.793	.249	N=1,594
	[.674, .76]	[.954, 1.152]	[.932, 1.093]	[.822, 1.095]	[.683, .764]	[.892, 1.11]	[.763, .824]	[.202, .297]	
India	.791***	.99	.967***	1.012	.765***	1.001	.816	.149	N=243,598
	[.787, .795]	[.976, 1.003]	[.961, .974]	[.9955, 1.028]	[.761, .77]	[.987, 1.015]	[.813, .819]	[.144, .154]	
Indonesia	.742***	1.002	.989	.954	.734***	.956*	.79	.367	N=5,009
	[.718, .767]	[.944, 1.064]	[.941, 1.039]	[.888, 1.025]	[.706, .762]	[.907, 1.008]	[.771, .808]	[.321, .414]	
Kyrgyz Republic	.892***	.964*	1.013	1.006	.903***	.97	.915	.616	N=3,133
	[.871, .912]	[.926, 1.004]	[.985, 1.042]	[.961, 1.053]	[.887, .92]	[.929, 1.013]	[.895, .934]	[.569, .662]	
Morocco	.614***	1.038	.974	.874	.598***	.907	.678	.244	N=1,259
	[.583, .647]	[.882, 1.222]	[.892, 1.063]	[.721, 1.059]	[.57, .628]	[.799, 1.03]	[.62, .737]	[.157, .331]	
Pakistan	.643***	1.157*	.897***	1.073	.577***	1.241**	.68	.774	N=5,133
	[.618, .669]	[.977, 1.369]	[.829, .97]	[.866, 1.33]	[.544, .612]	[1.028, 1.499]	[.635, .725]	[.719, .829]	
Peru	.8***	.967*	.962***	1.02	.769***	.987	.817	.102	N=25,789
	[.79, .81]	[.93, 1.006]	[.946, .978]	[.963, 1.08]	[.758, .781]	[.942, 1.033]	[.809, .826]	[.0909, .113]	
Philippines	.791***	.968	.993	1.027	.786***	.995	.822	.489	N=2,278
	[.761, .821]	[.903, 1.038]	[.942, 1.048]	[.944, 1.118]	[.756, .816]	[.929, 1.065]	[.797, .847]	[.43, .548]	
South Africa	.943***	1.008	.982*	.9988	.926***	1.007	.94	.335	N=2,915
	[.931, .955]	[.981, 1.036]	[.962, 1.002]	[.959, 1.04]	[.912, .941]	[.975, 1.04]	[.93, .95]	[.302, .369]	
Tajikistan	.897***	.984	.947***	1.011	.85***	.9952	.893	.685	N=2,824
	[.882, .913]	[.938, 1.033]	[.917, .979]	[.938, 1.09]	[.828, .872]	[.935, 1.06]	[.877, .91]	[.644, .726]	

	Constant	Washer	Female	Interaction	Constant× Female	Interaction× Washer	Mean school attendance	Mean washer ownership	
Türkiye	.757***	1.047	.949***	1.085	.718***	1.136	.781	.945	N=7,392
	[.739, .775]	[.921, 1.191]	[.916, .982]	[.901, 1.307]	[.697, .74]	[.964, 1.34]	[.766, .797]	[.933, .958]	
**Large female-disadvantage**									
Pooled	.778***	1.009	.883***	1.027*	.687***	1.036***	.771	.371	N=679,003
	[.769, .787]	[.992, 1.027]	[.87, .896]	[.9968, 1.057]	[.676, .698]	[1.009, 1.064]	[.764, .779]	[.355, .387]	
Albania	.747***	.9964	.905***	1.104**	.676***	1.1**	.766	.71	N=4,688
	[.718, .776]	[.934, 1.063]	[.856, .956]	[1.01, 1.207]	[.649, .703]	[1.022, 1.184]	[.746, .785]	[.672, .748]	
Armenia	.763***	1.03		1.012	.763***	1.042	.81	.806	N=3,149
	[.736, .791]	[.972, 1.092]		[.914, 1.12]	[.736, .791]	[.955, 1.138]	[.792, .827]	[.775, .837]	
Azerbaijan	.888***	1.014	.921***	.9978	.818***	1.011	.873	.219	N=5,333
	[.871, .905]	[.979, 1.049]	[.892, .951]	[.935, 1.065]	[.8, .836]	[.946, 1.081]	[.858, .887]	[.189, .25]	
Colombia	.806***	1.016	.955	1.09	.77***	1.107**	.828	.418	N=1,672
	[.776, .838]	[.932, 1.107]	[.901, 1.012]	[.957, 1.24]	[.739, .802]	[1.012, 1.21]	[.806, .85]	[.372, .463]	
Egypt	.71***	1.042***	.776***	1.084***	.551***	1.129***	.688	.79	N=45,350
	[.701, .718]	[1.012, 1.073]	[.76, .793]	[1.034, 1.136]	[.539, .563]	[1.084, 1.176]	[.679, .696]	[.779, .801]	
Gabon	.915***	.925	.902***	.991	.826***	.917	.892	.0116	N=1,510
	[.894, .937]	[.809, 1.058]	[.85, .958]	[.768, 1.279]	[.79, .863]	[.755, 1.114]	[.867, .918]	[.00226, .0209]	
Guatemala	.625***	.956	.827***	.9999	.517***	.956	.643	.0559	N=18,494
	[.609, .642]	[.893, 1.022]	[.799, .856]	[.891, 1.122]	[.5, .535]	[.872, 1.048]	[.629, .657]	[.046, .0659]	
Guyana	.688***	.982	.863***	.979	.594***	.962	.747	.143	N=2,337
	[.647, .732]	[.896, 1.077]	[.791, .941]	[.815, 1.175]	[.556, .634]	[.825, 1.12]	[.716, .779]	[.105, .182]	
India	.742***	.985**	.903***	1.004	.67***	.989	.752	.0557	N=483,316
	[.739, .745]	[.974, .9971]	[.898, .907]	[.989, 1.02]	[.667, .673]	[.976, 1.003]	[.749, .754]	[.0538, .0575]	
Indonesia	.794***	1.009	.951	.937	.755***	.946	.826	.287	N=913
	[.763, .826]	[.933, 1.092]	[.883, 1.023]	[.823, 1.067]	[.716, .795]	[.833, 1.074]	[.797, .856]	[.222, .353]	
Kyrgyz Republic	.881***	1.037		.87	.881***	.902	.89	.56	N=103
	[.803, .967]	[.959, 1.121]		[.552, 1.37]	[.803, .967]	[.609, 1.335]	[.809, .971]	[.413, .706]	
Morocco	.514***	.929	.692***	.942	.356***	.876**	.527	.0716	N=9,308
	[.492, .537]	[.839, 1.029]	[.653, .733]	[.813, 1.092]	[.334, .378]	[.781, .981]	[.497, .556]	[.0511, .0921]	
Pakistan	.485***	.975	.618***	1.034	.3***	1.009	.473	.417	N=56,738
	[.473, .499]	[.937, 1.016]	[.595, .641]	[.977, 1.095]	[.289, .311]	[.959, 1.061]	[.459, .488]	[.397, .438]	
Peru	.756***	1.074**	.881***	.99	.666***	1.063*	.763	.0528	N=15,995
	[.741, .77]	[1.011, 1.14]	[.86, .903]	[.9, 1.088]	[.649, .683]	[.991, 1.14]	[.749, .776]	[.042, .0636]	
Philippines	.819***	1.107	.868***	1.018	.712***	1.127	.795	.269	N=693
	[.786, .854]	[.973, 1.26]	[.79, .954]	[.848, 1.222]	[.669, .758]	[.943, 1.348]	[.756, .833]	[.185, .354]	
South Africa	.93***	1.067*	.901***	1.006	.838***	1.073	.903	.48	N=1,012
	[.905, .956]	[.994, 1.145]	[.857, .947]	[.909, 1.114]	[.808, .869]	[.976, 1.18]	[.882, .924]	[.405, .555]	
Tajikistan	.867***	.9991	.857***	.988	.743***	.987	.843	.207	N=13,320
	[.855, .878]	[.975, 1.024]	[.835, .88]	[.949, 1.029]	[.726, .76]	[.956, 1.019]	[.833, .853]	[.185, .229]	

	Constant	Washer	Female	Interaction	Constant× Female	Interaction× Washer	Mean school attendance	Mean washer ownership	
Türkiye	.685***	1.002	.791***	1.291***	.542***	1.294***	.687	.79	N=15,072
	[.669, .702]	[.954, 1.052]	[.762, .82]	[1.185, 1.406]	[.525, .559]	[1.194, 1.401]	[.674, .7]	[.768, .812]	

Notes: *P<0.1; **P<0.05; ***P<0.01. Rate ratios and a constant from Poisson regression models are shown, in addition to two combinations obtained using post estimation. Each row of estimates was obtained from a separate model. All models included a baseline term for being female and baseline terms and interactions with being female for washer ownership, fridge ownership, TV ownership, having flush toilet, a wealth index z-score, number of household members, number of household members under age five, age, highest education level of a male in household, and highest education level of a female in household, as well as adjusting for neighborhood level factors. Except the baseline term for female, all independent variables were centered around a country-specific weighted mean: Therefore, the 'Constant' column shows school attendance for males with the mean on all other independent variables. The column labelled 'Washer' shows differences in school attendance for males with washer at home compared to males without washer. The column labelled 'Interaction' shows interaction terms for females and washer. The column labelled 'Constant×Female' shows school attendance for females with the mean on all other independent variables, obtained using post estimation. The column labelled 'Interaction×Washer' shows difference in school attendance for girls with washer at home compared to girls without washer, obtained using post estimation. Means for school attendance and washer ownership are also shown. Estimates were weighted using sampling weights, rescaled to sum up to one for the final sample from each survey. Pooled models were further rescaled such that each country contributed equally to the estimates.95% confidence intervals adjusted for clustering at the level of primary sampling units are shown in brackets below the point estimates.

Table S29. Results from Poisson regression models of school attendance on being female interacted with washer ownership adjusting for all household level factors

	Constant	Female	Interaction	Constant× Female	Interaction× Female	
Pooled	.787***	.994	1.022**	.782***	1.016	N=805,796
	[.779, .795]	[.983, 1.005]	[1.001, 1.044]	[.774, .79]	[.992, 1.041]	
Albania	.793***	1.014	1.088	.804***	1.103*	N=5,417
	[.77, .816]	[.976, 1.053]	[.979, 1.209]	[.784, .825]	[.995, 1.224]	
Armenia	.802***	1.081***	1.016	.866***	1.097**	N=4,263
	[.779, .825]	[1.045, 1.118]	[.943, 1.094]	[.849, .884]	[1.007, 1.196]	
Azerbaijan	.898***	.92***	.974	.826***	.896***	N=2,819
	[.875, .921]	[.884, .957]	[.916, 1.036]	[.799, .853]	[.843, .953]	
Colombia	.73***	1.066***	1.028	.778***	1.096***	N=25,388
	[.716, .744]	[1.04, 1.092]	[.989, 1.069]	[.766, .79]	[1.04, 1.155]	
Egypt	.753***	.908***	1.044***	.683***	.948***	N=58,851
	[.746, .76]	[.896, .92]	[1.017, 1.073]	[.675, .692]	[.921, .977]	
Gabon	.912***	1.004	1.01	.916***	1.014	N=4,583
	[.889, .936]	[.973, 1.036]	[.905, 1.126]	[.897, .936]	[.909, 1.131]	
Guatemala	.63***	.865***	1.008	.545***	.873***	N=13,701
	[.612, .649]	[.835, .897]	[.929, 1.094]	[.527, .564]	[.798, .954]	
Guyana	.664***	1.081**	.986	.718***	1.066	N=3,129
	[.63, .7]	[1.014, 1.152]	[.878, 1.108]	[.686, .751]	[.928, 1.225]	
India	.756***	.967***	1.003	.731***	.97***	N=535,646
	[.753, .759]	[.963, .971]	[.992, 1.015]	[.728, .734]	[.958, .982]	
Indonesia	.749***	1.043***	1.011	.782***	1.055**	N=12,101
	[.733, .765]	[1.015, 1.073]	[.967, 1.057]	[.766, .797]	[1.001, 1.112]	
Kyrgyz Republic	.879***	1.047***	1.036	.921***	1.085***	N=2,748
	[.858, .901]	[1.018, 1.077]	[.99, 1.083]	[.902, .939]	[1.025, 1.148]	
Moldova	.807***	1.08***	1.047	.872***	1.13***	N=1,644
	[.777, .839]	[1.037, 1.125]	[.963, 1.137]	[.847, .897]	[1.033, 1.236]	
Morocco	.55***	.8***	.925	.44***	.74***	N=7,994
	[.527, .573]	[.758, .845]	[.812, 1.053]	[.417, .464]	[.646, .848]	
Pakistan	.517***	.69***	1.066**	.357***	.736***	N=48,732
	[.503, .531]	[.668, .713]	[1.004, 1.132]	[.344, .37]	[.688, .787]	
Peru	.745***	1.01	1.021	.752***	1.031	N=34,040
	[.735, .755]	[.993, 1.027]	[.966, 1.079]	[.742, .763]	[.972, 1.093]	
Philippines	.742***	1.114***	.994	.827***	1.107***	N=19,973
	[.73, .754]	[1.094, 1.135]	[.953, 1.036]	[.816, .838]	[1.057, 1.16]	
South Africa	.913***	.992	1.055	.905***	1.047	N=2,704
	[.894, .932]	[.966, 1.019]	[.985, 1.13]	[.886, .925]	[.973, 1.126]	
Tajikistan	.876***	.872***	.993	.764***	.866***	N=8,830

	Constant	Female	Interaction	Constant× Female	Interaction× Female	
	[.863, .888]	[.849, .896]	[.952, 1.035]	[.744, .784]	[.824, .91]	
Türkiye	.685***	.831***	1.315***	.569***	1.093*	N=13,233
	[.667, .702]	[.802, .861]	[1.193, 1.449]	[.551, .588]	[.992, 1.203]	

Notes: *P<0.1; **P<0.05; ***P<0.01. Rate ratios and a constant from Poisson regression models are shown, in addition to two combinations obtained using post estimation. Each row of estimates was obtained from a separate model. All models included a baseline term for being female and age and interactions with being female for washer ownership, fridge ownership, TV ownership, having flush toilet, a wealth index z-score, number of household members, number of household members under age five, age, highest education level of a male in household, and highest education level of a female in household, as well as adjusting for household level factors using household level means of all independent variables (baseline and interactions with being female). Except the baseline term for female, all independent variables were centered around a country-specific weighted mean: Therefore, the 'Constant' column shows school attendance for males with the mean on all other independent variables. The column labelled 'Female' shows differences in school attendance for females without a washer at home compared to boys without a washer at home. The column labelled 'Interaction' shows interaction terms for females and having a washer. The column labelled 'Constant×Female' shows school attendance for females with the mean on all other independent variables, obtained using post estimation. The column labelled 'Interaction×Washer' shows difference in school attendance for girls with washer at home compared to boys with washer at home, obtained using post estimation. Households without at least one girl and one boy were excluded. Estimates were weighted using sampling weights, rescaled to sum up to one for the final sample from each survey. Pooled models were further rescaled such that each country contributed equally to the estimates.95% confidence intervals adjusted for clustering at the level of primary sampling units are shown in brackets below the point estimates.
